# Mobile Type VI secretion system loci of the gut Bacteroidales display extensive intra-ecosystem transfer, multi-species spread and geographical clustering

**DOI:** 10.1371/journal.pgen.1009541

**Published:** 2021-04-26

**Authors:** Leonor García-Bayona, Michael J. Coyne, Laurie E. Comstock

**Affiliations:** Division of Infectious Diseases, Brigham and Women’s Hospital, Harvard Medical School, Boston, Massachusetts, United States of America; Swiss Federal Institute of Technology Lausanne (EPFL), SWITZERLAND

## Abstract

The human gut microbiota is a dense microbial ecosystem with extensive opportunities for bacterial contact-dependent processes such as conjugation and Type VI secretion system (T6SS)-dependent antagonism. In the gut Bacteroidales, two distinct genetic architectures of T6SS loci, GA1 and GA2, are contained on Integrative and Conjugative Elements (ICE). Despite intense interest in the T6SSs of the gut Bacteroidales, there is only a superficial understanding of their evolutionary patterns, and of their dissemination among Bacteroidales species in human gut communities. Here, we combine extensive genomic and metagenomic analyses to better understand their ecological and evolutionary dynamics. We identify new genetic subtypes, document extensive intrapersonal transfer of these ICE to Bacteroidales species within human gut microbiomes, and most importantly, reveal frequent population fixation of these newly armed strains in multiple species within a person. We further show the distribution of each of the distinct T6SSs in human populations and show there is geographical clustering. We reveal that the GA1 T6SS ICE integrates at a minimal recombination site leading to their integration throughout genomes and their frequent interruption of genes, whereas the GA2 T6SS ICE integrate at one of three different tRNA genes. The exclusion of concurrent GA1 and GA2 T6SSs in individual strains is associated with intact T6SS loci and with an ICE-encoded gene. By performing a comprehensive analysis of mobile genetic elements (MGE) in co-resident Bacteroidales species in numerous human gut communities, we identify 74 MGE that transferred to multiple Bacteroidales species within individual gut microbiomes. We further show that only three other MGE demonstrate multi-species spread in human gut microbiomes to the degree demonstrated by the GA1 and GA2 ICE. These data underscore the ubiquity and dissemination of mobile T6SS loci within Bacteroidales communities and across human populations.

## Author summary

Bacteroidales are abundant members of the gut microbiota of human populations across the globe. Bacteroidales use Type VI secretion systems (T6SS) to deliver toxins to neighboring cells to gain a competitive advantage. Bacteroidales T6SS are of three different genetic architectures, two of which are contained on mobile genetic elements (MGEs). Due to the phenotypes conferred by cargo genes contained on MGE, their dissemination greatly impacts the ecology and evolution of human gut microbial communities. Here, we present a comprehensive analysis of the prevalence and dissemination of these mobile T6SS loci in gut Bacteroidales species across human populations. We reveal extensive dissemination of T6SS loci to numerous Bacteroidales species in human gut microbiomes with these newly armed strains outcompeting their progenitors. Few other Bacteroidales MGE spread in as many human communities to the degree detected for the T6SS MGEs. Considering the expected cost associated with maintaining these MGEs, these observations suggest that their acquisition confers a significant benefit, increasing fitness of individual strains and possibly the Bacteroidales community as a whole.

## Introduction

The order Bacteroidales encompasses numerous genera including the *Bacteroides*, *Parabacteroides* and *Prevotella*, which collectively are the most abundant Gram-negative bacteria of the healthy colonic microbiota of human populations. These bacteria secrete anti-bacterial proteins that antagonize closely related strains and species, providing a competitive advantage in the gut ecosystem (reviewed [1]). Type VI secretion systems (T6SSs) are also antagonistic systems of these gut bacteria. T6SSs are contractile nanomachines that inject toxic effectors in a contact-dependent manner into bacterial or eukaryotic cells (reviewed [2]). There are three distinct genetic architectures of T6SS in gut Bacteroidales termed genetic architecture 1, 2 and 3 (GA1, GA2, and GA3) [3]. GA3 T6SS loci are found exclusively and at high proportion in *B*. *fragilis* strains, and contain two variable regions containing genes encoding effector and immunity proteins [3]. The effectors of distinct GA3 T6SS have potent killing activity [4–6], targeting nearly all gut Bacteroidales species analyzed [4]. GA3 T6SSs were shown to be enriched among strains colonizing the infant gut and associated with increased abundance of *Bacteroides* in the human gut microbiota [7].

The GA1 and GA2 T6SS loci are contained on Integrative and Conjugative Elements (ICE) and are present in diverse Bacteroidales species [3]. In other bacterial lineages, T6SS loci are typically contained on non-core genomic islands, but, with few exceptions [8], are rarely found on conjugative elements. Some T6SS-associated genes, such as immunity genes [9], reside on mobile elements, and a full T6SS locus is present on a mobile prophage-like element in environmental *Vibrio cholerae* strains [10]. The presence of complete Bacteroidales T6SS loci on ICE allows for their distribution to other co-resident Bacteroidales species in the human gut.

We previously identified 48 human gut Bacteroidales strains of 13 different species that contain GA1 T6SSs. The ICE containing the GA1 T6SS loci are approximately 95% identical at the DNA level between different strains. We previously showed that GA1-containing ICE were transferred to several Bacteroidales species in the gut of two human subjects [3, 11]. Like the GA3 T6SSs, the GA1 T6SS loci contain two variable regions that encode identifiable effector and immunity proteins [3]. To date, the target cells antagonized by the GA1 T6SSs have not been conclusively identified.

The GA2-containing ICE are distinct from the ICE containing GA1 T6SS loci. ICEs containing GA2 T6SS are less identical to each other than are the GA1 ICE. The GA2 T6SSs contain three variable regions with genes encoding potential effector and immunity proteins. Unlike the GA1 T6SS loci, we did not previously detect the same GA2 T6SS in multiple species of an individual, and therefore had no evidence of its transfer to co-resident species within the human gut microbiota. Although *B*. *fragilis* strains can harbor both a GA1 and a GA3 T6SS locus, we did not identify a Bacteroidales strain that harbors a GA2 T6SS locus along with either a GA1 or GA3 T6SS locus, suggesting exclusion.

The present study was designed to address numerous important and outstanding questions regarding the T6SS of the gut Bacteroidales by in-depth analyses of genomic and metagenomic data. In this study, we document five sub-types of GA2 T6SS, find that GA2 T6SS loci are globally dominant and that the ICE containing them transfer to multiple species within a gut microbiota with subsequent fixation in the population (the cells that receive the ICE increase vastly in frequency relative to non-carriers) [12]. We identify the insertion recognition sequences of both GA1 and the different GA2 subtype ICE and find their exclusion is associated with both T6SS genes and a gene encoding a protein with both *N*^6^-adenine methylase and SNF2 helicase domains. In addition, we identify 74 mobile genetic elements that transfer to multiple Bacteroidales species of an individual, but few to the extent of the GA1 and GA2 ICE, that commonly spread in Bacteroidales communities in the human gut of diverse populations.

## Results and discussion

### Identification of five different GA2 subtypes

**Fig 1:**
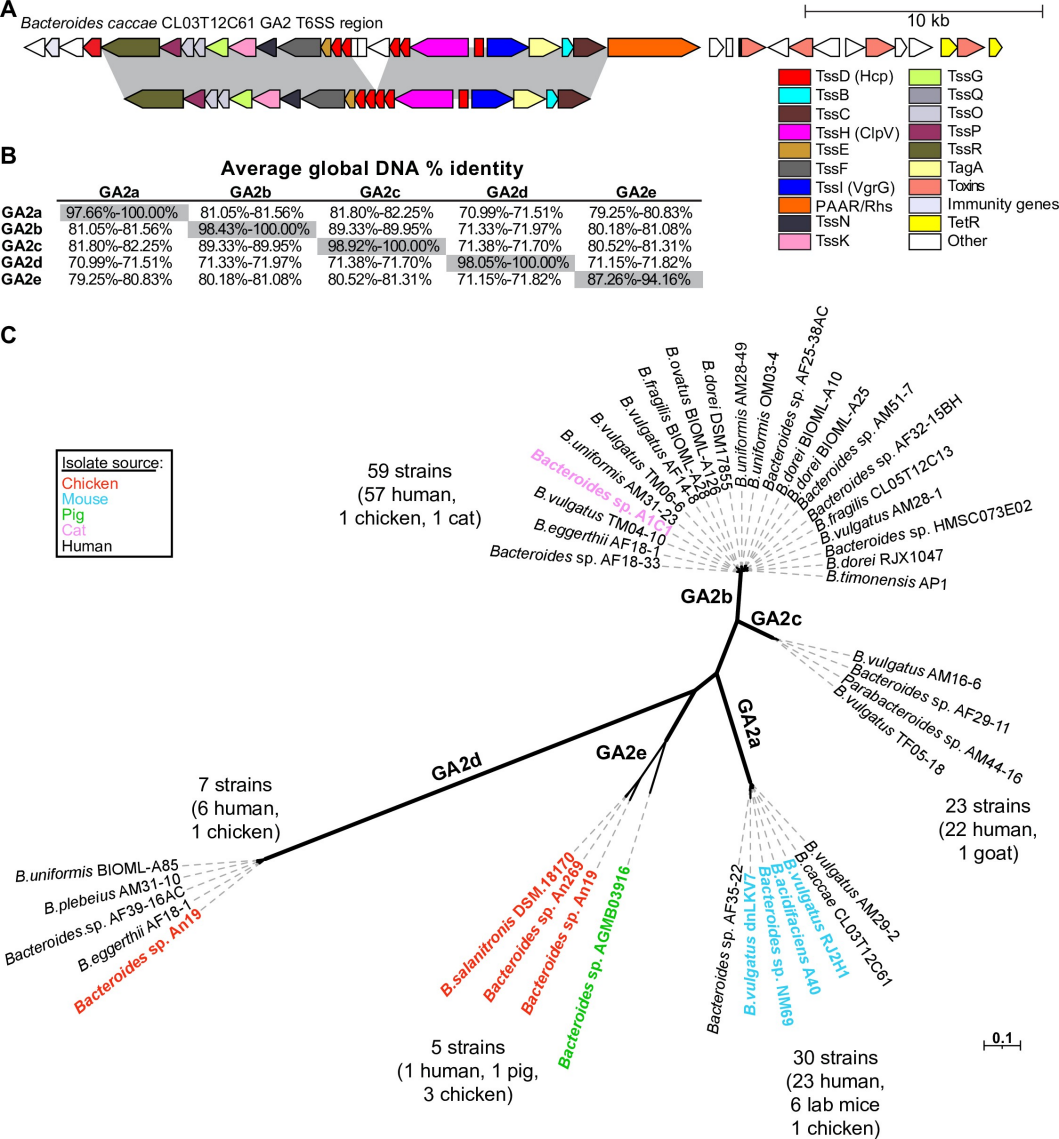
GA2 segregate into five different subtypes. **A.** Schematic of the GA2 locus of *B*. *caccae* CL03T12C61 as example. Segments highlighted in gray represent the conserved regions made into a concatemer used for the analyses shown in panels B and C. **B.** Average global DNA percent identity between the conserved concatemers of different GA2 subtypes. **C.** Maximum likelihood phylogenetic tree of the GA2 regions showing the separation into 5 subtypes. Only some strains are shown, with the numbers at the end of each branch indicating the total number of isolate genomes, out of the 1434 Bacteroidales genomes queried that contain that subtype of GA2. Some isolates from non-human sources are highlighted in colored font.

Unlike the GA1 and GA3 T6SS loci that are highly identical (~95%) outside of the effector and immunity gene regions, the GA2 T6SS loci are more variable with less than 80% DNA identity between some loci [3]. To better study this variability, we compared the conserved regions (excluding the effector and immunity genes, Fig 1A) of 45 different GA2 loci and found that they segregate into 5 distinct subtypes (GA2a-e). With the exception of GA2e, DNA-level identities within a subtype are high (>97%), whereas cross-type identities generally run in the low 80% (subtypes GA2b and GA2c are more alike, demonstrating ~89% identity) (Fig 1B). The sequence polymorphisms among the subtypes are not clustered but rather distributed across the length of the T6SS region (S1 and S2 Figs). Each GA2 subtype clearly segregates to a distinct branch of a phylogenetic tree (Fig 1C), yet each retains the gene order and predicted functions characteristic of the GA2 architecture, further supporting this subtyping.

### Prevalence of GA1, GA2, and GA3 T6SS loci in sequenced Bacteroidales isolates from human, animal, and environmental sources

**Fig 2:**
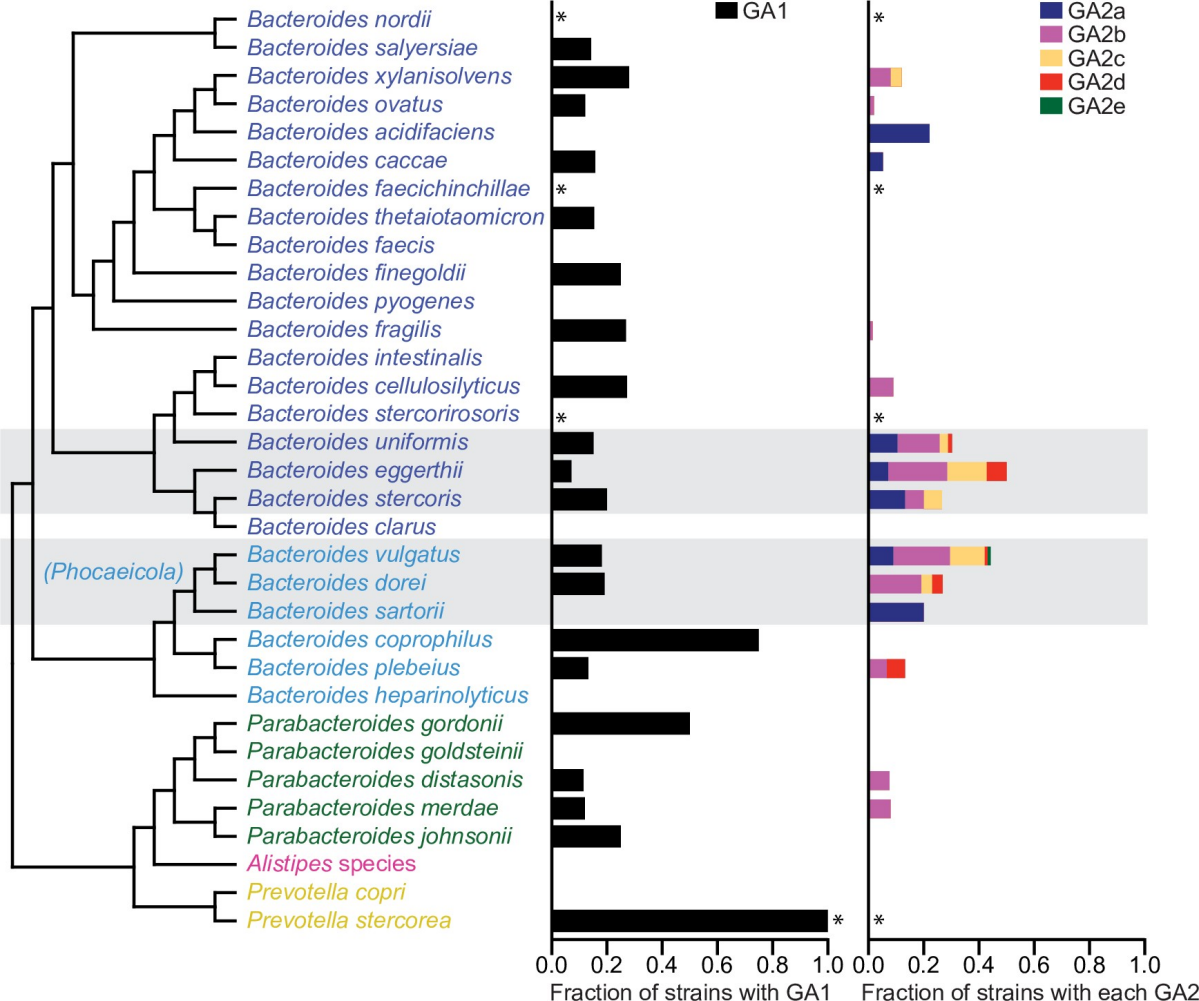
Abundance of GA1 and GA2 loci in different Bacteroidales species. Shown is the fraction of strains within each species harboring each type of GA. *Three or less sequenced isolates. Strains with no species call were not included. Shaded boxes highlight the species with high GA2 prevalence.

Our initial report of the prevalence of the three distinct genetic architectures of T6SSs in human gut Bacteroidales was performed with 205 human gut Bacteroidales genome sequences that were available in 2015 [3]. To provide a more comprehensive analysis, we include here genome sequences of all Bacteroidales strains from any source available as of June, 2020. We attempted, with the information available to us, to include only genomes from isolated bacteria excluding those assembled from metagenomic sequences, and to reduce redundant genomes (such as multiple longitudinal isolates of the same strain from the same person or other identical strains sequenced multiple times). The final set includes 1434 Bacteroidales genomes of 14 different families and 41 different genera S1 Table). These genomes were queried with concatemers of GA1, GA2a, GA2b, GA2c, GA2d, GA2e, and GA3 T6SS loci with the divergent genes removed (S1 Fig). Of these 41 genera, we detected GA1, GA2, and GA3 T6SSs in only three genera, *Bacteroides*, *Parabacteroides* and *Prevotella*, and nearly all of these strains were from gut sources (S1 Table). As previously described, and reinforced by this analysis, the GA3 T6SSs are present exclusively in *B*. *fragilis*, with 93 of the 126 *B*. *fragilis* genomes analyzed here containing a GA3 T6SS locus. The GA1 T6SSs loci were found to be relatively evenly distributed among *Bacteroides* and *Parabacteroides* genomes, with no obvious species preference (Fig 2); however, none of the 26 *Prevotella copri* genomes contains a GA1 locus, whereas both of the sequenced *P*. *stercorea* genomes do. In our previous analysis of 205 genomes, only nine genomes were found to contain a GA2 T6SS loci, suggesting then that GA2 loci may be rare in gut Bacteroidales. This expanded analysis shows that there are nearly as many GA2 loci (124) as GA1 loci (129) detectable in these sequenced strains. Unlike the GA1 loci, this analysis reveals a GA2 species-level distribution bias, with GA2 loci being found at high prevalence in some species [*B*. *eggerthii* (43% of strains), *B*. *vulgatus* (40%), *B*. *uniformis* (30*%)*, *B*. *stercoris* (23%), and *B*. *dorei* (26%)] and more rarely in other species [*B*. *ovatus* (2%), *B*. *fragilis* (1.6%), *B*. *thetaiotaomicron* (0%), *B*. *intestinalis* (0%)] (Fig 2 and S1 Table). Among the GA2 subtypes, GA2b is the most prevalent in this genome set, present in 62 genomes, followed by GA2a (26 genomes), GA2c (24 genomes), GA2d (7 genomes), and GA2e (5 genomes).

The absence of GA1 and GA2 loci in non-gut Bacteroidales species, such as those that occupy the oral cavity or vagina (S1 Table), could be due to lack of cell-cell contacts, but both oral and vaginal Bacteroidales can infrequently colonize the gut and even at low frequency, transfers to Bacteroidales in other ecosystems would be expected to occur. This observation suggests a fitness advantage conferred by the T6SSs that is unique to gut species. An interesting finding is the presence of some T6SS loci in the genomes of non-human isolates. Of the five GA2e loci detected, only one is from a human isolate, three are present in isolates from the ceca of chicken, and one from the stool of a pig (S1 Table). One of the seven GA2d loci is from a strain isolated from a chicken. Of the 26 isolates with GA2a loci, six are mouse isolates, and of the 62 strains with GA2b loci, one was isolated from a cat, and one from a chicken. These data suggest that the GA2e subtype is of non-human origin as only one human isolate contained a GA2e locus. In contrast, the few non-human strains containing the other four GA2 subtypes may have been acquired by these animals from a human source, as they were isolated from domestic or lab animals.

### Intra-ecosystem GA2 ICE transfers to co-resident species

**Fig 3:**
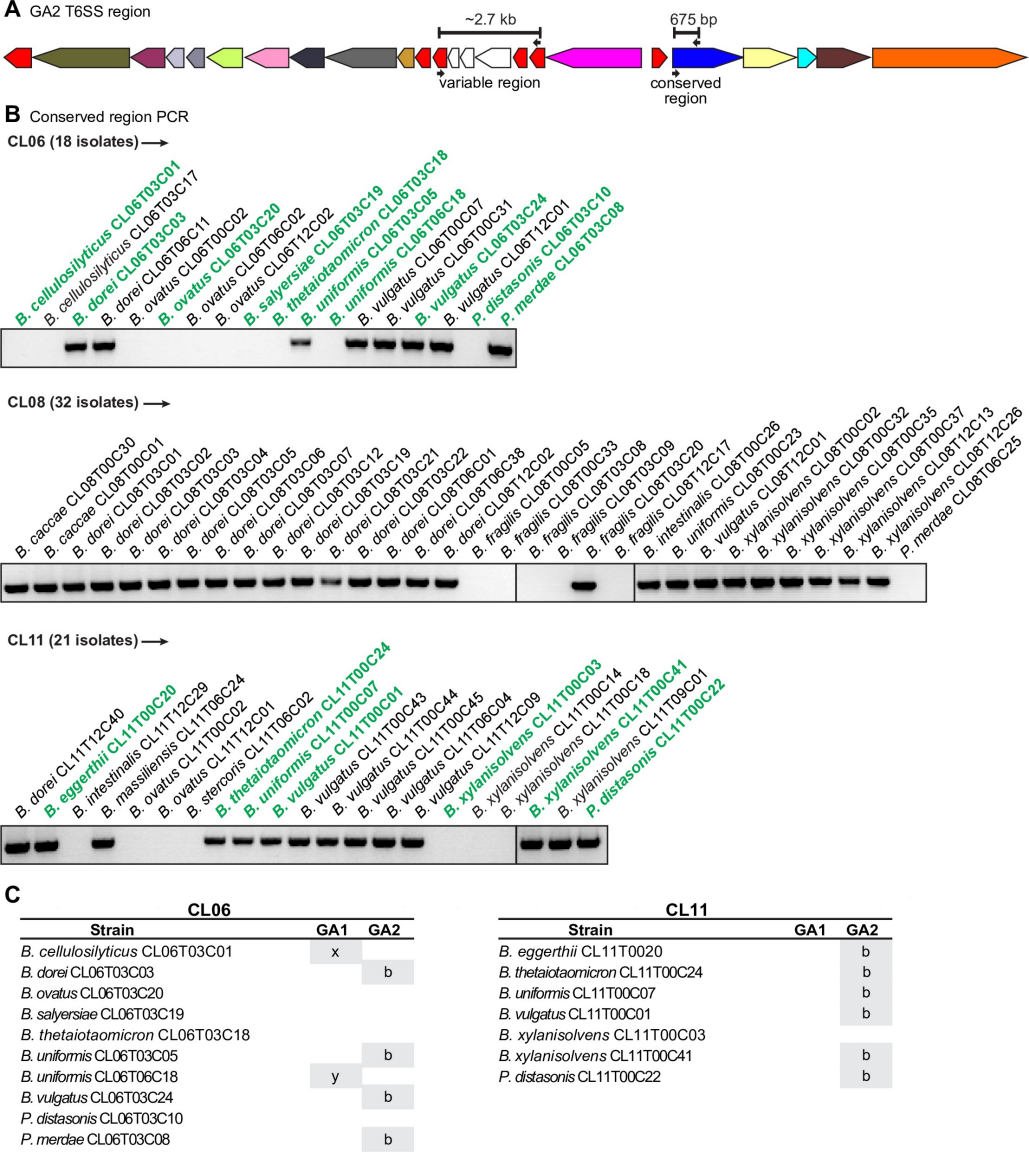
Screen for intra-community GA2 spread in the Comstock lab strain collection. **A.** Schematic of a GA2 T6SS locus. Small arrows indicate screening primer annealing sites and bars indicate the corresponding PCR product. Color scheme of genes is as described in Fig 1. **B.** PCR screen of the isolates from the three communities indicated, using primers for the GA2 conserved region. Strains bolded in green were selected for whole genome sequencing. **C.** Strains sequenced in this study and whether they contain GA1 or GA2 T6SS loci. x and y in GA1 column indicate the regions are distinct.

In our previous analyses of the Bacteroidales strains from four healthy human volunteers, we showed that a GA1 ICE transferred between multiple *Bacteroides* and *Parabacteroides* species in the gut of two of these individuals, such that they have 99.997%-100% nucleotide sequence identity among them [13]. In contrast, GA1 ICE from strains isolated from different individuals are 95–98% identical in the conserved regions. In that prior study, we observed no intra-ecosystem transfer events for GA2 ICEs [3]. To determine if we could identify GA2 ICE transfers among co-resident species in the human gut, we screened *Bacteroides* and *Parabacteroides* strains previously isolated as part of a longitudinal study [14] from four additional healthy human volunteers. Using PCR primers that amplify a 675-bp conserved region of GA2 loci (Fig 3A), we detected GA2 regions in numerous isolates from three of the four communities analyzed: CL06, CL08, and CL11 (Fig 3B). We PCR-amplified a ~2.7 kb variable region of these GA2-positive strains (Fig 3A) and sequenced the amplicons. These DNA regions were identical between different species of the same community, but differed between the three communities.

Using the previously determined species designation of each isolate [14], we selected one strain of each species from communities CL06 and CL11 and sequenced their genomes using SMRT sequencing (Fig 3C). The CL11 ecosystem had six species with GA2b T6SS loci, and comparison of the sequences of their ICE, excluding insertion sequences that frequently insert in these ICE [13], indicated 99.999–100% identity with only one mismatch occurring between any of these ICE. Of note is the identification of a *B*. *thetaiotaomicron* strain (BtCL11T00C24) containing a GA2 locus: this is the first *B*. *thetaiotaomicron* strain identified with a GA2 locus. Of the four CL06 species containing a GA2, all of which are of subtype GA2b, all loci are nearly 100% identical with only one mismatch, excluding a 445 bp duplication within the *tssC* gene in three strains. In contrast, a comparison of CL06 and CL11 GA2b ICE using only high scoring segment pairs of greater than 7500 bp and excluding all non-conserved regions revealed DNA identity of 97.85% with 1567 mismatches.

These data clearly demonstrate that the GA2b ICEs transferred to multiple species within the gut of these two individuals, followed by fixation in the population. For the CL06 community, two species lack the GA2 locus, but each has a distinct GA1 T6SS loci, demonstrating lack of community transfer of these GA1 ICE.

**Fig 4:**
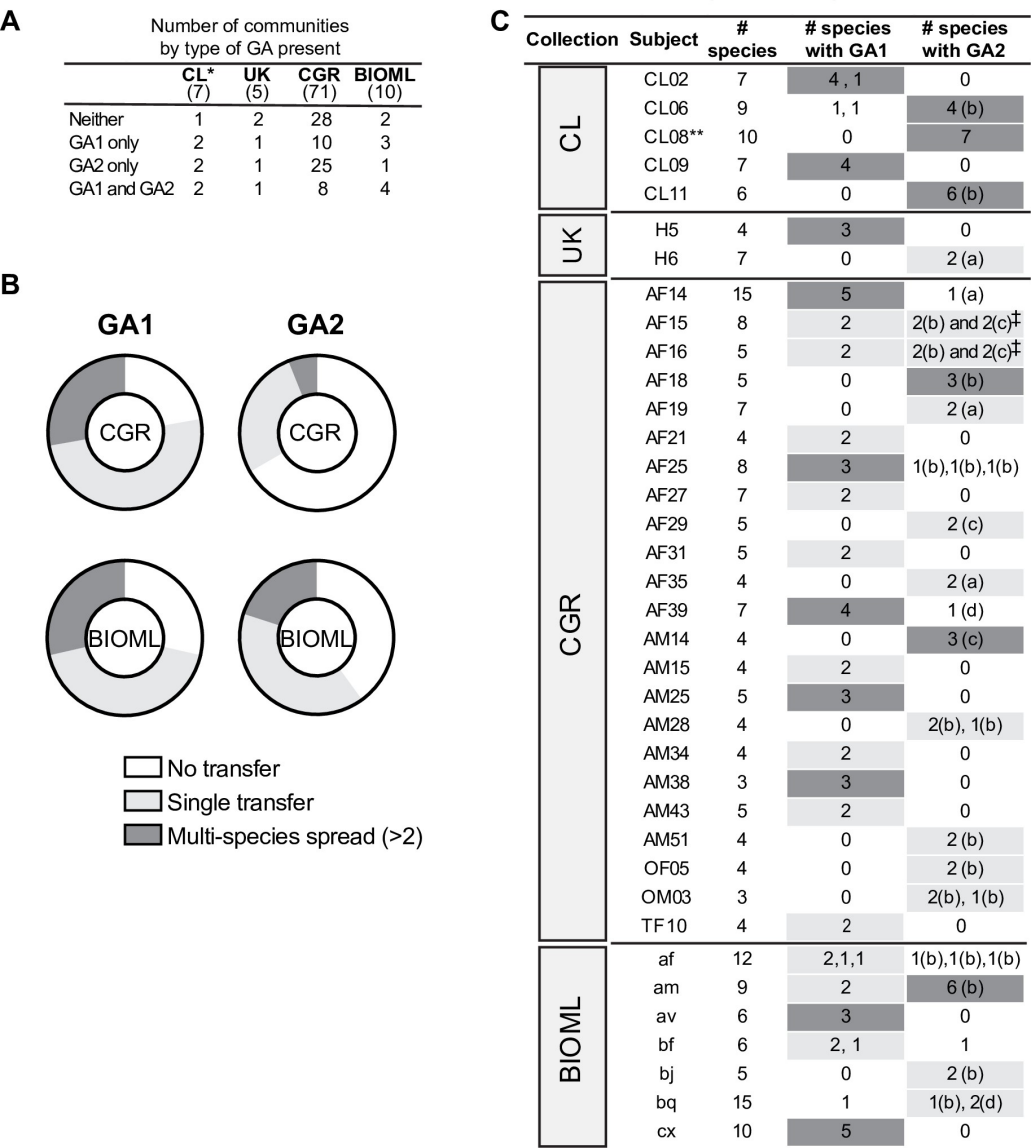
Intra-community spread is common for GA1 and GA2 loci. **A.** Number of communities per dataset, classified by GA1 and GA2 loci presence/absence. Numbers in parentheses under each dataset name indicate total number of volunteers analyzed in that collection. *Includes CL08 and CL14 which were screened by PCR as described in the text. **B.** Percentage of communities in the CGR and BIOML collections that show the indicated event, out of the total communities in that dataset that have at least one strain with the indicated GA type: no transfer, single-species spread (present in two species), multi-species spread (present in three species or more)**. C.** Communities with a single-species intra-community transfer (highlighted in light grey) or multi-species spread (highlighted in dark grey). Letters in parentheses indicate type of GA2. Numbers listed separately in an individual box indicate the T6SS regions are different (<99.99% DNA sequence identity and/or divergent variable regions). **Screened by PCR and sequencing of variable region, as described in the text. Species determined by 16S rRNA gene sequence. ‡The two relevant strains from donor AF15 and AF16 are nearly clonal and appear to have both a GA2b and GA2c (see details in text).

To further study GA1 and GA2 dissemination in a larger set of communities, we analyzed three available datasets of sequenced gut bacterial isolates from healthy adults: the BIOML (longitudinal study from USA) [15], CGR (China) [16] and UK [17] sets. From these datasets, we analyzed the Bacteroidales isolates from subjects from whom at least three different Bacteroidales species were isolated, yielding 10 subjects from the BIOML set, 12 from the UK set, and 71 from the CGR set (Fig 4A and S3 Table). We searched each genome collection for the GA1 T6SS region and for each of the GA2 T6SS subtype regions using the same T6SS region concatemer queries and blastn parameters as were used during analysis of the NCBI genome set. We assumed a recent (i.e. within the lifetime of the participant) intra-host transfer event if shared GA2 DNA regions are >99.99% identical [11, 18]. Shared T6SS regions between two species indicated a single-species spread event, whereas we inferred multi-species spread if the identical region was found in at least three different species from the same community. For GA1, intra-community single-species spread was observed in nine CGR and three BIOML communities (Fig 4, S3 and S4 Tables). For GA2, intra-community single-species spread events were observed in two BIOML, one UK and nine CGR communities. Interestingly, for subject AF15 from the CGR set, two strains (*B*. *uniformis* AF15-14LB and *B*. *vulgatu*s AF15-6A) each contain both a GA2b and a GA2c, integrated at different chromosomal sites. This same phenomenon is observed in individual AF16, where both *B*. *uniformis* AF16-7 and *B*. *vulgatus* AF16-11 have very high sequence similarity across the whole genome with their AF15 cognates. These data indicate inter-person transmission of bacteria and that the GA2 transfer events occurred prior to passage of these bacteria to at least one of these study subjects.

We determined that GA1 and GA2 multi-species spread (three or more species sharing an identical T6SS) are common (Fig 4). For communities containing a strain with a GA1 locus, multi-species spread occurred in 27.8% of the CGR and 28.6% of the BIOML communities. For communities containing a species with a GA2 locus, 6.1% of the CGR and 20% of the BIOML communities showed evidence of spread, whereas no multi-species spread was observed in the UK communities. Transfer of both GA1 and GA2 to a strain was never observed, suggesting some degree of exclusion. GA1 may be more readily transferred and fixed in a bacterial population than GA2, since for the majority of communities with a GA1, transfer occurred to at least one other species (Fig 4B and 4C). In contrast, a majority of communities from the CGR dataset with a GA2 locus did not show evidence of transfer events. This phenomenon could be partially attributable to the species that are present in a particular community, as GA2 demonstrates a species bias. However, this does not fully account for the observed variation, as species that frequently contain GA2 are present in many of the communities with no transfer. It is therefore possible that transfer and fixation in the population may be limited by specific strain physiology and other ecosystem factors.

The longitudinal BIOML dataset comprises many isolates (often more than 20) of the same species per individual community. In most cases these isolates are nearly isogenic (designated here as the same strain), but sometimes two or more distinct strains of the same species coexist at the same collection time-point [15]. This allowed us to evaluate the completeness of transfer and fixation within the population of a strain. GA1 fixation was complete within a strain (all closely-related isolates had the GA1), with a conserved insertion site, indicating they originated from a single transfer event (discussed in the following section). We observed only one exception, where near-isogenic isolates with and without a GA1 T6SS were detected (S5A and S5C Table, partial fixation bolded in S5C Table). Interestingly, two or more unrelated co-existing strains of the same species often had differences in GA1 presence or absence (S5C Table). That is to say, we observed five instances in which all isolates of one strain within a community had a GA1 while all the isolates of the other co-resident species-matched strain did not. The acquisition of a GA2 did not always lead to full fixation in the population of that strain, as we identified three instances of near isogenic co-resident strains some with and some without the GA2 T6SS (two for a GA2b and one for a GA2d) (S5A and S5B Table). Similar to GA1, we also detected four instances of communities harboring two different co-existing strains of the same species, one with and one without the GA2 T6SS. Finally, we identified four examples of GA2 within-species spread, and one of GA1 within-species spread, where some of the isolates of one strain lost a large part of the T6SS region, while in others it remained intact. All together, these results show that both T6SS genetic architectures contained on ICEs often transfer horizontally in the gut and are fixed in the population of multiple species. We were unable to detect events in the BIOML collection where a species or strain previously devoid of a T6SS acquired one during the sampling time, perhaps due to the short sampling time of 1.5 years or less. Individual transfer events may be infrequent or may occur early upon strain acquisition or during population bottlenecks.

Acquisition of an ICE carries with it the cost of maintaining a large genetic element, but the frequent nature of ICE spread suggests that the fitness benefit of its acquisition outweighs the metabolic cost. Despite numerous examples of ICE transfers and multi-species spread, there are also examples where no transfer or single-species transfers occurred, especially for the GA2 T6SS. Therefore, fitness conferred by T6SS ICE acquisition is likely context-dependent and may be contingent upon host physiology and emergent properties of the community and its microbial composition.

### GA1 and GA2 ICE integrate at different genomic sites

**Fig 5:**
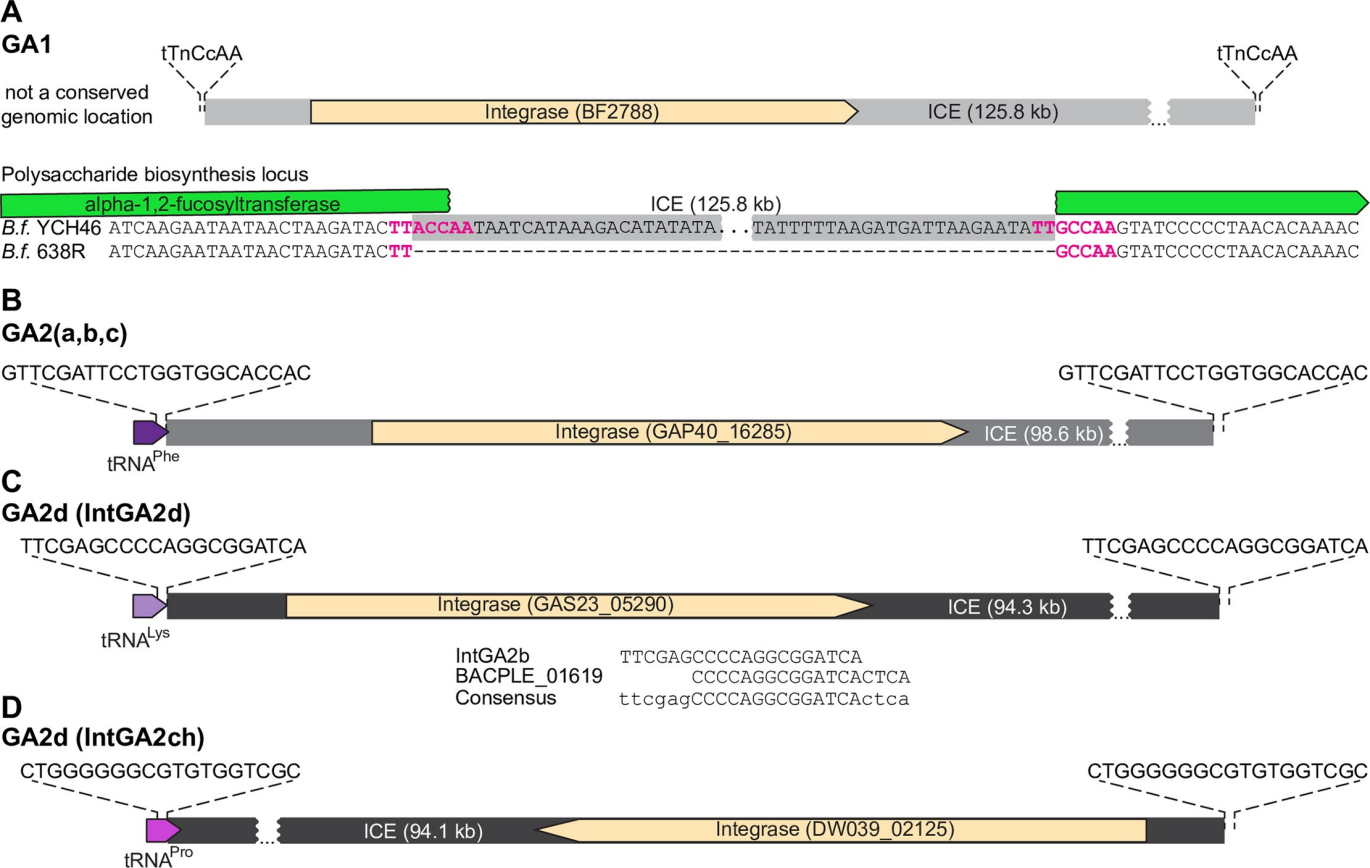
Sequence specificity of the GA1 and GA2 integrases in *Bacteroides* and *Parabacteroides* genomes. **A.** GA1 recombinase and integration site of the *B*. *fragilis* YCH46 ICE (ICE spans BF2788 to BF2921). The insertion of the GA1 ICE in the *B*. *fragilis* YCH46 genome relative to the 638R genome is shown. **B.** The recombinase and integration site of the GA2(a,b,c) showing the GA2b region from *B*. *uniformis* BIOML-A2 as an example (GAP40_16280 to GAP40_16650 and GAP40_07160 to GAP40_07320). This ICE integrates into the tRNA^Phe^ gene. **C, D**. The recombinases and integration sites of two different GA2d ICE. Panel C shows the GA2d region from *B*. *uniformis* BIOML-A77 (GAS23_04785 to GAS23_05290) with the IntGA2d integrase. Shown below are the proposed recognition sequences deduced from BIOML-A77 and BIOML-A84 comparison, the sequence reported for *B*. *plebeius* DSM 17135 (a PUL ICE), and the proposed consensus sequence recognized by IntGA2d. This integrase recognizes the sequence shown in tRNA^Lys^ genes. Panel D shows the GA2d ICE from *B*. *uniformis* AF39-16AC (DW039_02125 to DW039_02640) which harbors the IntGA2ch recombinase which integrates at a tRNA^Pro^ gene sequence.

The sites at which the GA1 and GA2 ICE insert into the recipient genome were not previously analyzed. ICE transfer is often a precisely regulated event [19], with integration into the recipient genome mediated by an integrase encoded within the ICE. Here, we mapped the termini of GA1 and GA2 ICE and determined their chromosomal integration sites and the sequence specificity of the integrases. For GA1 ICE, integration sites varied widely across genomes. For example, in *B*. *fragilis* YCH46, the GA1 ICE insertion disrupted a fucosyltransferase gene (BF2787) in a polysaccharide biosynthesis locus (Fig 5), while in *B*. *cellulosilyticus* CL06T03C01, the GA1 ICE integrated downstream of an *p-*aminobenzoyl-glutamate transporter gene cluster. Analysis of GA1 ICE flanks indicated that there is little sequence conservation between species at the regions directly upstream and downstream of the ICE. The first gene of the GA1 ICE encodes an integrase of the CTnBST tyrosine recombinase family, which are sequence-selective rather than sequence-specific [20]. Wang *et al*. [21] showed that this integrase mediates recombination at sites with the consensus pattern tTnCcAA, where n is any residue, and lower-case letters indicate a single allowed mismatch at one of these two sites. We determined that the GA1 insertion sites in all the genomes we analyzed are flanked by this 7-bp pattern (Fig 5), explaining our observation that this ICE inserts into diverse locations throughout Bacteroidales genomes. ICE insertion leads to a direct duplication of the 7-bp sequence such that it is found at the upstream and downstream junctions of the ICE. By aligning the flanking genomic regions in *B*. *fragilis* YCH46 (with a GA1 ICE insertion) and *B*. *fragilis* 638R (no insertion), we verified that the duplication only spans this 7-bp sequence (Fig 5). This low sequence selectivity may partially explain why GA1 ICEs are widely distributed across *Bacteroides* and *Parabacteroides* species.

In contrast, GA2 ICE insertions are sequence-specific. For subgroups a, b and c (by far the most abundant), all integrations occurred, with roughly equal likelihood, at either of the two tRNA^Phe^ genes. These ICE are flanked on both sides by the 22-bp sequence GTTCGATTCCTGGTGGCACCAC (Fig 5). A comparison of the isogenic strains *B*. *uniformis* BIOML-A2 (GA2 insertion) and BIOML-A3 (no insertion) confirmed duplication of this recognition pattern upon ICE insertion, as it is present only once in the isogenic strain lacking the GA1 ICE. A multiple sequence alignment of the two tRNA^Phe^ regions from species that often carry GA2a/b/c ICE insertions (*B*. *stercoris*, *B*. *uniformis*, and *B*. *vulgatus*) indicates very little conservation in the 15-bp region downstream of the 22-bp recognition pattern. This downstream region is often AT-rich, but is also AT-rich for at least one of the tRNA^Phe^ loci from *B*. *thetaiotaomicron* and other species that rarely acquire GA2a/b/c, indicating that this high AT region may not be a factor in species prevalence. Moreover, GA2a/b/c insertions do not strictly require an unoccupied tRNA^Phe^ site, as we identified one instance (*B*. *xylanisolvens* CL11T00C41) where a GA2b ICE integrated in tandem downstream of a distinct ICE that integrates at the same site. This GA2b ICE integration follows the 22-bp direct repeat generated by the other ICE’s integration (integrases INE93_03024 and INE93_03153, respectively). The tyrosine recombinase located at the beginning of the GA2a/b/c ICE (IntGA2abc, GAP40_16285) is 81.55% identical at the protein level to the integrase of an α-mannan utilization ICE from *Bacteroides thetaiotaomicron* VPI-5482 [22]. This integrase recognizes the same 22-bp sequence as IntGA2abc, and generates the same direct repeats upon integration. Therefore, the presence or absence of the integrase recognition sequence by itself does not explain the species distribution and abundance of GA2, in particular its relative absence from *B*. *thetaiotaomicron* and *B*. *ovatus*. The 22-bp sequence is conserved in *Porphyromonas* species, and while less conserved in *Prevotella* and *Alistipes* species, based on target sequence alone, a significant number of strains that do not contain GA2a/b/c ICE contain the sequence for integration.

The GA2d ICEs do not have a single conserved integrase and therefore do not integrate at a single site. The genomes of seven strains in our collection (S1 Table) contain a GA2d ICE. In one isolate, *Bacteroides eggerthii* AF18-1 (from the CGR collection), the GA2d T6SS region is contained on an ICE similar to GA2a/b/c ICE, with the same insertion site described above. For the other strains, the GA2d ICE is unrelated to the GA2a/b/c ICE and has one of two different integrases that dictate their site of integration. The ICE of five strains, *B*. *vulgatus* BIOML-A119, *B*. *uniformis* BIOML-A85, *B*. *dorei* BIOML-A25, *B*. *plebeius* AM31-10, and *B*. *vulgatus* VPI-4496.2, inserted into the tRNA^Lys^ locus. By comparing the isogenic strains *B*. *uniformis* BIOML-A84 (no GA2d ICE insertion) and BIOML-A85 (GA2d insertion) isolated from the same person, we determined that the 20-bp recognition pattern for the integrase of this ICE (IntGA2d, GAS23_05290 in *B*. *uniformis* BIOML-A77) is TTCGAGCCCCAGGCGGATCA, which duplicates upon ICE insertion (Fig 5). The ICE also inserted at this site in the other four species, and the sequence repeats on each side of the ICE, with only one mismatch in one flank of the insertion in *B*. *vulgatus* VPI-4496.2 (the underlined A in the pattern was substituted by a G). Interestingly, a closely related ICE harboring a porphyran utilization locus (98.57% sequence identity over 48.5% of the core ICE excluding cargo) was characterized in *B*. *plebeius* DSM 17135 [22]. The phage-like recombinase from this ICE is 100% identical to IntGA2d. These authors concluded, based on PCR and sequencing of the products from rare ICE excision events, that the 18-bp recognition sequence for the *B*. *plebeius* integrase (BACPLE_01619) is CCCCAGGCGGATCACTCA. This pattern does not exactly match the direct repeat we identified for IntGA2d (Fig 5). Some *Bacteroides* integrases are known to tolerate a small number of mismatches in the overlap sequence, which may account for this discrepancy [23]. Combining these data, it is predicted that this integrase recognizes/duplicates a 24-bp sequence ttcgagCCCCAGGCGGATCActca with some tolerance for mismatches (lower case) outside the core region (capitalized).

Intriguingly, the GA2d ICE from one isolate, *B*. *uniformis* AF39-16AC, shares lower sequence similarity with the rest of GA2d ICE regions (84.3% average identity) and does not have IntGA2d at the beginning of the ICE (Fig 5). Instead, a 4 kb insertion located at the 3’ end of the ICE harbors a different phage-like recombinase (IntGA2ch), which bears only 24.1% protein sequence identity to IntGA2d and has no characterized orthologs. A similar GA2d ICE with IntGA2ch is present in the chicken isolate *Bacteroides* sp. An19. The 4 kb insertion carrying IntGA2ch is also present in a closely related ICE (96% identical) harboring a different cargo (the *B*. *dorei* HS1_L_1_B010 ICE up to the EL88_18285 integrase). In all three cases, the ICE insertion occurred at a tRNA^Pro^ locus and is flanked by the 19-bp sequence CTGGGGGGCGTGTGGTCGC.

In sum, these observations highlight that recombination events between related ICEs in the Bacteroidales can lead to changes in genomic target location due to integrase swaps, as well as changes in gene cargo of the ICE. In all examples identified, GA2 ICE integrate in tRNA genes, integrating at tRNA^Phe^, tRNA^Lys^ or tRNA^Pro^ genes.

Based on these insertion sites, it is not known why GA2 T6SS loci are scarce in species such as *B*. *thetaiotaomicron*, *B*. *ovatus*, *B*. *fragilis*, and *B*. *intestinalis*, while present in more than 30% of the strains of other species such as *B*. *uniformis*, *B*. *vulgatus* and *B*. *eggerthii*. Whether this bias occurs at the transfer, integration, or maintenance stage is currently unknown. It is possible that these large ICE with their T6SS loci may impose a fitness cost in some species that would outweigh any fitness advantage, precluding their selection. Alternatively, there may be species-level differences in the ability to properly regulate or selectively silence elements on the ICE or T6SS that may lead to decreased fitness. Successful integration and proper regulation of the complex T6SS machinery into different underlying cell physiologies and environmental niches is expected to be highly variable. Our data suggest that GA1, which are present in numerous species with no obvious bias, may present fewer barriers to their acquisition and maintenance.

### Exclusion of GA2 loci with GA1 and GA3 T6SS loci

**Fig 6:**
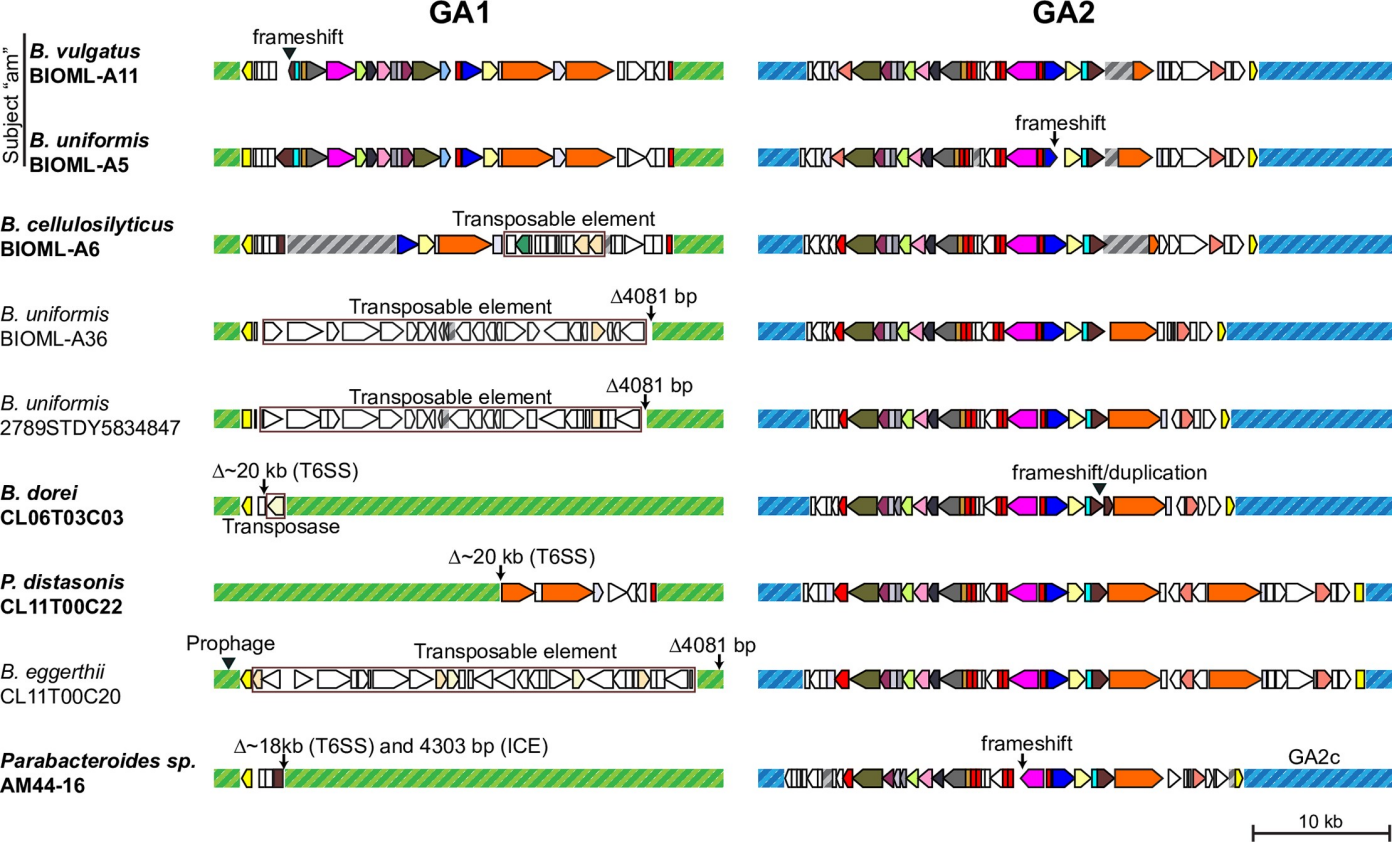
Schematic of genomes that have a GA1 and a GA2, where one of the regions is disrupted. Corresponding genomic coordinates for each region shown in S6 Table are included. Regions of the GA1 ICE outside of the T6SS locus are shown as hatched green boxes and those outside the GA2 T6SS locus are indicated by hatched blue boxes. All GA2 regions shown are of the GA2b subtype except where indicated. Grey hatched boxes indicate gaps in contigs or contigs that were joined manually. Color legend for the T6SS genes as specified in Fig 1. Strains with bolded names have disrupted T6SS regions, whereas non-bolded correspond to the same ICE carrying a different cargo. Insertions are indicated by filled triangles, deletions by arrows. Boxed regions indicate the bounds of a transposable element insertion or recombination event that replaced the cargo.

Based on the previous observations of the nine originally identified genomes with GA2 [3] and our analysis of the distribution of the T6SS genetic architectures and their spread within ecosystems, we suspected that the presence of a GA2 T6SS or ICE may preclude the acquisition of a GA1 ICE and *vice-versa*. In our Bacteroidales isolates genome collection (S1 Table), only eight strains were identified as having both a GA1 and GA2 T6SS locus (excluding from this count one of the two nearly-clonal strains *B*. *vulgatus* AF15-6A and AF16-11). In addition, *B*. *fragilis* strains frequently harbored both a GA3 and GA1 T6SS locus (23 strains), but rarely both a GA3 and GA2 T6SS loci (1 strain). Interestingly, two of the eight strains with both a GA1 and GA2 ICE have disruptions in the T6SS loci. In *B*. *vulgatus* BIOML-A11 (and all its other 79 isogenic co-isolates) the *tssC* gene from GA1 is frameshifted by 2 bp at codon 148 of 460 (Fig 6). In strain *B*. *uniformis* BIOML-A5, isolated from the same subject (“am”), the GA1 is intact but the *vgrG* gene has a 5 bp frameshift at codon 346 of 603 (Fig 6).

ICE entry exclusion is a relatively common phenomenon in Gram-positive bacteria and proteobacteria, where a gene encoded within the ICE acts to prevent the acquisition by the cell of the same or similar ICE [24]. However, this is an unlikely explanation for GA1/GA2 exclusion as there is little sequence conservation between them. To determine whether the main determinant of GA2 exclusion is the ICE or the cargo, we conducted a similarity search for GA1 and GA2a-d ICEs (rather than the T6SS locus) in all Bacteroidales isolate genomes listed in S1 Table and in the community isolate datasets (S3 Table). We identified four additional instances of GA1 and GA2 ICEs present in the same strain where one of the T6SS was frameshifted, truncated or replaced by a transposable element (Fig 6 and S6 Table). These observations suggest a functional and active T6SS may directly prevent acquisition of a second T6SS such that it only happens if the first T6SS is already disrupted. A similar phenomenon has been observed in *Acinetobacter baumannii*, where multi-drug resistance plasmids rely on silencing the T6SS in the host cell to allow for their conjugation [25]. Alternatively, there may be a fitness disadvantage associated with having an intact GA2 T6SS together with a GA1 or a GA3, such that when they co-occur there could be a selective pressure for disruption of one of the loci. However, we were unable to identify any salient features in the T6SS regions of the six strains with both intact GA1 and intact GA2 T6SS loci except that five are from the CGR dataset from Chinese individuals. Notably, for these strains, there was no protein sequence overlap in toxin/immunity genes between the two GA regions nor did they contain acquired interbacterial defense islands carrying the cognate immunity genes to the toxins in their GA1/GA2 [9]. It is possible that one or both of the T6SS may be silenced, in particular in the case of strain AF15-6A (and closely related AF16-11) which carries a GA1, a GA2b, and a GA2c.

Additionally, we found three instances of a GA2 ICE in a strain carrying an ICE closely related to that of GA1 (99.3% identity and 95.2% coverage) but with a different cargo instead of the T6SS locus (Fig 6 and S6 Table). A comparison of the ICE between strains with only one GA1 or GA2 ICE versus the 15 strains where the two co-occur (including the three with different cargo) revealed nothing remarkable about the GA2 locus in strains with GA1. In contrast, this comparison allowed us to identify a gene of interest in the GA1 ICE that may be involved in exclusion. This gene (BF2858 in *B*. *fragilis* YCH46) is absent or disrupted in 11 of the 15 strains (73.3%) due to independent instances of transposase insertions. In contrast, the gene is disrupted in 31.2% of the 125 non-redundant strains with only a GA1 ICE (S1 and S3 Tables) (p = 0.0049 on Fisher’s exact test, comparing disrupted vs. non-disrupted and GA1 alone vs. GA1 and GA2). The gene, which we named *mhgA* for (methylase-helicase GA1), encodes for a 1659-residue protein containing both an *N*^6^-adenine methylase domain and an SNF2 helicase domain. This architecture is reminiscent of type I/III restriction modification systems, although for MhgA the helicase domain does not have sequence signatures predictive of endonuclease activity [26]. Recently, another type of phage-defense system (DISARM) was described which also carries an adenine DNA methylase gene and a SNF2 helicase gene. These proteins, together with another helicase and a DUF1998 domain protein, provide protection from lytic and lysogenic phage infection in *Bacillus subtilis* through an unknown mechanism [27]. The association of the defect in the *mhgA* gene with strains that have both a GA1 and GA2 ICE is intriguing, but it is unknown how such a product would participate in the exclusion of an element acquired in single-stranded form. Nevertheless, the observed GA2 ICE exclusion may therefore be the result of two distinct factors: ICE exclusion of GA2 by an unknown property of MhgA and T6SS exclusion. The latter could be caused by the difficulty of a cell to simultaneously maintain two complex machines that may cross-talk and are each expensive to replicate and fire. Such a combination may therefore only rarely reach fixation in a population.

### Metagenomic analyses of T6SS

**Fig 7:**
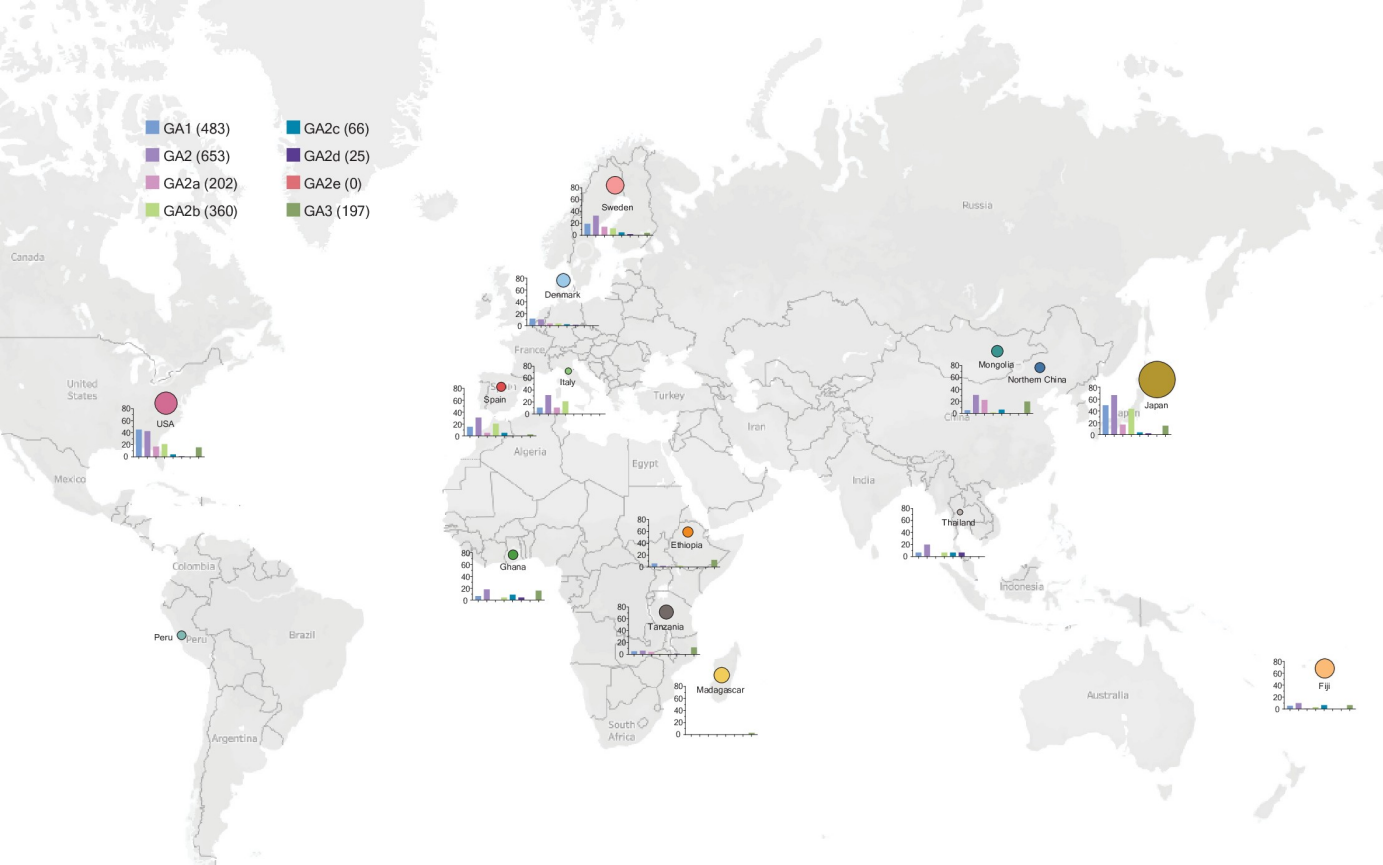
Distribution of the T6SS GA loci and subtypes in gut metagenomes from different human populations. The percentage of metagenomes with each GA1 and GA2 subtype of the total metagenomes for each population is shown by bar graph. The size of each population sampled is designated by the size of the circle. No GA loci were detected in the Peru metagenomic dataset. The exact numbers for each metagenomic dataset are reported in S7 Table as well as the health status of each individual in the various cohorts and the abundance of *Bacteroides spp*. and *Prevotella spp* in the metagenomic sample. Map data copyrighted OpenStreetMap contributors and available from https://www.openstreetmap.org.

Analyses of genome sequences are informative in many regards, but do not reveal the prevalence of these T6SS loci in the metagenomes of human populations. Among Bacteroidales species, human gut microbiomes tend to be dominated largely by *Prevotella* or by *Bacteroides/Parabacteroides* [28, 29]. We analyzed 15 different human gut metagenomic datasets including metagenomes from 1767 individuals to identify the global distribution of the three different T6SS GAs and the five different GA2 subtypes. Additionally, we analyzed each of these metagenomic datasets with MetaPhlAn 2.0 [30] to determine the proportionality of species from the *Bacteroides* and *Prevotella* genera in each individual (S7 Table). In these composite metagenomes, GA2 T6SS loci are the most abundant of the three T6SS GA, where their abundance in *Bacteroides*/*Parabacteroides* dominated communities such as the Japanese and US datasets is 67% and 43% of metagenomes, respectively. GA1 T6SSs are also very prevalent in *Bacteroides/Parabacteroides* dominated communities, present in 50% of Japanese metagenomes and 45% of US gut metagenomes. Other populations such as Mongolians and Fijians have a greater number of metagenomes with GA2 T6SS compared to GA1 T6SS loci (S7 Table and Fig 7). Among the different subtypes of GA2, there are associations with different populations. For example, 74% of the GA2 T6SS in the Mongolian dataset are subtype GA2a, with only two gut metagenomes containing a GA2b locus. In contrast, the GA2b subtype dominates in the population comprising the Japanese dataset (65% of GA2) and GA2c dominate in the Fiji dataset (65% of GA2 loci). The GA2d subtype are only present in 25 metagenomes with a global rather than clustered distribution. No GA2e were detected in any of the 1767 human gut metagenomes queried, further supporting that this GA2 subtype is largely present in Bacteroidales strains of animals. The datasets of individuals from Peru and Madagascar, whose gut microbiota are dominated by *Prevotella* over *Bacteroides* [31] (S7 Table) have 0/36 or 3/112 individuals with strains harboring a Bacteroidales T6SS locus, and in all cases they are GA3 T6SS loci.

### Other MGEs that spread among co-resident Bacteroidales species

To identify other mobile genetic elements (MGE) in gut Bacteroidales that undergo multi-species spread within a person’s microbiota, we scanned all four community isolate datasets (CL, BIOML, UK and CGR collections) for MGEs (4 kb or larger) that are 99.99% identical in at least three different species within a community. We identified multi-species spread of at least one MGE in 40 out of 91 (43.9%) human gut communities. We identified 112 occurrences of MGE multi-species spread, which clustered into 74 MGE groups based on a threshold requiring 80% identity with at least 80% of the element shared within a group (S8 Table). Many of these MGEs are present in communities where they did not spread to multiple species, and therefore were not counted. The majority of the MGEs only spread to multiple species in one community(60), while eight spread in two different communities.

Only six MGEs, including GA1 and GA2b/c ICEs, were found to spread through multiple species in three or more communities with a fully conserved architecture (S8 and S9 Tables). From these conserved and commonly spreading MGEs, one ICE, CTn341 [32] carrying a tetracycline resistance gene, is ubiquitous, present in 94% of communities. Since this MGE is so ubiquitous and conserved across isolates from different subjects, we cannot directly conclude that CTn341 was subject to recent intra-ecosystem transfer. Additionally, a 98 kb conjugative megaplasmid, which we named pMMCAT, spreads to many species in five communities and carries an extracellular or capsular polysaccharide biosynthesis gene cluster and a locus with fimbriae genes. These data highlight the abundant and rapid evolution of human gut Bacteroidales species via mobile genetic elements encoding numerous still unknown functions, many likely related to microbe-microbe and microbe-host interactions. Moreover, these results highlight that GA1 and GA2 ICEs show intra ecosystem spread to multiple species at higher frequencies than any other MGE. Rearrangements in these MGE were common, such that different MGEs are partially overlapping, indicating occasional loss, gain or swapping of genes. Other studies of inter-species horizontal gene transfer showed a similar trend in that clusters of functional genes (cargo) and their encompassing mobilization vehicles often undergo recombination and other rearrangements, such that MGE architectures are not conserved like the GA1 and GA2 are [33, 34]. These frequently transferring T6SS seem to have a favored conserved architecture, with infrequent rearrangements except for swapping of the variable regions containing toxic effector and immunity genes and insertions of IS elements.

We analyzed the metagenomic dataset for the presence of the MGE with evidence of spread in more than three ecosystems (clusters 2, 4, and 7) (S9 Table). Cluster 2 (CTn341 and related ICEs), was strikingly ubiquitous across all sampled populations, locales, and lifestyles, including individuals with very low *Bacteroides* and *Parabacteroides*. Individuals where this ICE is absent are very rare. pMMCAT, which we only detected among *Bacteroides* and *Parabacteroides*, is ubiquitous (>70% abundance) and highly conserved (98–100% identity) in populations from USA, Japan and Thailand. It is also present at substantial frequencies (22–70%) in all the other studied populations except for Madagascar, hunter-gatherers in Tanzania and Peru. Therefore, we cannot rule out that the observed high frequencies of communities with pMMCAT shared at 99.99% identity in multiple species, could in some cases be due to the strain already carrying pMMCAT prior to colonization of the current subject.

One caveat to note is that our MGE spread analysis was conducted using four datasets from industrialized lifestyles, largely from *Bacteroides*-dominated communities. Analyses of HGT events in *Prevotella*-dominated non-industrialized populations indicated less frequent HGT [18] and may show a different repertoire of MGEs and different frequency of multi-species spread.

Through the genomic analyses of more than 1500 Bacteroidales strains, many co-resident in the same individual, combined with analyses of nearly 1800 gut metagenomic samples of diverse global populations, we have uncovered numerous properties of the T6SS of the gut Bacteroidales. Previously thought to be rarely present in the human gut microbiota, we show that GA2 T6SS loci are the most prevalent of the three GA in the human gut metagenomes analyzed here and that T6SS of GA2 segregate into five subtypes, one of which is largely restricted to animals. GA2 subtypes in many cases demonstrate population clustering with GA2b the most abundant in Japan, but rarely present in the Mongolian population sampled where the GA2a subtype dominates. GA1 ICE acquisition and spread may be further facilitated by the low sequence selectivity necessary for integration. Most importantly, this study reveals the extensive intra-ecosystem transfer of these ICE to co-resident members of the gut microbiota, suggesting a potential community benefit by multi-species acquisition. This type of globally conserved architecture is rare for MGEs that spread to multiple species within an individual. This observation is especially interesting for GA1 and GA2, given that the T6SS variable regions harboring the effectors are frequently recombined, yet the general architecture is preserved.

## Materials and methods

### Creation and curation of a Bacteroidales isolate genome collection

All genomes classified by NCBI in April 2020, as belonging to the order Bacteroidales, excluding genomes that were suppressed or considered anomalous, and excluding genomes flagged as derived from metagenomics studies or surveillance projects, were identified by an Entrez query. This initial set of 2,324 genomes was further curated to reduce clearly redundant genomic sequences (e.g. the same strain sequenced multiple times, including equivalent entries from strain repositories), reduce longitudinal samples comprising the same strain isolated from the same individual multiple times, and to remove duplicate entries from both the GenBank and Refseq repositories, retaining the RefSeq sequence. Finally, genomic sequences that were assembled from metagenomics studies despite not being flagged as such by NCBI and those genomes not identified to at least the genus level were removed. The final set comprises 1,434 Bacteroidales genomes (S1 Table).

### PCR screening for GA2 T6SS

We screened 136 *Bacteroides* and *Parabacteroides* isolates collected from four volunteers in the Comstock lab strain collection previously isolated under a protocol approved by the Partners Human Research Committee IRB and complied with all relevant federal guidelines and institutional policies [14]. Each strain was spotted on a BHIS plate and grown anaerobically. A very small amount of cell material was collected from the colony, resuspended in 50 μl water, boiled for 10 minutes and centrifuged. 1 μl from the supernatant was used for a 20 μl PCR reaction with Phusion polymerase (NEB), using primers oLGB21 (TGGGAGCAAGTTTTCTGAATTTGG) and oLGB22 (TGTTCTCCTGCGCTACATAATCGTATC) for the conserved region, and primers oLGB27 (CKTGAATTGAACATCCATTCCAR, where K = G,T and R = A,G) and oLGB28 (GATCCAGTGGATGCTGGATG) for the variable region (Fig 3A). The annealing temperatures were 54°C for the conserved region and 64.5°C for the variable region, with extension times of 50s and 1m45s, respectively. For the variable regions, PCR bands were purified from an agarose gel and sequenced using Sanger sequencing.

### DNA extraction, sequencing and genome assembly

The genomic sequencing of bacterial strains isolated from human fecal samples who provided formal written consent, was approved by the Partners Human Research Committee IRB and complied with all relevant federal guidelines and institutional policies. Strains (S2 Table) were grown anaerobically in basal medium as described previously [35] to an OD_600_ of ~0.8. DNA was recovered using a CTAB/NaCl DNA extraction protocol followed by sodium acetate/ethanol precipitation. SMRT sequencing was carried out at the University of Maryland’s Institute for Genome Sciences Genomics Resource Center, who performed the initial quality control, library preparation, and sequencing of the genomes using PacBio Sequel v3 SMRTcell technology. The genomes were assembled separately using Falcon/Unzip 1.2.0 [36] and Flye 2.8.2 [37], then reconciled using the Flye ‘subassemblies’ option. In cases where unresolved bubbles where still present (*B*. *uniformis* CL06T03C05, *B*. *dorei* CL06T03C03, *B*. *cellulosilyticus* CL06T03C01, *B*. *xylanisolvens* CL11T00C03, *B*. *eggerthi*i CL11T00C20) as assessed by Bandage [38], the two assemblies were also reconciled with a Canu 1.8 [39] assembly. Remaining ambiguities due to invertible DNA regions were fixed manually, using the inverted repeat regions as bounds and available complete reference genomes as guidance. Reconciled assemblies were polished using GCpp 2.0.0 (Pacific Biosciences). Genomes from *B*. *ovatus* CL03T12C18, *B*. *vulgatus* CL11T00C01 and *B*. *xylanisolvens* CL03T12C04, where multiple contigs were obtained or bubbles remained unresolved, were scaffolded using Ragout 2.3 using complete genomes from the same species as reference [40]. Genes were called using Prodigal 2.6.3 [41] and annotation was performed using a customized version of Prokka 1.14.6 [42]. Small contigs were classified as plasmids based on circularization during assembly and using PlasFlow [43]. The genomes were submitted to GenBank and assigned BioProject accession number PRJNA669351.

### Community collections

In addition to our own longitudinal isolates (see below), we utilized three other publicly available isolate sequence sets in our analysis of intra-community DNA transfer. Genomes from these sets were included if they were identified as belonging to the order Bacteroidales and comprised at least three different species isolated from the same individual. These included: 967 genomes from BioProject accession PRJNA544527 (BIOML collection, [15] representing 23 species of 4 genera collected from 10 individuals); 380 genomes from BioProject accession PRJNA482748 (CGR collection, [16] comprising 30 species of 6 genera collected from 71 individuals); 27 genomes from BioProject accession PRJEB10915 (UK collection, [17] representing 13 species from 4 genera sampled from 5 individuals); and finally the CL collection comprised 42 genomes from 18 species of two genera collected from five volunteers (S3 Table). It included the 23 genomes sequenced in this study (S2 Table, BioProject accession PRJNA669351) plus GenBank accessions GCA_001640865.1, GCA_000307345.1, GCA_000273015.1, GCA_000273035.1, GCA_000273055.1, GCA_000273175.1, GCA_000273235.1, GCA_000273235.1, GCA_000307395.1, GCA_000307375.1, GCA_001535615.1, GCA_001535595.1, GCA_000269545.1, GCA_001535605.1, GCA_000273295.1, GCA_000307435.1 and GCA_000307495.1.

These 1,530 genomes were used to create blast databases for intra-community DNA transfer analysis.

### Distribution of T6SS genetic architectures in genome sequences of Bacteroidales isolates

The T6SS loci found in species of the Bacteroidales order fall into three genetic architectures (GA). All three of these GAs contain structural genes (i.e. the genes named with the *tss* nomenclature) which are consistent as to order and identity within a GA. They also each contain variable regions encoding such things as toxin and immunity proteins, RHS proteins, and evolved TssD proteins. These variable regions would complicate analysis of the distribution of these GAs in both metagenomic datasets and in sequenced isolates. We therefore made DNA-level concatemers by removing the sequence encompassing the variable region(s) from a representative of each GA, and used these as templates for metagenomic read mapping (see above) and as queries for analysis of sequenced isolates.

The curated set of 1,452 Bacteroidales genomes (see above and S1 Table) were processed into nucleotide and protein blast databases using Blast v. 2.10.0+, and the T6SS concatemer templates were used as queries using blastn. The results were parsed and the presence or absence of T6SS GA(s) was determined for each genome (see S1 Table). The majority of the results for all seven queries (GA1, five GA2 subtypes, and GA3) were unambiguously clear (e.g. a sequence span detected in an isolate that was greater than 95% identical covering 95% or more of the query). More ambiguous returns with less coverage or lower DNA identity were inspected manually by retrieving the segment(s) involved, usually including some flanking DNA, from the isolate’s genome sequence, and scaffolding hits spanning multiple contigs from heavily fragmented genome sequences. Further comparative analyses were also performed between all concatemer templates and all subject sequences using Clustal Omega v 1.2.4 [44] under Linux (CentOS v 8).

### Analysis of DNA sequence-level relationships among the GA2 T6SS loci subtypes and the GA2 ICE

Sequence identity differences among the five T6SS GA2 subtypes were initially detected in high-scoring segment pairs returned during BLAST analyses. In order to confirm that these sequence identity disparities were not anomalies limited to a particular region of the loci, further analyses were performed using global alignment approaches. S2 Fig was produced by passing the Clustal Omega alignment of the GA2 subtype concatemers to MView v. 1.63 [44] via the EMBL-EBI portal, using the default settings. Examination of the within- and between-community GA2 ICEs also utilized these tools. Intracommunity multiple sequence alignments revealed several insertion sequences present on some but not all of the ICEs within a community. These IS elements were removed from the ICE sequences, and the remaining sequence was used as blast queries against the entire repertoire of CL06T03 and CL11T00 ICEs.

### Phylogenetic trees

Phylogenetic analyses were based on 16S sequences acquired from public repositories such as RDP and Silva. Representative 16S sequences of Bacteroidia identified as type strains by JGI were analyzed for phylogeny using MEGA X [45]. After alignment with Clustal as implemented by MEGA X, the Maximum Likelihood trees were created under the General Time Reversible model (GTR), using a discrete gamma distribution to model evolutionary rate and a rate variation model that allowed some sites to be evolutionarily invariable. Initial trees were obtained via Maximum Parsimony method. The trees shown are the bootstrap consensus trees inferred from 500 replicates. To build the tree of GA2 T6SS regions, sequences were aligned in Clustal Omega and the tree was computed using RAxML [46] using the GTR model, ML estimate of stationary base frequencies, gamma distribution to model among-site rate heterogeneity and a bootstrapping cutoff of 0.03.

### Analysis of T6SS transfer events within co-resident strains

T6SS regions in the community genome collection were identified as specified previously.

To determine if T6SS regions were identical within isolates from the same community, regions were retrieved with 10 kb flanking regions on both sides and aligned using Clustal Omega. Regions were determined to be subject to a recent transfer event if they were >99.99% identical over the complete span of the T6SS, including the variable regions.

### Identification of other mobile genetic elements that transfer within individual microbiomes

A separate nucleotide blast database was created for each community set. Each genome from the community was compared against the database using blast, with cutoffs of 99.99% identity and minimum alignment length of 4000 bp. Hits were retained if they were present in three or more isolates of different species. Redundancy between hits was reduced using cd-hit-est with a sequence identity cutoff of 0.99 [47]. Since many of the genomes analyzed are heavily fragmented (and to by-pass the abundant transposase insertions in Bacteroidales genomes), non-redundant hits were compared against each other using blast and joined using the Flye ‘subassemblies’ option if there was more than 4000 bp overlap at 99.99% identity. The resulting regions, together with those that didn’t require joining, were once again used to search the community genome database, to verify that each sequence was still present in at least three different species with 99.99% identity. Genomic regions that fulfilled these conditions were considered to be MGEs subject to recent (i.e. during the lifetime of the subject) within-host multi-species spread. MGEs were clustered into similar groups using blast, with an 80% identity cutoff and requiring that the alignment covers 80% of the larger fragment.

### Metagenomic datasets, read mapping, and compositional profiling

Fifteen publicly available metagenomic datasets [31, 33, 48–58] were utilized in our investigations into the prevalence and distribution of various sequences of interest. Briefly, these sets collectively comprised sequencing reads from 1,767 individuals from geographically diverse regions of the world, and encompassed varying ethnic, cultural, age, gender, health, and lifestyle groups. The metagenomics read sets (see S7 Table) were downloaded from the European Nucleotide Archive using Aspera. Tools from the BBMap ver. 38.86 (http://sourceforge.net/projects/bbmap) collection of analysis utilities were used to map reads from these sets to sequences of interest. Though the five GA2x T6SS regions are demonstrably different from one another, they do share a somewhat high level of sequence similarity that might influence short read mapping results. Thus, for these analyses, BBsplit was used, as it maps reads to multiple references simultaneously and, in the case of ambiguity (the read maps to more than one template), will determine the best match and count that read only once. Other mapping analyses were ambiguous matches were not an issue utilized BBmap.

## Supporting information

S1 Fig Concatemers of the various gut Bacteroidales T6SS genetic architectures. The bottom gene map of each pair shows the concatemers that were created after removing all genes that diverge within the same genetic architecture. These concatemers were used to query the various datasets analyzed in this study.
(PDF)

S2 Fig Clustal Omega alignment of the GA2 T6SS region concatemers of each of the five subtypes. (PDF)

S1 Table Prevalence of T6SS regions in a non-redundant collection of sequenced Bacteroidales genomes. (XLSX)

S2 Table Prevalence of T6SS regions in the different Bacteroidales species. (XLSX)

S3 Table Prevalence of GA1 and GA2 T6SS in the CL(**A**), UK(**B**), CGR(**C**) and BIOML(**D**) strain collections.
(XLSX)

S4 Table Genomic coordinates for the GA1(**A**) and GA2 subtype(**B**-**E**) T6SS regions in the CL, BIOML, CGR and UK culture collections.
(XLSX)

S5 Table **A.** Complete GA1 and GA2 fixation in cases where only one strain per species is present in the community (for this table we only included strains with at least 4 related isolates). **B.** Partial GA2 fixation in cases where only one strain per species is present in the community. **C.** GA1 and GA2 spread in cases where more than one unrelated strain per species is present in the community. Isolates were classified into strain groups, each designated by a different letter.
(XLSX)

S6 Table **A.** Strains with two GA ICE integrations including at least one GA2. **B.** Genomic coordinates of GA ICE in strains with two integrations including at least one GA2.
(XLSX)

S7 Table Presence of T6SS loci in metagenomic datasets. (XLSX)

S8 Table Mobile genetic elements that spread to three or more species in Bacteroidales communities. Dataset includes genomic coordinates for one representative from each cluster; and the list of communities where each MGE spread is observed.
(XLSX)

S9 Table Prevalence in metagenomes of highly transferred MGEs listed in S8 Table (excluding GA1 and GA2 ICE). (XLSX)
