# Supplementary material for: Mobile Type VI secretion system loci of the gut Bacteroidales display extensive intra-ecosystem transfer, multi-species spread and geographical clustering
Source: PLoS Genet. 2021 Apr 26;17(4):e1009541. doi: 10.1371/journal.pgen.1009541 (PMC8102008; doi:10.1371/journal.pgen.1009541)
Supplement: S1 Fig — The bottom gene map of each pair shows the concatemers that were created after removing all genes that diverge within the same genetic architecture. These concatemers were used to query the various datasets analyzed in this study. (PDF) [file pgen.1009541.s001.pdf]

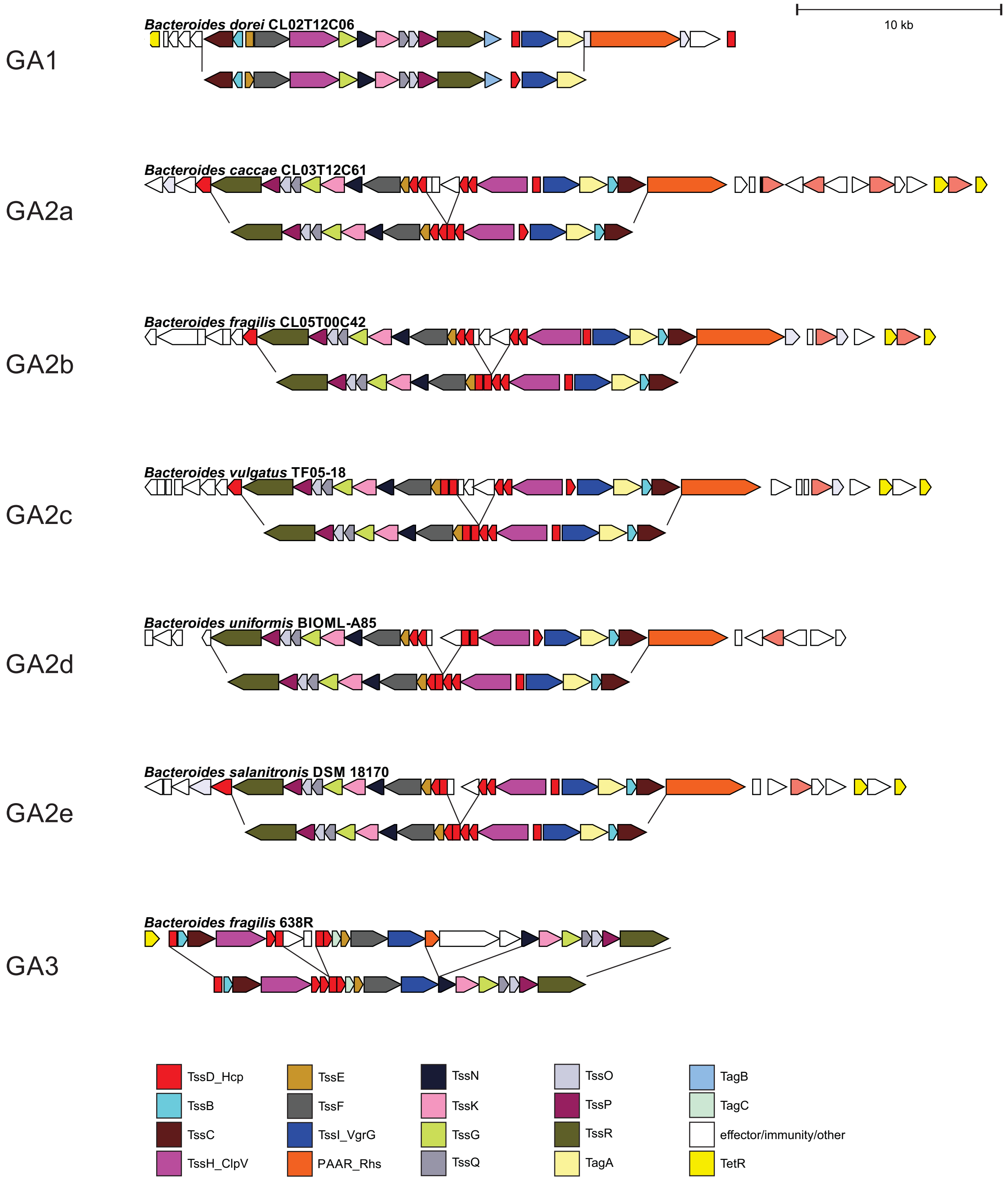

Figure S1. **Concatemers of the various gut Bacteroidales T6SS genetic architectures.** The bottom gene map of each pair shows the concatemers that were created after removing all genes that diverge within the same genetic architecture. These concatemers were used to query the various datasets analyzed in this study.
