# Supplementary material for: Mobile Type VI secretion system loci of the gut Bacteroidales display extensive intra-ecosystem transfer, multi-species spread and geographical clustering
Source: PLoS Genet. 2021 Apr 26;17(4):e1009541. doi: 10.1371/journal.pgen.1009541 (PMC8102008; doi:10.1371/journal.pgen.1009541)
Supplement: S2 Fig — (PDF) [file pgen.1009541.s002.pdf]

Figure S2. MView-colored Clustal Omega alignment of the GA2 T6SS concatemer regions from each of the five subtypes.

```
Reference sequence (1): GA2a.Bcac.CL03T12C61
Identities normalized by aligned length.
Colored by: identity

                        cov    pid   1 .        .         .         .         .         .         .         .         . 80
GA2a.Bcac.CL03T12C61 100.0% 100.0%    ctatggtaacaaatcacgactaatccaataataagcctgcccgttggaccggaacgccttgccggcagcctcatccagca
GA2b.Bfra.CL05T12C13  99.7%  81.0%    ctatggtaataggtcacggctgatccaatagtatgtctgcccgttggactggaacgacttgccggcggcttcgtccagca
GA2c.Bvul.TF05-18     99.8%  82.0%    ctaaggtaataaatcacggctgatccagtagtacgtctgcccgttggattggaatgacttgccggcggcttcgtccagca
GA2d.Buni.BIOML-A83   99.2%  69.8%    ctatggtaacaaatctcggctaatccaataatacgtctgtccattagattgaaatgatttaccggcagcttcgtccaaca
GA2e.Bsal.DSM.18170   99.7%  79.1%    ttagggtaacagctcacggctgatccagtaataggtctctccgttggatttgaacgatttgccggctgcttcgtccaaca
consensus/100%                        sTAsGGTAAsAusTCsCGuCTuATCCAuTAuTAsGsCTssCCuTTuGAsssuAAsGssTTuCCGGCsGCsTCuTCCAuCA
consensus/90%                         sTAsGGTAAsAusTCsCGuCTuATCCAuTAuTAsGsCTssCCuTTuGAsssuAAsGssTTuCCGGCsGCsTCuTCCAuCA
consensus/80%                         CTAsGGTAAsAuuTCACGGCTuATCCAuTAuTAsGTCTGsCCGTTGGAsTGGAAsGAsTTGCCGGCuGCTTCGTCCAuCA
consensus/70%                         CTAsGGTAAsAuuTCACGGCTuATCCAuTAuTAsGTCTGsCCGTTGGAsTGGAAsGAsTTGCCGGCuGCTTCGTCCAuCA

                        cov    pid  81 .        .         .         .         .         .         .         .         .   160
GA2a.Bcac.CL03T12C61 100.0% 100.0%    gttttttcttttccttgaaatactcaatcagcccgtccagttccacgtccgtaatcatctcctttttccgcaagtccttg
GA2b.Bfra.CL05T12C13  99.7%  81.0%    gttttttcttttccttgaaatattcaatcagcatgtccagctccgcatccgtaatcattttctttttccgcaagtccttg
GA2c.Bvul.TF05-18     99.8%  82.0%    gttttttcttttccttgaaatattcaatcagcatgtccagttccacatccgtaatcattttctttttccgcaagtccttg
GA2d.Buni.BIOML-A83   99.2%  69.8%    gtttcttcttttccttaaaatattcaatcagtttgtccaattctacatcggtaatcatctcctttttcttcaagtcctta
GA2e.Bsal.DSM.18170   99.7%  79.1%    ttttcttcttttccttgaagtattcaatcagcatgtccagttccacgtccgtaatcatatccttgtctttcaggtctttg
consensus/100%                        sTTTsTTCTTTTCCTTuAAuTAsTCAATCAGsssGTCCAusTCsuCuTCsGTAATCATsTsCTTsTssssCAuGTCsTTu
consensus/90%                         sTTTsTTCTTTTCCTTuAAuTAsTCAATCAGsssGTCCAusTCsuCuTCsGTAATCATsTsCTTsTssssCAuGTCsTTu
consensus/80%                         GTTTsTTCTTTTCCTTGAAATATTCAATCAGCsTGTCCAGTTCCACuTCCGTAATCATsTsCTTTTTCssCAAGTCCTTG
consensus/70%                         GTTTsTTCTTTTCCTTGAAATATTCAATCAGCsTGTCCAGTTCCACuTCCGTAATCATsTsCTTTTTCssCAAGTCCTTG

                        cov    pid 161 .        .         .         .         .         .         .         .         . 240
GA2a.Bcac.CL03T12C61 100.0% 100.0%    acacggtaaacctcgaagaacggataatccacagggcacgatgtgaaccgttgcaacgcttccgacaaaggcatgttcag
GA2b.Bfra.CL05T12C13  99.7%  81.0%    acacggtaaacttcaaagaaaggatagtcggtcgggcaggaggtaaacctttgcaaggcctctgacaggggcattctcag
GA2c.Bvul.TF05-18     99.8%  82.0%    acacggtaaacctcaaagaaaggatagtcagccggacaggaggtaaacctttgcaagacctctgacaggggcattttcag
GA2d.Buni.BIOML-A83   99.2%  69.8%    atccgatatacctcaaaaaacggatagtccaccggacatgaagtaaatcttcgtaaagattctgatataggcattctcaa
GA2e.Bsal.DSM.18170   99.7%  79.1%    acccgatagacctcaaagaacgggtagtccgccgggcaggaggtaaaccgctgcaaggcttccgatatgggcattctcaa
consensus/100%                        AssCGuTAsACsTCuAAuAAsGGuTAuTCsussGGuCAsGAsGTuAAsCsssGsAAsussTCsGAsAsuGGCATssTCAu
consensus/90%                         AssCGuTAsACsTCuAAuAAsGGuTAuTCsussGGuCAsGAsGTuAAsCsssGsAAsussTCsGAsAsuGGCATssTCAu
consensus/80%                         ACsCGuTAuACCTCAAAGAAsGGATAGTCsuCCGGuCAsGAuGTAAACCsTTGCAAuGCsTCsGAsAsuGGCATTsTCAu
consensus/70%                         ACsCGuTAuACCTCAAAGAAsGGATAGTCsuCCGGuCAsGAuGTAAACCsTTGCAAuGCsTCsGAsAsuGGCATTsTCAu

                        cov    pid 241 .        .         .         .         .         .         .         .         . 320
GA2a.Bcac.CL03T12C61 100.0% 100.0%    gaaatcccgtcttcccgtcttgcagtacttgtccctgttgcgttccgaaagcagatgccttaccagttgccttctcacac
GA2b.Bfra.CL05T12C13  99.7%  81.0%    gaaagtctttctccctaccttgcaatatctgtccctgttgcgccctgaaaggaaatgtctgaccagttttcttcttacac
GA2c.Bvul.TF05-18     99.8%  82.0%    gaaagtctttctccccaccctgcaatacctgtccctgttgcgctctgaaaggaaatgtctgaccagcttccttcttacat
GA2d.Buni.BIOML-A83   99.2%  69.8%    ggaagccttccttttaattttacaatacttatctgtatttctctcagaaaggaaatgccgtactagctttcttctgacac
GA2e.Bsal.DSM.18170   99.7%  79.1%    aaaggtcttcctcttcaccttgcaatacttgtccgtgttgcgctcggacaggaaatgccgtaccaactttctccgcacac
consensus/100%                        uuAussCsssCTssssusssTuCAuTAssTuTCssTuTTsCsssCsGAsAGsAuATGsCssACsAusTssCTsCssACAs
consensus/90%                         uuAussCsssCTssssusssTuCAuTAssTuTCssTuTTsCsssCsGAsAGsAuATGsCssACsAusTssCTsCssACAs
consensus/80%                         GAAAGsCTTsCTssssAsCTTGCAATACsTGTCCsTGTTGCGCTCsGAAAGGAAATGsCssACCAGsTTsCTTCTsACAC
consensus/70%                         GAAAGsCTTsCTssssAsCTTGCAATACsTGTCCsTGTTGCGCTCsGAAAGGAAATGsCssACCAGsTTsCTTCTsACAC
```

```
                           cov     pid  321 .         .         .         .         .         .         .         .         . 400
GA2a.Bcac.CL03T12C61   100.0%  100.0%      ggcgcgtcggggcatattccggtatgttcaggctgtccgtcttcacacgtatggtagcctcctctgtatctgtcggcaga
GA2b.Bfra.CL05T12C13    99.7%   81.0%      gtcgggttgaggcatattctggtgcgttcaggctgtccgtctttacacgtacggtagattcttcggcatctgtctgtagg
GA2c.Bvul.TF05-18       99.8%   82.0%      gtcgggttgaggcatattccggcacgttcaggctgtccgtcttgatgcgcacggtcgattcttcggtgtctgtctgtagg
GA2d.Buni.BIOML-A83     99.2%   69.8%      gacgagttggagcatactcaggaacattcagactgtctctctttatactaatagctgtttcatcgttattatctgttata
GA2e.Bsal.DSM.18170     99.7%   79.1%      ggcgggtagaggcatattccggcacgttcaggctgtccgtcttcacgtgtacggtggattcttcggtatctgtttcgaag
consensus/100%                             GsCGsGTsGuuGCATAsTCsGGsusuTTCAGuCTGTCssTCTTsAsusssAsuGssGssTCsTCsssuTssssssssAsu
consensus/90%                              GsCGsGTsGuuGCATAsTCsGGsusuTTCAGuCTGTCssTCTTsAsusssAsuGssGssTCsTCsssuTssssssssAsu
consensus/80%                              GsCGuGTsGuGGCATATTCsGGsACGTTCAGGCTGTCCGTCTTsAsuCGsAsGGTsGsTTCsTCGGTATCTGTssssAuu
consensus/70%                              GsCGuGTsGuGGCATATTCsGGsACGTTCAGGCTGTCCGTCTTsAsuCGsAsGGTsGsTTCsTCGGTATCTGTssssAuu

                           cov     pid  401 .         .         .         .         .         .         .         .         . 480
GA2a.Bcac.CL03T12C61   100.0%  100.0%      taatcgtccggacagttgcagatatcggccttcgcctgctttttctttctgaccgcctcatatttatagtccagaaccag
GA2b.Bfra.CL05T12C13    99.7%   81.0%      tagtcgtccggacagtcgcagatatccaccttcgcttgctttttcttttgaccgcttcgtatttatagtctacgatcag
GA2c.Bvul.TF05-18       99.8%   82.0%      taatcgtccggacagtcgcaaatatccgctttcgcccgctttttcttttgaccgcctcgtatttgtagtccacaatcag
GA2d.Buni.BIOML-A83     99.2%   69.8%      taatcttccggacaatcacagatattaaccttagcctgtttctttttcttacagcttcaactttataatcaatcactaa
GA2e.Bsal.DSM.18170     99.7%   79.1%      tagtcgttcgggcagtcgcagatgtccactttcgcctgtttttcttcttgacggcctcgtatttatagtccacaatcag
consensus/100%                             TAuTCsTsCGGuCAuTsuCAuATuTssuCsTTsGCssGsTTsTTsTTssTsACsGCsTCussTTTuTAuTCsAssAssAu
consensus/90%                              TAuTCsTsCGGuCAuTsuCAuATuTssuCsTTsGCssGsTTsTTsTTssTsACsGCsTCussTTTuTAuTCsAssAssAu
consensus/80%                              TAuTCGTCCGGACAGTCGCAGATATCsuCsTTCGCCTGsTTTTTCTTTsTGACsGCsTCuTATTTATAGTCsAsuAsCAG
consensus/70%                              TAuTCGTCCGGACAGTCGCAGATATCsuCsTTCGCCTGsTTTTTCTTTsTGACsGCsTCuTATTTATAGTCsAsuAsCAG

                           cov     pid  481 .         .         .         .         .         .         .         .         . 560
GA2a.Bcac.CL03T12C61   100.0%  100.0%      cttggcgggcacttccacatactttctcagtcgcttgaactcctcttcattgacgagcaggtaatagtccgttacggacg
GA2b.Bfra.CL05T12C13    99.7%   81.0%      cttggagggtgcttccacatactttctcaaccgttgaactcctcttcgttgacaagcagataatagtccagtgtggagg
GA2c.Bvul.TF05-18       99.8%   82.0%      tttggcgggtgcttccacatatttctcaaccgttgaactcctcttcattggcaagcaggtaatagtccagtgtggagg
GA2d.Buni.BIOML-A83     99.2%   69.8%      cttggctggttcttctatatatttcctgagccgtttaaattcatcttcattcaccagcaaataataatccaatgtagaag
GA2e.Bsal.DSM.18170     99.7%   79.1%      cttggcgggttcttccacatatttctcaggcgcttgaactcgtcttcgttggcaagcagataatactccagtgtccgtg
consensus/100%                             sTTGGssGGssCTTCsAsATAsTTsCTsAusCGsTTuAAsTCsTCTTCuTTsuCsAGCAuuTAATAsTCCusTusssusG
consensus/90%                              sTTGGssGGssCTTCsAsATAsTTsCTsAusCGsTTuAAsTCsTCTTCuTTsuCsAGCAuuTAATAsTCCusTusssusG
consensus/80%                              CTTGGCGGGTsCTTCCACATAsTTTCTCAusCGsTTGAACTCsTCTTCuTTGuCuAGCAGuTAATAuTCCAuTGTuGAsG
consensus/70%                              CTTGGCGGGTsCTTCCACATAsTTTCTCAusCGsTTGAACTCsTCTTCuTTGuCuAGCAGuTAATAuTCCAuTGTuGAsG

                           cov     pid  561.         .         .         .         .         .         .         .         . 640
GA2a.Bcac.CL03T12C61   100.0%  100.0%      aaagcgagtcgggcaatacgacaggctctgccggcagtttccatcccggctcgatacccggcaggacagacaatatcggt
GA2b.Bfra.CL05T12C13    99.7%   81.0%      acagcgagtccggcagtacgattggctgtgcaggcaggctccaggcaggttcggtatccggaagtacggatagcatgggt
GA2c.Bvul.TF05-18       99.8%   82.0%      acagcgagtccggcaatgcgacaggttgcgcaggcaggctccaggtgggttcgatacccggaagtacggacaatatgggt
GA2d.Buni.BIOML-A83     99.2%   69.8%      acagactgtcaggtaatacaacaggagataatggaagcgaccatccaggttctatagaaggacatgctgacattatggtc
GA2e.Bsal.DSM.18170     99.7%   79.1%      atagcgagtctggtaacacgacaggctgtgccggcaggttccaggctggttcgacacccggaagcacagacaatatcggt
consensus/100%                             AsAGsssGTCsGGsAusuCuAssGGssssussGGsAGsssCCAssssGGsTCsusAsssGGssusuCsGAsAssATsGss
consensus/90%                              AsAGsssGTCsGGsAusuCuAssGGssssussGGsAGsssCCAssssGGsTCsusAsssGGssusuCsGAsAssATsGss
consensus/80%                              AsAGCGAGTCsGGsAATACGACAGGsTuTGCsGGCAGssTCCAssCsGGTTCGATAsCCGGAAGsACuGACAuTATsGGT
consensus/70%                              AsAGCGAGTCsGGsAATACGACAGGsTuTGCsGGCAGssTCCAssCsGGTTCGATAsCCGGAAGsACuGACAuTATsGGT
```

```
                            cov    pid   641 .         .         .         .         .         .         .         .       . 720
GA2a.Bcac.CL03T12C61 100.0% 100.0%   tgcacgcctgacctgcggatatgatagtactccaccagcgtcgagtcaagtttataccggtgggttcccacctcctcaaa
GA2b.Bfra.CL05T12C13  99.7%  81.0%   tgtacccccgactggcggatatggtaataagctgtcattgtcgaatctgtcctatatcggtgtgtacccacttcgtcgaa
GA2c.Bvul.TF05-18     99.8%  82.0%   tgcacacccgactgtcggatatggtaataatctgtcattgtcgagtccgtcctatatcggtgtgtacccacttcgtcgaa
GA2d.Buni.BIOML-A83   99.2%  69.8%   tgtttatcagttttctttatatgatgataagctacaaatgttgaatcaagtttataccgatttgtaccgacctccgcaaa
GA2e.Bsal.DSM.18170   99.7%  79.1%   tgcacccctgcctggcggatgtggtagtagtccgtcatagtcgagtccgccttataccgttgggttcccacttcgtcgaa
consensus/100%                       TGsssssCsGssssssssATuTGuTuuTAssCsussAssGTsGAuTCsusssTATAsCGsTssGTsCCsACsTCssCuAA
consensus/90%                        TGsssssCsGssssssssATuTGuTuuTAssCsussAssGTsGAuTCsusssTATAsCGsTssGTsCCsACsTCssCuAA
consensus/80%                        TGsACsCCsGsCTssCGGATATGuTAuTAusCsusCAssGTCGAuTCsusssTATAsCGuTGsGTsCCCACsTCsTCuAA
consensus/70%                        TGsACsCCsGsCTssCGGATATGuTAuTAusCsusCAssGTCGAuTCsusssTATAsCGuTGsGTsCCCACsTCsTCuAA

                            cov    pid   721 .         .         .         .         .         .         .         .      .800
GA2a.Bcac.CL03T12C61 100.0% 100.0%   cgcccttgtcaggctgttgcccaacagcgtattgtcaaacttcacctgcaacagcatggagtccacggcggtagtcagcc
GA2b.Bfra.CL05T12C13  99.7%  81.0%   tgcccttgccaggctgttgctcagcagggtattgtcaaacttcacttgtatcagcatggaatccacggcggtggtcagtc
GA2c.Bvul.TF05-18     99.8%  82.0%   tgcccttgccaggctgttgctcaacagggtattgtcaaacttcacttgcagcagcatggagtccacggcggcggtcagtc
GA2d.Buni.BIOML-A83   99.2%  69.8%   agctctttccaattggttgtacaatatggtattatcaagtttcacttgcaacaacatggaatctacagcctgagtcagac
GA2e.Bsal.DSM.18170   99.7%  79.1%   tgctcttgtcaggctgttgctcagcagggtgttgtcaaacttcacttgcagcagcatggaatccaaggcggaggtcagcc
consensus/100%                       sGCsCTTssCAusssGTTGssCAusAssGTuTTuTCAAusTTCACsTGsAsCAuCATGGAuTCsAsuGCsssuGTCAGsC
consensus/90%                        sGCsCTTssCAusssGTTGssCAusAssGTuTTuTCAAusTTCACsTGsAsCAuCATGGAuTCsAsuGCsssuGTCAGsC
consensus/80%                        sGCsCTTGsCAGGCTGTTGCsCAuCAGGGTATTGTCAAACTTCACTTGCAuCAGCATGGAuTCCACGGCGGsuGTCAGsC
consensus/70%                        sGCsCTTGsCAGGCTGTTGCsCAuCAGGGTATTGTCAAACTTCACTTGCAuCAGCATGGAuTCCACGGCGGsuGTCAGsC

                            cov    pid   801 .         .         .         .         .         .         .         .       . 880
GA2a.Bcac.CL03T12C61 100.0% 100.0%   cctccagctccagtgactcgtttttctgcggaaagacaagccatccctgtgtcatgctccggttcggaaagtccaggcaa
GA2b.Bfra.CL05T12C13  99.7%  81.0%   cttccagttccagcgattcgttcttttgcggaaacaccagccatccctgtgtcatactccggtttggaaagtccagacaa
GA2c.Bvul.TF05-18     99.8%  82.0%   cttccaattccagcgattcgttcttttgcggaaataccagccatccctgcgtcatgctccggttgggaaaatccaggcaa
GA2d.Buni.BIOML-A83   99.2%  69.8%   tttccatttccaaagattcattcttctgtggaaatactagccatccctgagtcatacttctatctggaaagtccagacaa
GA2e.Bsal.DSM.18170   99.7%  79.1%   cttccagttccagcgattcgttcttttgcggaaacaccagccatccctgtgtcatgctccggttggggaagtccaggcaa
consensus/100%                       ssTCCAssTCCAusGAsTCuTTsTTsTGsGGAAAsACsAGCCATCCCTGsGTCATuCTsCsuTssGGuAAuTCCAGuCAA
consensus/90%                        ssTCCAssTCCAusGAsTCuTTsTTsTGsGGAAAsACsAGCCATCCCTGsGTCATuCTsCsuTssGGuAAuTCCAGuCAA
consensus/80%                        CTTCCAuTTCCAGsGATTCGTTCTTsTGCGGAAAsACsAGCCATCCCTGsGTCATuCTCCGGTTsGGAAAGTCCAGuCAA
consensus/70%                        CTTCCAuTTCCAGsGATTCGTTCTTsTGCGGAAAsACsAGCCATCCCTGsGTCATuCTCCGGTTsGGAAAGTCCAGuCAA

                            cov    pid   881 .         .         .         .         .         .         .         .       . 960
GA2a.Bcac.CL03T12C61 100.0% 100.0%   tacgtattgtggttcacctccgcatattcgtttccgttccgcagttgttcgggatacacaatcagttcccgtttcttccg
GA2b.Bfra.CL05T12C13  99.7%  81.0%   taggtgttatggttcacctccgcatattcgttttcatttctgatttgttcgggatatacaatcagttcccgttttttccg
GA2c.Bvul.TF05-18     99.8%  82.0%   tagatattatggttcacctccgcgtattcgctttcattcctgatttgctcgggatatacaatcagctcccgtttcttccg
GA2d.Buni.BIOML-A83   99.2%  69.8%   taagtattatgatttacttccatatattcattgccattccgtatcaaatccggataaaccatcagttctcttttcttctg
GA2e.Bsal.DSM.18170   99.7%  79.1%   taggtattatggcccacttccgcgtattcgttttcatttcggacttgttcgggatataccaccagctcccgtttctttcg
consensus/100%                       TAsuTuTTuTGusssACsTCCusuTATTCusTssCuTTsCssAsssusTCsGGATAsACsAsCAGsTCsCsTTTsTTssG
consensus/90%                        TAsuTuTTuTGusssACsTCCusuTATTCusTssCuTTsCssAsssusTCsGGATAsACsAsCAGsTCsCsTTTsTTssG
consensus/80%                        TAuGTATTATGGTTCACsTCCGCuTATTCGTTTsCATTsCssAsTTGsTCGGGATAsACsATCAGsTCCCGTTTCTTCCG
consensus/70%                        TAuGTATTATGGTTCACsTCCGCuTATTCGTTTsCATTsCssAsTTGsTCGGGATAsACsATCAGsTCCCGTTTCTTCCG
```

	cov	pid	961 1040
GA2a.Bcac.CL03T12C61	100.0%	100.0%	gctgatgcgtggagccgagcagtctatcatgttcccgatttgcagcacgaaattgttgaagttgtccggttctccgccgt
GA2b.Bfra.CL05T12C13	99.7%	81.0%	gctgatgcgtggtgccgagcagtctatcatgtttccgatttgcagcacgaagttgttgaagttgtccggttctcctccgt
GA2c.Bvul.TF05-18	99.8%	82.0%	gctgatgcgtggtgccgagcagtctatcatgttcccgatttgcagcacgaagttgttgaagttgtccggctctcctccgt
GA2d.Buni.BIOML-A83	99.2%	69.8%	gctgatccgtggagcataacaatcaatcatattaccaagttgaagtacaaagttgttaaaattatcaggctcgcccccgt
GA2e.Bsal.DSM.18170	99.7%	79.1%	gctgatgcgtggtgccgagcagtctatcatgttcccgatttgcagcacaaagttgttgaagttgtccggctctcctccgt
consensus/100%			GCTGATsCGTGGsGCssAuCAuTCsATCATuTTsCCuAsTTGsAGsACuAAuTTGTTuAAuTTuTCsGGsTCsCCsCCGT
consensus/90%			GCTGATsCGTGGsGCssAuCAuTCsATCATuTTsCCuAsTTGsAGsACuAAuTTGTTuAAuTTuTCsGGsTCsCCsCCGT
consensus/80%			GCTGATGCGTGGsGCCGAGCAGTCTATCATGTTsCCGATTTGCAGCACuAAGTTGTTGAAGTTGTCCGGsTCTCCsCCGT
consensus/70%			GCTGATGCGTGGsGCCGAGCAGTCTATCATGTTsCCGATTTGCAGCACuAAGTTGTTGAAGTTGTCCGGsTCTCCsCCGT

	cov	pid	1041 1120
GA2a.Bcac.CL03T12C61	100.0%	100.0%	aaagctggaaccccagcaaccggcagttattctccaccagccggtccaccagagtggaatccgcccattcgctgttgaac
GA2b.Bfra.CL05T12C13	99.7%	81.0%	agagctggaagccgatcatgcggcagttgttttttaccagcttgttcaccaaggtggagtctgcccactcgctgttgaac
GA2c.Bvul.TF05-18	99.8%	82.0%	agagctggaaaccaagcagacggcagttgttctttaccaatttgttcaccaaggtggagtccgcccactcgctgttgaat
GA2d.Buni.BIOML-A83	99.2%	69.8%	acagttggaatcccaacaaacggcaattgttttttaccatctgattcaccaaggcagagtctgcgaactcagtattatag
GA2e.Bsal.DSM.18170	99.7%	79.1%	aaagctggaaacccagcaggcggcagttgttcttcaccagtttgttcaccagagtagagtccgcccattcgctgttgaat
consensus/100%			AsAGsTGGAAsCCsAsCAssCGGCAuTTuTTsTssACCAssssuTsCACCAuuGsuGAuTCsGCssAsTCusTuTTusAs
consensus/90%			AsAGsTGGAAsCCsAsCAssCGGCAuTTuTTsTssACCAssssuTsCACCAuuGsuGAuTCsGCssAsTCusTuTTusAs
consensus/80%			AuAGCTGGAAsCCsAuCAuuCGGCAGTTGTTsTTsACCAusTsGTTCACCAuuGTuGAGTCsGCCCAsTCGCTGTTGAAs
consensus/70%			AuAGCTGGAAsCCsAuCAuuCGGCAGTTGTTsTTsACCAusTsGTTCACCAuuGTuGAGTCsGCCCAsTCGCTGTTGAAs

	cov	pid	1121 1200
GA2a.Bcac.CL03T12C61	100.0%	100.0%	cccttgtcacctaccaccaccaggacattgctctcgtcccgacatttgttgaacagttccacccctgtgcggacacccga
GA2b.Bfra.CL05T12C13	99.7%	81.0%	cctttatcgccgatgacaaccaggatattggtttcatccggacatttgttgaattgctccactccgatccgaaggccgga
GA2c.Bvul.TF05-18	99.8%	82.0%	cctttgtctccgataacaaccagaatattggtctcatccggacatttgttgaattgttccaccccgatttgaaggccgga
GA2d.Buni.BIOML-A83	99.2%	69.8%	cctttatcaccaattacgaccaatacattcgtttcatctttatgatgattgaacatatctacacctattcttactccaga
GA2e.Bsal.DSM.18170	99.7%	79.1%	cctttgtcgccgatgacgaccaggatgtttgtctcatctgggcatttgttgaactgttccaccccgatttgaacacctga
consensus/100%			CCsTTuTCsCCsAssACsACCAusAsuTTssTsTCuTCsssususTsuTTGAAssssTCsACsCCsuTssssAssCCsGA
consensus/90%			CCsTTuTCsCCsAssACsACCAusAsuTTssTsTCuTCsssususTsuTTGAAssssTCsACsCCsuTssssAssCCsGA
consensus/80%			CCTTTuTCuCCuATsACuACCAGuAsATTsGTsTCATCssGACATTTGTTGAAssGsTCCACsCCsATssGuAsuCCsGA
consensus/70%			CCTTTuTCuCCuATsACuACCAGuAsATTsGTsTCATCssGACATTTGTTGAAssGsTCCACsCCsATssGuAsuCCsGA

	cov	pid	1201 1280
GA2a.Bcac.CL03T12C61	100.0%	100.0%	ccagctgccgaatcttccgtacacaggtttcagttttttcgcgttccgtgccttgtccgacagaaagtccagaaattcca
GA2b.Bfra.CL05T12C13	99.7%	81.0%	ccagctgccgaatcgtccgtatgtaggtttcagttgctcggcattacgtgctttggcggaaaggaaatcaagcagctcca
GA2c.Bvul.TF05-18	99.8%	82.0%	ccagctgccgaaccgcccgtatgtgggtttcagttgcccggcattgcgtgccttggcggaaaggaagtcaagcaattcca
GA2d.Buni.BIOML-A83	99.2%	69.8%	ccagcttccaaatctgccatatacaggcttcaacttttccgaatttttagctttggcagagaggaaatcaagcatctcca
GA2e.Bsal.DSM.18170	99.7%	79.1%	ccagctgccgaaccgtccgtatgtaggttttagttgcttgacgttacgtgccttggcggaaaggaagtcaagcagctcca
consensus/100%			CCAGCTsCCuAAsCssCCuTAsusuGGsTTsAusTsssssusuTTssssGCsTTGsCsGAsAGuAAuTCsAGsAssTCCA
consensus/90%			CCAGCTsCCuAAsCssCCuTAsusuGGsTTsAusTsssssusuTTssssGCsTTGsCsGAsAGuAAuTCsAGsAssTCCA
consensus/80%			CCAGCTGCCGAAsCssCCGTATusAGGTTTCAGTTssTssGCuTTsCGTGCsTTGGCuGAuAGGAAuTCAAGCAusTCCA
consensus/70%			CCAGCTGCCGAAsCssCCGTATusAGGTTTCAGTTssTssGCuTTsCGTGCsTTGGCuGAuAGGAAuTCAAGCAusTCCA

```
                    cov    pid 1281 .         .         .         .         .         .         .         . 1360
GA2a.Bcac.CL03T12C61 100.0% 100.0%  tatagtcgggagtcagtttgcagatgggatcttcccggctgtccggctcgttgaaagtgagcacggcgccgaacttgaag
GA2b.Bfra.CL05T12C13  99.7%  81.0%  tgtagtcgggagtcagtttgcagatggggtctttccggttgtccggttcgttgaaggtaagcacggcaccgaacctgaaa
GA2c.Bvul.TF05-18     99.8%  82.0%  tgtagtcgggagtcagcttgcagatagggtcctcccggttgtccggttcgttgaaggtaagcacggcgccgaacttgaaa
GA2d.Buni.BIOML-A83   99.2%  69.8%  tataatcaggggtcaatttacagataggatcgttcaaactaccttcttcattaaaagtcagtaccgctccaaacctgaat
GA2e.Bsal.DSM.18170   99.7%  79.1%  tgtagtcgggagtcagtttgcagatggggtctgtcgggctgtccggttcgttgaaggtcagcacggcgccgaacctgaaa
consensus/100%                      TuTAuTCuGGuGTCAusTTuCAGATuGGuTCsssCsuusTusCssssTCuTTuAAuGTsAGsACsGCsCCuAACsTGAAs
consensus/90%                       TuTAuTCuGGuGTCAusTTuCAGATuGGuTCsssCsuusTusCssssTCuTTuAAuGTsAGsACsGCsCCuAACsTGAAs
consensus/80%                       TuTAGTCGGGAGTCAGTTTGCAGATuGGuTCsTsCsGGsTGTCCGGTTCGTTGAAuGTsAGCACGGCuCCGAACsTGAAu
consensus/70%                       TuTAGTCGGGAGTCAGTTTGCAGATuGGuTCsTsCsGGsTGTCCGGTTCGTTGAAuGTsAGCACGGCuCCGAACsTGAAu

                    cov    pid 1361 .         .         .         .         .         .         .         . 1440
GA2a.Bcac.CL03T12C61 100.0% 100.0%  gagaaactctcatcgttcgccagctgggactgcaacccttggatggcattcaccacggcaggaaactgttcgatggcctt
GA2b.Bfra.CL05T12C13  99.7%  81.0%  gaaaagctgtcgtcattcaccagttgcgattgcagtccctggatggcattcaccacggctggaaaccgttggatggtgtt
GA2c.Bvul.TF05-18     99.8%  82.0%  gaaaagctctcgtcatttgccagttgcgactgtaatccctggatggcattcaccacggcaggaaaccgctggatggtgct
GA2d.Buni.BIOML-A83   99.2%  69.8%  gtaaaactttcatcgtttaccagaactgactgcaggctttgaactgcatttaccaaaaccgggaaacgttcaatggtaat
GA2e.Bsal.DSM.18170   99.7%  79.1%  gaaaagctctcgtcatttaccagttgcgactgcaatccctgaatggtgttcactacggctggaaactgctggatggtgtt
consensus/100%                      GsuAAuCTsTCuTCuTTsuCCAGssssGAsTGsAusCssTGuAssGsuTTsACsAsuuCsGGuAAssGsTsuATGGsssT
consensus/90%                       GsuAAuCTsTCuTCuTTsuCCAGssssGAsTGsAusCssTGuAssGsuTTsACsAsuuCsGGuAAssGsTsuATGGsssT
consensus/80%                       GAAAAuCTsTCuTCuTTsuCCAGsTGsGACTGCAusCCsTGuATGGCATTCACCACGGCsGGAAACsGsTsGATGGTusT
consensus/70%                       GAAAAuCTsTCuTCuTTsuCCAGsTGsGACTGCAusCCsTGuATGGCATTCACCACGGCsGGAAACsGsTsGATGGTusT

                    cov    pid 1441 .         .         .         .         .         .         .         . 1520
GA2a.Bcac.CL03T12C61 100.0% 100.0%  atcctttccttccaggacaaacacgaggtttattttttgcaggtccttttctatcttgttcttgaaggttccgtaataaa
GA2b.Bfra.CL05T12C13  99.7%  81.0%  ctcttttccttccaggacaaacatgaggtttattttttgcaggtccttctctatcttgtttttgaaagttccgtaataga
GA2c.Bvul.TF05-18     99.8%  82.0%  ctctttcccttccaggacgaacatgagatttatttttcgcaggtcctcctcaatcttgtgtttgaatgtgccgtaatata
GA2d.Buni.BIOML-A83   99.2%  69.8%  atctttcccttccactacaaatactatattgatatgttgtaaatcttgttcgattttgttcttaaaagtatcataataga
GA2e.Bsal.DSM.18170   99.7%  79.1%  ctcttttccttccagtacaaacatcaggtttatttctgcaagtccttctctatcttattcttgaaagtgctgtaataaa
consensus/100%                      sTCsTTsCCTTCCAssACuAAsAssAsuTTsATsTsssGsAuuTCsTssTCsATsTTuTssTTuAAsGTsssuTAATAsA
consensus/90%                       sTCsTTsCCTTCCAssACuAAsAssAsuTTsATsTsssGsAuuTCsTssTCsATsTTuTssTTuAAsGTsssuTAATAsA
consensus/80%                       sTCTTTsCCTTCCAGsACAAACAssAGuTTTATTTTTTGCAuGTCCTssTCsATCTTGTTsTTGAAuGTsCCGTAATAuA
consensus/70%                       sTCTTTsCCTTCCAGsACAAACAssAGuTTTATTTTTTGCAuGTCCTssTCsATCTTGTTsTTGAAuGTsCCGTAATAuA

                    cov    pid 1521 .         .         .         .         .         .         .         . 1600
GA2a.Bcac.CL03T12C61 100.0% 100.0%  tcggatggccgttcacgttcagcacatagctttcccgtttgtcaatgaccggcatcggcacatgcgttctgaaacagaac
GA2b.Bfra.CL05T12C13  99.7%  81.0%  ttggattgccgttgacattcagcacatagctttcccgtttgtcgatgaccggcatcgaaagccgggttttgaagcagaag
GA2c.Bvul.TF05-18     99.8%  82.0%  tcggatgcccgttgacgttcagcacatagctctcccgtttgtctatgaccggcatcggcacatgcgttctgaaacagaac
GA2d.Buni.BIOML-A83   99.2%  69.8%  ttggatttccatttacattcaagacataactgtctcccttgtcaataaccggcataggaagatgagtccgaaagcatagt
GA2e.Bsal.DSM.18170   99.7%  79.1%  tcggatttccgttaacattcagcacatagctttcctgtttgtcaatgatcggcatcggaatatgcgtcctgaaacacagg
consensus/100%                      TsGGATssCCuTTsACuTTCAusACATAuCTsTCssssTTGTCsATuAsCGGCATsGusAsssGsGTsssuAAuCAsAus
consensus/90%                       TsGGATssCCuTTsACuTTCAusACATAuCTsTCssssTTGTCsATuAsCGGCATsGusAsssGsGTsssuAAuCAsAus
consensus/80%                       TsGGATssCCGTTsACuTTCAGCACATAGCTsTCCCGTTTGTCuATGACCGGCATCGGsAsATGsGTsCTGAAuCAsAus
consensus/70%                       TsGGATssCCGTTsACuTTCAGCACATAGCTsTCCCGTTTGTCuATGACCGGCATCGGsAsATGsGTsCTGAAuCAsAus
```

```
                         cov    pid 1601 .       .         .         .         .         .         .         .         . 1680
GA2a.Bcac.CL03T12C61 100.0% 100.0%  gccgtatcattgtgccggtaggacaacaccggactgtaccggatggcgggatgccgcccggcaaactcccgaacctgccg
GA2b.Bfra.CL05T12C13  99.7%  81.0%  ctcgtgtcattgttccggtaggacagtaccgggctgtagcggatagcaggctggctggccgaaaattgcagtttccgctt
GA2c.Bvul.TF05-18     99.8%  82.0%  gccgtatcattgtgccggtaggacaacaccgggctgtaccggatggtgggttggttatcggcgaattgcagtttttgtcg
GA2d.Buni.BIOML-A83   99.2%  69.8%  gttgtatcactttctctataagaaagtaccggactatattttatagctggattaagtacagacaaattccggcttttgat
GA2e.Bsal.DSM.18170   99.7%  79.1%  ctcgtgtcattctcttttgtaaacaatacaggactgtaccatattgcaggctggctctctgcaaattccagtttctgcct
consensus/100%                      sssGTuTCAsTsTssssuTAuuAsAusACsGGuCTuTAssssATsGssGGsTssssssCsGssAAsTsCsGsssssssss
consensus/90%                       sssGTuTCAsTsTssssuTAuuAsAusACsGGuCTuTAssssATsGssGGsTssssssCsGssAAsTsCsGsssssssss
consensus/80%                       ssCGTuTCATTsTssCsGTAuGACAusACCGGuCTGTAsCusATuGCuGGsTGussssCsGsuAAsTsCsGssTsTGsss
consensus/70%                       ssCGTuTCATTsTssCsGTAuGACAusACCGGuCTGTAsCusATuGCuGGsTGussssCsGsuAAsTsCsGssTsTGsss

                         cov    pid 1681 .       .         .         .         .         .         .         .         . 1760
GA2a.Bcac.CL03T12C61 100.0% 100.0%  catatcatcggatgccagcgtcagggtatcctttcgttcactgtccttaaacaacagtgcgctgtcgggcagcgaggcga
GA2b.Bfra.CL05T12C13  99.7%  81.0%  caggtcgttggatgatagcggcaaggtgtctttccgttctttgtctttaaagacgagcgtgctgtccggcagcgaggtta
GA2c.Bvul.TF05-18     99.8%  82.0%  catctcatcggatgacagatgtaaggtgtccttccgttcccggtctttgaaaacaagcgtgctgtccggcagcgaggcta
GA2d.Buni.BIOML-A83   99.2%  69.8%  tctttcagaggcacataagtttatcgtatctccttcttctttatcaatcgtggtgaaaacgtccttaggcaatgacgcta
GA2e.Bsal.DSM.18170   99.7%  79.1%  catgtcgccggccgacagctgcaaggtgtcctttcgttccctgtctttgaagatgagcgtactgtccggcagcgaggcta
consensus/100%                      ssssTCussGGsssssAussssAssGTuTCssssssTTCsssuTCssTsussussAusususssTssGGCAusGAsGssA
consensus/90%                       ssssTCussGGsssssAussssAssGTuTCssssssTTCsssuTCssTsussussAusususssTssGGCAusGAsGssA
consensus/80%                       CATsTCussGGssGAsAGssssAuGGTuTCsTTsCGTTCssTGTCsTTuAAuAsuAGsGsGCTGTCsGGCAGCGAGGCTA
consensus/70%                       CATsTCussGGssGAsAGssssAuGGTuTCsTTsCGTTCssTGTCsTTuAAuAsuAGsGsGCTGTCsGGCAGCGAGGCTA

                         cov    pid 1761 .       .         .         .         .         .         .         .         . 1840
GA2a.Bcac.CL03T12C61 100.0% 100.0%  tatccacatgcaattgctggcctatttcggtcagcagccgccggtctatccacccgacaggcatccccccgatactgtcg
GA2b.Bfra.CL05T12C13  99.7%  81.0%  ggtctatatggagctgttgttcgggggcggtcagcaggcgtccgtctatccatccgatgacagcatgccctatgctgtct
GA2c.Bvul.TF05-18     99.8%  82.0%  tgtctatatggagctgctgttcgggagcggtcagcaacctcccgtccatccatccgatggcggtctgcccgatactgtct
GA2d.Buni.BIOML-A83   99.2%  69.8%  catctatgtgcaactgctgtccggaagatgtcaacaatctgttatcaatccatcctatagtttttagatcaatgctgtcc
GA2e.Bsal.DSM.18170   99.7%  79.1%  tgtctatatggagttgttgcctggcagcggtcagcagccctctgtctatccatccgacaaccgtctgtcctatgctgtcg
consensus/100%                      suTCsAsuTGsAusTGsTGssssusssssGTCAuCAusCssssuTCsATCCAsCCsAsuusssssssssCsATuCTGTCs
consensus/90%                       suTCsAsuTGsAusTGsTGssssusssssGTCAuCAusCssssuTCsATCCAsCCsAsuusssssssssCsATuCTGTCs
consensus/80%                       suTCTATATGsAusTGsTGssCGGsuGCGGTCAGCAusCssCsGTCsATCCATCCGAsuussuTssGsCCsATuCTGTCs
consensus/70%                       suTCTATATGsAusTGsTGssCGGsuGCGGTCAGCAusCssCsGTCsATCCATCCGAsuussuTssGsCCsATuCTGTCs

                         cov    pid 1841 .       .         .         .         .         .         .         .         . 1920
GA2a.Bcac.CL03T12C61 100.0% 100.0%  gcgtcgagtttgggccggtccgccacaagcacgcatcctttgtccgccgatgcctgataaggatacacaatgccgtagaa
GA2b.Bfra.CL05T12C13  99.7%  81.0%  gcgtcaagctgcggcttgtctgccacgagggtgcatcctttgtcttccgatatttgataaggatatacgacactgtaaaa
GA2c.Bvul.TF05-18     99.8%  82.0%  gcgtcaagttgcggtttgttcgccacgaggacacatcctttgtccgcagctgtttgataaggatacacgataccgtagaa
GA2d.Buni.BIOML-A83   99.2%  69.8%  gcatcaatcacggggctacaaccgacaagtatatgtgttccatctaccgctttcttgtaaggatacacaagactatacat
GA2e.Bsal.DSM.18170   99.7%  79.1%  gcgtcaagctgcggtttgtttgccacgagtacacatcccttgtcttccgatattttataagggtacacgatactgcaaaa
consensus/100%                      GCuTCuAsssssGGsssussssCsACuAGsususuTsssssuTCssCsGsTsssTsuTAAGGuTAsACuAsuCsusAsAs
consensus/90%                       GCuTCuAsssssGGsssussssCsACuAGsususuTsssssuTCssCsGsTsssTsuTAAGGuTAsACuAsuCsusAsAs
consensus/80%                       GCGTCAAGsTssGGssTGTssGCCACuAGsAsuCATCCTTTGTCssCCGsTuTsTsATAAGGATACACuAsACsGTAuAA
consensus/70%                       GCGTCAAGsTssGGssTGTssGCCACuAGsAsuCATCCTTTGTCssCCGsTuTsTsATAAGGATACACuAsACsGTAuAA
```

```
                    cov    pid 1921 .       .       .       .       .       .       .       .       . 2000
GA2a.Bcac.CL03T12C61 100.0% 100.0% cgggattctgcccgcttcctgtgtcaggtcggtatctttgaacagttttacggaatcatcgacgaaatatgtttccggtt
GA2b.Bfra.CL05T12C13  99.7%  81.0% tggaatcttgccagcctcttgtgtcaggtctgtattttgaacagttttacggagtcgttggcaaaatacgttttgggg
GA2c.Bvul.TF05-18     99.8%  82.0% tgggatttgcccgcttcttgcgtcaggtcgggattttaaacagctttacggaatcgttggcaaaataggtatcgggat
GA2d.Buni.BIOML-A83   99.2%  69.8% tggaatttaccaatctcttggtcaagtctgtgtttccgtatagctttatggaatcatttgcaaaatatttttcggcat
GA2e.Bsal.DSM.18170   99.7%  79.1% cggaatcttgccggcttcctgtgtcaggtcggtattttcaaacagtttcacagaatcattggcaaaatacgctgagggat
consensus/100%                     sGGuATssTuCCsussTCsTssGTCAuGTCsGsuTsTssusAsAGsTTsAsuGAuTCuTssuCuAAATAsssssssGsss
consensus/90%                      sGGuATssTuCCsussTCsTssGTCAuGTCsGsuTsTssusAsAGsTTsAsuGAuTCuTssuCuAAATAsssssssGsss
consensus/80%                      sGGuATsTTGCCsGCsTCsTGsGTCAGGTCsGTATTTTsuAACAGsTTTACGGAATCuTTGGCAAAATAsGTTTssGGuT
consensus/70%                      sGGuATsTTGCCsGCsTCsTGsGTCAGGTCsGTATTTTsuAACAGsTTTACGGAATCuTTGGCAAAATAsGTTTssGGuT
```

```
                    cov    pid 2001 .       .       .       .       .       .       .       .       . 2080
GA2a.Bcac.CL03T12C61 100.0% 100.0% cagccagtgccgcactgtcggccggcatcaccacctgcttgttcttgaagcccgtagctatgtcggtcacaccgcttcgg
GA2b.Bfra.CL05T12C13  99.7%  81.0% tggccaatgccacgctgtcgttcagcatgaccacctgtttgtttttgaatccggtaacaatgtcggtaaagccgctccgg
GA2c.Bvul.TF05-18     99.8%  82.0% cggcaaacgccagggtgtcggtcagcatgaccacctgcttgttcttgaatcccgtaacgacatcggtaaagccgctccgg
GA2d.Buni.BIOML-A83   99.2%  69.8% ttacaaacattgtggtatcatttggcaacagaaccatcttgtttttaaatcctgtcatgacatccgtaataccattcctt
GA2e.Bsal.DSM.18170   99.7%  79.1% tggccagtgccacggtgtcgctcagcatgaccacttgcttgttcttgaatcccgtgaggacatcggtaaatccgctccgg
consensus/100%                     ssuCsAusussususTuTCusssuGCAssAssACssssTTGTTsTTuAAsCCsGTsussAsuTCsGTsAssCCusTsCss
consensus/90%                      ssuCsAusussususTuTCusssuGCAssAssACssssTTGTTsTTuAAsCCsGTsussAsuTCsGTsAssCCusTsCss
consensus/80%                      suGCsAusGCCusGsTGTCGsTCuGCATsACCACCTGCTTGTTsTTGAATCCsGTuAsuAsuTCGGTAAsuCCGCTCCGG
consensus/70%                      suGCsAusGCCusGsTGTCGsTCuGCATsACCACCTGCTTGTTsTTGAATCCsGTuAsuAsuTCGGTAAsuCCGCTCCGG
```

```
                    cov    pid 2081 .       .       .       .       .       .       .       .       . 2160
GA2a.Bcac.CL03T12C61 100.0% 100.0% gtcagcaacaggttgtcccggttgatccatccgcaataccgggcctgcttccactctttcagccttccgtttttcaggat
GA2b.Bfra.CL05T12C13  99.7%  81.0% gtcagcaacaggtcgtttggttgatccatccgcagtattttgcctgtttccattctttcagtttcccgtttttcagaat
GA2c.Bvul.TF05-18     99.8%  82.0% gtcagcaacaggtcgttctggttgatccacccgcaatacttggcctgcttccactctttcagcctcccgtttttcaggat
GA2d.Buni.BIOML-A83   99.2%  69.8% gtcagcaaagttcattcttgttaatccaaccgacatatttcgcctttttccactcctttaatctgccattcttcaaaat
GA2e.Bsal.DSM.18170   99.7%  79.1% gtcagcagcaggtcgttctggttcatccagccgcaatactgggcctgtttccattctttcagcttcccgttcttcagaat
consensus/100%                     GTCAGCAusAGsTsuTssssGTTsATCCAsCCGssuTAssssGCCTssTTCCAsTCsTTsAussTsCCuTTsTTCAuuAT
consensus/90%                      GTCAGCAusAGsTsuTssssGTTsATCCAsCCGssuTAssssGCCTssTTCCAsTCsTTsAussTsCCuTTsTTCAuuAT
consensus/80%                      GTCAGCAACAGGTCGTTCTGGTTuATCCAsCCGCAATAsTssGCCTGsTTCCAsTCTTTCAGssTsCCGTTsTTCAGuAT
consensus/70%                      GTCAGCAACAGGTCGTTCTGGTTuATCCAsCCGCAATAsTssGCCTGsTTCCAsTCTTTCAGssTsCCGTTsTTCAGuAT
```

```
                    cov    pid 2161 .       .       .       .       .       .       .       .       . 2240
GA2a.Bcac.CL03T12C61 100.0% 100.0% ggcggggtcatactggatcaactggagccagtcgcctttgcgcttgatgaccagaaacgcatccatatagttcacggggc
GA2b.Bfra.CL05T12C13  99.7%  81.0% ggctgggtcgtattgaatgactctgagccagtctcctttcgcttgatgacgagaaatgcgtccatgtatttcacagact
GA2c.Bvul.TF05-18     99.8%  82.0% ggcggggtcgtaccggatcagccggagccagtcacctttcgtttgatgactaaaaacgcgtccatatattccaccgcct
GA2d.Buni.BIOML-A83   99.2%  69.8% agacggatcatatttatcagcttcaaccagtcacctttctctttatgaccagaaacgcatccatatactccacattat
GA2e.Bsal.DSM.18170   99.7%  79.1% ggcgggattatattggatcagtttgagccagtcccctttccgtttgatgaccagaaaggcatccatgtattccacggcct
consensus/100%                     uGssGGuTsuTAssssATsAsssssAuCCAGTCsCCTTTssssTTsATGACsAuAAAsGCuTCCATuTAsTsCACsssss
consensus/90%                      uGssGGuTsuTAssssATsAsssssAuCCAGTCsCCTTTssssTTsATGACsAuAAAsGCuTCCATuTAsTsCACsssss
consensus/80%                      GGCsGGuTCuTAsTGuATCAusssGAGCCAGTCsCCTTTsCGsTTGATGACsAGAAAsGCuTCCATuTAsTsCACuGssT
consensus/70%                      GGCsGGuTCuTAsTGuATCAusssGAGCCAGTCsCCTTTsCGsTTGATGACsAGAAAsGCuTCCATuTAsTsCACuGssT
```

```
                         cov    pid 2241 .       .         .         .         .         .         .         .         . 2320
GA2a.Bcac.CL03T12C61 100.0% 100.0%  gcatttcattcttcccgctcggactcaggtaggttgtccccggctcgttggcaaagacaatccacgggtcatgcttgaac
GA2b.Bfra.CL05T12C13  99.7%  81.0%  gcatctcgttctttccgctcggactcagataggtcgttcctgcttcattggcaaagacaatccacgggtatgcttgagc
GA2c.Bvul.TF05-18     99.8%  82.0%  gcatctcgttctttccgctcggactcagataggtcgttccggcttcacgggcaaaaacgatccacgggtatgcctgaac
GA2d.Buni.BIOML-A83   99.2%  69.8%  ccatcgcattctttccacttggactcatgtaagaagaggaagattcctgagcaaatacaatccacggattccccttgaat
GA2e.Bsal.DSM.18170   99.7%  79.1%  gcatctcgttttttccgctcggactcagatagcttgtcccttgttccttggcgaagacaatccacggatgatgcttgaac
consensus/100%                      sCATssCuTTsTTsCCuCTsGGACTCAsuTAusssGssssssssTCsssuGCuAAsACuATCCACGGusssssCsTGAus
consensus/90%                       sCATssCuTTsTTsCCuCTsGGACTCAsuTAusssGssssssssTCsssuGCuAAsACuATCCACGGusssssCsTGAus
consensus/80%                       GCATCTCuTTCTTTCCGCTCGGACTCAGuTAGGTsGTsCCsGsTTCsTsGGCAAAuACAATCCACGGussATGCTTGAAC
consensus/70%                       GCATCTCuTTCTTTCCGCTCGGACTCAGuTAGGTsGTsCCsGsTTCsTsGGCAAAuACAATCCACGGussATGCTTGAAC

                         cov    pid 2321 .       .         .         .         .         .         .         .         . 2400
GA2a.Bcac.CL03T12C61 100.0% 100.0%  agcgacaggctgcgggggactttcacgccttcgacggaatagttccgtacatattcgcgggggacattcttcacgcgtgt
GA2b.Bfra.CL05T12C13  99.7%  81.0%  agtgtctggctgcggggagcctttaccccttctatggaatagttccgcatatattcacggggtacattctttatccgcgt
GA2c.Bvul.TF05-18     99.8%  82.0%  agtgtctggctgcgtggaaccttcactccttccacggaatagttccgcatatactcacgggggacattctttatccgggt
GA2d.Buni.BIOML-A83   99.2%  69.8%  actgcaaaatcacgtggtgcttttaatccttctatacaataattgttcacgtactcacgaggtactttctttaccctcgt
GA2e.Bsal.DSM.18170   99.7%  79.1%  agcgacaggctgcggggggctttcactccttccaccgaatagttccgcacatattcgcgcggaacattcttcacccgtgt
consensus/100%                      AssGsssuussuCGsGGsuCsTTsAssCCTTCsAsssAATAuTTssssAsuTAsTCuCGsGGsACsTTCTTsAssCssGT
consensus/90%                       AssGsssuussuCGsGGsuCsTTsAssCCTTCsAsssAATAuTTssssAsuTAsTCuCGsGGsACsTTCTTsAssCssGT
consensus/80%                       AGsGsCsGGCTGCGsGGuuCsTTsACsCCTTCsAsuGAATAGTTCCGCAsATAsTCuCGuGGsACATTCTTsAsCCGsGT
consensus/70%                       AGsGsCsGGCTGCGsGGuuCsTTsACsCCTTCsAsuGAATAGTTCCGCAsATAsTCuCGuGGsACATTCTTsAsCCGsGT

                         cov    pid 2401 .       .         .         .         .         .         .         .         . 2480
GA2a.Bcac.CL03T12C61 100.0% 100.0%  aaaacggtgtaccggtccgcaggccgccag---acagataatgcttccgcaaatccatcctcttttcaacaggattgaca
GA2b.Bfra.CL05T12C13  99.7%  81.0%  aaaacggtgtaccggtccgcaggatgccat---acagatgatgcacccgcaaatccatcctccttttatgagtgtcgata
GA2c.Bvul.TF05-18     99.8%  82.0%  aaaacggtgtacaggtccgcaggatatcat---acaggcgatacatccgcaaatccatcctcctttaacaagtgccgata
GA2d.Buni.BIOML-A83   99.2%  69.8%  aaaacgatttaccggaccacatgctgccatcaaaccgataaaacaactgcacagacatccggcatggaacagacttgaca
GA2e.Bsal.DSM.18170   99.7%  79.1%  aaaacggtgtaccggcccgcaggctgtcag---gcaggcaatgct---gaaaatacaccctgtctgaatcagatgcgata
consensus/100%                      AAAACGuTsTACsGGsCCuCAsGssusCAs...uCsGusuAsuCs...GsAsAssCAsCCssssTssAssAGssssGAsA
consensus/90%                       AAAACGuTsTACsGGsCCuCAsGssusCAs...uCsGusuAsuCs...GsAsAssCAsCCssssTssAssAGssssGAsA
consensus/80%                       AAAACGGTGTACCGGsCCGCAGGsTGsCAs...ACAGusuATuCssCsGCAAATsCATCCTsssTssAssAGssssGAsA
consensus/70%                       AAAACGGTGTACCGGsCCGCAGGsTGsCAs...ACAGusuATuCssCsGCAAATsCATCCTsssTssAssAGssssGAsA

                         cov    pid 2481 .       .         .         .         .         .         .         .         . 2560
GA2a.Bcac.CL03T12C61 100.0% 100.0%  aacagttctttttcatgcctcacattacttttttgattcggaaaaacgttcgtgctgcgtcaccatcagttgcatcacgc
GA2b.Bfra.CL05T12C13  99.7%  81.0%  tgcaattctttttcatgcctcatttgttttttt--gttcggaaaagcgttcgtgctgtgttaccatcagctgcatcacac
GA2c.Bvul.TF05-18     99.8%  82.0%  tatagttctttttcatgcctcattctatttttttgattcggaaaagcgttcgtgctgggttaccatcaactgcatcacac
GA2d.Buni.BIOML-A83   99.2%  69.8%  atctatttcttttcataact-----taccttttttgttctgaaaaccgttcgtgctgagttaccatcagctgcataacac
GA2e.Bsal.DSM.18170   99.7%  79.1%  taaagttctttttcatggctcg--ttacttttttgattcggaaaaacgttcgtgttgggtcaccatcagctgcatgacgc
consensus/100%                      ssssuTTssTTTTCATusCT.....ssssTTTT..uTTCsGAAAAsCGTTCGTGsTGsGTsACCATCAusTGCATsACuC
consensus/90%                       ssssuTTssTTTTCATusCT.....ssssTTTT..uTTCsGAAAAsCGTTCGTGsTGsGTsACCATCAusTGCATsACuC
consensus/80%                       susAuTTCTTTTTCATGsCTCu..sTAsTTTTTTsuTTCGGAAAAuCGTTCGTGCTGsGTsACCATCAGCTGCATsACuC
consensus/70%                       susAuTTCTTTTTCATGsCTCu..sTAsTTTTTTsuTTCGGAAAAuCGTTCGTGCTGsGTsACCATCAGCTGCATsACuC
```

```
                      cov    pid 2561 .         .         .         .         .         .         .         .         . 2640
GA2a.Bcac.CL03T12C61 100.0% 100.0%  attcattgctcaggttatcacccatatccacaaacacctcgtcgatgagggtcttccgacgggcaatgattttcaatccc
GA2b.Bfra.CL05T12C13  99.7%  81.0%  attcgttgttcaggttgtctcccatatcgacgaacacctcgtctataagggttttcctgcgcgctatgattttcagtcct
GA2c.Bvul.TF05-18     99.8%  82.0%  attcgttgttcaggttgtctcccatatcgacgaacacctcgtctataagggttttcctgcgtgctatgattttcagtcct
GA2d.Buni.BIOML-A83   99.2%  69.8%  attcattatttagatcatcacccatatctacaaatacatcatcaataagtgtctttttacgggcaattattttcagacct
GA2e.Bsal.DSM.18170   99.7%  79.1%  attcgttgttcaggtcatcgcccatatcgacaaacacttcatcaatcacggttttctttcgcgcaatgattttcagcccc
consensus/100%                      ATTCuTTusTsAGuTsuTCsCCCATATCsACuAAsACsTCuTCsATsAssGTsTTssssCGsGCsATsATTTTCAusCCs
consensus/90%                       ATTCuTTusTsAGuTsuTCsCCCATATCsACuAAsACsTCuTCsATsAssGTsTTssssCGsGCsATsATTTTCAusCCs
consensus/80%                       ATTCuTTGTTCAGGTsuTCsCCCATATCsACuAACACsTCuTCsATuAGGGTsTTCsTuCGsGCsATGATTTTCAGsCCs
consensus/70%                       ATTCuTTGTTCAGGTsuTCsCCCATATCsACuAACACsTCuTCsATuAGGGTsTTCsTuCGsGCsATGATTTTCAGsCCs

                      cov    pid 2641 .         .         .         .         .         .         .         .         . 2720
GA2a.Bcac.CL03T12C61 100.0% 100.0%  tggcaataggagtagaagtcattctttttgttattgttgaccgtcaccgcaatgtccggatttccacagaggtacttctt
GA2b.Bfra.CL05T12C13  99.7%  81.0%  tggcaataggaatagaaatcattcttcttgctgttgttgacggttaccgctatgtccggatttccgcaaaggtatttctt
GA2c.Bvul.TF05-18     99.8%  82.0%  tggcaataggaatagaaatcattcttcttgctgttgttgacagttaccgctatgtccggatttccgcaaaggtatttttt
GA2d.Buni.BIOML-A83   99.2%  69.8%  tgacaataagaatagaaatcattctttttgttattatttatagtgacagccacatcaggattaccgcacaaatatttttt
GA2e.Bsal.DSM.18170   99.7%  79.1%  tggcagtaagaatagaagtcattctttttatggttgttgactgtgacggctatgtccggatttccacagagatatttctt
consensus/100%                      TGuCAuTAuGAuTAGAAuTCATTCTTsTTussuTTuTTsAssGTsACsGCsAsuTCsGGATTsCCuCAsAuuTAsTTsTT
consensus/90%                       TGuCAuTAuGAuTAGAAuTCATTCTTsTTussuTTuTTsAssGTsACsGCsAsuTCsGGATTsCCuCAsAuuTAsTTsTT
consensus/80%                       TGGCAATAuGAATAGAAuTCATTCTTsTTGsTuTTGTTGACsGTsACsGCsATGTCCGGATTTCCuCAuAGuTATTTsTT
consensus/70%                       TGGCAATAuGAATAGAAuTCATTCTTsTTGsTuTTGTTGACsGTsACsGCsATGTCCGGATTTCCuCAuAGuTATTTsTT

                      cov    pid 2721 .         .         .         .         .         .         .         .         . 2800
GA2a.Bcac.CL03T12C61 100.0% 100.0%  gaggatatagttgtaatggacattgaacggcttgccgtccacgatagcctgcaaatgctcgcggatgtcgttgccgataa
GA2b.Bfra.CL05T12C13  99.7%  81.0%  caggatataattgtaatgggtattgaacggcttgccgtccacgatggcttgcaagtgttcacggatgtcgttgccgatga
GA2c.Bvul.TF05-18     99.8%  82.0%  caggatataattgtaatgggtattgaacggcttgccgtccacgatggcctgcaagtgttcacggatgtcattaccgataa
GA2d.Buni.BIOML-A83   99.2%  69.8%  caatatatagttataatgagtgttgaatggctttccatccacgatagcttgcaaatgctcacgaatatcattccctataa
GA2e.Bsal.DSM.18170   99.7%  79.1%  caggatatagttataatgggtattgaacggtctgccgtccacgatggcctgcaaatgttctcggatatcgttcccgataa
consensus/100%                      sAusATATAuTTuTAATGuusuTTGAAsGGssTsCCuTCCACGATuGCsTGCAAuTGsTCsCGuATuTCuTTsCCsATuA
consensus/90%                       sAusATATAuTTuTAATGuusuTTGAAsGGssTsCCuTCCACGATuGCsTGCAAuTGsTCsCGuATuTCuTTsCCsATuA
consensus/80%                       CAGGATATAuTTuTAATGGGTATTGAACGGCTTGCCGTCCACGATuGCsTGCAAuTGsTCuCGGATuTCuTTsCCGATAA
consensus/70%                       CAGGATATAuTTuTAATGGGTATTGAACGGCTTGCCGTCCACGATuGCsTGCAAuTGsTCuCGGATuTCuTTsCCGATAA

                      cov    pid 2801 .         .         .         .         .         .         .         .         . 2880
GA2a.Bcac.CL03T12C61 100.0% 100.0%  cgacaagcacgtccgtcgagtcgttctccgcatattgcggaagaacctcaatggtgtgccggaccggatattgggtctct
GA2b.Bfra.CL05T12C13  99.7%  81.0%  tgacaagcacgtctgtcgagtcgttctccgtatattggggaaggatttcaatggtgtgccggaccggatattgggtattc
GA2c.Bvul.TF05-18     99.8%  82.0%  tgacaagcacgtccgtcgagtcgttctccgtatattggggaaggatttcaatggtgtgcctgaccggatattgggtattc
GA2d.Buni.BIOML-A83   99.2%  69.8%  ccaccaatatgtctgttgaatcattttcggtatattgaggaatgacttcaatcgtatgtcttatcgggtattgggtattc
GA2e.Bsal.DSM.18170   99.7%  79.1%  tgacaagcacgtccgtagagtcgttttctgtgtactgggaaaggattttaatcgtgtgccgtacggggtattgtgtgtct
consensus/100%                      ssACsAusAsGTCsGTsGAuTCuTTsTCsGsuTAsTGsGuAAsuAssTsAATsGTuTGsCssAssGGuTATTGsGTsTss
consensus/90%                       ssACsAusAsGTCsGTsGAuTCuTTsTCsGsuTAsTGsGuAAsuAssTsAATsGTuTGsCssAssGGuTATTGsGTsTss
consensus/80%                       sGACAAGCACGTCsGTsGAGTCGTTsTCsGTATATTGuGGAAGGAsTTCAATsGTGTGCCssACCGGuTATTGGGTuTss
consensus/70%                       sGACAAGCACGTCsGTsGAGTCGTTsTCsGTATATTGuGGAAGGAsTTCAATsGTGTGCCssACCGGuTATTGGGTuTss
```

```
                           cov     pid 2881 .         .         .         .         .         .         .         .         . 2960
GA2a.Bcac.CL03T12C61   100.0%  100.0%  tccgtggtcagcagtacctcgtacctgcccgcctcggagtaggcatagagcggattctgctcccgtgagtccacaatgcc
GA2b.Bfra.CL05T12C13    99.7%   81.0%  tccgtagtcagcagaacctcgtatatgccgggttctgtataggcgtagagtgggttctgctcccgcgaatcgacaattcc
GA2c.Bvul.TF05-18       99.8%   82.0%  tccgtagtcagcagaacctcgtatatgccgggttctgtataggcgtagagcgggttctgctcccgcgaatcgacaattcc
GA2d.Buni.BIOML-A83     99.2%   69.8%  tcagtcataagctgcacctcatatttgcctggctcagaataagaatataatggattctgctcccttgagtctacaatacc
GA2e.Bsal.DSM.18170     99.7%   79.1%  tccgtagtcagcagcacttcatattcgcctggttccgaataggcatagagcggattttgttctcgggaatcaacgattcc
consensus/100%                         TCsGTsuTsAGCsGsACsTCuTAsssGCCsGssTCsGsuTAuGsuTAsAusGGuTTsTGsTCsCssGAuTCsACuATsCC
consensus/90%                          TCsGTsuTsAGCsGsACsTCuTAsssGCCsGssTCsGsuTAuGsuTAsAusGGuTTsTGsTCsCssGAuTCsACuATsCC
consensus/80%                          TCCGTuGTCAGCAGsACCTCuTATsTGCCsGGsTCsGsATAGGCuTAGAGsGGuTTCTGCTCCCGsGAuTCsACAATsCC
consensus/70%                          TCCGTuGTCAGCAGsACCTCuTATsTGCCsGGsTCsGsATAGGCuTAGAGsGGuTTCTGCTCCCGsGAuTCsACAATsCC
```

```
                           cov     pid 2961 .         .         .         .         .         .         .         .         . 3040
GA2a.Bcac.CL03T12C61   100.0%  100.0%  cgattcaccgaactgccagcgccattccctcgacggcccgtaccccttgaatgaaatgatctcaccctggaaggcggtcg
GA2b.Bfra.CL05T12C13    99.7%   81.0%  ggattctccgaactgccagcgccattctttcgaaggaccgtatcctttgaaagatacgatttctccttgaaaggcggttg
GA2c.Bvul.TF05-18       99.8%   82.0%  ggactctccgaactgccagcgccattctttcgacggaccgtatcctttgaaagatacgatttctccttggaaggcggttg
GA2d.Buni.BIOML-A83     99.2%   69.8%  agactcaccaaactgccatctccattccttagatacaccataccctctgaatgtaacaatctcaccttgcatagcagttg
GA2e.Bsal.DSM.18170     99.7%   79.1%  tgactcgccgaattgccaccgccactctttcgaggggccgtatcctttgaaagacacgatttcaccttggaaagcgcttg
consensus/100%                         sGAsTCsCCuAAsTGCCAsCsCCAsTCssTsGAsussCCuTAsCCssTGAAsGssAsuATsTCsCCsTGsAsuGCusTsG
consensus/90%                          sGAsTCsCCuAAsTGCCAsCsCCAsTCssTsGAsussCCuTAsCCssTGAAsGssAsuATsTCsCCsTGsAsuGCusTsG
consensus/80%                          sGAsTCsCCGAACTGCCAsCGCCATTCsTTCGAsGGuCCGTAsCCTTTGAAsGAsACGATsTCsCCTTGuAAuGCGGTTG
consensus/70%                          sGAsTCsCCGAACTGCCAsCGCCATTCsTTCGAsGGuCCGTAsCCTTTGAAsGAsACGATsTCsCCTTGuAAuGCGGTTG
```

```
                           cov     pid 3041 .         .         .         .         .         .         .         .         . 3120
GA2a.Bcac.CL03T12C61   100.0%  100.0%  ccggcgcctctattttgaccagctggtcgcttccgtaattattggcccgtctgttcaccttgacgatttgcttcttctcc
GA2b.Bfra.CL05T12C13    99.7%   81.0%  ccggtgctttcatcctgacaagttcgtcgcttccgtaatcatcccggctcctgtgtactgtgatgatctgtttcctttcc
GA2c.Bvul.TF05-18       99.8%   82.0%  caggtgctttcatcctgacaagttcgtcgcttccgtaatcatcccggctcttgtgtactgtgatgatctgtttcctttcc
GA2d.Buni.BIOML-A83     99.2%   69.8%  acggagcttcaattttcaccagctgctcacttccataatcattcttttttcttcttacagtcacaatcttgctttcttcc
GA2e.Bsal.DSM.18170     99.7%   79.1%  ccggagctttcatttttacaagctgatcgctcccgtaagtgtttgttttcttccgtacggtgataatctgcttcttttcc
consensus/100%                         ssGGsGCsTssATssTsACsAGsTssTCuCTsCCuTAAssuTsssssssssTssssACssTsAsuATsTsssTssssTCC
consensus/90%                          ssGGsGCsTssATssTsACsAGsTssTCuCTsCCuTAAssuTsssssssssTssssACssTsAsuATsTsssTssssTCC
consensus/80%                          CCGGsGCTTssATssTsACsAGsTsuTCGCTTCCGTAATsATssssssTssTsssTACsGTGAsuATCTGsTTCsTTTCC
consensus/70%                          CCGGsGCTTssATssTsACsAGsTsuTCGCTTCCGTAATsATssssssTssTsssTACsGTGAsuATCTGsTTCsTTTCC
```

```
                           cov     pid 3121 .         .         .         .         .         .         .         .         . 3200
GA2a.Bcac.CL03T12C61   100.0%  100.0%  agcgaatggtccacctgaaggcggacctggtaacgccccggttccttgaacacgtactgtccgctccgttcatgcgaaac
GA2b.Bfra.CL05T12C13    99.7%   81.0%  agtcctccgtccacttgaagacgcacttggtagcgtcccggtttcttgaacacgtattctccgctccgttcgtgtgatat
GA2c.Bvul.TF05-18       99.8%   82.0%  agtcctccgtccacctgaaggcgtacttggtagcggccgggttccttgaacacatattctccgcttcgttcgtgtgatat
GA2d.Buni.BIOML-A83     99.2%   69.8%  agcgcatcatctattctgaggcgtatctgataacgtccaggctccataaaaatatattccccgctttgcttgtttgactt
GA2e.Bsal.DSM.18170     99.7%   79.1%  agcgaatggtccacttgcaggcgtacctggtaacgccccggttctttgaacacatattgcccgtttcgttcgtgtgatac
consensus/100%                         AGssssssuTCsAsssssAGuCGsAssTGuTAuCGsCCsGGsTsssTuAAsAsuTAsTssCCGsTssGsTsuTssGAsss
consensus/90%                          AGssssssuTCsAsssssAGuCGsAssTGuTAuCGsCCsGGsTsssTuAAsAsuTAsTssCCGsTssGsTsuTssGAsss
consensus/80%                          AGssssssGTCCACsTGuAGGCGsACsTGGTAuCGsCCsGGTTCCTTGAACACuTATTssCCGCTsCGTTCGTGTGAsAs
consensus/70%                          AGssssssGTCCACsTGuAGGCGsACsTGGTAuCGsCCsGGTTCCTTGAACACuTATTssCCGCTsCGTTCGTGTGAsAs
```

```
                          cov    pid 3201 .         .         .         .         .         .         .         .         . 3280
GA2a.Bcac.CL03T12C61 100.0% 100.0%  atccccgttgccgaactcccacagccaggaacccgcgttgcgggtgctgtccgcataatgaacggcttccccgacttcca
GA2b.Bfra.CL05T12C13  99.7%  81.0%  gtccccgttgccgaattcccaaagccaggagcgtgcgttgcgggtgctgtctgcataatggaccgtttctccggtttcca
GA2c.Bvul.TF05-18     99.8%  82.0%  gtccccgttgccgaactcccaaagccaggaacgtgcgttgcgggtgctgtccgcgtaatggacggcttctccggtttcca
GA2d.Buni.BIOML-A83   99.2%  69.8%  gtctccgttgccgaactcccaaagccatttttttgcattacgtgtactgtctgaatagtgaacaggttcgccgacttcta
GA2e.Bsal.DSM.18170   99.7%  79.1%  atccccgttgccgaactcccagagccatgagtttgccctgcgggtactgtccgcataatagataggttctccgacctcca
consensus/100%                      uTCsCCGTTGCCGAAsTCCCAsAGCCAsssssssGCssTuCGsGTuCTGTCsGsuTAuTuuAssGsTTCsCCGussTCsA
consensus/90%                       uTCsCCGTTGCCGAAsTCCCAsAGCCAsssssssGCssTuCGsGTuCTGTCsGsuTAuTuuAssGsTTCsCCGussTCsA
consensus/80%                       uTCCCCGTTGCCGAACTCCCAuAGCCAsGAussTGCuTTGCGGGTuCTGTCsGCATAATGuACuGsTTCsCCGusTTCCA
consensus/70%                       uTCCCCGTTGCCGAACTCCCAuAGCCAsGAussTGCuTTGCGGGTuCTGTCsGCATAATGuACuGsTTCsCCGusTTCCA

                          cov    pid 3281 .         .         .         .         .         .         .         .         . 3360
GA2a.Bcac.CL03T12C61 100.0% 100.0%  cctcgatgggcgtcagggtcatccgtacgatacggttggaaaaaaacagccggatcagcacggcgacaagcacacacagc
GA2b.Bfra.CL05T12C13  99.7%  81.0%  cctctatgggggtcagtgtcatacgcacggtgcggctggaaaagaacagccggatcagtatggcgacaaggatgcacagc
GA2c.Bvul.TF05-18     99.8%  82.0%  cctctatgggggtcagtgtcatccgtatggtacggttggaaaagaacagccggatcagtatggcgacaagaacgcatagc
GA2d.Buni.BIOML-A83   99.2%  69.8%  cctctatcggatttaatgtcatcctgattgtcctgcttgagaacaacaatcgtatcaatagagccagtactatacaaagc
GA2e.Bsal.DSM.18170   99.7%  79.1%  cttctatgggggtcagtgtcatccgcacggtccggctggagaaaaatatccggatcagcacggcaataagtatgcacagc
consensus/100%                      CsTCsATsGGssTsAusGTCATsCssAssuTsCsGsTsGAuAAsAAsAssCGsATCAusAsuGCsAssAssAsuCAsAGC
consensus/90%                       CsTCsATsGGssTsAusGTCATsCssAssuTsCsGsTsGAuAAsAAsAssCGsATCAusAsuGCsAssAssAsuCAsAGC
consensus/80%                       CCTCTATGGGuGTCAGTGTCATCCGsAsGGTsCGGsTGGAuAAuAACAuCCGGATCAGsAsGGCuAsAAGsAsuCAsAGC
consensus/70%                       CCTCTATGGGuGTCAGTGTCATCCGsAsGGTsCGGsTGGAuAAuAACAuCCGGATCAGsAsGGCuAsAAGsAsuCAsAGC

                          cov    pid 3361 .         .         .         .         .         .         .         .         . 3440
GA2a.Bcac.CL03T12C61 100.0% 100.0%  agcatcgctgcaactacaaacagaatctttctggtacgaatctttcccatggcgtcatttgtttttagttcccgagcgca
GA2b.Bfra.CL05T12C13  99.7%  81.0%  agcatggctgcgattacgaacagaatctttctggtataaatttttcccatgtcattt---gtttttagttcctgagcgca
GA2c.Bvul.TF05-18     99.8%  82.0%  agcatggctgcgattacgaacagaatctttctggtatagatttttcccatgtcattttccatttttagtgcccgagcgca
GA2d.Buni.BIOML-A83   99.2%  69.8%  aacaaagctcctataacaagcataaatttacgcgtataaatctttcccataatat--ttatttttttgtaccggaacgca
GA2e.Bsal.DSM.18170   99.7%  79.1%  agcatggctgccgctacaaacagaatctttctggtataaatctttcccatgtcat--tcattttttagtgcctgaacgca
consensus/100%                      AuCAssGCTsCsussACuAuCAsAAssTTsCssGTAsuuATsTTTCCCATussuT.....sTTTTTsGTsCCsGAuCGCA
consensus/90%                       AuCAssGCTsCsussACuAuCAsAAssTTsCssGTAsuuATsTTTCCCATussuT.....sTTTTTsGTsCCsGAuCGCA
consensus/80%                       AGCATuGCTGCsAsTACuAACAGAATCTTTCTGGTATAAATsTTTCCCATGsCAT..TsssTTTTTAGTsCCsGAuCGCA
consensus/70%                       AGCATuGCTGCsAsTACuAACAGAATCTTTCTGGTATAAATsTTTCCCATGsCAT..TsssTTTTTAGTsCCsGAuCGCA

                          cov    pid 3441 .         .         .         .         .         .         .         .         . 3520
GA2a.Bcac.CL03T12C61 100.0% 100.0%  ggtcttcctctttcttccgcaagcctatctcgcactcctccagattggctttgaacaggcgtatgttttgttcgatgctc
GA2b.Bfra.CL05T12C13  99.7%  81.0%  agtcttcctcctttttctgcaagcctatctcgcactcctccagattggctttgaacagacttatgttttgtccgatactc
GA2c.Bvul.TF05-18     99.8%  82.0%  ggtcttcctcttttttccgcaagccgatctcgcactcttccagattggctttgaacaggcttatgttttgttcgatgctc
GA2d.Buni.BIOML-A83   99.2%  69.8%  gatcttcaagtttcttctgcaatccaatttcacattcctccaaatttgccttgaacgtaatgatattctgctgaatactc
GA2e.Bsal.DSM.18170   99.7%  79.1%  agtcttcttcttttttctgcaatccgatctcgcattcttccagattggctttgaacagttgtatgttttgttcgatactc
consensus/100%                      uuTCTTCssssTTsTTCsGCAAsCCsATsTCuCAsTCsTCCAuATTsGCsTTGAACusssssATuTTsTGsssuATuCTC
consensus/90%                       uuTCTTCssssTTsTTCsGCAAsCCsATsTCuCAsTCsTCCAuATTsGCsTTGAACusssssATuTTsTGsssuATuCTC
consensus/80%                       uGTCTTCsTCTTTsTTCsGCAAsCCsATCTCGCAsTCsTCCAGATTGGCTTTGAACAGussTATGTTTTGTTCGATuCTC
consensus/70%                       uGTCTTCsTCTTTsTTCsGCAAsCCsATCTCGCAsTCsTCCAGATTGGCTTTGAACAGussTATGTTTTGTTCGATuCTC
```

```
                           cov     pid 3521 .         .         .         .         .         .         .         .         . 3600
GA2a.Bcac.CL03T12C61 100.0% 100.0%  cacagctgtttcttgtccgacagccacatggcataaaaatcggcgatatgcatgaacagcttgtaccgtttgtcccagct
GA2b.Bfra.CL05T12C13  99.7%  81.0%  catagctgtttcctgtcggacagccacatggcataaaagtcggcaatgtgcatgaacagcttgtatcgcttgtcccagct
GA2c.Bvul.TF05-18     99.8%  82.0%  catagctgtttcttgtcggacagccacatggcataaaagtcggcaatatgcatgaacagcttgtaacgcttgtcccagtt
GA2d.Buni.BIOML-A83   99.2%  69.8%  cacaattgctttttatccgacaaccacatgtcgtaaaaatcagcaatgtgcataaataatttatacctcttatcccaatt
GA2e.Bsal.DSM.18170   99.7%  79.1%  cacaattgttttttgtctgccaaccacatggcgtaaaagtcggctatgtgcataaacagcttgtaccgtttgtcccagct
consensus/100%                      CAsAusTGsTTssTuTCsGsCAuCCACATGsCuTAAAAuTCuGCsATuTGCATuAAsAusTTuTAsCssTTuTCCCAusT
consensus/90%                       CAsAusTGsTTssTuTCsGsCAuCCACATGsCuTAAAAuTCuGCsATuTGCATuAAsAusTTuTAsCssTTuTCCCAusT
consensus/80%                       CAsAusTGTTTsTTGTCsGACAuCCACATGGCuTAAAAuTCGGCuATuTGCATuAACAGCTTGTAsCGsTTGTCCCAGsT
consensus/70%                       CAsAusTGTTTsTTGTCsGACAuCCACATGGCuTAAAAuTCGGCuATuTGCATuAACAGCTTGTAsCGsTTGTCCCAGsT

                           cov     pid 3601 .         .         .         .         .         .         .         .         . 3680
GA2a.Bcac.CL03T12C61 100.0% 100.0%  gttcctttcgtaggtgttccgcaggttgttgatcaggtagtggatgtcgtcctcctcgtattgagcatacacgccgggct
GA2b.Bfra.CL05T12C13  99.7%  81.0%  gttcctttcgtatgtgttccgcaggttgtttatcagataatggatgtcgtcttcttcatactgggcgtaaacgcccggtt
GA2c.Bvul.TF05-18     99.8%  82.0%  gtttctttcgtatgtgttccgcaagttgtttatcagataatggatatcgtcctcctcatattgggcataaacgcccggtt
GA2d.Buni.BIOML-A83   99.2%  69.8%  gtttttttcgtatgtgtttttcagattgtttatcagataacgaatatcatcttcttcatattgagcatatacgcccggtt
GA2e.Bsal.DSM.18170   99.7%  79.1%  gtttctttcgtatgtgttgcgcaggttgtttatcaggtagtggatatcgtcttcttcatattgggcatgtacccctggtt
consensus/100%                      GTTssTTTCGTAsGTGTTsssCAuuTTGTTsATCAGuTAusGuATuTCuTCsTCsTCuTAsTGuGCuTusACsCCsGGsT
consensus/90%                       GTTssTTTCGTAsGTGTTsssCAuuTTGTTsATCAGuTAusGuATuTCuTCsTCsTCuTAsTGuGCuTusACsCCsGGsT
consensus/80%                       GTTsCTTTCGTATGTGTTsCGCAGGTTGTTTATCAGuTAuTGGATuTCGTCsTCsTCATATTGuGCATAsACGCCsGGTT
consensus/70%                       GTTsCTTTCGTATGTGTTsCGCAGGTTGTTTATCAGuTAuTGGATuTCGTCsTCsTCATATTGuGCATAsACGCCsGGTT

                           cov     pid 3681 .         .         .         .         .         .         .         .         . 3760
GA2a.Bcac.CL03T12C61 100.0% 100.0%  ggaaagcctctatcttccggaacagggagtccacgggcatctggctttcggcctgttcctgctggaagtccttgatacgg
GA2b.Bfra.CL05T12C13  99.7%  81.0%  caaatgtttctattttacggaacagggagtccaccggcaactggctttccgcttgttcctgctggaaatccttgatgcgg
GA2c.Bvul.TF05-18     99.8%  82.0%  caaacgcctcgatcttacggaacagggagtctgtcggaagctggcattccgcctgctcctgttggaattccttaatgcgg
GA2d.Buni.BIOML-A83   99.2%  69.8%  caaacttctctatcttacgaaacaaggaatcaacagaaacttgatgatctgcctgttcttgttgatagtccttgatacgg
GA2e.Bsal.DSM.18170   99.7%  79.1%  cgaaggtctcgattttacggaacagggaatctatggcctgttggctgtcggcttgttcctgttggaaatctttaatgcgg
consensus/100%                      suAAssssTCsATsTTsCGuAACAuGGAuTCsussGsssssTGusssTCsGCsTGsTCsTGsTGusAsTCsTTuATuCGG
consensus/90%                       suAAssssTCsATsTTsCGuAACAuGGAuTCsussGsssssTGusssTCsGCsTGsTCsTGsTGusAsTCsTTuATuCGG
consensus/80%                       CuAAsGsCTCsATsTTACGGAACAGGGAuTCsAssGusAssTGGCssTCsGCsTGTTCCTGsTGGAAuTCCTTuATuCGG
consensus/70%                       CuAAsGsCTCsATsTTACGGAACAGGGAuTCsAssGusAssTGGCssTCsGCsTGTTCCTGsTGGAAuTCCTTuATuCGG

                           cov     pid 3761 .         .         .         .         .         .         .         .         . 3840
GA2a.Bcac.CL03T12C61 100.0% 100.0%  ttcatcttgattttcacaaactccttctgctcgaacatcctgaagtccgaattccacatgaagatggcaaggcagcacgc
GA2b.Bfra.CL05T12C13  99.7%  81.0%  ttcatcttgatctttacgaactctttctgctcaaacatcttgaaatctgagttccacatgaagattaccagacaacacgc
GA2c.Bvul.TF05-18     99.8%  82.0%  ttcattttgattttcacgaactctttctgttcaaatatcttgaaatccgaattccacatgaatatcgccagacaacacgc
GA2d.Buni.BIOML-A83   99.2%  69.8%  ttcattttactttttacaaactctttctgctcaaacatactgaagtctgaattccacatgaatatactaatgcaacatat
GA2e.Bsal.DSM.18170   99.7%  79.1%  tccattttcattttcacgaactccttctcttcaaacatcttgaaatccgaattccacatgaatattgccaggcaacatac
consensus/100%                      TsCATsTTsAssTTsACuAACTCsTTCTssTCuAAsATssTGAAuTCsGAuTTCCACATGAAsATssssAsuCAuCAsus
consensus/90%                       TsCATsTTsAssTTsACuAACTCsTTCTssTCuAAsATssTGAAuTCsGAuTTCCACATGAAsATssssAsuCAuCAsus
consensus/80%                       TTCATsTTsATTTTsACuAACTCsTTCTGsTCAAACATCsTGAAuTCsGAATTCCACATGAAsATsuCsAGuCAACAsuC
consensus/70%                       TTCATsTTsATTTTsACuAACTCsTTCTGsTCAAACATCsTGAAuTCsGAATTCCACATGAAsATsuCsAGuCAACAsuC
```

```
                    cov    pid 3841 .        .         .         .         .         .         .         .         . 3920
GA2a.Bcac.CL03T12C61 100.0% 100.0%  cacggtcgtcaggaaaaaaaacaggaatacgtatgcgtgtcccataaggatttcccgtttgttcagtgtttttctattat
GA2b.Bfra.CL05T12C13  99.7%  81.0%  tacggttgtcaggaaaaagaacaggaatacataggcatgtcccatgaatatttcccgtttgttcattgtctttctgttgt
GA2c.Bvul.TF05-18     99.8%  82.0%  tacggtcgtcaggaaaaagaacaggaatacataggcatgccccatgaagatttcccgtttgttcattgtctttctgttgt
GA2d.Buni.BIOML-A83   99.2%  69.8%  tacagttgttatgataaagaacaggaatacataggcatgtcccataaagacttcccgtttattcaggttatttctattat
GA2e.Bsal.DSM.18170   99.7%  79.1%  cacggttgttacgaaaaagaacaggaatacataaatatgccccatcagggtctcccgcttgttcagtgtttttttattgt
consensus/100%                      sACuGTsGTsAsGAsAAAuAACAGGAATACuTAsusuTGsCCCATsAusussTCCCGsTTuTTCAsssTsTTTsTuTTuT
consensus/90%                       sACuGTsGTsAsGAsAAAuAACAGGAATACuTAsusuTGsCCCATsAusussTCCCGsTTuTTCAsssTsTTTsTuTTuT
consensus/80%                       sACGGTsGTsAsGAAAAAGAACAGGAATACATAuGCATGsCCCATuAuGATTTCCCGTTTGTTCAsTGTsTTTCTuTTuT
consensus/70%                       sACGGTsGTsAsGAAAAAGAACAGGAATACATAuGCATGsCCCATuAuGATTTCCCGTTTGTTCAsTGTsTTTCTuTTuT

                    cov    pid 3921 .        .         .         .         .         .         .         .         . 4000
GA2a.Bcac.CL03T12C61 100.0% 100.0%  tgtccatc-----atagcgttctcattgtttgttattgggtcccacactcaggctgcgccgtgtcttccagttgtcctgg
GA2b.Bfra.CL05T12C13  99.7%  81.0%  tgtccatggcttt-ccgtttattgtttgttgccgccggaacccacattcaggttacgtcgtgtttccagttgtcctgg
GA2c.Bvul.TF05-18     99.8%  82.0%  tatccataaatttacagtttatttatgattgttgttgggaccgacattcatgttacgccgtgtcttccagttatcctga
GA2d.Buni.BIOML-A83   99.2%  69.8%  tgtccataatcctctatttttat---ttgttattaccagagcctacattaagattcctacgagtcttccaattatccttg
GA2e.Bsal.DSM.18170   99.7%  79.1%  cattcataatatg-cagtttat------ttgttgttggaaccgacattcagattgcgacgtgtcttccagttgtcttgg
consensus/100%                      suTsCATs......sssssTTsT......TTussussuGusCCsACAsTsAsusTsCssCGsGTsTTCCAuTTuTCsTsu
consensus/90%                       suTsCATs......sssssTTsT......TTussussuGusCCsACAsTsAsusTsCssCGsGTsTTCCAuTTuTCsTsu
consensus/80%                       TuTCCATuussss.sAGTTTTAT...TssTTGTTussGGuuCCsACATTCAGuTTuCGsCGTGTCTTCCAGTTuTCCTGG
consensus/70%                       TuTCCATuussss.sAGTTTTAT...TssTTGTTussGGuuCCsACATTCAGuTTuCGsCGTGTCTTCCAGTTuTCCTGG

                    cov    pid 4001 .        .         .         .         .         .         .         .         . 4080
GA2a.Bcac.CL03T12C61 100.0% 100.0%  atgcactgcatcaggtcttttttaccatctcctcctgaatgttcaacaggcggatggaatccttcaccccgaggaatat
GA2b.Bfra.CL05T12C13  99.7%  81.0%  atgcactgcatcaggtctttcttcaccatctcttcctggatattcagcagacggatggagtctttaccccgaggaagat
GA2c.Bvul.TF05-18     99.8%  82.0%  atacattgcatcaggtctttcttcaccatctcttcctggatattcagcagacggatggagtcctttactccgaggaagat
GA2d.Buni.BIOML-A83   99.2%  69.8%  atacattgcatcaaatcatttttaccatttcctcctggatttcagtaaccggatagaatccttaactcctaagaacag
GA2e.Bsal.DSM.18170   99.7%  79.1%  atacattgcatcaggtctttctttaccatctcttcctgaatgctcagcaggcggatggagtctttacactcaggaacat
consensus/100%                      ATuCAsTGCATCAuuTCsTTsTTsACCATsTCsTCCTGuATssTCAusAusCGGATuGAuTCsTTsACsCssAuGAAsAs
consensus/90%                       ATuCAsTGCATCAuuTCsTTsTTsACCATsTCsTCCTGuATssTCAusAusCGGATuGAuTCsTTsACsCssAuGAAsAs
consensus/80%                       ATuCAsTGCATCAGGTCTTTsTTsACCATCTCsTCCTGuATuTTCAGCAGuCGGATGGAuTCsTTsACsCCsAGGAAsAT
consensus/70%                       ATuCAsTGCATCAGGTCTTTsTTsACCATCTCsTCCTGuATuTTCAGCAGuCGGATGGAuTCsTTsACsCCsAGGAAsAT

                    cov    pid 4081 .        .         .         .         .         .         .         .         . 4160
GA2a.Bcac.CL03T12C61 100.0% 100.0%  gttgatatttcccagcagccggctgtaaagcctacaatccctggcatccatgccctggatatccttcagcaggttcatct
GA2b.Bfra.CL05T12C13  99.7%  81.0%  gttgaggttttccagcagctgcctgtaaagtctgcaatctttggtatccatgccttggacatattgagcaggttcatct
GA2c.Bvul.TF05-18     99.8%  82.0%  gttgatgtttcccaaaagttgcctgtagagcctgcaatccttggcatccatgccctggacatattgagcaggttcatct
GA2d.Buni.BIOML-A83   99.2%  69.8%  attgatatttccaagcagttgtctgtacaatttacagtcccttcttccatatcctgtatgttttaaggatattcattt
GA2e.Bsal.DSM.18170   99.7%  79.1%  gttgatgtttcccagcagttgcttgtaaagcctgcaatccttgtcatccatactttgcatgtacttcaacaggttcattt
consensus/100%                      uTTGAsuTTTsCsAusAGssGssTGTAsAussTuCAuTCssTssssTCCATusssTGsAsuTssTTsAusAsuTTCATsT
consensus/90%                       uTTGAsuTTTsCsAusAGssGssTGTAsAussTuCAuTCssTssssTCCATusssTGsAsuTssTTsAusAsuTTCATsT
consensus/80%                       GTTGATuTTTCCCAGCAGsTGsCTGTAuAGsCTuCAATCCsTGsCATCCATuCCsTGsAsuTssTTsAGCAGGTTCATsT
consensus/70%                       GTTGATuTTTCCCAGCAGsTGsCTGTAuAGsCTuCAATCCsTGsCATCCATuCCsTGsAsuTssTTsAGCAGGTTCATsT
```

```
                         cov    pid  4161 .         .         .         .         .         .         .         .  4240
GA2a.Bcac.CL03T12C61  100.0% 100.0%       tgcgtgtgctgaccacactttgcaaaagcacgtcgttgatcttcggcgaggtgttcatcagtttcatgtattggtacacg
GA2b.Bfra.CL05T12C13   99.7%  81.0%       tgcgcgtactgactacgctttgcaggagcttgtcgtttatttgcggtgaagtgttcatcagtttcatgtactggtagatg
GA2c.Bvul.TF05-18      99.8%  82.0%       tgcgtgtactgacaacgctttgtaggagcatgtcgttgatctggggggaagtgttcatcagtttcatgtactggtagacg
GA2d.Buni.BIOML-A83    99.2%  69.8%       tttttgtactgattactccctgaagtaatacatcattaatctgtggagaagtgttcatcagtttcatatattggtagaca
GA2e.Bsal.DSM.18170    99.7%  79.1%       tccgcacgctgacaacgctttgcagaagaacatcgttaatctgtggcgaggtgttcatcagcttcatgtactgatagacc
consensus/100%                            TssssusuCTGAssACsCssTGsAusAusssuTCuTTsATsTssGGsGAuGTGTTCATCAGsTTCATuTAsTGuTAsAss
consensus/90%                             TssssusuCTGAssACsCssTGsAusAusssuTCuTTsATsTssGGsGAuGTGTTCATCAGsTTCATuTAsTGuTAsAss
consensus/80%                             TsCGsGTuCTGACsACuCTTTGsAGuAGsAsuTCGTTuATCTGsGGsGAuGTGTTCATCAGTTTCATGTAsTGGTAGACu
consensus/70%                             TsCGsGTuCTGACsACuCTTTGsAGuAGsAsuTCGTTuATCTGsGGsGAuGTGTTCATCAGTTTCATGTAsTGGTAGACu

                         cov    pid  4241 .         .         .         .         .         .         .         .  4320
GA2a.Bcac.CL03T12C61  100.0% 100.0%       gaatccacgctgtttgccaaggcgagttccctggcatagacatggtcgtattccagcgtctgctccgtaatccgtttcac
GA2b.Bfra.CL05T12C13   99.7%  81.0%       gagtccatgctgttcgacagggacagctctttggcatagatatggtcatattccagtgcctgctccgtgattcgctttac
GA2c.Bvul.TF05-18      99.8%  82.0%       gaatccacgctgttcgacagggacagctccttggcatagatgtggtcatactccagtgcctgttccgtaatccgctttac
GA2d.Buni.BIOML-A83    99.2%  69.8%       gaatccacactatttgaaagggctatttcacgggtatatacccggtcatactcctgggttttcgttgttattcttttaac
GA2e.Bsal.DSM.18170    99.7%  79.1%       gaatccacgctgtttgacaacgaaagttctttggcatagacatagtcatacttcaatgtctgttccgtgatccgtttcac
consensus/100%                            GAuTCCAsuCTuTTsGssAusGssAssTCsssGGsATAsAsssuGTCuTAsTsCsusGssTsssssGTsATsCssTTsAC
consensus/90%                             GAuTCCAsuCTuTTsGssAusGssAssTCsssGGsATAsAsssuGTCuTAsTsCsusGssTsssssGTsATsCssTTsAC
consensus/80%                             GAATCCACGCTGTTsGACAuGGssAGsTCssTGGCATAGAsuTGGTCATAsTCCAGsGsCTGsTCCGTuATsCGsTTsAC
consensus/70%                             GAATCCACGCTGTTsGACAuGGssAGsTCssTGGCATAGAsuTGGTCATAsTCCAGsGsCTGsTCCGTuATsCGsTTsAC

                         cov    pid  4321 .         .         .         .         .         .         .         .  4400
GA2a.Bcac.CL03T12C61  100.0% 100.0%       ctccgtgccggaggttttgaggaaacaggcaaagatggttaccgcaagcacgatacagcttgtcaggtagccggagaacc
GA2b.Bfra.CL05T12C13   99.7%  81.0%       ttctatgccggaggttttcaggaaacaggcaaagatgcttaccgcaaggattgtgcagcatgtcagatagcccgcgaatc
GA2c.Bvul.TF05-18      99.8%  82.0%       ttctatgccggaggtcttcaggaaacaggcaaagatggttaccgcaaggatgatgcagcatgtcagatagcctgcgaacc
GA2d.Buni.BIOML-A83    99.2%  69.8%       ctcaacgcctgatgtctttaagaaacaggcaaaaattaaaacggcaaatatgacgcaagccattaaataagcggcaaact
GA2e.Bsal.DSM.18170    99.7%  79.1%       ttccgtactggacgttttaaggaaactggcaaatatggccacggcaagaatgacgcaacctgtcaggtaacaggaaaatt
consensus/100%                            sTCsusuCssGAsGTsTTsAuGAAACsGGCAAAsATssssACsGCAAusAssusuCAusssuTsAuuTAusssGsuAAss
consensus/90%                             sTCsusuCssGAsGTsTTsAuGAAACsGGCAAAsATssssACsGCAAusAssusuCAusssuTsAuuTAusssGsuAAss
consensus/80%                             sTCsuTGCCGGAsGTsTTsAGGAAACAGGCAAAuATGussACsGCAAGsATGAsGCAuCsTGTCAGuTAuCCsGsuAAss
consensus/70%                             sTCsuTGCCGGAsGTsTTsAGGAAACAGGCAAAuATGussACsGCAAGsATGAsGCAuCsTGTCAGuTAuCCsGsuAAss

                         cov    pid  4401 .         .         .         .         .         .         .         .  4480
GA2a.Bcac.CL03T12C61  100.0% 100.0%       tcagataggcctttctcacttcggtgttatttttcggtttcatgtcggatgatgttttgtttctttataaataagtactg
GA2b.Bfra.CL05T12C13   99.7%  81.0%       tcagataagctttccttacttctgtgttgtttttcggtttcatataggatgatgtttta--tattcataaataggtgctg
GA2c.Bvul.TF05-18      99.8%  82.0%       tcaaataggctttccttacttctgtgttgtttttcggtttcatatagaataat-ttttg--tgtttataaataagtgctg
GA2d.Buni.BIOML-A83    99.2%  69.8%       tcaaatatgcttttcttacttctgaattattcctaggtttcata------ctcttatttttatttacaggtaactactt
GA2e.Bsal.DSM.18170    99.7%  79.1%       tcagataggctttccttacttccgtattatttctcggtttcatatcggataggtgttttatgggttacaaataagtgctg
consensus/100%                            TCAuATAsGCsTTsCTsACTTCsGsuTTuTTssTsGGTTTCATu......sss.ssTTs..sssTsAsAuuTAusTuCTs
consensus/90%                             TCAuATAsGCsTTsCTsACTTCsGsuTTuTTssTsGGTTTCATu......sss.ssTTs..sssTsAsAuuTAusTuCTs
consensus/80%                             TCAuATAuGCTTTsCTTACTTCsGTuTTuTTTsTCGGTTTCATATsGuATuussTTTTs..TuTTTAsAAATAAGTuCTG
consensus/70%                             TCAuATAuGCTTTsCTTACTTCsGTuTTuTTTsTCGGTTTCATATsGuATuussTTTTs..TuTTTAsAAATAAGTuCTG
```

```
                        cov     pid 4481 .         .         .         .         .         .         .         .         . 4560
GA2a.Bcac.CL03T12C61 100.0% 100.0%  attcccaggtacgcgcactgtccgtccggtgtacccagctggaaacgggcgtcctcctgttggatgatatagcggacttg
GA2b.Bfra.CL05T12C13  99.7%  81.0%  atacccagataggcgccttgttcggttggggttcccagccagaaccgggcgtcttcctgctggatgatgtagcggatctg
GA2c.Bvul.TF05-18     99.8%  82.0%  atgcccagataggcgccttgttcgtctgggatccccagtctgaaccgggcatcttcctgttggatgatgtagcggatctg
GA2d.Buni.BIOML-A83   99.2%  69.8%  attcctaaaaaagccccttgctcagaagagcaaccaagcttgaaacgagagtcttcctgctggattatgtaacgtgtatt
GA2e.Bsal.DSM.18170   99.7%  79.1%  attcctaaataggctccttgttccgttggatttcccagcttgaactgggcgtcctcctgctgtatgatataacggacttg
consensus/100%                      ATsCCsAuusAsGCsCssTGssCssssGussssCCsAGsssGAAssGuGsuTCsTCCTGsTGsATsATuTAuCGsussTs
consensus/90%                       ATsCCsAuusAsGCsCssTGssCssssGussssCCsAGsssGAAssGuGsuTCsTCCTGsTGsATsATuTAuCGsussTs
consensus/80%                       ATsCCsAuATAuGCsCCTTGTTCusssGGusTsCCCAGCssGAAsCGGGCGTCsTCCTGsTGGATGATuTAuCGGAssTG
consensus/70%                       ATsCCsAuATAuGCsCCTTGTTCusssGGusTsCCCAGCssGAAsCGGGCGTCsTCCTGsTGGATGATuTAuCGGAssTG

                        cov     pid 4561 .         .         .         .         .         .         .         .         . 4640
GA2a.Bcac.CL03T12C61 100.0% 100.0%  tacctccttgtccgatggcaggaacagctccatgagggcgttcaatatccggttgttggcggctcccgggaaaaaggtct
GA2b.Bfra.CL05T12C13  99.7%  81.0%  tatctccttgtccgagggaaggaacagttctgtcagcgcattcattattttactgtcggcggcgccgggaaagaagctct
GA2c.Bvul.TF05-18     99.8%  82.0%  tatctccttgtccgaagggaggaacagctccgccagcgcattcaatatttggctgtcgatggcaccggggaagaaacttt
GA2d.Buni.BIOML-A83   99.2%  69.8%  tattattttgtcggctggcaaaaaaagctcaaccaaagattgcaatatctgataatctttggctcccggagagaaacctt
GA2e.Bsal.DSM.18170   99.7%  79.1%  tatttctttgtcggcaggcaggaacaggtccatcaggtcgcagagtatctggcggtcaatggtatcggagaatagggtct
consensus/100%                      TAsssssTTGTCsGssGGsAuuAAsAGsTCsussAusssssssAsTATssussuTssssGGsssCsGuuuAsAuussssT
consensus/90%                       TAsssssTTGTCsGssGGsAuuAAsAGsTCsussAusssssssAsTATssussuTssssGGsssCsGuuuAsAuussssT
consensus/80%                       TATsTCsTTGTCsGssGGsAGGAACAGsTCsusCAGsGCuTsCAuTATsTGussGTCuusGGCsCCsGGuAAuAAusTsT
consensus/70%                       TATsTCsTTGTCsGssGGsAGGAACAGsTCsusCAGsGCuTsCAuTATsTGussGTCuusGGCsCCsGGuAAuAAusTsT

                        cov     pid 4641 .         .         .         .         .         .         .         .         . 4720
GA2a.Bcac.CL03T12C61 100.0% 100.0%  ccattttccgggcggacagggggccgatttccagcagcagccggtatgtgccgtcggcaaaacggttccccgccaccatc
GA2b.Bfra.CL05T12C13  99.7%  81.0%  ccatccgtcgggcggacaggggaccgatttccaccagcatctggtaggtgccgtcggcaaaactgtttcccagaaccatg
GA2c.Bvul.TF05-18     99.8%  82.0%  ccatccgccgggcgggcagggggccgatttccatcagcatccggaaagtaccgtcggcaaaactgtttcccagaaccata
GA2d.Buni.BIOML-A83   99.2%  69.8%  ccattctggatgcagagataggaccgatttcaagttccatttcataagtcccgtcatcaaaactattgccaattaccata
GA2e.Bsal.DSM.18170   99.7%  79.1%  ccatcttctgtgctggtagcgggccgatttccaacagcatttggtaagtgccgtcggcaaaacggttccccaggaccatg
consensus/100%                      CCATsssssusGCsGusAssGGuCCGATTTCsAssssCAssssusAsGTsCCGTCusCAAAACsuTTsCCsussACCATs
consensus/90%                       CCATsssssusGCsGusAssGGuCCGATTTCsAssssCAssssusAsGTsCCGTCusCAAAACsuTTsCCsussACCATs
consensus/80%                       CCATsssssGsGCuGusAGuGGuCCGATTTCCAsCAGCATssGGTAuGTuCCGTCGGCAAAACsGTTsCCCAssACCATu
consensus/70%                       CCATsssssGsGCuGusAGuGGuCCGATTTCCAsCAGCATssGGTAuGTuCCGTCGGCAAAACsGTTsCCCAssACCATu

                        cov     pid 4721 .         .         .         .         .         .         .         .         . 4800
GA2a.Bcac.CL03T12C61 100.0% 100.0%  gtgtcgcccaaaaggcaccggcctaaccgggggacaagaccgggaggcgcctctgtcacgcatttgtcgcctctcttcag
GA2b.Bfra.CL05T12C13  99.7%  81.0%  tcgtctcccagaatgcagcttcccaatttgggaataaggcaggaatccacttccaccacgcatttgtctcctctcttgat
GA2c.Bvul.TF05-18     99.8%  82.0%  ttgtctcccaggatacagcttcccaatttgggaataaggccggtatccgcttccactacgcacttgtctcctttcctgag
GA2d.Buni.BIOML-A83   99.2%  69.8%  ttgtctcccaaaatacactcacctaatttcgggacaatgtccggatcaacttccgtaacgcatttcccacccttttttcag
GA2e.Bsal.DSM.18170   99.7%  79.1%  gtgtcccccaaaaggcattctcccagtttagggacaagatgcgggtccgcttccaccacgcttttctcacctttctgcag
consensus/100%                      ssGTCsCCCAuuAsuCAssssCCsAussssGGuAsAAsusssGsusssuCsTCsussACGCssTTssCsCCssTssssAs
consensus/90%                       ssGTCsCCCAuuAsuCAssssCCsAussssGGuAsAAsusssGsusssuCsTCsussACGCssTTssCsCCssTssssAs
consensus/80%                       sTGTCsCCCAuAAsuCAssssCCsAATTTuGGuAsAAGusssGuATCCuCTTCCussACGCATTTsTCsCCTsTCTTsAG
consensus/70%                       sTGTCsCCCAuAAsuCAssssCCsAATTTuGGuAsAAGusssGuATCCuCTTCCussACGCATTTsTCsCCTsTCTTsAG
```

```
                      cov    pid 4801 .         .         .         .         .         .         .         .         . 4880
GA2a.Bcac.CL03T12C61 100.0% 100.0%   gcggacaggcaccccggtcaggatgctcatgcactcgcccgtcttcgcaaggctggcggtaatctgttccagatagggca
GA2b.Bfra.CL05T12C13  99.7%  81.0%   atgcacgggcacaccggtcagaatggtcatgcactcgccggtttggagaggctcgctgtaatctgttccatataggcca
GA2c.Bvul.TF05-18     99.8%  82.0%   acggacgggcacacccgtcaggatggtcatgcactcgccgatcttggagggactggccgtaatctgctccatataggcaa
GA2d.Buni.BIOML-A83   99.2%  69.8%   ggatacaggtacttccatcagtatgctcatacatttacctatattctccgtacttactgttatctgttccagataggtca
GA2e.Bsal.DSM.18170   99.7%  79.1%   gcggataggcactccggtcagcatgctcatgcactcgcttatttgggaaggactgacggtaatctgttccatataggcaa
consensus/100%                       ususAsuGGsACssCsuTCAGsATGsTCATuCAsTsuCssuTsTssssusuCTsuCsGTsATCTGsTCCAsATAGGssA
consensus/90%                        ususAsuGGsACssCsuTCAGsATGsTCATuCAsTsuCssuTsTssssusuCTsuCsGTsATCTGsTCCAsATAGGssA
consensus/80%                        usGsACuGGCACsCCsGTCAGsATGsTCATGCACTCGCCsuTsTTsGsuuGuCTsuCsGTAATCTGTTCCAsATAGGssA
consensus/70%                        usGsACuGGCACsCCsGTCAGsATGsTCATGCACTCGCCsuTsTTsGsuuGuCTsuCsGTAATCTGTTCCAsATAGGssA

                      cov    pid 4881 .         .         .         .         .         .         .         .         . 4960
GA2a.Bcac.CL03T12C61 100.0% 100.0%   gcagatggaccagcatgaccgccagatggagcggcagcagcttgagcaccggccactgtccggagaagatgctcacaaag
GA2b.Bfra.CL05T12C13  99.7%  81.0%   gcatatggacaagcatgaccgccagatgggtcggcagcaattccagtacgggccattgaccggaaaagatgcttacgaaa
GA2c.Bvul.TF05-18     99.8%  82.0%   gcatatggacaagcatgaccgccaaatgggttggcagcagttccagcacgggccattgtccggaaaagatactcacgaaa
GA2d.Buni.BIOML-A83   99.2%  69.8%   gcatgtgtatcagcattacagctaaatggacgggaagcaaatcaaggactggccattgccgggagaatatatttataaaa
GA2e.Bsal.DSM.18170   99.7%  79.1%   gcatgcggataagcataatcgccagatggaccggcagcagtttcagcaccggccactggtcggagaatatgcttacaaag
consensus/100%                       GCAsusGsAssAGCATsAssGCsAuATGGussGGsAGCAusTssAGsACsGGCCAsTGsssGGAuAAsATusTsAsuAAu
consensus/90%                        GCAsusGsAssAGCATsAssGCsAuATGGussGGsAGCAusTssAGsACsGGCCAsTGsssGGAuAAsATusTsAsuAAu
consensus/80%                        GCATuTGGAssAGCATuACCGCCAuATGGussGGCAGCAusTssAGsACsGGCCAsTGsCCGGAuAAsATuCTsACuAAu
consensus/70%                        GCATuTGGAssAGCATuACCGCCAuATGGussGGCAGCAusTssAGsACsGGCCAsTGsCCGGAuAAsATuCTsACuAAu

                      cov    pid 4961 .         .         .         .         .         .         .         .         . 5040
GA2a.Bcac.CL03T12C61 100.0% 100.0%   tcggcgtgcacgttcatctcgtcgatacggcgctccttgttctggaaagcgaccagcagatggtccagcgagatttcaaa
GA2b.Bfra.CL05T12C13  99.7%  81.0%   tccggatgtacgttcatctcgtcaatgcggcgttccttgttctggaaggctaccagcatatggtccagggagatttcgaa
GA2c.Bvul.TF05-18     99.8%  82.0%   tccggatgcacgttcatctcgtctatgcggcgttccttgtttggaaagtgaccagcatatggtccagggagatttcaaa
GA2d.Buni.BIOML-A83   99.2%  69.8%   tcccgatgaacattcatttcatcaatacgccgttccttgttttgaaaagcaaccagcatataatccaaggaaatttcgaa
GA2e.Bsal.DSM.18170   99.7%  79.1%   tccggatgtacgttcatttcatcgatacgtcgttccttgtttggaaactcaccagcatatagtccagggttatctcgaa
consensus/100%                       TCsssuTGsACuTTCATsTCuTCsATuCGsCGsTCCTTGTTsTGuAAusssACCAGCAsATuuTCCAusGssATsTCuAA
consensus/90%                        TCsssuTGsACuTTCATsTCuTCsATuCGsCGsTCCTTGTTsTGuAAusssACCAGCAsATuuTCCAusGssATsTCuAA
consensus/80%                        TCCGGATGsACGTTCATsTCuTCuATuCGsCGTTCCTTGTTsTGGAAAGssACCAGCATATuGTCCAGGGAuATTTCuAA
consensus/70%                        TCCGGATGsACGTTCATsTCuTCuATuCGsCGTTCCTTGTTsTGGAAAGssACCAGCATATuGTCCAGGGAuATTTCuAA

                      cov    pid 5041 .         .         .         .         .         .         .         .         . 5120
GA2a.Bcac.CL03T12C61 100.0% 100.0%   aggcttgaaaaaacgccgggcgaagaattcctcctcgcggtggatgcggatttcatccagtacctgttctttcgacactt
GA2b.Bfra.CL05T12C13  99.7%  81.0%   aggcttgaagaaacggcgggcgaaaaattccttcttcacggtggatgcggatttcgtccagcacctgttccttcgatattt
GA2c.Bvul.TF05-18     99.8%  82.0%   aggcttgaagaaacggcgggcgaagaactcctcctcccggtggacacggatttcatccagcacctgctccttcgatatct
GA2d.Buni.BIOML-A83   99.2%  69.8%   aggtttgaaaaatcttcttgcaaagaactcttcatcacgatggatacgaatctcctccaggacctgctcttttgaaattt
GA2e.Bsal.DSM.18170   99.7%  79.1%   aggcttgaaaaagcgccgggcatagaactcttcgtcccggtggatccggatttcatccagtatctgctctttggagcttt
consensus/100%                       AGGsTTGAAuAAsCssCssGCusAuAAsTCsTCsTCsCGuTGGAssCGuATsTCsTCCAGsAsCTGsTCsTTsGAssssT
consensus/90%                        AGGsTTGAAuAAsCssCssGCusAuAAsTCsTCsTCsCGuTGGAssCGuATsTCsTCCAGsAsCTGsTCsTTsGAssssT
consensus/80%                        AGGCTTGAAuAAuCGsCGGGCuAAGAAsTCsTCsTCsCGGTGGATuCGGATTTCuTCCAGsACCTGsTCsTTsGAsATTT
consensus/70%                        AGGCTTGAAuAAuCGsCGGGCuAAGAAsTCsTCsTCsCGGTGGATuCGGATTTCuTCCAGsACCTGsTCsTTsGAsATTT
```

```
                       cov     pid 5121 .       .         .         .         .         .         .         .         . 5200
GA2a.Bcac.CL03T12C61 100.0% 100.0%  tccccgactgatggagattcttgtggaagatgccttccggaagggcgtcgtaaatgctgcgcctgttcacgtggagatac
GA2b.Bfra.CL05T12C13  99.7%  81.0%  tctcggactggtaaaggttcttgtggaatatgccttcgggcagctcgtcataaaggcttctcctgttcacataaagatac
GA2c.Bvul.TF05-18     99.8%  82.0%  tttcggactggtaaaggttcttgtggaatatgccttcgggcagtgcgtcataaagactccgcctgttcacataaagatac
GA2d.Buni.BIOML-A83   99.2%  69.8%  ttcctgtctgataaaggttacgatgaaatatagactcaggtaacatgtcataaagactgcgcctgtttacatacagataa
GA2e.Bsal.DSM.18170   99.7%  79.1%  tgtcggactggtagaggttcttatgaaaaatgccttcaggcagtgtgtcataaatactccgcctgttcacgtagagatac
consensus/100%                      TssCsGsCTGuTuuAGuTTsssuTGuAAsATusssTCsGGsAusssGTCuTAAAsuCTsCsCCTGTTsACuTusAGATAs
consensus/90%                       TssCsGsCTGuTuuAGuTTsssuTGuAAsATusssTCsGGsAusssGTCuTAAAsuCTsCsCCTGTTsACuTusAGATAs
consensus/80%                       TssCsGACTGuTAuAGGTTCTTuTGuAAsATGCCTTCuGGsAGsusGTCATAAAsuCTsCGCCTGTTCACuTAuAGATAC
consensus/70%                       TssCsGACTGuTAuAGGTTCTTuTGuAAsATGCCTTCuGGsAGsusGTCATAAAsuCTsCGCCTGTTCACuTAuAGATAC

                       cov     pid 5201 .       .         .         .         .         .         .         .         . 5280
GA2a.Bcac.CL03T12C61 100.0% 100.0%  agataatccgtcaggtcgtaatgggagtattccgagcgtatttcctgaatgtccttcgagaagccgcgacgcatgtcgcc
GA2b.Bfra.CL05T12C13  99.7%  81.0%  agatagtctgtcaggtcataatgggaatattccgggtggatgtcatggatgtctttcgagaatccgcgccggatgtctcc
GA2c.Bvul.TF05-18     99.8%  82.0%  aggtagtccgtcaggtcgtaatgggaatattcagagtggatgtcatgaatgtctttcgagaatccgcgccgtatatcccc
GA2d.Buni.BIOML-A83   99.2%  69.8%  agataatcggtcaagtcatagtgtgaatattctgaatgtatatcatgaatatcctttgaataaccgcgtcttatatcccc
GA2e.Bsal.DSM.18170   99.7%  79.1%  aggtaatctgtcaggtcgtattgggaatattccgagtgggcatcatggatgtctttcgaaaagccacgccgtatgtcccc
consensus/100%                      AGuTAuTCsGTCAuGTCuTAsTGsGAuTATTCsGuusGsussTCsTGuATuTCsTTsGAusAsCCuCGsCssATuTCsCC
consensus/90%                       AGuTAuTCsGTCAuGTCuTAsTGsGAuTATTCsGuusGsussTCsTGuATuTCsTTsGAusAsCCuCGsCssATuTCsCC
consensus/80%                       AGuTAuTCsGTCAGGTCuTAuTGGGAATATTCsGAGTGsATuTCATGuATGTCsTTCGAuAAsCCGCGsCGsATuTCsCC
consensus/70%                       AGuTAuTCsGTCAGGTCuTAuTGGGAATATTCsGAGTGsATuTCATGuATGTCsTTCGAuAAsCCGCGsCGsATuTCsCC

                       cov     pid 5281 .       .         .         .         .         .         .         .         . 5360
GA2a.Bcac.CL03T12C61 100.0% 100.0%  ccgcctggagataagggtgcgttccgcgtcatacccgccctccttcagcccgctggccaccacttccgccttgaagtcgg
GA2b.Bfra.CL05T12C13  99.7%  81.0%  ccgtctgaatatgagggtacgctctgcatcatacccggcctctttcagtccgctggccaccacttctgccttgaagtcgg
GA2c.Bvul.TF05-18     99.8%  82.0%  ctttctggaaataagggtacgttccgcgtcatatccggcttctttcagcccgctggcaaccacttccgccttgaagtccg
GA2d.Buni.BIOML-A83   99.2%  69.8%  acgccttaaaatgagcgtccgttctgcatcataaccctgttctttcaatccactggcaacaacttcagccttgaaatcag
GA2e.Bsal.DSM.18170   99.7%  79.1%  caatctggaaatgagggtgcgctccgcgtcgtagccgctttctttcagtccgctggccaccacttctgccttgaaatcgg
consensus/100%                      ssssCTsuAsATuAGsGTsCGsTCsGCuTCuTAsCCssssTCsTTCAusCCuCTGGCsACsACTTCsGCCTTGAAuTCsG
consensus/90%                       ssssCTsuAsATuAGsGTsCGsTCsGCuTCuTAsCCssssTCsTTCAusCCuCTGGCsACsACTTCsGCCTTGAAuTCsG
consensus/80%                       CsusCTGuAuATuAGGGTuCGsTCsGCuTCATAsCCGsssTCTTTCAGsCCGCTGGCsACCACTTCsGCCTTGAAuTCuG
consensus/70%                       CsusCTGuAuATuAGGGTuCGsTCsGCuTCATAsCCGsssTCTTTCAGsCCGCTGGCsACCACTTCsGCCTTGAAuTCuG

                       cov     pid 5361 .       .         .         .         .         .         .         .         . 5440
GA2a.Bcac.CL03T12C61 100.0% 100.0%  tatccggctggttgaacagatgggggcgttcctcaaaacatacgatttgtctgtctcttctgccatggttgttcttgta
GA2b.Bfra.CL05T12C13  99.7%  81.0%  tatccggcaggttgaatgaatgggaatgttcgtcaaaccgtactatttatcttgttcttccgtcatgtccgttcgctta
GA2c.Bvul.TF05-18     99.8%  82.0%  tatccggcaggttgaacgaatggggatgttcgtcaaaacatacgatttatctgtttcttctgtcatggctgttcgttta
GA2d.Buni.BIOML-A83   99.2%  69.8%  tatcaggttgattgaatgcatgcacactctcatcaaaattcactatgttatctatttccttaccatacaaaaaactgtt
GA2e.Bsal.DSM.18170   99.7%  79.1%  tgtccggcaaattgaacgaatgtgggcgttcgtcaaaacatacgatttgtcagtttcttctgccataacttttcgggta
consensus/100%                      TuTCsGGssuuTTGAAsusATGsusussTCsTCAAAssssACsATsTTuTCssssTCsTssusCATusssssssssssTs
consensus/90%                       TuTCsGGssuuTTGAAsusATGsusussTCsTCAAAssssACsATsTTuTCssssTCsTssusCATusssssssssssTs
consensus/80%                       TATCCGGCsGuTTGAAsGuATGsGuusGTTCuTCAAAACuTACsATTTTuTCTuTTTCTTCTGsCATusssuTTCsssTA
consensus/70%                       TATCCGGCsGuTTGAAsGuATGsGuusGTTCuTCAAAACuTACsATTTTuTCTuTTTCTTCTGsCATusssuTTCsssTA
```

```
                            cov     pid 5441 .         .         .         .         .         .         .         .         . 5520
GA2a.Bcac.CL03T12C61 100.0% 100.0%  tgaat---tatcagtccaatatgttccagcgtgactcgttcgtctccttcggttgtctggagcgttcggcaaccacgatg
GA2b.Bfra.CL05T12C13  99.7%  81.0%  taggg---gattagtccagtatgttccagcgtgactcgctcgattctttcggttgtctggagcgttcggcgacaacgatg
GA2c.Bvul.TF05-18     99.8%  82.0%  tgagg---gattaatccaagatgttccagcgtgattcgctggactctttcggttgtctggaacgttcggcgaccacgatg
GA2d.Buni.BIOML-A83   99.2%  69.8%  taagtacagactaatccaaaatattccagcgtgtttctgtggtttccttagattgacgtgaacgctcagccacaacaatg
GA2e.Bsal.DSM.18170   99.7%  79.1%  ttcag---g-ttagtccaatatgttccaacgtgtctcgctggattccttggggtgtctggagcgttcggcaaccacgatg
consensus/100%                      Tssus...s.ssAuTCCAusATuTTCCAuCGTGssTCssTsGssTCsTTsGusTGsCssGAuCGsTCuGCsACsACuATG
consensus/90%                       Tssus...s.ssAuTCCAusATuTTCCAuCGTGssTCssTsGssTCsTTsGusTGsCssGAuCGsTCuGCsACsACuATG
consensus/80%                       Tuuus...GATTAuTCCAAsATGTTCCAGCGTGssTCGsTsGssTCsTTsGGTTGTCTGGAuCGTTCGGCuACsACGATG
consensus/70%                       Tuuus...GATTAuTCCAAsATGTTCCAGCGTGssTCGsTsGssTCsTTsGGTTGTCTGGAuCGTTCGGCuACsACGATG

                            cov     pid 5521 .         .         .         .         .         .         .         .         . 5600
GA2a.Bcac.CL03T12C61 100.0% 100.0%  ttttccttgtgcttgccgatgtattccagccggctgagcgtggtccacaagtcattgaactggtacaggaaacgctccgc
GA2b.Bfra.CL05T12C13  99.7%  81.0%  ttttccttgtgcttgccgatatattccagacggctgagggttgtccacaggtcattgaacaggtataggaaacgttccgc
GA2c.Bvul.TF05-18     99.8%  82.0%  ttctctttgtgcttgccgatatactccagacggctgagagtggtccacaggtcattgaacaggtataggaaacgttctgc
GA2d.Buni.BIOML-A83   99.2%  69.8%  ttttccttatgtttaccgatatattcaagacggctcaacgtagtccacaaatcattaaagaggtaaagaaagcgctctgt
GA2e.Bsal.DSM.18170   99.7%  79.1%  ttctccttgtgcttgccaatgtattcaaggcggctgagggtggtccacaagtcattgaacaggtataggaaacgctctgt
consensus/100%                      TTsTCsTTuTGsTTuCCuATuTAsTCsAGsCGGCTsAusGTsGTCCACAuuTCATTuAAssGGTAsAGuAAuCGsTCsGs
consensus/90%                       TTsTCsTTuTGsTTuCCuATuTAsTCsAGsCGGCTsAusGTsGTCCACAuuTCATTuAAssGGTAsAGuAAuCGsTCsGs
consensus/80%                       TTsTCCTTGTGCTTGCCGATuTATTCsAGuCGGCTGAGsGTuGTCCACAuGTCATTGAACAGGTAsAGGAAACGsTCsGs
consensus/70%                       TTsTCCTTGTGCTTGCCGATuTATTCsAGuCGGCTGAGsGTuGTCCACAuGTCATTGAACAGGTAsAGGAAACGsTCsGs

                            cov     pid 5601 .         .         .         .         .         .         .         .         . 5680
GA2a.Bcac.CL03T12C61 100.0% 100.0%  ggttatcatcagcggatggatgttctggtggttgtacaacaggccgctgttttcccgcaacagcccctcgtaagcgccgg
GA2b.Bfra.CL05T12C13  99.7%  81.0%  cgtaaccatcagttggcggatattgtggtggttgtacagcagcccgcagttttccctcagcaggccttcgtaggcaccgg
GA2c.Bvul.TF05-18     99.8%  82.0%  cgtaaccatcagctggcggatattgtggtggttgtacagcagaccgctgttttccctcagcagaccttcgtaagcgcctg
GA2d.Buni.BIOML-A83   99.2%  69.8%  cgttaccatcagtaaacgaatatcatggtggtcataacgcattccacaattttcacggagcacgccttcgtaagcaccgg
GA2e.Bsal.DSM.18170   99.7%  79.1%  tgttaccatcagttggcggatattgtgatggtcatacagcaagccgcaattttcccgcaacagcccttcgtatgcgccgg
consensus/100%                      sGTsAsCATCAGssuusGuATuTssTGuTGGTsuTAssuCAssCCuCsuTTTTCsCssAuCAssCCsTCGTAsGCuCCsG
consensus/90%                       sGTsAsCATCAGssuusGuATuTssTGuTGGTsuTAssuCAssCCuCsuTTTTCsCssAuCAssCCsTCGTAsGCuCCsG
consensus/80%                       sGTsACCATCAGssGuCGGATATTuTGGTGGTsuTACAGCAusCCGCsuTTTTCCCsCAuCAGsCCTTCGTAuGCuCCGG
consensus/70%                       sGTsACCATCAGssGuCGGATATTuTGGTGGTsuTACAGCAusCCGCsuTTTTCCCsCAuCAGsCCTTCGTAuGCuCCGG

                            cov     pid 5681 .         .         .         .         .         .         .         .         . 5760
GA2a.Bcac.CL03T12C61 100.0% 100.0%  gattgatgccgttccactcgttgaaatatttcagcaggtcctccttctcctccttgttcataaatcccaggcagacatac
GA2b.Bfra.CL05T12C13  99.7%  81.0%  gagtaatgccgttccattcgttgaagtatttcagcaggtcctccttgtcttccttgtccatgaaactcaggcatacatac
GA2c.Bvul.TF05-18     99.8%  82.0%  gggtgatgccgttccactcgttgaaatatttcagcaagtcctctttctcttccttgtccataaaactcaggcatacatac
GA2d.Buni.BIOML-A83   99.2%  69.8%  gcttgacgccattccattcataaaaatatttcaacaaatcttctttttcttctttacccataaagcccagacagatatat
GA2e.Bsal.DSM.18170   99.7%  79.1%  gagtgatgccgttccattcattgaagtatttcaacagatcttctttctcctccttgctcatgaaactcaggcatacgtac
consensus/100%                      GssTuAsGCCuTTCCAsTCuTsuAAuTATTTCAuCAuuTCsTCsTTsTCsTCsTTussCATuAAsCsCAGuCAsAsuTAs
consensus/90%                       GssTuAsGCCuTTCCAsTCuTsuAAuTATTTCAuCAuuTCsTCsTTsTCsTCsTTussCATuAAsCsCAGuCAsAsuTAs
consensus/80%                       GusTGATGCCGTTCCAsTCuTTGAAuTATTTCAuCAuuTCsTCsTTsTCsTCCTTGssCATuAAuCsCAGGCAsACATAC
consensus/70%                       GusTGATGCCGTTCCAsTCuTTGAAuTATTTCAuCAuuTCsTCsTTsTCsTCCTTGssCATuAAuCsCAGGCAsACATAC
```

```
                    cov     pid  5761 .         .         .         .         .         .         .         .         . 5840
GA2a.Bcac.CL03T12C61 100.0% 100.0%    aggcggtgtgccagcgtggagaatacgttcaggaaacggaggggcggataatactgcccttcgttacggtaggagaaata
GA2b.Bfra.CL05T12C13  99.7%  81.0%    aggcggtgagccaatgtggagaatacattcaggaagtgtagcggtgaatagtattgcccctcgttgcggaaggagaagta
GA2c.Bvul.TF05-18     99.8%  82.0%    agatgatgcgccaacgtggagaacacgttcaaaaagcgcagcggcgagtaatactgcccctcattgcggaaagagaaata
GA2d.Buni.BIOML-A83   99.2%  69.8%    aaacggtgcgcaagtgtcgaaaatacatttaagaaacgtataggcgaataatactgtccttcgttacggaatgaaaagta
GA2e.Bsal.DSM.18170   99.7%  79.1%    aggtggtgggccagtgtggagaatacattcagaaaacgcaagggggaatagtattgtccttcattacggtaggaaaaata
consensus/100%                        AuusGuTGsGCsAusGTsGAuAAsACuTTsAuuAAusGsAssGGsGuuTAuTAsTGsCCsTCuTTuCGGsAsGAuAAuTA
consensus/90%                         AuusGuTGsGCsAusGTsGAuAAsACuTTsAuuAAusGsAssGGsGuuTAuTAsTGsCCsTCuTTuCGGsAsGAuAAuTA
consensus/80%                         AGusGGTGsGCCAusGTGGAGAATACuTTCAuuAAuCGsAusGGsGAATAuTAsTGsCCsTCuTTuCGGsAuGAuAAuTA
consensus/70%                         AGusGGTGsGCCAusGTGGAGAATACuTTCAuuAAuCGsAusGGsGAATAuTAsTGsCCsTCuTTuCGGsAuGAuAAuTA

                    cov     pid  5841 .         .         .         .         .         .         .         .         . 5920
GA2a.Bcac.CL03T12C61 100.0% 100.0%    gatgtcggatatgaacagcatgacatggcggcacaacatgccgatgttcacggcaagcggggtctgcttctccgcattct
GA2b.Bfra.CL05T12C13  99.7%  81.0%    gatatcggatatgaacagcatgacatggcggcacagcataccgatgttggtggccaggctggtctgtttttctgcattct
GA2c.Bvul.TF05-18     99.8%  82.0%    gatgtcggatatgaacagcatgacatggcggcacagcataccgatgttggtggcaaggtcggtttgtttctccgcattct
GA2d.Buni.BIOML-A83   99.2%  69.8%    tatatcagatatatataacattacatgacggcataacattcctatatttgctgctaatttagtctgtttctctgcgttct
GA2e.Bsal.DSM.18170   99.7%  79.1%    aatgtcggatatgaaaagcatgatatggcggcacaacatgccgatgtttgtggccagtgccgtttgcttttccgcatttt
consensus/100%                        sATuTCuGATATusAsAuCATsAsATGuCGGCAsAuCATsCCsATuTTsussGCsAussssGTsTGsTTsTCsGCuTTsT
consensus/90%                         sATuTCuGATATusAsAuCATsAsATGuCGGCAsAuCATsCCsATuTTsussGCsAussssGTsTGsTTsTCsGCuTTsT
consensus/80%                         uATuTCGGATATGAAsAGCATGACATGGCGGCACAuCATuCCGATGTTsGsGGCsAGsssuGTsTGsTTsTCsGCATTCT
consensus/70%                         uATuTCGGATATGAAsAGCATGACATGGCGGCACAuCATuCCGATGTTsGsGGCsAGsssuGTsTGsTTsTCsGCATTCT

                    cov     pid  5921 .         .         .         .         .         .         .         .         . 6000
GA2a.Bcac.CL03T12C61 100.0% 100.0%    ccaccttggcgagaatatccgacgaggctttctcgatggaatcgaccagctgcccgaatttcagatagtagtttttcagg
GA2b.Bfra.CL05T12C13  99.7%  81.0%    ccactttggcaaggatgtcaaacgaggccttttctatggaatccacaagttgcccgaatttggtatagtagcctttcagg
GA2c.Bvul.TF05-18     99.8%  82.0%    ccaccttggcaaggatgtcaaacgaggctttctcgatggaatcgacaagctggccgaatttggtataatagcctttcagg
GA2d.Buni.BIOML-A83   99.2%  69.8%    ctacttttgccagaatatcaatagaagctttttctatcgaatctataagttgaccgaatttcgcataataacctttcagg
GA2e.Bsal.DSM.18170   99.7%  79.1%    ccaccttggcgagaatatcgaatgaggctttttcgatggaatcgacaagctgcccgaatttggtgtaatagttcttcagg
consensus/100%                        CsACsTTsGCsAGuATuTCsussGAuGCsTTsTCsATsGAATCsAssAGsTGsCCGAATTTsusuTAuTAusssTTCAGG
consensus/90%                         CsACsTTsGCsAGuATuTCsussGAuGCsTTsTCsATsGAATCsAssAGsTGsCCGAATTTsusuTAuTAusssTTCAGG
consensus/80%                         CCACsTTGGCuAGuATuTCuAAsGAGGCTTTsTCsATGGAATCsACAAGsTGsCCGAATTTsGsATAuTAGssTTTCAGG
consensus/70%                         CCACsTTGGCuAGuATuTCuAAsGAGGCTTTsTCsATGGAATCsACAAGsTGsCCGAATTTsGsATAuTAGssTTTCAGG

                    cov     pid  6001 .         .         .         .         .         .         .         .         . 6080
GA2a.Bcac.CL03T12C61 100.0% 100.0%    tccggatggctggacatgctggtacagggagggatgaagttcccgtccacctcgcagcggttcccgtttttgcgcaggcg
GA2b.Bfra.CL05T12C13  99.7%  81.0%    tccggatggctggacatgcttgtgcatggcgggatgaagtttccgtccacttcacagcggtttccgttcttgcgcaagcg
GA2c.Bvul.TF05-18     99.8%  82.0%    tccggatgactggacatgctcgtgcatggagggatgaagtttccgtccacctcgcaacggttcccgttcttacgcaggcg
GA2d.Buni.BIOML-A83   99.2%  69.8%    tccggatgactggacatactggaacatggaggtataaaattaccatccacttcacaacggtttccattcctacgcaatcg
GA2e.Bsal.DSM.18170   99.7%  79.1%    tcaggatggctggacatgctggtacagggaggtatgaagttaccgtccacttcgcagcggttgccgttcttgcgcaaacg
consensus/100%                        TCsGGATGuCTGGACATuCTsGsuCAsGGsGGsATuAAuTTsCCuTCCACsTCuCAuCGGTTsCCuTTssTuCGCAusCG
consensus/90%                         TCsGGATGuCTGGACATuCTsGsuCAsGGsGGsATuAAuTTsCCuTCCACsTCuCAuCGGTTsCCuTTssTuCGCAusCG
consensus/80%                         TCCGGATGuCTGGACATGCTsGTuCAsGGAGGsATGAAGTTsCCGTCCACsTCuCAuCGGTTsCCGTTCTTuCGCAuuCG
consensus/70%                         TCCGGATGuCTGGACATGCTsGTuCAsGGAGGsATGAAGTTsCCGTCCACsTCuCAuCGGTTsCCGTTCTTuCGCAuuCG
```

```
                          cov    pid  6081 .         .         .         .         .         .         .         .         . 6160
GA2a.Bcac.CL03T12C61 100.0% 100.0%  gccgattaccaggaagtgccttcccaggttgttgccgtcaatctgccccgtcggaacgacgctcaacgcgtagttgggca
GA2b.Bfra.CL05T12C13  99.7%  81.0%  gccgatcagcagaaagtgtcttcccagctcgcttccgtcggtctgtccgctcggcagcacgctcaatgcatatttgggta
GA2c.Bvul.TF05-18     99.8%  82.0%  gccgatcagcaggaagtgtcttcctaactcgcttccgtctgtctgtccggtcggtaacacgctcaatgcgtatttgggca
GA2d.Buni.BIOML-A83   99.2%  69.8%  gccgattacaagataatatcttcccagttcccctccatcaacctgtcctgccggcaatacattcaagcgatatttaggca
GA2e.Bsal.DSM.18170   99.7%  79.1%  accgatcagcaggaagtgcctgcctagctcgcctccgttggtttgccctgttggcagtacgctcaacgcatatttaggca
consensus/100%                      uCCGATsAssAGusAuTusCTsCCsAusTsssssCCuTssussTGsCCssssGGsAssACusTCAAsssuTAsTTuGGsA
consensus/90%                       uCCGATsAssAGusAuTusCTsCCsAusTsssssCCuTssussTGsCCssssGGsAssACusTCAAsssuTAsTTuGGsA
consensus/80%                       GCCGATsAsCAGuAAGTGsCTTCCsAGsTCGCsTCCGTCuuTCTGsCCsGTCGGsAusACGCTCAAsGCuTATTTuGGCA
consensus/70%                       GCCGATsAsCAGuAAGTGsCTTCCsAGsTCGCsTCCGTCuuTCTGsCCsGTCGGsAusACGCTCAAsGCuTATTTuGGCA

                          cov    pid  6161 .         .         .         .         .         .         .         .         . 6240
GA2a.Bcac.CL03T12C61 100.0% 100.0%  gcgcgtcaggctggcggggactggtctccttttcgtccgggatgcccgaaggcagacgctcgaaagggtctgccatcaga
GA2b.Bfra.CL05T12C13  99.7%  81.0%  aggcattgggctggcgtggactgatttccttctcgttcggaatacccgaaggaagatgctcgaaagggtctgccatcagt
GA2c.Bvul.TF05-18     99.8%  82.0%  gggcattgggctggcggggggcttatctctttctcgtccggaacgcccgatggcagatgctcgaaagggtctgccatcagt
GA2d.Buni.BIOML-A83   99.2%  69.8%  atgcgtccggttgtctgggtacactttctgtttcatccggagtaccggaaggctgcctttcaaacggatctgccatcaat
GA2e.Bsal.DSM.18170   99.7%  79.1%  atgcgttgggttgccgcggacttaattccttttcatccgggacacctgaaggacggtgctcgaacggatcagccatcagg
consensus/100%                      usGCuTssGGsTGsCssGGsssssssTCssTsTCuTsCGGuusuCCsGAsGGssGssssTCuAAsGGuTCsGCCATCAus
consensus/90%                       usGCuTssGGsTGsCssGGsssssssTCssTsTCuTsCGGuusuCCsGAsGGssGssssTCuAAsGGuTCsGCCATCAus
consensus/80%                       usGCuTsuGGsTGsCGsGGuCTsuTsTCsTTsTCuTCCGGuAsuCCsGAAGGssGusGCTCGAAsGGuTCTGCCATCAGs
consensus/70%                       usGCuTsuGGsTGsCGsGGuCTsuTsTCsTTsTCuTCCGGuAsuCCsGAAGGssGusGCTCGAAsGGuTCTGCCATCAGs

                          cov    pid  6241 .         .         .         .         .         .         .         .         . 6320
GA2a.Bcac.CL03T12C61 100.0% 100.0%  atgacatcccaccgctggtccttatcctccgtctcgtcaatggcgatatggaaactggcggtcagctcgtcgtctccccc
GA2b.Bfra.CL05T12C13  99.7%  81.0%  atgacatcccaccgcttgtccttttcttcttcctcgtctgtagggatatggaaactggcggtgagttcgtcatcctcacc
GA2c.Bvul.TF05-18     99.8%  82.0%  atgacatcccagcgccggtccttttcttccgtttcatccgtggagatatggaaactggcggtcagctcgtcatcgtcacc
GA2d.Buni.BIOML-A83   99.2%  69.8%  atcacatcccatttctgttctctatcatcactatcttcaacagaaatgtggaagctggctgtcagttcttcattttcatc
GA2e.Bsal.DSM.18170   99.7%  79.1%  atgacgtcccatcgcagggctttgtcttccatttcatcggcagaaatatggaaactggccgtcagttcatcattgtctcc
consensus/100%                      ATsACuTCCCAsssCssssCssTsTCsTCssssTCsTCsusuGsuATuTGGAAuCTGGCsGTsAGsTCsTCuTsssCssC
consensus/90%                       ATsACuTCCCAsssCssssCssTsTCsTCssssTCsTCsusuGsuATuTGGAAuCTGGCsGTsAGsTCsTCuTsssCssC
consensus/80%                       ATGACATCCCAsCGCsGGTCsTTsTCsTCssTsTCuTCsusuGuuATATGGAAACTGGCsGTCAGsTCuTCATssTCsCC
consensus/70%                       ATGACATCCCAsCGCsGGTCsTTsTCsTCssTsTCuTCsusuGuuATATGGAAACTGGCsGTCAGsTCuTCATssTCsCC

                          cov    pid  6321 .         .         .         .         .         .         .         .         . 6400
GA2a.Bcac.CL03T12C61 100.0% 100.0%  cgcgtcataatctatcaggtagccgccggcggtaagggcgaggcagcgtttcaaacggacttccagagtgcccgtgacaa
GA2b.Bfra.CL05T12C13  99.7%  81.0%  ggcatcataatctatcaggtaacctccggaagtaagggcgtggcaacgtttcagacggacttccaaggtgcctgtaacga
GA2c.Bvul.TF05-18     99.8%  82.0%  ggcatcgtaatctatcaggtagcctccggccgtaagggcatggcaacgtttcagacggacttccaaagtgccggtaacaa
GA2d.Buni.BIOML-A83   99.2%  69.8%  agcatcataatctataagatagcccccagcagtaacagcatggcaacgtttcaacctgacttcgagagtacctgtaacaa
GA2e.Bsal.DSM.18170   99.7%  79.1%  tgcgtcataatcaatcaggtatcctccggaagtaatggcatagcaccgtttcagacggacttccaaagttcccgtgacaa
consensus/100%                      sGCuTCuTAATCsATsAGuTAsCCsCCuGssGTAAsuGCusuGCAsCGTTTCAusCsGACTTCsAuuGTsCCsGTuACuA
consensus/90%                       sGCuTCuTAATCsATsAGuTAsCCsCCuGssGTAAsuGCusuGCAsCGTTTCAusCsGACTTCsAuuGTsCCsGTuACuA
consensus/80%                       sGCuTCATAATCTATCAGGTAuCCsCCGGsuGTAAsGGCuTGGCAuCGTTTCAuACGGACTTCCAuAGTuCCsGTuACAA
consensus/70%                       sGCuTCATAATCTATCAGGTAuCCsCCGGsuGTAAsGGCuTGGCAuCGTTTCAuACGGACTTCCAuAGTuCCsGTuACAA
```

```
                          cov    pid  6401 .         .         .         .         .         .         .         .         . 6480
GA2a.Bcac.CL03T12C61 100.0% 100.0%  gttccgtgatgctgaaatcaccggaaacggcttctcctttcctgaaggggagcaacccgtagttggcacggttcagctgg
GA2b.Bfra.CL05T12C13  99.7%  81.0%  gttccgtaatgctgaaatcgccggaaaccggttctcctttcttgaatggcaggagtccgtaggtattgcggttcagatga
GA2c.Bvul.TF05-18     99.8%  82.0%  gttccgtaatgctgaaatcaccggaaacggattctcctttttctgaacggcaggagtccgtaggtgttgcggttcagataa
GA2d.Buni.BIOML-A83   99.2%  69.8%  gctctgtaatgctgaaatcaccggaaacaggttctcctttttctgaaaggtaaaaagccatagtttgttctgttgagatga
GA2e.Bsal.DSM.18170   99.7%  79.1%  gttccgtgatgctgaaatctccggaaacgggttcccccttcttgaagggtaaaagcccgtagttggtgcggttcagatga
consensus/100%                      GsTCsGTuATGCTGAAATCsCCGGAAACsGsTTCsCCsTTssTGAAsGGsAusAusCCuTAGsTssssCsGTTsAGsTuu
consensus/90%                       GsTCsGTuATGCTGAAATCsCCGGAAACsGsTTCsCCsTTssTGAAsGGsAusAusCCuTAGsTssssCsGTTsAGsTuu
consensus/80%                       GTTCCGTuATGCTGAAATCuCCGGAAACuGuTTCTCCTTTssTGAAsGGsAuuAusCCGTAGsTusTuCGGTTCAGATGA
consensus/70%                       GTTCCGTuATGCTGAAATCuCCGGAAACuGuTTCTCCTTTssTGAAsGGsAuuAusCCGTAGsTusTuCGGTTCAGATGA

                          cov    pid  6481 .         .         .         .         .         .         .         .         . 6560
GA2a.Bcac.CL03T12C61 100.0% 100.0%  aatgactggtattcgcacagccggtccgtgaaatggtcctccagctggataaaatgcttcttgttgaccggcattccgtc
GA2b.Bfra.CL05T12C13  99.7%  81.0%  aatgactggtattcgcacaaccggtccgtaaagtggtcttcctgttgggtgaaatgccctttgttgacaggcattccgtc
GA2c.Bvul.TF05-18     99.8%  82.0%  aatgactggtattcgcacaaccgatccgtaaagtggtcttcctgttgcgtgaaatgccctttgttgacaggcattccgtc
GA2d.Buni.BIOML-A83   99.2%  69.8%  tacgactgatattcacacaactgattgatgaaatgatcttccagttgtatcaaatgatttttccttacaagcaatccgtc
GA2e.Bsal.DSM.18170   99.7%  79.1%  tacgactggtattcgcacagccggtgcgtaaaatggttttcctgctggacgaagtgttccttcttgaccggcatgccgtc
consensus/100%                      sAsGACTGuTATTCuCACAuCsGuTssuTuAAuTGuTssTCCsGsTGsussAAuTGssssTTssTsACsuGCAssCCGTC
consensus/90%                       sAsGACTGuTATTCuCACAuCsGuTssuTuAAuTGuTssTCCsGsTGsussAAuTGssssTTssTsACsuGCAssCCGTC
consensus/80%                       sAsGACTGGTATTCGCACAuCCGuTsCGTuAAuTGGTCTTCCsGsTGsuTuAAATGssssTTsTTGACsGGCATTCCGTC
consensus/70%                       sAsGACTGGTATTCGCACAuCCGuTsCGTuAAuTGGTCTTCCsGsTGsuTuAAATGssssTTsTTGACsGGCATTCCGTC

                          cov    pid  6561 .         .         .         .         .         .         .         .         . 6640
GA2a.Bcac.CL03T12C61 100.0% 100.0%  cgcccagtttaccagcggatatctctttcccggtttctgttttcctgtattcatcatgtctgtttttttaatattccttg
GA2b.Bfra.CL05T12C13  99.7%  81.0%  tgcccagtttaccagcggatattttttcttttggctctgttttcctatcataa-----tgtttctatttaatattcgtta
GA2c.Bvul.TF05-18     99.8%  82.0%  tgcccagtttaccagcgggtattttttcttttggctctgttttcctatcatga-----cgtctctatttaaaattcatta
GA2d.Buni.BIOML-A83   99.2%  69.8%  tgtccaatttaccagaggatatgatttccttttattctgtttcgttgccataa-----tccacaatattaatattctcta
GA2e.Bsal.DSM.18170   99.7%  79.1%  cgcccagttcacaagcggatagctgccccttttattttgatttcctgccatag-----tattgcgttttaatattcttta
consensus/100%                      sGsCCAuTTsACsAGsGGuTAssssssssssssssTsTGsTTsssTusssTsu.....sssssssssTTAAsATTCssTu
consensus/90%                       sGsCCAuTTsACsAGsGGuTAssssssssssssssTsTGsTTsssTusssTsu.....sssssssssTTAAsATTCssTu
consensus/80%                       sGCCCAGTTTACCAGCGGATATsTsTTCsTTTsusTCTGTTTTCCTusCATuA.....TsTsssssTTTAATATTCsTTA
consensus/70%                       sGCCCAGTTTACCAGCGGATATsTsTTCsTTTsusTCTGTTTTCCTusCATuA.....TsTsssssTTTAATATTCsTTA

                          cov    pid  6641 .         .         .         .         .         .         .         .         . 6720
GA2a.Bcac.CL03T12C61 100.0% 100.0%  taattcactatttcgttcgccacgcgcttggctatgatcacatcatggcttttcaaccggttctcggcaagcgtgacatc
GA2b.Bfra.CL05T12C13  99.7%  81.0%  tactttataagttcgtttgccacacgcttggctatgattacatcgtgtcttttcagcttgttgccggcaagtgtaacgtc
GA2c.Bvul.TF05-18     99.8%  82.0%  tactttacaagttcgttcgccacacgcttggctatgattacatcgtgctgtttcagcctgttgtcggcaagcgtgaggtc
GA2d.Buni.BIOML-A83   99.2%  69.8%  tattttaccaattcgtttgctacacgcttggcaataattacatcatgtcttttcagcttattttctgcaagtgttacttc
GA2e.Bsal.DSM.18170   99.7%  79.1%  tatgttactagttcattcgctacccgtttggcgataatcacatcatgcatcttcagcttgttttccgcaagcgtgagttc
consensus/100%                      TAssTsAssAsTTCuTTsGCsACsCGsTTGGCsATuATsACATCuTGssssTTCAuCssuTTssCsGCAAGsGTsAssTC
consensus/90%                       TAssTsAssAsTTCuTTsGCsACsCGsTTGGCsATuATsACATCuTGssssTTCAuCssuTTssCsGCAAGsGTsAssTC
consensus/80%                       TAsTTTACsAuTTCGTTsGCsACuCGCTTGGCsATuATsACATCuTGssTTTTCAGCsTGTTsTCsGCAAGsGTuAssTC
consensus/70%                       TAsTTTACsAuTTCGTTsGCsACuCGCTTGGCsATuATsACATCuTGssTTTTCAGCsTGTTsTCsGCAAGsGTuAssTC
```

```
                          cov     pid 6721 .         .         .         .         .         .         .         .         . 6800
GA2a.Bcac.CL03T12C61  100.0% 100.0%  cgggtctatatagaaccttctcttgaaaaaggaggatttggtatagaacacccattcgtaataggcgccgccgtcattct
GA2b.Bfra.CL05T12C13   99.7%  81.0%  ggggtctatatagcgccgtctcttgaaaaaggacggtttggtatagaatacccattcgtaataggctcctccttcattct
GA2c.Bvul.TF05-18      99.8%  82.0%  cgggtctatatagaaccgccttttgaaaaaggagggtttggtatagaatacccattcgtaatatgcgccgccctcgttct
GA2d.Buni.BIOML-A83    99.2%  69.8%  cggatctatatagaatctccttctgaagaaagacggtttagtataaaatacccattcgtaataagttccaccttcattac
GA2e.Bsal.DSM.18170    99.7%  79.1%  cgggtcaatatagaacctctttctgaaaaaagaggatttggtatagaatacccattcataatatgccccgccttcatttt
consensus/100%                       sGGuTCsATATAGsusCsssTssTGAAuAAuGAsGuTTTuGTATAuAAsACCCATTCuTAATAsGssCCsCCsTCuTTss
consensus/90%                        sGGuTCsATATAGsusCsssTssTGAAuAAuGAsGuTTTuGTATAuAAsACCCATTCuTAATAsGssCCsCCsTCuTTss
consensus/80%                        CGGGTCTATATAGAACCssCTssTGAAAAAuGAsGuTTTGGTATAGAATACCCATTCGTAATAsGCsCCuCCsTCATTsT
consensus/70%                        CGGGTCTATATAGAACCssCTssTGAAAAAuGAsGuTTTGGTATAGAATACCCATTCGTAATAsGCsCCuCCsTCATTsT

                          cov     pid 6801 .         .         .         .         .         .         .         .         . 6880
GA2a.Bcac.CL03T12C61  100.0% 100.0%  gatagacaatcggggaggacggcgatttgaggttgcagtcgtcaatcatgcgctggaaccaatgcccgaaaatgacatct
GA2b.Bfra.CL05T12C13   99.7%  81.0%  tatatacgatgggcgaagacggtgatttgaggttgcagtcgtcaatcatgcgctggaaccactgcccgaagatgacatcc
GA2c.Bvul.TF05-18      99.8%  82.0%  tgtacacgatgggagaggatggcgatttgaggttgcagtcgtcgatcatgcgctggaaccattgtccgaagatgacatcc
GA2d.Buni.BIOML-A83    99.2%  69.8%  gatattcaattggagctgacggagacttcagattacagtcatttatcatacgctgaaaccactgtccgaatatgacatcc
GA2e.Bsal.DSM.18170    99.7%  79.1%  gataaacaatgggcgaagaaggcgatttgaggttgcagtcatcaatcatgcgctggaaccattgcccgaaaatgacatca
consensus/100%                       suTAssCuATsGGsGssGAsGGsGAsTTsAGuTTuCAGTCuTssATCATuCGCTGuAACCAsTGsCCGAAsATGACATCs
consensus/90%                        suTAssCuATsGGsGssGAsGGsGAsTTsAGuTTuCAGTCuTssATCATuCGCTGuAACCAsTGsCCGAAsATGACATCs
consensus/80%                        sATAsACuATsGGsGAuGAsGGsGATTTGAGGTTGCAGTCuTCuATCATGCGCTGGAACCAsTGsCCGAAuATGACATCs
consensus/70%                        sATAsACuATsGGsGAuGAsGGsGATTTGAGGTTGCAGTCuTCuATCATGCGCTGGAACCAsTGsCCGAAuATGACATCs

                          cov     pid 6881 .         .         .         .         .         .         .         .         . 6960
GA2a.Bcac.CL03T12C61  100.0% 100.0%  tcggacgccttgccggtaatacgctccgcggcgggatcgtccaccttgcggaaaatttccacatccaccacgatgcttcg
GA2b.Bfra.CL05T12C13   99.7%  81.0%  tcggatgccttgccggttatgcgttccgatgcgggtcatccaccctgcggaatatgtccacatccattacgatgctccg
GA2c.Bvul.TF05-18      99.8%  82.0%  tcggatgccttgccggtaatacgttccgatgccggatcgtccaccttgcggaatatctccacatccatcacgatgctccg
GA2d.Buni.BIOML-A83    99.2%  69.8%  tccgaggctttccggtaattcgttcagcaaccgggtcatttacactttaaagacctccacatcaatcacaatactctt
GA2e.Bsal.DSM.18170    99.7%  79.1%  tcggaggccttgccggtaatacgttccgacactgggtcgtctaccctgcggaaaatgtccacgtcgatgacgatacttcg
consensus/100%                       TCsGAsGCsTTsCCGGTsATsCGsTCsGssuCsGGuTCuTssACssTsssuAAsAssTCCACuTCsAssACuATuCTsss
consensus/90%                        TCsGAsGCsTTsCCGGTsATsCGsTCsGssuCsGGuTCuTssACssTsssuAAsAssTCCACuTCsAssACuATuCTsss
consensus/80%                        TCGGAsGCCTTGCCGGTAATuCGTTCCGssuCsGGuTCuTCsACCsTGCGGAAsATsTCCACATCsATsACGATuCTsCG
consensus/70%                        TCGGAsGCCTTGCCGGTAATuCGTTCCGssuCsGGuTCuTCsACCsTGCGGAAsATsTCCACATCsATsACGATuCTsCG

                          cov     pid 6961 .         .         .         .         .         .         .         .         . 7040
GA2a.Bcac.CL03T12C61  100.0% 100.0%  ctcacggttgatgtacaaggagtccgaatcatactcttccgaatacttccagaccttgtatatttcaaggggtatgtcaa
GA2b.Bfra.CL05T12C13   99.7%  81.0%  ttcacggttgatgtacaaggtatccgagtcatattcctccgaatatttccatactttataaatttcaatgggtatgtcga
GA2c.Bvul.TF05-18      99.8%  82.0%  tttgcggttgatgcgcagggtgtccgagtcatattcctccgaatacttccagactttatagatttcaatgggtatgtcaa
GA2d.Buni.BIOML-A83    99.2%  69.8%  atcacgattgatatacaacgtatccgaatcatactcctctgaatacttccagactttatagatttccaaaggaatcgcaa
GA2e.Bsal.DSM.18170    99.7%  79.1%  ttcgcggttgatatacagggtttctgaatcgtattcttccgagtatttccacactttatagatttctacaggtatgtcga
consensus/100%                       sTsuCGuTTGATusuCAusGssTCsGAuTCuTAsTCsTCsGAuTAsTTCCAsACsTTuTAsATTTCsAsuGGsATssCuA
consensus/90%                        sTsuCGuTTGATusuCAusGssTCsGAuTCuTAsTCsTCsGAuTAsTTCCAsACsTTuTAsATTTCsAsuGGsATssCuA
consensus/80%                        sTCuCGGTTGATuTACAuGGTuTCCGAuTCATAsTCsTCCGAATAsTTCCAsACTTTATAuATTTCsAsuGGTATGTCuA
consensus/70%                        sTCuCGGTTGATuTACAuGGTuTCCGAuTCATAsTCsTCCGAATAsTTCCAsACTTTATAuATTTCsAsuGGTATGTCuA
```

```
                         cov    pid 7041 .         .         .         .         .         .         .         .  7120
GA2a.Bcac.CL03T12C61 100.0% 100.0%  aatagctgtttaccgtctgggtgtacaggagtgggaagacgaaaggcagcaggcaggtggaggcccagatgccgtactgg
GA2b.Bfra.CL05T12C13  99.7%  81.0%  aatagctgttcaccgtttgggaatacaggagtggaaatgcaaagggcagcaggcaggtggaggcccagatgccgtactgg
GA2c.Bvul.TF05-18     99.8%  82.0%  aatagctgttcaccgtttgggtatacaggagcgggaatacaaaaggcagcaggcaggtggaggcccagattccatactgg
GA2d.Buni.BIOML-A83   99.2%  69.8%  aatagcagtcaacagtctgtgcatataataaaggaaatgaaaaaggaagcaggcaggtggatgcccatattccatattgc
GA2e.Bsal.DSM.18170   99.7%  79.1%  aatagctttttaccgtctgggtatatagaaggggaaatacaaaaggcagtatgcaggtcgaagcccataccccgtattga
consensus/100%                      AATAGCssTssACsGTsTGsGsuTAsAusAusGGuAAsusuAAuGGsAGsAsGCAGGTsGAsGCCCAsAssCCuTAsTGs
consensus/90%                       AATAGCssTssACsGTsTGsGsuTAsAusAusGGuAAsusuAAuGGsAGsAsGCAGGTsGAsGCCCAsAssCCuTAsTGs
consensus/80%                       AATAGCTGTTsACCGTsTGGGsATAsAGuAGsGGuAATuCAAAAGGCAGCAGGCAGGTGGAuGCCCAsATsCCuTAsTGu
consensus/70%                       AATAGCTGTTsACCGTsTGGGsATAsAGuAGsGGuAATuCAAAAGGCAGCAGGCAGGTGGAuGCCCAsATsCCuTAsTGu

                         cov    pid 7121 .         .         .         .         .         .         .         .  7200
GA2a.Bcac.CL03T12C61 100.0% 100.0%  agttcgttgcagaggttgaacactacggagaagagccccgcgcccagcagcacgactgtaaccgtcagcaggcaccggat
GA2b.Bfra.CL05T12C13  99.7%  81.0%  agttcgttgcagaggttgaacactacggaaaagagtccggctcccagaagcatgaccgtaaccgtcagcaggcattggat
GA2c.Bvul.TF05-18     99.8%  82.0%  agttcgttgcagaggttgaagaccacggagaacagtccggctcccaatagcgtgaccgtaaccgtcagcaggcattggat
GA2d.Buni.BIOML-A83   99.2%  69.8%  agttcattgcaaagattgaaaaccaatgaaaaaagaccggctccgaacaacataaccgatactgtaatcagaaattggat
GA2e.Bsal.DSM.18170   99.7%  79.1%  agttcgttgcagaggttgaagacgaccgaaaaaattccggcacctaacagcacgaccgtgactgtcatcaggcactgtat
consensus/100%                      AGTTCuTTGCAuAGuTTGAAsACsAssGAuAAsAssCCsGCsCCsAusAuCusuACsGssACsGTsAsCAGusAssGsAT
consensus/90%                       AGTTCuTTGCAuAGuTTGAAsACsAssGAuAAsAssCCsGCsCCsAusAuCusuACsGssACsGTsAsCAGusAssGsAT
consensus/80%                       AGTTCGTTGCAGAGGTTGAAsACsACsGAuAAuAGsCCGGCsCCsAusAGCAsGACCGTuACsGTCAsCAGGCAsTGGAT
consensus/70%                       AGTTCGTTGCAGAGGTTGAAsACsACsGAuAAuAGsCCGGCsCCsAusAGCAsGACCGTuACsGTCAsCAGGCAsTGGAT

                         cov    pid 7201 .         .         .         .         .         .         .         .  7280
GA2a.Bcac.CL03T12C61 100.0% 100.0%  accgaaagagggtgtctccttgcccgaa---gcgaacactttccgcagcaggaaccggttgtaatacccggcggtccagt
GA2b.Bfra.CL05T12C13  99.7%  81.0%  gccgaaagaaggcatttccttgcttgat---gcgaacactttgcgaaggacgagccggttgtaatatccggcagtccagt
GA2c.Bvul.TF05-18     99.8%  82.0%  gccgaatgatggcatctccttgcccgag---gcgaataccttgcgcaggatgaaccggttataatgtccggcagtccagt
GA2d.Buni.BIOML-A83   99.2%  69.8%  tccaaatgaaggttgctcattgctggaggatgaaaatactttttctcaataccatcctgttgtaatatccagcgatccaat
GA2e.Bsal.DSM.18170   99.7%  79.1%  gccgaaagagggcatctccttgcccgaa---gaaaacactttgcgcaagacaaatcggttgtaatacccgccaatccagt
consensus/100%                      sCCuAAsGAsGGssssTCsTTGCssGAs...GsuAAsACsTTsCssAusAssAssCsGTTuTAATusCCusCuuTCCAuT
consensus/90%                       sCCuAAsGAsGGssssTCsTTGCssGAs...GsuAAsACsTTsCssAusAssAssCsGTTuTAATusCCusCuuTCCAuT
consensus/80%                       uCCGAAsGAuGGsuTCTCCTTGCssGAu...GsuAAsACTTTsCGCAusAsuAuCCGGTTGTAATAsCCGGCuuTCCAGT
consensus/70%                       uCCGAAsGAuGGsuTCTCCTTGCssGAu...GsuAAsACTTTsCGCAusAsuAuCCGGTTGTAATAsCCGGCuuTCCAGT

                         cov    pid 7281 .         .         .         .         .         .         .         .  7360
GA2a.Bcac.CL03T12C61 100.0% 100.0%  acaggatggcaagcaggacatagatgtagggcatgaagttgtagtccatgaatcccgacaggccgggcagggcaagtaca
GA2b.Bfra.CL05T12C13  99.7%  81.0%  acaggatgaccagcaggatataggcatagggcatgaagttatagtccatgaatccggacagaccgggcagggcaaggatt
GA2c.Bvul.TF05-18     99.8%  82.0%  acaggacaaccagcaggatatagacatagggcatgaagctatagtccatgaacccgcacagggcgggcagggcaagaatc
GA2d.Buni.BIOML-A83   99.2%  69.8%  aaactacaaccaataaaatatacatatatggcataaagttatagttcatgaaacccgccagtcctggtaacgctagtaca
GA2e.Bsal.DSM.18170   99.7%  79.1%  ataggataaccagcaggatatagatataaggcatgaaattgtagtccataaatccggacaggccgggcagggcaagcacg
consensus/100%                      AsAssAsuuCsAusAuuAsATAsusuTAsGGCATuAAusTuTAGTsCATuAAsCCsssCAGssCsGGsAusGCsAGsAss
consensus/90%                       AsAssAsuuCsAusAuuAsATAsusuTAsGGCATuAAusTuTAGTsCATuAAsCCsssCAGssCsGGsAusGCsAGsAss
consensus/80%                       AsAGGAsuACCAGCAGGATATAGAsATAuGGCATGAAGTTuTAGTCCATGAAsCCsGACAGuCCGGGCAGGGCAAGsAss
consensus/70%                       AsAGGAsuACCAGCAGGATATAGAsATAuGGCATGAAGTTuTAGTCCATGAAsCCsGACAGuCCGGGCAGGGCAAGsAss
```

```
                      cov     pid 7361 .         .         .         .         .         .         .         .         . 7440
GA2a.Bcac.CL03T12C61 100.0% 100.0%  atgccgcacagcagcagatagacaatcagtttcttattgttgagcagcttgttcttccgtgcgataaaataggctgcaat
GA2b.Bfra.CL05T12C13  99.7%  81.0%  atgccgcacagcagcagatacgcaatcagtttttgttgttgagcagcttgttcttgcgtgcgataaaataggctgcgat
GA2c.Bvul.TF05-18     99.8%  82.0%  atgccgcacaacagcagatacacgatcagttttctattgttgagcagtttgttcttgcgtgcgataaaataggccgcgat
GA2d.Buni.BIOML-A83   99.2%  69.8%  ataccactcagaagcaaatatacaataagcctcttattattaagcagtttgtttttcctggcaataaaatatgccataac
GA2e.Bsal.DSM.18170   99.7%  79.1%  atactgcacacgagcagataaacaatcagtctcttgttgttcaatagtctgttcttccgtgcgacgaaataggctgctat
consensus/100%                      ATuCsuCsCAssAGCAuATAsuCuATsAGssTssTuTTuTTsAusAGssTGTTsTTsCssGCuAsuAAATAsGCsussAs
consensus/90%                       ATuCsuCsCAssAGCAuATAsuCuATsAGssTssTuTTuTTsAusAGssTGTTsTTsCssGCuAsuAAATAsGCsussAs
consensus/80%                       ATuCCGCACAusAGCAGATAsACAATCAGTsTsTTuTTGTTuAGCAGsTTGTTCTTsCGTGCGATAAAATAGGCsGCuAT
consensus/70%                       ATuCCGCACAusAGCAGATAsACAATCAGTsTsTTuTTGTTuAGCAGsTTGTTCTTsCGTGCGATAAAATAGGCsGCuAT

                      cov     pid 7441 .         .         .         .         .         .         .         .         . 7520
GA2a.Bcac.CL03T12C61 100.0% 100.0%  cccgaaaatcacagtcagtacgggcatcagcaggtacgatgaaatcagatgttgggtgacagggtccataag---ttcta
GA2b.Bfra.CL05T12C13  99.7%  81.0%  tccgaaaatcacggtcagcagcggcatcagcaggtacgatgaaatgagatgttgggtaactgggttcataag---tatgg
GA2c.Bvul.TF05-18     99.8%  82.0%  tccgaaaatcacggttagcagcggcatcagcaggtacgatgaaatgagatgttgggtaactgggttcataag---tatga
GA2d.Buni.BIOML-A83   99.2%  69.8%  tccaaatattatggtaaggagaggcatgagcaaatacgatgatacaaaatgtagtgcaactggattcatata---aaatc
GA2e.Bsal.DSM.18170   99.7%  79.1%  cccgaaaattacggtaagcaggggcatcagcagatacgatgaaataagatgttgggtaactgggtccatatgctataagt
consensus/100%                      sCCuAAsATsAsuGTsAGsAssGGCATsAGCAuuTACGATGAsAssAuATGTsGsGsuACsGGuTsCATAsu...sssss
consensus/90%                       sCCuAAsATsAsuGTsAGsAssGGCATsAGCAuuTACGATGAsAssAuATGTsGsGsuACsGGuTsCATAsu...sssss
consensus/80%                       sCCGAAAATsACGGTsAGsAGsGGCATCAGCAGuTACGATGAAATuAGATGTTGGGTAACTGGGTsCATAsG...TAsss
consensus/70%                       sCCGAAAATsACGGTsAGsAGsGGCATCAGCAGuTACGATGAAATuAGATGTTGGGTAACTGGGTsCATAsG...TAsss

                      cov     pid 7521 .         .         .         .         .         .         .         .         . 7600
GA2a.Bcac.CL03T12C61 100.0% 100.0%  tgtttatgcgttattgtattcgatgaatatgcggtaattgaatgtgtccggcgagcaggcagacagtctggtgtgcagac
GA2b.Bfra.CL05T12C13  99.7%  81.0%  tatttatgctttattgtattctatgaatatccggtaattgaatgtgtccggtgaacaggccgaaagtttggtacgcaggt
GA2c.Bvul.TF05-18     99.8%  82.0%  tatttatgctttattgtattctatgaatatccggtagttgaatgtgtccggcgaacaggccgagagtttggtacgcaggt
GA2d.Buni.BIOML-A83   99.2%  69.8%  tgtttatgcgttattatattcaataaatatcctatagttaaaagtatccggagagcaggctgccaatttggtaagcagat
GA2e.Bsal.DSM.18170   99.7%  79.1%  tatttaggctttattgtattcgatgaatatccggtaattgaatgtatctggcgaacaggctgaaagcttggttctcagat
consensus/100%                      TuTTTAsGCsTTATTuTATTCsATuAATATsCsuTAuTTuAAsGTuTCsGGsGAuCAGGCsGssAussTGGTsssCAGus
consensus/90%                       TuTTTAsGCsTTATTuTATTCsATuAATATsCsuTAuTTuAAsGTuTCsGGsGAuCAGGCsGssAussTGGTsssCAGus
consensus/80%                       TuTTTATGCsTTATTGTATTCsATGAATATCCGGTAuTTGAATGTuTCCGGsGAuCAGGCsGAsAGTTTGGTusGCAGuT
consensus/70%                       TuTTTATGCsTTATTGTATTCsATGAATATCCGGTAuTTGAATGTuTCCGGsGAuCAGGCsGAsAGTTTGGTusGCAGuT

                      cov     pid 7601 .         .         .         .         .         .         .         .         . 7680
GA2a.Bcac.CL03T12C61 100.0% 100.0%  tgtctttcatctgtttttcctgcgacggcccgtccagcttcgttcccaagaccagatgcacttctttggtgcgtatcagc
GA2b.Bfra.CL05T12C13  99.7%  81.0%  tgtcttttatctgtttttcctgcgaaggttcatccagtttcgttccaagaatcagatggacttctttggttcggatcagt
GA2c.Bvul.TF05-18     99.8%  82.0%  tgtcttttatctgtttttcctgcgaaggctcatccagtttcgttccaagaatcagatggacctctttggtccgcaccagc
GA2d.Buni.BIOML-A83   99.2%  69.8%  tgtctttcatccgtttttcctgtaatggttcatccaatcttgttcccaaaatcagatgaacatcctttgtacgtatcaac
GA2e.Bsal.DSM.18170   99.7%  79.1%  tgtctttcatctgcttctcctgtgacgggtcgtccagctttcttgagaatcaagtgtacctctttggtccgtatcagt
consensus/100%                      TGTCTTTsATCsGsTTsTCCTGsuAsGGssCuTCCAussTsssTsssAuuAsCAuuTGsACsTCsTTsGTsCGsAsCAus
consensus/90%                       TGTCTTTsATCsGsTTsTCCTGsuAsGGssCuTCCAussTsssTsssAuuAsCAuuTGsACsTCsTTsGTsCGsAsCAus
consensus/80%                       TGTCTTTsATCTGTTTTTCCTGsGAsGGsTCuTCCAGsTTsGTTCCsAuAATCAGATGsACsTCTTTGGTsCGsATCAGs
consensus/70%                       TGTCTTTsATCTGTTTTTCCTGsGAsGGsTCuTCCAGsTTsGTTCCsAuAATCAGATGsACsTCTTTGGTsCGsATCAGs
```

```
                              cov    pid 7681 .         .         .         .         .         .         .         .         . 7760
GA2a.Bcac.CL03T12C61 100.0% 100.0%   ccttcggtgggaactgggctgatttccaccccgttgcggatttctgtccgcaacagaagctcgccaagctctttcttgca
GA2b.Bfra.CL05T12C13  99.7%  81.0%   ccttcgtgcggcatcgggctgatttccaccccgttccggatttctgtacgcaccagcagttcgccgagttcttttttgca
GA2c.Bvul.TF05-18     99.8%  82.0%   ccttcatggggcatcgggctgatctccactccgttccggatttctgtccgcaccaatagctcaccgagttccttcttgca
GA2d.Buni.BIOML-A83   99.2%  69.8%   ccctcatgaggcatcgaactgattgccaccccattacgaatatcagtacgtaataatagttctcccagttctttcttaca
GA2e.Bsal.DSM.18170   99.7%  79.1%   ccttcactgggcatcgggctgatttccactccgttcttaatttctgtacgtaccagcagctctccgagttcttttttaca
consensus/100%                       CCsTCusssGGsAssGuuCTGATssCCACsCCuTTsssuATsTCsGTsCGsAssAusAGsTCsCCsAGsTCsTTsTTuCA
consensus/90%                        CCsTCusssGGsAssGuuCTGATssCCACsCCuTTsssuATsTCsGTsCGsAssAusAGsTCsCCsAGsTCsTTsTTuCA
consensus/80%                        CCTTCussuGGCATCGGGCTGATTTCCACsCCGTTsCGuATTTCTGTsCGsAsCAusAGsTCsCCuAGTTCTTTsTTuCA
consensus/70%                        CCTTCussuGGCATCGGGCTGATTTCCACsCCGTTsCGuATTTCTGTsCGsAsCAusAGsTCsCCuAGTTCTTTsTTuCA

                              cov    pid 7761 .         .         .         .         .         .         .         .         . 7840
GA2a.Bcac.CL03T12C61 100.0% 100.0%   gaagctggctatgtcgttttgcgtcagcacacgcccgtgtgaaataatcccgtatttgtagatatcgatcagctcccggt
GA2b.Bfra.CL05T12C13  99.7%  81.0%   gaaactggctatgtcgttctgtgtcaggacacggccgtgcgaaatgaaggcgtatttgtagatgtcaatccgttctctat
GA2c.Bvul.TF05-18     99.8%  82.0%   gaaactggctatgtcgttttgtgtcaggattcggccgtgcgagatgaaaccgtatttgtagatgtcgatctgttccctgt
GA2d.Buni.BIOML-A83   99.2%  69.8%   gaaactggctatatcattctgagtaagaacacgtccgtgagaaagaaaaccatatttgtaatgttctatttgttccatgt
GA2e.Bsal.DSM.18170   99.7%  79.1%   gaaactgataatatcattctgggtcaatacacggccatgtgaaatgaaaccgtatttgtaaatgtctatgcgttcccggt
consensus/100%                       GAAuCTGussATuTCuTTsTGsGTsAusAssCGsCCuTGsGAuAsuAsssCuTATTTGTAusssTCsATssGsTCsssuT
consensus/90%                        GAAuCTGussATuTCuTTsTGsGTsAusAssCGsCCuTGsGAuAsuAsssCuTATTTGTAusssTCsATssGsTCsssuT
consensus/80%                        GAAACTGGCTATuTCuTTsTGsGTCAGsACACGsCCGTGsGAAATuAAuCCGTATTTGTAuATuTCsATssGTTCCCsGT
consensus/70%                        GAAACTGGCTATuTCuTTsTGsGTCAGsACACGsCCGTGsGAAATuAAuCCGTATTTGTAuATuTCsATssGTTCCCsGT

                              cov    pid 7841 .         .         .         .         .         .         .         .         .7920
GA2a.Bcac.CL03T12C61 100.0% 100.0%   tcttgggggcttgcttcccgccgtagatggtactgacaagcgcgagtgtcttcgggtccagataggtggtggcgtatggc
GA2b.Bfra.CL05T12C13  99.7%  81.0%   tttggggggcttgtttcccgccgtaggtggtactgaccagagccagtgtcttcgggtccaggtaagtggtggcgttgggg
GA2c.Bvul.TF05-18     99.8%  82.0%   ttttgggggcttgttttccgccgtaggtggtgctgaccagtgcgagtgtcttcgagtccagataagtggtggcattgggt
GA2d.Buni.BIOML-A83   99.2%  69.8%   ttttaggagagtgcttgcctccataagtcgtactgacaagtgccaatgtttttggattcacataggtattggtattaggg
GA2e.Bsal.DSM.18170   99.7%  79.1%   tcttgggagcctgtttgccgccataggtggtgctgacaagcgcaagagtccttggctccagataggtgttggtattgggc
consensus/100%                       TsTsuGGuGssTGsTTsCCsCCuTAuuTsGTuCTGACsAGsGCsAusGTssTsGusTsCAsuTAuGTusTGGsuTssGGs
consensus/90%                        TsTsuGGuGssTGsTTsCCsCCuTAuuTsGTuCTGACsAGsGCsAusGTssTsGusTsCAsuTAuGTusTGGsuTssGGs
consensus/80%                        TsTTGGGuGCsTGsTTsCCGCCuTAGGTGGTuCTGACsAGsGCsAGTGTCTTsGGuTCCAGATAuGTGsTGGsuTTuGGs
consensus/70%                        TsTTGGGuGCsTGsTTsCCGCCuTAGGTGGTuCTGACsAGsGCsAGTGTCTTsGGuTCCAGATAuGTGsTGGsuTTuGGs

                              cov    pid 7921 .         .         .         .         .         .         .         .         . 8000
GA2a.Bcac.CL03T12C61 100.0% 100.0%   gacagctccacgccggcctgtatttggttgcctgtctcgcagtgggtggtccagtatttcacggtgacctgccctttgcc
GA2b.Bfra.CL05T12C13  99.7%  81.0%   gacaggtttacgcctgcctgtatcaggtttcctacctcacagttggttgtccagtatttgacggtaatctgccctttgcc
GA2c.Bvul.TF05-18     99.8%  82.0%   gacaggttcacaccggcctgtatccggttgcccacctcgcagttggtggtccagtatttgacggtaatctgtcccttgcc
GA2d.Buni.BIOML-A83   99.2%  69.8%   gacaactcgactccggcttgtatctggttgccaatctcgcaattcgtagtccagtatttcaccgttatctgtccgttgcc
GA2e.Bsal.DSM.18170   99.7%  79.1%   gacagatccacgccggcttgtatctggtttcctatctcacagttggtggtccagtatttgacggtaacctggcctttacc
consensus/100%                       GACAusTssACsCCsGCsTGTATssGGTTsCCsusCTCuCAuTssGTsGTCCAGTATTTsACsGTsAsCTGsCCsTTuCC
consensus/90%                        GACAusTssACsCCsGCsTGTATssGGTTsCCsusCTCuCAuTssGTsGTCCAGTATTTsACsGTsAsCTGsCCsTTuCC
consensus/80%                        GACAGsTssACuCCGGCsTGTATCsGGTTsCCsAsCTCuCAGTTGGTuGTCCAGTATTTsACGGTuAsCTGsCCsTTGCC
consensus/70%                        GACAGsTssACuCCGGCsTGTATCsGGTTsCCsAsCTCuCAGTTGGTuGTCCAGTATTTsACGGTuAsCTGsCCsTTGCC
```

```
                             cov    pid 8001 .       .         .         .         .         .         .         .         . 8080
GA2a.Bcac.CL03T12C61 100.0% 100.0%  ggacatttggtcgatcatcaggtaatagggtgtctccctgtattcgttcatgttgtcggttgcctgtttcagctgcgccg
GA2b.Bfra.CL05T12C13  99.7%  81.0%  ggacagttcgtcaatcataaggtaataaggtgtttccctgaactcgttcatgttgtctgctgtctgtttcatttgcgcca
GA2c.Bvul.TF05-18     99.8%  82.0%  ggacaactggtcgatcatcagataatagggagtctccctggactcgttcatgctgtcggctgtctgcttcatttgtgcca
GA2d.Buni.BIOML-A83   99.2%  69.8%  attcatacggtctatcattaaataataaggtgtttcccttgtttcattcatttgctctgttatctgcttcatttgctcca
GA2e.Bsal.DSM.18170   99.7%  79.1%  ggtcaattggtcgatcatcaggtagtagggcgtctctctgaactcattcatgttatcggctgtttgtttcatttgtgcca
consensus/100%                      ussCAssssGTCsATCATsAuuTAuTAuGGsGTsTCsCTssssTCuTTCATssssTCsGsTussTGsTTCAssTGssCCu
consensus/90%                       ussCAssssGTCsATCATsAuuTAuTAuGGsGTsTCsCTssssTCuTTCATssssTCsGsTussTGsTTCAssTGssCCu
consensus/80%                       GGsCAssTGGTCuATCATsAGuTAATAuGGsGTsTCCCTGuAsTCuTTCATGTTuTCsGsTGTCTGsTTCATTTGsGCCA
consensus/70%                       GGsCAssTGGTCuATCATsAGuTAATAuGGsGTsTCCCTGuAsTCuTTCATGTTuTCsGsTGTCTGsTTCATTTGsGCCA

                             cov    pid 8081 .       .         .         .         .         .         .         .         . 8160
GA2a.Bcac.CL03T12C61 100.0% 100.0%  tcagttgctccatctggccggcgagcgcttgcagcttgacactcgaaacggcatggaaggcgtgggtctcgtcatccagc
GA2b.Bfra.CL05T12C13  99.7%  81.0%  tcagctgctccatctggatggtaagtgcctgtagcttgatgcttgatacggcattgaaagcatgggtctcgtcatccagt
GA2c.Bvul.TF05-18     99.8%  82.0%  tcagttgctccatctggtggtaagtgcctgtagcttgatgcttgaaacggcatggaaggcatgggtctcgtcatcgagc
GA2d.Buni.BIOML-A83   99.2%  69.8%  tcaattgctccatctgaccaaccaacgcttgtagtttcgcactggaaacagcagaaaaagcatgtgattcgtcatcaagc
GA2e.Bsal.DSM.18170   99.7%  79.1%  ccagttgctccatttgggtggcaagtgcctgtagcttgatgctcgaaacggaaccgaatgcatgggtctcatcatccagc
consensus/100%                      sCAusTGCTCCATsTGussuussAusGCsTGsAGsTTsusuCTsGAsACuGsAssuAAsGCuTGsGssTCuTCATCsAGs
consensus/90%                       sCAusTGCTCCATsTGussuussAusGCsTGsAGsTTsusuCTsGAsACuGsAssuAAsGCuTGsGssTCuTCATCsAGs
consensus/80%                       TCAGTTGCTCCATCTGGssGGsuAGsGCsTGTAGCTTGAsuCTsGAAACGGCAssGAAuGCATGGGTCTCGTCATCsAGC
consensus/70%                       TCAGTTGCTCCATCTGGssGGsuAGsGCsTGTAGCTTGAsuCTsGAAACGGCAssGAAuGCATGGGTCTCGTCATCsAGC

                             cov    pid 8161 .       .         .         .         .         .         .         .         . 8240
GA2a.Bcac.CL03T12C61 100.0% 100.0%  aggtccagcagatagccgagcagttccttggcggagcgggagtcaaaacgctcgcagcctcccttgcggatggaataggt
GA2b.Bfra.CL05T12C13  99.7%  81.0%  aggtcggacagatagccgagcagctccttggcggaacgggagtcgaaacgctcgcatcctcctttgcggatggagtaggt
GA2c.Bvul.TF05-18     99.8%  82.0%  aggtctgacaggtagccgagcagttccttggcggaacgggaatcgaaacgttcgcatccgcctttgcggatggagtaggt
GA2d.Buni.BIOML-A83   99.2%  69.8%  aagtcaagcagatatacaagaagttcctttgccgaacgggagtcaaaacgttcacagccaccctttctcaaagaataagt
GA2e.Bsal.DSM.18170   99.7%  79.1%  aggtccagcagatagcatagcagttctttggcagaacgggagtcaaaccgttcgcaccctcctttgcggatggaatacgt
consensus/100%                      AuGTCsuuCAGuTAssssAGsAGsTCsTTsGCsGAuCGGGAuTCuAAsCGsTCuCAsCCsCCsTTsCssAsuGAuTAsGT
consensus/90%                       AuGTCsuuCAGuTAssssAGsAGsTCsTTsGCsGAuCGGGAuTCuAAsCGsTCuCAsCCsCCsTTsCssAsuGAuTAsGT
consensus/80%                       AGGTCsuuCAGATAGCCuAGCAGTTCCTTGGCuGAACGGGAGTCuAAACGsTCGCAsCCsCCsTTGCGGATGGAuTAuGT
consensus/70%                       AGGTCsuuCAGATAGCCuAGCAGTTCCTTGGCuGAACGGGAGTCuAAACGsTCGCAsCCsCCsTTGCGGATGGAuTAuGT

                             cov    pid 8241 .       .         .         .         .         .         .         .         . 8320
GA2a.Bcac.CL03T12C61 100.0% 100.0%  gccgtaactctccgtggaaccgggtaccggatactggagctcatgatagctctttccgtccgagtccttcacgctgtgca
GA2b.Bfra.CL05T12C13  99.7%  81.0%  tccatagctttccgtagaatcctttgcgggatagaggagttcatggtagttctttccgtcagagtccttcacgctgtgta
GA2c.Bvul.TF05-18     99.8%  82.0%  tccattgctttccgtagaatcctttcgggatagagaagttcatgataagtcttcccgtcggagtctttcacgctgtgta
GA2d.Buni.BIOML-A83   99.2%  69.8%  tccacaatcctccatactgctttcctgctggaacatcagctcgtaataattttaccattagagtccttcacgctatgca
GA2e.Bsal.DSM.18170   99.7%  79.1%  gccatagcccgccttggaatttccctccgggtacagaagctcatgatagctttcccgtccgagtctttcacactatgta
consensus/100%                      sCCussusssCCsTussusssssssssGusAssssAGsTCuTuuTAusTsTTsCCuTssGAGTCsTTCACuCTuTGsA
consensus/90%                       sCCussusssCCsTussusssssssssGusAssssAGsTCuTuuTAusTsTTsCCuTssGAGTCsTTCACuCTuTGsA
consensus/80%                       sCCATAuCssTCCuTuGAAsssssssCsGGuTAsAGuAGsTCATGATAusTsTTsCCGTCsGAGTCsTTCACGCTuTGsA
consensus/70%                       sCCATAuCssTCCuTuGAAsssssssCsGGuTAsAGuAGsTCATGATAusTsTTsCCGTCsGAGTCsTTCACGCTuTGsA
```

```
                       cov    pid 8321 .         .         .         .         .         .         .         .         . 8400
GA2a.Bcac.CL03T12C61 100.0% 100.0%    cggacagcagggactcgtaattgccggtacgcaggggaatcaccctgaacgtgtcttcgagggaggtggtctgttcgtgc
GA2b.Bfra.CL05T12C13  99.7%  81.0%    cggacagaagggattcgtaactgcccgtgcgcaagggtatcaccctgaaagtttcctcaagggtggtcgtctgttcgtga
GA2c.Bvul.TF05-18     99.8%  82.0%    cggacagcagggactcatggttgcctgtgcgtaatggcaccaccctgaaggtgtcctcaaggggagttgtttgttcatgc
GA2d.Buni.BIOML-A83   99.2%  69.8%    ctgccaataaagactcactttttcctgtacgcaatggaattaccctaaatgtatcttctatattcgtattttgttcatgg
GA2e.Bsal.DSM.18170   99.7%  79.1%    cggacaacagggattcatgattttccgtccgcaggggaatcaccctgaaagtgtcttcaagggtgctcacttgttcacgc
consensus/100%                        CsGsCAusAuuGAsTCussssTssCsGTsCGsAusGGsAssACCCTuAAsGTsTCsTCsAsussssTssssTGTTCusGs
consensus/90%                         CsGsCAusAuuGAsTCussssTssCsGTsCGsAusGGsAssACCCTuAAsGTsTCsTCsAsussssTssssTGTTCusGs
consensus/80%                         CGGACAusAGGGAsTCuTuuTTsCCsGTuCGCAusGGsATCACCCTGAAsGTuTCsTCuAGGGsuGTsuTsTGTTCuTGs
consensus/70%                         CGGACAusAGGGAsTCuTuuTTsCCsGTuCGCAusGGsATCACCCTGAAsGTuTCsTCuAGGGsuGTsuTsTGTTCuTGs

                       cov    pid 8401 .         .         .         .         .         .         .         .         . 8480
GA2a.Bcac.CL03T12C61 100.0% 100.0%    agcagcttgttttccacaggcacgatgttgatgcccgcatgaagctcctccaggatttccggagtgaaatgggcggggaa
GA2b.Bfra.CL05T12C13  99.7%  81.0%    agcgtcttgttctctacaggcacgatgttgatgccggcatgaagtccttcaaggacttccgctgtgaaatgggcggggaa
GA2c.Bvul.TF05-18     99.8%  82.0%    agtgtcttgttctcgacaggtacgatattgatgccggcatgcagttcttccaggacttccggcgtgaaatgggcgggaaa
GA2d.Buni.BIOML-A83   99.2%  69.8%    agtaacttgttttctacaggtacaacatttactccgacatgcaggtcttctaatatatctggtgtgaaatgtgctgggaa
GA2e.Bsal.DSM.18170   99.7%  79.1%    aacagtttgttttcgacgggcacgatattgattcccgcttgcaattcatccataacctcagctgtgaaatgggcgggaaa
consensus/100%                        AusussTTGTTsTCsACuGGsACuAsuTTsAssCCsuCsTGsAussCsTCsAssAssTCsGssGTGAAATGsGCsGGuAA
consensus/90%                         AusussTTGTTsTCsACuGGsACuAsuTTsAssCCsuCsTGsAussCsTCsAssAssTCsGssGTGAAATGsGCsGGuAA
consensus/80%                         AGsusCTTGTTsTCsACAGGsACGATuTTGATsCCsGCATGsAGsTCsTCsAuuAssTCsGssGTGAAATGGGCGGGuAA
consensus/70%                         AGsusCTTGTTsTCsACAGGsACGATuTTGATsCCsGCATGsAGsTCsTCsAuuAssTCsGssGTGAAATGGGCGGGuAA

                       cov    pid 8481 .         .         .         .         .         .         .         .         . 8560
GA2a.Bcac.CL03T12C61 100.0% 100.0%    tacgattttcacccacaccagcttctcttgcatcttttcccgcacacgctccccgaaacaggaagacaaggctttcggca
GA2b.Bfra.CL05T12C13  99.7%  81.0%    ctgtatcttcacccataccagtctgtcctgcatcttctccagaacggcctctttgaaacagggacgcagggtgtccggaa
GA2c.Bvul.TF05-18     99.8%  82.0%    ctgtatcttcacccagaccagcttgtcctgcattttctccaggacggcttctttgaaacaggagcgcaggatgtccggaa
GA2d.Buni.BIOML-A83   99.2%  69.8%    actcacctttacccatatcagcctgtctttcattttatccacgacagactccttgaaataatctgtgagctcttcaggaa
GA2e.Bsal.DSM.18170   99.7%  79.1%    ttgtatttttagccagaccagcctgtcctgcatcttttcccgcacggcttccttgaaacaaggatgcagttcttcaggca
consensus/100%                        ssssAssTTsAsCCAsAsCAGssTsTCsTsCATsTTsTCCsssACusssTCsssGAAAsAussssssAussssTssGGsA
consensus/90%                         ssssAssTTsAsCCAsAsCAGssTsTCsTsCATsTTsTCCsssACusssTCsssGAAAsAussssssAussssTssGGsA
consensus/80%                         ssssATsTTsACCCAsACCAGCsTGTCsTGCATsTTsTCCsGsACuGssTCsTTGAAACAuGuusuCAGssssTCsGGsA
consensus/70%                         ssssATsTTsACCCAsACCAGCsTGTCsTGCATsTTsTCCsGsACuGssTCsTTGAAACAuGuusuCAGssssTCsGGsA

                       cov    pid 8561 .         .         .         .         .         .         .         .         . 8640
GA2a.Bcac.CL03T12C61 100.0% 100.0%    ggggctccttgtcaacctcatcgagggggaccggttgcgaaacccgcaggaaatgcttgttgtagagttccatcacctcc
GA2b.Bfra.CL05T12C13  99.7%  81.0%    ggtttttcctgcaggaaccgtcgagtggaaaggactgcgataccttcagaaaatgtttgctgtaaatatccatgacttcc
GA2c.Bvul.TF05-18     99.8%  82.0%    ggtttttcttccaggaactgtcaaatggaaaggattgcgataccttcaagaaatgtttgctgtaaatatccatgacttcc
GA2d.Buni.BIOML-A83   99.2%  69.8%    atgagaccttatccttatcatctataacaagctgctgggaaatggtaagataatgcttgttgtatatatccatcacttct
GA2e.Bsal.DSM.18170   99.7%  79.1%    atgtctctttgcaggaatcatcaagcgggaaagactgccggacgctcaggaaatgtttattgtagatttccatgatttcc
consensus/100%                        ussssssssTsssssssssuTCsAssusuAsssusTGssusAsssssAuusAATGsTTusTGTAsAssTCCATsAssTCs
consensus/90%                         ussssssssTsssssssssuTCsAssusuAsssusTGssusAsssssAuusAATGsTTusTGTAsAssTCCATsAssTCs
consensus/80%                         ussssTsCTTussuusAsCuTCuAusGGuAusGusTGCGAsACssTCAGuAAATGsTTGsTGTAuATsTCCATsACTTCC
consensus/70%                         ussssTsCTTussuusAsCuTCuAusGGuAusGusTGCGAsACssTCAGuAAATGsTTGsTGTAuATsTCCATsACTTCC
```

```
                              cov     pid 8641 .         .         .         .         .         .         .         .         . 8720
GA2a.Bcac.CL03T12C61 100.0% 100.0%      ttgtctatgacggacatcacatcatagttccggaagaaggcacgggtgtattcctccggcaaccggc-ttttctcataga
GA2b.Bfra.CL05T12C13  99.7%  81.0%      ttgtctatgacggacatcacgtcatagtcttggaaaaaggcacgggttggttctttctgtatggttg-tcttttcatgga
GA2c.Bvul.TF05-18     99.8%  82.0%      ctgtctatgacggacatcacatcgtagttctggaaaaaggcacgggtcggctcttccggtatggttg-tcctttcatgga
GA2d.Buni.BIOML-A83   99.2%  69.8%      ttatctatcataaccattatatcataattctggaaaaaactataagcagagtgtctggatggtatcattttctgataaa
GA2e.Bsal.DSM.18170   99.7%  79.1%      ttgtctatgaggaacatcgcatcatagtttggaaaaatgcacggaagggattatcttgtatatcgt-cctttcgtaga
consensus/100%                          sTuTCTATsAsuusCATsusuTCuTAuTsssGGAAuAAsuCsssuussusssTsssGssssssss.sssTsTsuTuuA
consensus/90%                           sTuTCTATsAsuusCATsusuTCuTAuTsssGGAAuAAsuCsssuussusssTsssGssssssss.sssTsTsuTuuA
consensus/80%                           TTGTCTATGAsGuACATCACATCATAGTTsTGGAAAAAuGCACGGussuGsTssTCssGsAsussss.TsTTsTCATuGA
consensus/70%                           TTGTCTATGAsGuACATCACATCATAGTTsTGGAAAAAuGCACGGussuGsTssTCssGsAsussss.TsTTsTCATuGA
```

```
                              cov     pid 8721 .         .         .         .         .         .         .         .         . 8800
GA2a.Bcac.CL03T12C61 100.0% 100.0%      tgcccggctccatccggatggggggttccaccgacggaccattccgtgcacggaagcaggaacaggtattcatagctttcg
GA2b.Bfra.CL05T12C13  99.7%  81.0%      ttccaccttccatcacgactgtcttcccttctacggaccattccgtacaggggagcaggagcagatattcatagctttcc
GA2c.Bvul.TF05-18     99.8%  82.0%      tgcctcgttccatcacaaccggcttcccctctacggaccattccgtacaggggagcaggagcagatattcatagctctcc
GA2d.Buni.BIOML-A83   99.2%  69.8%      tgcctttctgcatatcaatcctttttccatctatataccattctgtacaaggtagcaggaacagatattcatagctgtcc
GA2e.Bsal.DSM.18170   99.7%  79.1%      ttccccgctgcatgacaacctgttctcccttgacggaccattccgtacagggaagcaggaacaggtattcatagctttcc
consensus/100%                          TsCCssssTsCATsssuAssssssssCCssssAsusACCATTCsGTuCAsGGsAGCAGGAuCAGuTATTCATAGCTsTCs
consensus/90%                           TsCCssssTsCATsssuAssssssssCCssssAsusACCATTCsGTuCAsGGsAGCAGGAuCAGuTATTCATAGCTsTCs
consensus/80%                           TsCCssssTsCATssCuAsssssTTsCCsTCsACGGACCATTCCGTACAuGGuAGCAGGAuCAGuTATTCATAGCTsTCC
consensus/70%                           TsCCssssTsCATssCuAsssssTTsCCsTCsACGGACCATTCCGTACAuGGuAGCAGGAuCAGuTATTCATAGCTsTCC
```

```
                              cov     pid 8801 .         .         .         .         .         .         .         .         . 8880
GA2a.Bcac.CL03T12C61 100.0% 100.0%      gtaaggttggggaagtcaagatagaaagaaatgttttccaggtccgtcagcgagtcgtccaccgccaggccgagccacac
GA2b.Bfra.CL05T12C13  99.7%  81.0%      gtaaggttgggaaaatccagatagaacgaaaagtcttcaaggtcggaaatggattcatccaccgccattcctaaccagac
GA2c.Bvul.TF05-18     99.8%  82.0%      atgaggtttgggaaatccagatagaaggaaaagtcctcaaggtcggaaatggattcatccaccgccacccctaaccagac
GA2d.Buni.BIOML-A83   99.2%  69.8%      gttaaattcgggaagtcaagataaaatgacagattttccagtccgttgatggcttcatcaacagtcaacccaatccacac
GA2e.Bsal.DSM.18170   99.7%  79.1%      gtaagattgggaaaatcaagatagaaagagaattccgaaggttggtaatggattcgtccacagccaggccgagccaaac
consensus/100%                          uTsAuuTTsGGuAAuTCsAGATAuAAsGAsAsuTsssssAGsssssssAssGssTCuTCsACsGsCAssCCsAsCCAsAC
consensus/90%                           uTsAuuTTsGGuAAuTCsAGATAuAAsGAsAsuTsssssAGsssssssAssGssTCuTCsACsGsCAssCCsAsCCAsAC
consensus/80%                           GTuAGuTTsGGuAAuTCsAGATAGAAsGAuAuuTssTCsAGGTCGGsuATGGATTCuTCCACsGCCAssCCsAuCCAsAC
consensus/70%                           GTuAGuTTsGGuAAuTCsAGATAGAAsGAuAuuTssTCsAGGTCGGsuATGGATTCuTCCACsGCCAssCCsAuCCAsAC
```

```
                              cov     pid 8881 .         .         .         .         .         .         .         .         . 8960
GA2a.Bcac.CL03T12C61 100.0% 100.0%      cgtacgtgcgggcgtccttgtttccgtacgggcaatcatggttttcgacagggtatggtccacctgatagagaatgtcgt
GA2b.Bfra.CL05T12C13  99.7%  81.0%      ggtacgcggagacatccttgtctctgcacgggcaatcatggtcttctccaaggtagggctatccggtagagaaggtcgt
GA2c.Bvul.TF05-18     99.8%  82.0%      ggtacgtggaggcatccttgtttccgtacgggcaatcatggtctttccaaggttgggctatccggtagagaaggtcgt
GA2d.Buni.BIOML-A83   99.2%  69.8%      ggagtctgacggcatttaacctcgctacgggctaccattgtcttctccaaagtatgactgacttcataaagcaggtcat
GA2e.Bsal.DSM.18170   99.7%  79.1%      ggtacgcggagtcatttggattccgtacgggcaatcatggttttctccagggtatggtctatccggtacaggagactat
consensus/100%                          sGsusssGssGsCuTssTsussTCsssACGGGCsAsCATsGTsTTsssCAuuGTssGusssAssssuTAsAGsAsussuT
consensus/90%                           sGsusssGssGsCuTssTsussTCsssACGGGCsAsCATsGTsTTsssCAuuGTssGusssAssssuTAsAGsAsussuT
consensus/80%                           GGTACGsGuuGuCATssTsGssTCsGTACGGGCAATCATGGTsTTCTCCAuGGTAsGGsCsAsCsGuTAuAGuAGGTCuT
consensus/70%                           GGTACGsGuuGuCATssTsGssTCsGTACGGGCAATCATGGTsTTCTCCAuGGTAsGGsCsAsCsGuTAuAGuAGGTCuT
```

```
                        cov    pid 8961 .         .         .         .         .         .         .         .         . 9040
GA2a.Bcac.CL03T12C61 100.0% 100.0% cacacaccatgtatttcacatcggctttgtgcagcagggtgtcacaggccggataaaaggaaatgcttccggcggacagc
GA2b.Bfra.CL05T12C13  99.7%  81.0% cgcagaccatatatttcacgtcggctttgtgcagcagggtatcgcaggcgggataaaacgatacgcttccggtcgatagc
GA2c.Bvul.TF05-18     99.8%  82.0% cgcagaccatatatttcacatcggctttgtgcagaagggtatcgcaggcaggataaaacgacacgcttccggtcgatagt
GA2d.Buni.BIOML-A83   99.2%  69.8% cggatattatatacttgacttcaaccttgcgcaatatcatatcacaggccggataaaatgaaagcgtgcctgtggaaagt
GA2e.Bsal.DSM.18170   99.7%  79.1% tgcagaccatatatcttatgtcggctctatgtagcagtgtatcacatgccggataaaacgaaatacttcctgtcgacagc
consensus/100%                     susAsAssATuTAssTsAssTCuuCssTusGsAusAssuTuTCuCAsGCsGGATAAAAsGAsAsssTsCCsGssGAsAGs
consensus/90%                      susAsAssATuTAssTsAssTCuuCssTusGsAusAssuTuTCuCAsGCsGGATAAAAsGAsAsssTsCCsGssGAsAGs
consensus/80%                      CGCAsACCATATATTTsACuTCGGCTTTGTGCAGsAGsGTATCuCAGGCsGGATAAAAsGAsAsuCTTCCsGTsGAsAGs
consensus/70%                      CGCAsACCATATATTTsACuTCGGCTTTGTGCAGsAGsGTATCuCAGGCsGGATAAAAsGAsAsuCTTCCsGTsGAsAGs

                        cov    pid 9041.         .         .         .         .         .         .         .         . 9120
GA2a.Bcac.CL03T12C61 100.0% 100.0% ttctgggtcagcaggtcattctcataatacatgcccgactctttcgttatcaggtagccgggctcgatgggagaggcatg
GA2b.Bfra.CL05T12C13  99.7%  81.0% ttccgggtaagcgagtcgctctcgtaatacatgcccgattcccgggttatcaggtaacacggttctatgggataggcgtg
GA2c.Bvul.TF05-18     99.8%  82.0% ttccgggtaagggaatcgctctcgtaatacatgcctgattccctggttatctgatagcatggctctatgggataggcgtg
GA2d.Buni.BIOML-A83   99.2%  69.8% tttctgttcaacacatcactttcataatacataccagattcacgtgttatcaagtattcaggatcaataggggaagcatg
GA2e.Bsal.DSM.18170   99.7%  79.1% ttccggatcagtgagtcgctttcataatacatgcccgattcccgggtgatcagataaacgggttcaatgggaaaagcgtg
consensus/100%                     TTsssGsTsAususuTCusTsTCuTAATACATuCCsGAsTCssssGTsATCsuuTAssssGGsTCsATuGGusAuGCuTG
consensus/90%                      TTsssGsTsAususuTCusTsTCuTAATACATuCCsGAsTCssssGTsATCsuuTAssssGGsTCsATuGGusAuGCuTG
consensus/80%                      TTCCGGuTsAGsuuuTCuCTsTCuTAATACATGCCsGATTCsCssGTTATCAGuTAusssGGsTCsATGGGAsAuGCuTG
consensus/70%                      TTCCGGuTsAGsuuuTCuCTsTCuTAATACATGCCsGATTCsCssGTTATCAGuTAusssGGsTCsATGGGAsAuGCuTG

                        cov    pid 9121 .         .         .         .         .         .         .         .         . 9200
GA2a.Bcac.CL03T12C61 100.0% 100.0% cacgacaccatgcgcgggaagtgccgaggtcagtatgtccggtgtcagaatgcgggcggttttttccagcagccgcgtct
GA2b.Bfra.CL05T12C13  99.7%  81.0% tacgatgccgtgtgcgggaaagggtgaggtaaggatgtccggagtaaggattcgggcggtcttttccagcagccttgtct
GA2c.Bvul.TF05-18     99.8%  82.0% tacgatgccgtgtgcgggaaagggtgaggtcagtatgcccggagtaaggattcgggcggtcttttccagcagcctggtct
GA2d.Buni.BIOML-A83   99.2%  69.8% tacaatgccatgcgcagggaatggtgatgccagcatacctggtgtaagtatgtgggctgtcttttccaacagtctggttt
GA2e.Bsal.DSM.18170   99.7%  79.1% tacgatgccgtgtgcggggaagggggaggtcaggatacctggtgtcagaatccgggcggtcttttccagcagccttgttt
consensus/100%                     sACuAsuCCuTGsGCuGGuAusGssGAsGssAGsATusCsGGsGTsAGsATssGGGCsGTsTTTTCCAuCAGsCssGTsT
consensus/90%                      sACuAsuCCuTGsGCuGGuAusGssGAsGssAGsATusCsGGsGTsAGsATssGGGCsGTsTTTTCCAuCAGsCssGTsT
consensus/80%                      TACGATGCCuTGsGCGGGuAAsGGsGAGGTCAGsATusCsGGsGTsAGuATsCGGGCGGTCTTTTCCAGCAGCCTsGTsT
consensus/70%                      TACGATGCCuTGsGCGGGuAAsGGsGAGGTCAGsATusCsGGsGTsAGuATsCGGGCGGTCTTTTCCAGCAGCCTsGTsT

                        cov    pid 9201.         .         .         .         .         .         .         .         . 9280
GA2a.Bcac.CL03T12C61 100.0% 100.0% cgatggccgtgaagtcttcccccagcatatacagttcgttggacagcgcctcaagcatcagtttgacgacagggtcaaaa
GA2b.Bfra.CL05T12C13  99.7%  81.0% cgatggcggtaaaatcctctcccagcatatatatctcgttggacaacgcttccagcaggagcttgacaaccgggtcgaag
GA2c.Bvul.TF05-18     99.8%  82.0% cgatggcggtaaaatcctctcccagcatatatacctcgttggacaaggcttccagcaggagcttgacaaccgggtcgaag
GA2d.Buni.BIOML-A83   99.2%  69.8% caattgctgtaaaatcctctccgagtgtgtaaacctcattcgccagagcctccaacataagtttgaccaccggatcgaaa
GA2e.Bsal.DSM.18170   99.7%  79.1% caatggcggtaaaatcttctcccagcatatacatttcattggccaatgcttccagcaggagcttgacgacgggatcaaaa
consensus/100%                     CuATsGCsGTuAAuTCsTCsCCsAGsuTuTAsAssTCuTTsGsCAusGCsTCsAuCAssAGsTTGACsACsGGuTCuAAu
consensus/90%                      CuATsGCsGTuAAuTCsTCsCCsAGsuTuTAsAssTCuTTsGsCAusGCsTCsAuCAssAGsTTGACsACsGGuTCuAAu
consensus/80%                      CuATGGCsGTAAAATCsTCTCCCAGCATATAsAssTCuTTGGsCAusGCsTCCAGCAsuAGsTTGACuACsGGuTCuAAu
consensus/70%                      CuATGGCsGTAAAATCsTCTCCCAGCATATAsAssTCuTTGGsCAusGCsTCCAGCAsuAGsTTGACuACsGGuTCuAAu
```

```
                       cov    pid 9281 .                                                                                9360
GA2a.Bcac.CL03T12C61 100.0% 100.0%   ttttccgctttcttgattccccagaacgccgccgcgtatttcagcatacggtttctgatctgctccttgttatatttctc
GA2b.Bfra.CL05T12C13  99.7%  81.0%   ttttccgccttttttattccccagaaagccgcagcgtatttcagcatacggtttctgatttgctctttattgtatttttc
GA2c.Bvul.TF05-18     99.8%  82.0%   ttctccgccttttttattccccagaaagccgcggcatatttcagcatacggtttctgatttgctctttattgtatttttc
GA2d.Buni.BIOML-A83   99.2%  69.8%   ttttccaccttctttataccccaaaatgctgctgcatacttcagcatccggttcctgatttgctctttattgtatttttc
GA2e.Bsal.DSM.18170   99.7%  79.1%   ttttcggcttttttgattccccagaaagtcgcagcatactttagcatacggttcctgatttgttccttgttatatttctc
consensus/100%                       TTsTCsuCsTTsTTsATsCCCCAuAAsGssGCsGCuTAsTTsAGCATsCGGTTsCTGATsTGsTCsTTuTTuTATTTsTC
consensus/90%                        TTsTCsuCsTTsTTsATsCCCCAuAAsGssGCsGCuTAsTTsAGCATsCGGTTsCTGATsTGsTCsTTuTTuTATTTsTC
consensus/80%                        TTTTCCGCsTTsTTsATTCCCCAGAAsGCCGCsGCuTAsTTCAGCATACGGTTsCTGATTTGCTCsTTuTTuTATTTsTC
consensus/70%                        TTTTCCGCsTTsTTsATTCCCCAGAAsGCCGCsGCuTAsTTCAGCATACGGTTsCTGATTTGCTCsTTuTTuTATTTsTC
```

```
                       cov    pid 9361 .                                                                                9440
GA2a.Bcac.CL03T12C61 100.0% 100.0%   catactcata-cgcttgtcatcgggttgaaatgggtcctaaataaatcacatagtaaaaacaacgcttctctccggtatg
GA2b.Bfra.CL05T12C13  99.7%  81.0%   tgtactcata-atcctttcatcgggttgaaatcggtcctaaatagattacataatagaaacatcgtttttctccggtatg
GA2c.Bvul.TF05-18     99.8%  82.0%   tgtactcata-cgcctcttatcgggttgaaatgggtcctaaataaatcacataatagaaacagcgtttttctccggtatg
GA2d.Buni.BIOML-A83   99.2%  69.8%   ttcactcata----ctgttatcgtgttgaaataggtcctaagtaaatcacataatagaatgtccttctttctcctgtacg
GA2e.Bsal.DSM.18170   99.7%  79.1%   tatgctcatataccttgttatcgggttgaaatgggacctaaataaatcacataataaaagcaccgcttctctcctgtgtg
consensus/100%                       sssuCTCATA....sTsTsATCGsGTTGAAATsGGsCCTAAuTAuATsACATAuTAuAAssssCsssTsTCTCCsGTusG
consensus/90%                        sssuCTCATA....sTsTsATCGsGTTGAAATsGGsCCTAAuTAuATsACATAuTAuAAssssCsssTsTCTCCsGTusG
consensus/80%                        TuTACTCATA.ssCsTsTsATCGGGTTGAAATuGGTCCTAAATAAATCACATAATAuAAuCAsCGsTTsTCTCCsGTATG
consensus/70%                        TuTACTCATA.ssCsTsTsATCGGGTTGAAATuGGTCCTAAATAAATCACATAATAuAAuCAsCGsTTsTCTCCsGTATG
```

```
                       cov    pid 9441 .                                                                                9520
GA2a.Bcac.CL03T12C61 100.0% 100.0%   caccagattgccatagacatatacctgcacgcgtttttttatggtcgaagcctcgaacacattgtcctgccgggtggtat
GA2b.Bfra.CL05T12C13  99.7%  81.0%   taccagggttccatatacatatacttgtacacgtttctttacggcgggagcgtcaaatacattgtcctgtcgggtggtgt
GA2c.Bvul.TF05-18     99.8%  82.0%   caccaaggttccatatacatatacctgtacacgtttctttacggcgggagcgtcgaacacattgtcctgccgggtggtgt
GA2d.Buni.BIOML-A83   99.2%  69.8%   aaccagatttccagacacataaacctgaactctcttctttatacttgatgtttcatacatactgtcctgcaacattgtgt
GA2e.Bsal.DSM.18170   99.7%  79.1%   taccagagtgccatacacatatacctgaacgcgtttttttatggtcgaagtgccggacacactgacctgtcgggtcgtgt
consensus/100%                       sACCAuusTsCCAsAsACATAsACsTGsACsCssTTsTTTAsusssGusGsssCusAsAsAsTGsCCTGssusuTsGTuT
consensus/90%                        sACCAuusTsCCAsAsACATAsACsTGsACsCssTTsTTTAsusssGusGsssCusAsAsAsTGsCCTGssusuTsGTuT
consensus/80%                        sACCAGusTsCCATAsACATATACCTGsACuCGTTTsTTTAsGGssGuAGssTCuuACACAsTGTCCTGsCGGGTsGTGT
consensus/70%                        sACCAGusTsCCATAsACATATACCTGsACuCGTTTsTTTAsGGssGuAGssTCuuACACAsTGTCCTGsCGGGTsGTGT
```

```
                       cov    pid 9521 .                                                                                9600
GA2a.Bcac.CL03T12C61 100.0% 100.0%   cgtcgataatgacccggacgttcacattcgtaagacgcttctcgtaccgggtgatggcttgggtgatatagtcggaaaac
GA2b.Bfra.CL05T12C13  99.7%  81.0%   catcaatgatgacacgtacatttacacctgtaagccgcttctcgtgccgggtaattgccttggttatgtattccgagaat
GA2c.Bvul.TF05-18     99.8%  82.0%   catcaatgatgacccggacattcacacccgtaagccgcttctcatgctgggtgatcgccttggtgatgtattccgagaat
GA2d.Buni.BIOML-A83   99.2%  69.8%   cgtctataattacacgcacactgatattggtcagtctcttttcatatgtggtaacagctgtagtgatatattcggagaac
GA2e.Bsal.DSM.18170   99.7%  79.1%   catctatggcgacccgtacatatacgttcataagacgctttttcgtatcgggtgattgccttcgtaatgtattctgagaat
consensus/100%                       CuTCsATuussACsCGsACussssAsusssuTsAGsCsCTTsTCuTusssGGTuAssGCssssGTsATuTAsTCsGAuAAs
consensus/90%                        CuTCsATuussACsCGsACussssAsusssuTsAGsCsCTTsTCuTusssGGTuAssGCssssGTsATuTAsTCsGAuAAs
consensus/80%                        CuTCsATuATGACsCGsACATTsACAsssGTAAGsCGCTTsTCuTussGGGTuATsGCsTTuGTuATuTATTCsGAGAAs
consensus/70%                        CuTCsATuATGACsCGsACATTsACAsssGTAAGsCGCTTsTCuTussGGGTuATsGCsTTuGTuATuTATTCsGAGAAs
```

```
                        cov     pid 9601 .         .         .         .         .         .         .         .         . 9680
GA2a.Bcac.CL03T12C61 100.0% 100.0%  tgcccttcccagcgggtatgcgagatgatactctcaaaatcaagataaaaaatctcgcagccgaactccttgtcaaaggc
GA2b.Bfra.CL05T12C13  99.7%  81.0%  tgtccttcccaacgggtgtgtgaaacaatgctttcaaagtcgagaaaaagatctcacagccgaactccttgtcaaaggc
GA2c.Bvul.TF05-18     99.8%  82.0%  tgcccttcccaacgggtgtgtgaaacgatgctctcaaagtcgaggaaaagatctcacagccgaactccttgtcaaaggc
GA2d.Buni.BIOML-A83   99.2%  69.8%  tgtccttcccaacgtgtatgtgacacaatactttcgaaatcaagataaaaaatcttacagccaaacttcttgtcaaaggt
GA2e.Bsal.DSM.18170   99.7%  79.1%  tgcccttcccaacgggtatggagacaatgctttcaaaatcaagataaaatatctcacagccgaactccttgtcaaaggt
consensus/100%                      TGsCCTTCCCAuCGsGTuTGsGAsAsuATuCTsTCuAAuTCuAGusAAAAsATCTsuCAGCCuAACTsCTTGTCAAAGGs
consensus/90%                       TGsCCTTCCCAuCGsGTuTGsGAsAsuATuCTsTCuAAuTCuAGusAAAAsATCTsuCAGCCuAACTsCTTGTCAAAGGs
consensus/80%                       TGsCCTTCCCAACGGGTuTGsGAuACuATuCTsTCAAAuTCuAGAsAAAAuATCTCACAGCCGAACTCCTTGTCAAAGGs
consensus/70%                       TGsCCTTCCCAACGGGTuTGsGAuACuATuCTsTCAAAuTCuAGAsAAAAuATCTCACAGCCGAACTCCTTGTCAAAGGs

                        cov     pid 9681 .         .         .         .         .         .         .         .         . 9760
GA2a.Bcac.CL03T12C61 100.0% 100.0%  gtgctccccgggagccgtgtcaatcagcatctcgatgaactggtcgatggactctatctccgtacaggacgcgaggcggc
GA2b.Bfra.CL05T12C13  99.7%  81.0%  atgttcgccgggagccgtactgatcagcagttcgataaactggtcgatggactcgatttccgagcaggatgcaagacggc
GA2c.Bvul.TF05-18     99.8%  82.0%  atgttcgccgggagccgtgctgataagcagctcgataaactggtcgatggactcgatttccgaacaggatacgagacggc
GA2d.Buni.BIOML-A83   99.2%  69.8%  atgctcacccggagctgtactaatcaacagctcaataaactgatcaatggactctatttccgagcaggactccagacggc
GA2e.Bsal.DSM.18170   99.7%  79.1%  atgctctccgggagccgtactgatcagcaactcgatgaactggtcaatcgaatcaatctcgttgcacgaggcaagatggt
consensus/100%                      uTGsTCsCCsGGAGCsGTussuATsAuCAssTCuATuAACTGuTCuATsGAsTCsATsTCsssuCAsGAssCsAGusGGs
consensus/90%                       uTGsTCsCCsGGAGCsGTussuATsAuCAssTCuATuAACTGuTCuATsGAsTCsATsTCsssuCAsGAssCsAGusGGs
consensus/80%                       ATGsTCsCCGGGAGCCGTuCTuATCAGCAuCTCGATuAACTGGTCuATGGACTCsATsTCCGsuCAGGAsuCuAGACGGC
consensus/70%                       ATGsTCsCCGGGAGCCGTuCTuATCAGCAuCTCGATuAACTGGTCuATGGACTCsATsTCCGsuCAGGAsuCuAGACGGC

                        cov     pid 9761 .         .         .         .         .         .         .         .         . 9840
GA2a.Bcac.CL03T12C61 100.0% 100.0%  tgttttccacatcctcgctgagcaaggctccaaaatccagaggcagtttgcaaaatttcgtttccatacttgtcagcgcc
GA2b.Bfra.CL05T12C13  99.7%  81.0%  tgttttccacatcctcgctgagcagggcttcaaaatccagggcaacttgcaaaactttgtttccatacttgtcagtgcc
GA2c.Bvul.TF05-18     99.8%  82.0%  tgttttccacatcctcgctgagcaaggcaccaaaatccagggcaatttgcaaaactttgtttccatgcttgtcagtgcc
GA2d.Buni.BIOML-A83   99.2%  69.8%  tattctcgacatcttcgctcaacaatgctccaaagtctataggtaatttacagaatttgtttccatatttactaacgcc
GA2e.Bsal.DSM.18170   99.7%  79.1%  tgtttcccacatcttcgttcagcagggcctcaaagtccaaaggcagtttgcaaaatttcgtttccatatccgtcagcacc
consensus/100%                      TuTTssCsACATCsTCGsTsAuCAusGCssCAAAuTCsAsuGGsAusTTuCAuAAsTTsGTTTCCATusssussAusuCC
consensus/90%                       TuTTssCsACATCsTCGsTsAuCAusGCssCAAAuTCsAsuGGsAusTTuCAuAAsTTsGTTTCCATusssussAusuCC
consensus/80%                       TGTTTTCCACATCsTCGCTsAGCAuGGCssCAAAuTCCAuuGGCAuTTTGCAAAAsTTsGTTTCCATAsTTGTCAGsGCC
consensus/70%                       TGTTTTCCACATCsTCGCTsAGCAuGGCssCAAAuTCCAuuGGCAuTTTGCAAAAsTTsGTTTCCATAsTTGTCAGsGCC

                        cov     pid 9841 .         .         .         .         .         .         .         .         . 9920
GA2a.Bcac.CL03T12C61 100.0% 100.0%  attcgttattctcaaattcgtcgtcactgaattgcaatacccttgcggcaaacaggagtgtgatgcagctttttggttc
GA2b.Bfra.CL05T12C13  99.7%  81.0%  attcgttt---tcaaattcttcgtctatgaacttcagtgttcttgcggcaaacagaaggacgatgctgctggaattggat
GA2c.Bvul.TF05-18     99.8%  82.0%  attcgttt---tcaaattcttcgttcgtaaatctcatcgtccttgccgcaaacagaaggacgatgctgccggaatgggat
GA2d.Buni.BIOML-A83   99.2%  69.8%  attgattt---tcaaactcatcattgataaacaccaacaatcgtgcagcaaatcttataagcatatattgcttatgattg
GA2e.Bsal.DSM.18170   99.7%  79.1%  attcgttt---tcaaattcctcgttcgtaaatttcagtgttctcgcggcaaacagcaggacgatgctgcactcatgggta
consensus/100%                      ATTsuTTs...TCAAAsTCsTCuTsssTuAAsssCAssussCssGCsGCAAAssssAssussATusssssssssTsusss
consensus/90%                       ATTsuTTs...TCAAAsTCsTCuTsssTuAAsssCAssussCssGCsGCAAAssssAssussATusssssssssTsusss
consensus/80%                       ATTCGTTT...TCAAATTCsTCGTssuTuAAsssCAusussCTTGCuGCAAACAGsAGuAsGATGCsGCssssATGGsss
consensus/70%                       ATTCGTTT...TCAAATTCsTCGTssuTuAAsssCAusussCTTGCuGCAAACAGsAGuAsGATGCsGCssssATGGsss
```

```
                        cov     pid 9921 .       .       .       .       .       .       .       .       . 10000
GA2a.Bcac.CL03T12C61 100.0% 100.0%  atacggtcaaccttacgctgcaaacggatgcagttcccttccacgaaacgcaggacgaatacggcttcctggtccggttt
GA2b.Bfra.CL05T12C13  99.7%  81.0%  atacggtcaaccttgcgttgcaggcggacgcagttcccttccacaaagcgcaaggcaaatgccgcctgttggtcaggttg
GA2c.Bvul.TF05-18     99.8%  82.0%  atacggtcaaccttgcgttgcaggcggatgcagttcccttccacaaagcgtaaggcaaatgctgcctgttggtcaggttg
GA2d.Buni.BIOML-A83   99.2%  69.8%  acattgtcaacctttcgttggaactgaatgcaattaccatctgtaaaatgagctacaaatgcagcctgttgattgggctg
GA2e.Bsal.DSM.18170   99.7%  79.1%  ctatggtctattcttcgccggaaacggatgcagttgccctccgtaaagcgcagggcgaatgcggcctgctggtcgggttc
consensus/100%                      ssAssGTCsAsssTsCGssGsAussGuAsGCAuTTsCCsTCsusuAAusGsussuCuAATuCsGCsTssTGuTssGGsTs
consensus/90%                       ssAssGTCsAsssTsCGssGsAussGuAsGCAuTTsCCsTCsusuAAusGsussuCuAATuCsGCsTssTGuTssGGsTs
consensus/80%                       ATAsGGTCAACCTTsCGsTGsAuuCGGATGCAGTTsCCsTCCusAAAuCGsAuGuCuAATGCsGCCTGsTGGTCuGGTTs
consensus/70%                       ATAsGGTCAACCTTsCGsTGsAuuCGGATGCAGTTsCCsTCCusAAAuCGsAuGuCuAATGCsGCCTGsTGGTCuGGTTs

                        cov     pid 10001.       .       .       .       .       .       .       .       . 10080
GA2a.Bcac.CL03T12C61 100.0% 100.0%  gtctttccggtagacccgcaggtcaccgtcctcgcatggttggctgtaggcaccccaccgcagcaggccgtcacccggca
GA2b.Bfra.CL05T12C13  99.7%  81.0%  atccttctgataaatccgtatttcgccgttctcacgggagtcgttgtatgcggcccatctcaacagctcgtcacccggca
GA2c.Bvul.TF05-18     99.8%  82.0%  gtccttttggtagattcgtatttcgccgtcctcacgggagtcgctgtatgcggcccatctcaacagttcgttacccggca
GA2d.Buni.BIOML-A83   99.2%  69.8%  gtctttctgatatactcgtatctccccattcatatatgtttgggtgaaagttccccatcttaacagggtatcaccgggca
GA2e.Bsal.DSM.18170   99.7%  79.1%  gtctttccggtaaatccgcaggtctccgtcctcgtacatcctgctgtacgaggcccatctcagcagtccgtcccccggca
consensus/100%                      uTCsTTssGuTAsAssCGsAssTCsCCuTsCssusussssGsTGsAsGsssCCCAsCssAuCAGsssuTssCCsGGCA
consensus/90%                       uTCsTTssGuTAsAssCGsAssTCsCCuTsCssusussssGsTGsAsGsssCCCAsCssAuCAGsssuTssCCsGGCA
consensus/80%                       GTCsTTCsGuTAuAssCGsAssTCsCCGTsCTCususGssTsGsTGTAsGsusCCCATCTCAuCAGssCGTCACCCGGCA
consensus/70%                       GTCsTTCsGuTAuAssCGsAssTCsCCGTsCTCususGssTsGsTGTAsGsusCCCATCTCAuCAGssCGTCACCCGGCA

                        cov     pid 10081.       .       .       .       .       .       .       .       . 10160
GA2a.Bcac.CL03T12C61 100.0% 100.0%  tacctgcaagggtacaggtaacgaaaccgccgtatgcctcaccttccggcaatccgtcacggtcggtattccgtttgaat
GA2b.Bfra.CL05T12C13  99.7%  81.0%  tcccgctgagggtacaggtgacgaatccgccgtaggcttcaccttcaggcaatccgtcacggtctgtattccgcttgaat
GA2c.Bvul.TF05-18     99.8%  82.0%  taccggtaagggtacaggtaatgaatccgccgtatgcctcaccttcaggcaatccgtcacggtctgtattccgcttgaat
GA2d.Buni.BIOML-A83   99.2%  69.8%  ttccctgtaacatacacataatgataccaccgtatgcttccccttccggaaaaccgatacgattagtatcacgcttaaat
GA2e.Bsal.DSM.18170   99.7%  79.1%  tgccggtaagtgtgcaggtgacaaagccgccgtatgcctcaccctccggcagtccgtcgcggtcggtatcgcgcttgaat
consensus/100%                      TsCCssssAusuTuCAsuTuAsuAssCCuCCGTAsGCsTCsCCsTCsGGsAusCCGssuCGuTssGTATssCGsTTuAAT
consensus/90%                       TsCCssssAusuTuCAsuTuAsuAssCCuCCGTAsGCsTCsCCsTCsGGsAusCCGssuCGuTssGTATssCGsTTuAAT
consensus/80%                       TsCCsssuAGsGTACAGGTuAsGAAsCCGCCGTATGCsTCACCTTCsGGCAATCCGTCACGGTCsGTATssCGCTTGAAT
consensus/70%                       TsCCsssuAGsGTACAGGTuAsGAAsCCGCCGTATGCsTCACCTTCsGGCAATCCGTCACGGTCsGTATssCGCTTGAAT

                        cov     pid 10161.       .       .       .       .       .       .       .       . 10240
GA2a.Bcac.CL03T12C61 100.0% 100.0%  tgcaggtcaaattcttctaccgtgtatatctcgtgacatacggacagcttcatcgcataacggtcattcaccgttccggg
GA2b.Bfra.CL05T12C13  99.7%  81.0%  tgcaggtcgaattcttcaatgctgtatgtctcatggcagacggacagccgcatcacgtagcggtcatccggcattgagga
GA2c.Bvul.TF05-18     99.8%  82.0%  tgcaggtcgaactcttcaatgctgtatgtctcatggcagacggacagccgcatcacgtagcggtcatccggcattgagga
GA2d.Buni.BIOML-A83   99.2%  69.8%  tgcaggttgaagtcttcaatatcatatatttcttcacccacgcacaatttcattatgtaatggtcatcatttcccattac
GA2e.Bsal.DSM.18170   99.7%  79.1%  ttcaggtcaaactcctctaccgcgtaggtctcgtggcagacagtcagccgcattacgtaccggtcatccggtacggcggc
consensus/100%                      TsCAGGTsuAAsTCsTCsAssssuTAsuTsTCsTsuCssACussCAusssCATsusuTAssGGTCATssssssssssus
consensus/90%                       TsCAGGTsuAAsTCsTCsAssssuTAsuTsTCsTsuCssACussCAusssCATsusuTAssGGTCATssssssssssus
consensus/80%                       TGCAGGTCuAAsTCTTCsAssssGTATuTCTCuTGuCAsACGGACAGCssCATsACGTAuCGGTCATCCussussusGGs
consensus/70%                       TGCAGGTCuAAsTCTTCsAssssGTATuTCTCuTGuCAsACGGACAGCssCATsACGTAuCGGTCATCCussussusGGs
```

```
                        cov     pid 10241.       .         .         .         .         .         .         .         . 10320
GA2a.Bcac.CL03T12C61 100.0% 100.0%  ctctttccgtttaaacaaggcagcaaaccagttcataatctcatttcaaatttaggtgacacgaatcgccggcttccatt
GA2b.Bfra.CL05T12C13  99.7%  81.0%  ttttttgtttttgaacaatgaagtgaaccagttcatg-----attataagtttaagtgacacgaatcgccggtctccatt
GA2c.Bvul.TF05-18     99.8%  82.0%  ttttttttgcttaaacaaagaggtaaaccagttcatg-----attataagtttaagtgacacgaatcgccggtctccatt
GA2d.Buni.BIOML-A83   99.2%  69.8%  atttttctttttgaatagggtattgaaccagctcatagcat--tcacatatttaagtagcacgaatctccagtttccatt
GA2e.Bsal.DSM.18170   99.7%  79.1%  gtcttttcgtttgaacaaagaggtaaaccagttcatg-----attttaagtttaagtgacacgaatcaccggcttccatt
consensus/100%                      sTsTTTssssTTuAAsAusGsussuAACCAGsTCATu......TsssAsuTTTAuGTuuCACGAATCsCCuGssTCCATT
consensus/90%                       sTsTTTssssTTuAAsAusGsussuAACCAGsTCATu......TsssAsuTTTAuGTuuCACGAATCsCCuGssTCCATT
consensus/80%                       sTsTTTsssTTTuAACAAuGsuGTuAACCAGTTCATu.....ATTssAAuTTTAAGTGACACGAATCuCCGGssTCCATT
consensus/70%                       sTsTTTsssTTTuAACAAuGsuGTuAACCAGTTCATu.....ATTssAAuTTTAAGTGACACGAATCuCCGGssTCCATT

                        cov     pid 10321.       .         .         .         .         .         .         .         . 10400
GA2a.Bcac.CL03T12C61 100.0% 100.0%  tccctgaaatggataagcagcgaggtacaactctcttttacggtcgaatgcacatggaaatccagacattgggctccctt
GA2b.Bfra.CL05T12C13  99.7%  81.0%  tcctggaagtggatgaatagtgaggtacggctctctttggttgttgaatgcacattgaaatcaaggcatttgctccctt
GA2c.Bvul.TF05-18     99.8%  82.0%  tcccggaagtggatgaatagtgatatacggctctctttggttgttgaatgcacattgaaatcaaggcactgtgcgccttt
GA2d.Buni.BIOML-A83   99.2%  69.8%  tccttaaaatgaatcagaagtttagtccgacattctttggttgtagagtgaacattcatttcaagacactgtgcgcccct
GA2e.Bsal.DSM.18170   99.7%  79.1%  tccctgaaatgaatggatagcgaagtccggctttcttttactgtcgaatgcacgtcgagttcaaggcattgggcgcctct
consensus/100%                      TCCssuAAuTGuATsuusAGssssuTsCuuCssTCTTTsussGTsGAuTGsACuTssAssTCsAGuCAsTssGCsCCssT
consensus/90%                       TCCssuAAuTGuATsuusAGssssuTsCuuCssTCTTTsussGTsGAuTGsACuTssAssTCsAGuCAsTssGCsCCssT
consensus/80%                       TCCssGAAuTGuATuAusAGsGAuGTsCGuCTsTCTTTsusTGTsGAATGCACATsGAusTCAAGuCAsTGsGCsCCssT
consensus/70%                       TCCssGAAuTGuATuAusAGsGAuGTsCGuCTsTCTTTsusTGTsGAATGCACATsGAusTCAAGuCAsTGsGCsCCssT

                        cov     pid 10401.       .         .         .         .         .         .         .         . 10480
GA2a.Bcac.CL03T12C61 100.0% 100.0%  gaacctgattgtttccttcggtgttcccagctttcccgaagtattcatcacttccccgcttttcaggatacgtgaattga
GA2b.Bfra.CL05T12C13  99.7%  81.0%  gaaacggacaatctcttgggagtaccgggcaggtcggaagtgttcatcacctctccgtccttcaggattcttgaattga
GA2c.Bvul.TF05-18     99.8%  82.0%  gaaacggatgatctcttgggagtaccggacaggccggaagtgttcatcacctctccgtccttcaggattcttgaattga
GA2d.Buni.BIOML-A83   99.2%  69.8%  gaagcgtataacttctttctgccttccaagaatacctgaagaattcattaattctccattttatggatttgcgaactaa
GA2e.Bsal.DSM.18170   99.7%  79.1%  gaaacggataacttccttgggcgcaccgctcaagccggaagtgttcattacctccccgtctttcaggattcttgaattga
consensus/100%                      GAAsCssAssussTCsTTssGssssCCsssssssCsGAAGsuTTCATsAssTCsCCusssTTssGGATssssGAAsTuA
consensus/90%                       GAAsCssAssussTCsTTssGssssCCsssssssCsGAAGsuTTCATsAssTCsCCusssTTssGGATssssGAAsTuA
consensus/80%                       GAAuCGGATuAssTCsTTsGGsGTsCCuuuCAsuCCsGAAGTuTTCATsACsTCsCCGTssTTCAGGATTCsTGAATTGA
consensus/70%                       GAAuCGGATuAssTCsTTsGGsGTsCCuuuCAsuCCsGAAGTuTTCATsACsTCsCCGTssTTCAGGATTCsTGAATTGA

                        cov     pid 10481.       .         .         .         .         .         .         .         . 10560
GA2a.Bcac.CL03T12C61 100.0% 100.0%  acatccattccagcaactccttcgagggaaatccggctatgcttacctgcatctgtcccccggtggtctttccggccgct
GA2b.Bfra.CL05T12C13  99.7%  81.0%  acatccattccaagagttcctctgtaggaaatccggcaatgcttacccgtatttaccgccgtaggtcttcccggttacc
GA2c.Bvul.TF05-18     99.8%  82.0%  atatccattccaagagttcctccgttggaaatccggcaatacttacccgtatttaccgccgtatgtctttccggttact
GA2d.Buni.BIOML-A83   99.2%  69.8%  gcatccacgccaataagtcatcagtcggaaagcctgatattataacaccaatctttccgccttgcatattgccgattacc
GA2e.Bsal.DSM.18170   99.7%  79.1%  acatccattccagcagttcttccgcagggaatcccgcaatgcttgcccgcatcctgcctccgtgtgtctcgccagttact
consensus/100%                      usATCCAssCCAusAusTCsTssGssGGuAAsCCsGssATssTsuCssssATssssCCsCCssssuTsTssCCuussuCs
consensus/90%                       usATCCAssCCAusAusTCsTssGssGGuAAsCCsGssATssTsuCssssATssssCCsCCssssuTsTssCCuussuCs
consensus/80%                       ACATCCATTCCAusAusTCsTCsGssGGAAATCCsGCsATuCTTACCCGsATsTTsCCsCCGTusGTCTTsCCGGTTACs
consensus/70%                       ACATCCATTCCAusAusTCsTCsGssGGAAATCCsGCsATuCTTACCCGsATsTTsCCsCCGTusGTCTTsCCGGTTACs
```

```
                                cov     pid 10561.       .         .         .         .         .         .         .         . 10640
GA2a.Bcac.CL03T12C61 100.0% 100.0%  tgcccgcatggggtcatcggcttctggaagtcatattcataactcaacaggtcgtaagcctgcacctcatcatgttccga
GA2b.Bfra.CL05T12C13  99.7%  81.0%  tttccattaggagtggtttctttccggaagtcatactcgtagcccagcaggtcgtatgcctgcacttcctcatgctccga
GA2c.Bvul.TF05-18     99.8%  82.0%  tgcccgctataggtggtctgtttccggaagtcatactcgtagcccagcaggtcgtatgcctgcacttcctcatgctccga
GA2d.Buni.BIOML-A83   99.2%  69.8%  cgtccagtcttatcagcatcctgactgaaatcatattcatattccaataggtcataagtctgaacatcttcatgttccga
GA2e.Bsal.DSM.18170   99.7%  79.1%  tgtccgttcgggcgggcctgtttctcgaagtcatactcaaagctcagcaggtcgtatgcctgcacttcttcatgctccga
consensus/100%                      sssCCussssssusssussssssTssssGAAuTCATAsTCusAsssCAusAGGTCuTAsGsCTGsACsTCsTCATGsTCCGA
consensus/90%                       sssCCussssssusssussssssTssssGAAuTCATAsTCusAsssCAusAGGTCuTAsGsCTGsACsTCsTCATGsTCCGA
consensus/80%                       TGsCCusTssuussuGssTssTTCssGAAGTCATAsTCuTAuCsCAuCAGGTCGTAsGCCTGCACsTCsTCATGsTCCGA
consensus/70%                       TGsCCusTssuussuGssTssTTCssGAAGTCATAsTCuTAuCsCAuCAGGTCGTAsGCCTGCACsTCsTCATGsTCCGA

                                cov     pid 10641.       .         .         .         .         .         .         .         . 10720
GA2a.Bcac.CL03T12C61 100.0% 100.0%  gggctcggcggtccaaagtttgatatacgattgtgcactcatttatttcacccaccgattatcaaattcaataccattca
GA2b.Bfra.CL05T12C13  99.7%  81.0%  gggttcggcggtccagaacttgatatacgattgtgcactcattta---cacccatctattgtcgaaatcaacaccttgaa
GA2c.Bvul.TF05-18     99.8%  82.0%  gggttcggctgtccagaacttgatatacgattgcgcactcatttatttcacccaccgattatcaaattcaataccattca
GA2d.Buni.BIOML-A83   99.2%  69.8%  tggctcagcagtccataatttgatatatgattgtgcactcattta-----------------------------------
GA2e.Bsal.DSM.18170   99.7%  79.1%  gggttcggcggtccagaacttgatatatgattgtgcactcatttatttttacccatctattatcaaattcaataccattca
consensus/100%                      sGGsTCuGCsGTCCAsAusTTGATATAsGATTGsGCACTCATTTA...................................
consensus/90%                       sGGsTCuGCsGTCCAsAusTTGATATAsGATTGsGCACTCATTTA...................................
consensus/80%                       GGGsTCGGCuGTCCAuAAsTTGATATAsGATTGTGCACTCATTTA...sACCCAsCsATTuTCuAAsTCAAsACCsTssA
consensus/70%                       GGGsTCGGCuGTCCAuAAsTTGATATAsGATTGTGCACTCATTTA...sACCCAsCsATTuTCuAAsTCAAsACCsTssA

                                cov     pid 10721.       .         .         .         .         .         .         .         . 10800
GA2a.Bcac.CL03T12C61 100.0% 100.0%  cgcttacactttcaggagacaaaatcaaggtagttgacaagccttgccccaaagattcaataacccgtttcaaattgaca
GA2b.Bfra.CL05T12C13  99.7%  81.0%  cagttaatgtttcaggagaaatcgttaatgttgtagataaccctgtccgaaac---tgatacttctgtgtagccttatg
GA2c.Bvul.TF05-18     99.8%  82.0%  cgcttacactctcaggagagagaatcaaagtagttgacaatccttgccccaaagcttcgacaacccgtttcagattgaca
GA2d.Buni.BIOML-A83   99.2%  69.8%  ----------------------------------------------------------------tattgtttcattgaca
GA2e.Bsal.DSM.18170   99.7%  79.1%  cgctaacactctcaggagatagaattagagtagttgacaatccttgtcccaaagcctctataactcgtctcagattgaca
consensus/100%                      ................................................................sssssssssssTsAsu
consensus/90%                       ................................................................sssssssssssTsAsu
consensus/80%                       CusTsAsssTsTCAGGAGAsAssuTsAusGTsGTsGAsAAsCCsTGsCCsAAAs...ssAsAsssCsTsTsAuATTGACA
consensus/70%                       CusTsAsssTsTCAGGAGAsAssuTsAusGTsGTsGAsAAsCCsTGsCCsAAAs...ssAsAsssCsTsTsAuATTGACA

                                cov     pid 10801.       .         .         .         .         .         .         .         . 10880
GA2a.Bcac.CL03T12C61 100.0% 100.0%  caatacgcattcgtaaacgccacctcaaatatcaccccggacgtaccggtcttgaacagtaccgccccgctctttttcat
GA2b.Bfra.CL05T12C13  99.7%  81.0%  caatacgcgttcataaacgccacctcaaatatcaccccggacgttccggtcttgaacagaactgccccgttctttttcac
GA2c.Bvul.TF05-18     99.8%  82.0%  cagtacgcattcgtaaatgccacctcaaatatcaccccggacgttccggtcttgaacagaactgccccgttctttttcac
GA2d.Buni.BIOML-A83   99.2%  69.8%  caatatgcatttataaatgccacctcaaacactactccagacgtaccagtttaaacaataccgccccgttctttttttac
GA2e.Bsal.DSM.18170   99.7%  79.1%  cagtacgcatttgtaaacgccacctcaaatatcaccccggacgtaccggtcttgaacagcaccgccccgttcttctttac
consensus/100%                      CAuTAsGCuTTsuTAAAsGCCACCTCAAAsAssACsCCuGACGTsCCuGTsTTuAACAusACsGCCCCGsTCTTsTTsAs
consensus/90%                       CAuTAsGCuTTsuTAAAsGCCACCTCAAAsAssACsCCuGACGTsCCuGTsTTuAACAusACsGCCCCGsTCTTsTTsAs
consensus/80%                       CAuTACGCATTsuTAAAsGCCACCTCAAATATCACCCCGGACGTsCCGGTCTTGAACAGsACsGCCCCGTTCTTTTTsAC
consensus/70%                       CAuTACGCATTsuTAAAsGCCACCTCAAATATCACCCCGGACGTsCCGGTCTTGAACAGsACsGCCCCGTTCTTTTTsAC
```

```
                      cov    pid 10881.         .         .         .         .         .         .         .         . 10960
GA2a.Bcac.CL03T12C61 100.0% 100.0%  ccacggtttcacggcccatccgtagatgttgttcttcactgtttgcgaaagagtaatcatgatgcgtccgccacgcactt
GA2b.Bfra.CL05T12C13  99.7%  81.0%  ccacggtttcacagcccatccgtagatgttgcttttcaccgtctgcgaaagggttatcatgattcgtccccctcgcactt
GA2c.Bvul.TF05-18     99.8%  82.0%  ccacggtttcacagcccatccgtagatgttgcttttcaccgtctgcgaaagggttatcatgattcgtcctccacgcactt
GA2d.Buni.BIOML-A83   99.2%  69.8%  ccaaggcttgattgcccatccgtagatgttatttttaacggtttgtgacaatgttatcataatacgccctccacgaactt
GA2e.Bsal.DSM.18170   99.7%  79.1%  ccacggcttgatggcccatccgtagatattgcttttcaccgtttgcgaaaggacaatcatgattcgccctccgcgcactt
consensus/100%                      CCAsGGsTTsAssGCCCATCCGTAGATuTTusTsTTsACsGTsTGsGAsAusussATCATuATsCGsCCsCCsCGsACTT
consensus/90%                       CCAsGGsTTsAssGCCCATCCGTAGATuTTusTsTTsACsGTsTGsGAsAusussATCATuATsCGsCCsCCsCGsACTT
consensus/80%                       CCACGGsTTsAsuGCCCATCCGTAGATGTTGsTTTTCACsGTsTGCGAAAGuGTsATCATGATsCGsCCsCCuCGCACTT
consensus/70%                       CCACGGsTTsAsuGCCCATCCGTAGATGTTGsTTTTCACsGTsTGCGAAAGuGTsATCATGATsCGsCCsCCuCGCACTT

                      cov    pid 10961.         .         .         .         .         .         .         .         . 11040
GA2a.Bcac.CL03T12C61 100.0% 100.0%  ccccttccggctgattctttatgccatcgacaggctggtgaaaaccgattttgaactgttccactgcatactcgtcaccg
GA2b.Bfra.CL05T12C13  99.7%  81.0%  cgccttccggttggttctttaggttatctactggctggtgaaaacctatcttgaactgttccactgcatattcgtcgccg
GA2c.Bvul.TF05-18     99.8%  82.0%  cgccttccggttggttctttagattatctactggctggtgaaaacctattttgaactgttccactgcatattcatcgccg
GA2d.Buni.BIOML-A83   99.2%  69.8%  caccttccggttgattcttattaccatcaacaggctggtgaaagccgattttgaactgttcgacttcatactcgtcacca
GA2e.Bsal.DSM.18170   99.7%  79.1%  ctccttccggttggttcttgtttccgtcaacaggctggtggaaacctatcttgaactgttctaccgcatattcgtcgccg
consensus/100%                      CsCCTTCCGGsTGuTTCTTssssssuTCsACsGGCTGGTGuAAuCCsATsTTGAACTGTTCsACssCATAsTCuTCuCCu
consensus/90%                       CsCCTTCCGGsTGuTTCTTssssssuTCsACsGGCTGGTGuAAuCCsATsTTGAACTGTTCsACssCATAsTCuTCuCCu
consensus/80%                       CsCCTTCCGGTTGuTTCTTsssussATCsACsGGCTGGTGAAAACCsATsTTGAACTGTTCsACTGCATAsTCGTCuCCG
consensus/70%                       CsCCTTCCGGTTGuTTCTTsssussATCsACsGGCTGGTGAAAACCsATsTTGAACTGTTCsACTGCATAsTCGTCuCCG

                      cov    pid 11041.         .         .         .         .         .         .         .         . 11120
GA2a.Bcac.CL03T12C61 100.0% 100.0%  tcaacactgaagatgacattcacattgctgtccacttgcggaaaactaaagagtcccataacattgttgtttttaaaata
GA2b.Bfra.CL05T12C13  99.7%  81.0%  tcaacgctgaatatgacattcacgttgctgtccacttgcggaaaattaaaaagtcccataacgctgctgtttttaaaaca
GA2c.Bvul.TF05-18     99.8%  82.0%  tcaacgctgaatatgacattcacgttgctgtccacttgcggaaaattaaaaagtcccataacgctgctgtttttaaaaca
GA2d.Buni.BIOML-A83   99.2%  69.8%  tcaacgcagaatattacattcacgttgctgtccacttgcggaaaattaaaaagtcccataacatt-ttctttttagagtg
GA2e.Bsal.DSM.18170   99.7%  79.1%  tcaatgcagaatatgacattcacatcgctgtccacttgtggaaaattaaagagtcccatagcattatcttttttagagta
consensus/100%                      TCAAsuCsGAAsATsACATTCACuTsGCTGTCCACTTGsGGAAAAsTAAAuAGTCCCATAuCusT.sssTTTTTAuAusu
consensus/90%                       TCAAsuCsGAAsATsACATTCACuTsGCTGTCCACTTGsGGAAAAsTAAAuAGTCCCATAuCusT.sssTTTTTAuAusu
consensus/80%                       TCAACGCsGAATATGACATTCACuTTGCTGTCCACTTGCGGAAAATTAAAuAGTCCCATAACusTusTsTTTTTAuAusA
consensus/70%                       TCAACGCsGAATATGACATTCACuTTGCTGTCCACTTGCGGAAAATTAAAuAGTCCCATAACusTusTsTTTTTAuAusA

                      cov    pid 11121.         .         .         .         .         .         .         .         . 11200
GA2a.Bcac.CL03T12C61 100.0% 100.0%  cccactgattttcaaattcctccgacccgaacgtcagcttctgagcctgcacggtcagcttggttgcgatatagctcttc
GA2b.Bfra.CL05T12C13  99.7%  81.0%  cccactgattttcaaactcttctgtaccgaatgccagtttctgtgcctgtacggtcagtttggtcgcaatatagcttttg
GA2c.Bvul.TF05-18     99.8%  82.0%  cccactgattttcaaactcttctgtaccgaatgccagtttctgtgcctgtacggtcagtttggtcgcaatatagcttttg
GA2d.Buni.BIOML-A83   99.2%  69.8%  cccactgattttcaaattcttctgtaccgaacattaacttctgtgcttgtacggtaagtttagttgcgatatagctcttg
GA2e.Bsal.DSM.18170   99.7%  79.1%  cccactggttttcaaattcctctgtaccgaacatcagtttctgtgcctgtacggtcagtttggtcgcaatatagccctta
consensus/100%                      CCCACTGuTTTTCAAAsTCsTCsGssCCGAAsussAusTTCTGsGCsTGsACGGTsAGsTTuGTsGCuATATAGCssTTs
consensus/90%                       CCCACTGuTTTTCAAAsTCsTCsGssCCGAAsussAusTTCTGsGCsTGsACGGTsAGsTTuGTsGCuATATAGCssTTs
consensus/80%                       CCCACTGATTTTCAAAsTCsTCTGTACCGAAsusCAGsTTCTGTGCCTGTACGGTCAGTTTGGTsGCuATATAGCTsTTu
consensus/70%                       CCCACTGATTTTCAAAsTCsTCTGTACCGAAsusCAGsTTCTGTGCCTGTACGGTCAGTTTGGTsGCuATATAGCTsTTu
```

```
                              cov    pid 11201.        .         .         .         .         .         .         .        . 11280
GA2a.Bcac.CL03T12C61 100.0% 100.0%   ccctgccggatatagctgatttccatatccacacaggtcgctttctcaaaaagtatcttttcgagcgtggaaccctggtt
GA2b.Bfra.CL05T12C13  99.7%  81.0%   ccctgccggatatagctgatttccatatccacacaagtcgctttctcaaagagtatcttttcgagcgtggagccttggtt
GA2c.Bvul.TF05-18     99.8%  82.0%   ccctgccggatatagctgatttccatatccacacaggtcgctttctcaaagagtatcttttcgagtgtggagccttggtt
GA2d.Buni.BIOML-A83   99.2%  69.8%   ccctgtcggatatagctgatttccatatccacacaggtcgctttctcaaagagtatcttttcaagagtggaaccttggtt
GA2e.Bsal.DSM.18170   99.7%  79.1%   ccctgccggatatagctgatttccatatccacgcaggttgctttctcaaagagtatcttctcaagcgcggagccttggtt
consensus/100%                       CCCTGsCGGATATAGCTGATTTCCATATCCACuCAuGTsGCTTTCTCAAAuAGTATCTTsTCuAGsGsGGAuCCsTGGTT
consensus/90%                        CCCTGsCGGATATAGCTGATTTCCATATCCACuCAuGTsGCTTTCTCAAAuAGTATCTTsTCuAGsGsGGAuCCsTGGTT
consensus/80%                        CCCTGCCGGATATAGCTGATTTCCATATCCACACAGGTCGCTTTCTCAAAGAGTATCTTTTCuAGsGTGGAuCCTTGGTT
consensus/70%                        CCCTGCCGGATATAGCTGATTTCCATATCCACACAGGTCGCTTTCTCAAAGAGTATCTTTTCuAGsGTGGAuCCTTGGTT

                              cov    pid 11281.        .         .         .         .         .         .         .        . 11360
GA2a.Bcac.CL03T12C61 100.0% 100.0%   gtcatgaaccacgacggctccattctgatacttgcgggcatccagcatccactggatcatgtcacggttgggaagatggg
GA2b.Bfra.CL05T12C13  99.7%  81.0%   gtcatgaactacgatggctccgctctggtacttacgggcatccagcatccactggatcatatcacggttgggaagatgag
GA2c.Bvul.TF05-18     99.8%  82.0%   gtcatgaactacgatggctccgctctggtatttacgggcatccagcatccactggatcatgtcacggttgggaacatgag
GA2d.Buni.BIOML-A83   99.2%  69.8%   atcatgaacgacaatagctccgctctgatatttgcgggcatccaacatccactgaatcatatcccggttaggcagatgag
GA2e.Bsal.DSM.18170   99.7%  79.1%   gtcgtgcacgacaatggcgccgctctgatatttgcgcgcatccagcatccactggatcatgtcccggttgggcaggtggg
consensus/100%                       uTCuTGsACsACuAsuGCsCCusTCTGuTAsTTuCGsGCATCCAuCATCCACTGuATCATuTCsCGGTTuGGsAsuTGuG
consensus/90%                        uTCuTGsACsACuAsuGCsCCusTCTGuTAsTTuCGsGCATCCAuCATCCACTGuATCATuTCsCGGTTuGGsAsuTGuG
consensus/80%                        GTCATGAACsACuATGGCTCCGCTCTGuTAsTTuCGGGCATCCAGCATCCACTGGATCATuTCsCGGTTGGGsAGATGuG
consensus/70%                        GTCATGAACsACuATGGCTCCGCTCTGuTAsTTuCGGGCATCCAGCATCCACTGGATCATuTCsCGGTTGGGsAGATGuG

                              cov    pid 11361.        .         .         .         .         .         .         .        . 11440
GA2a.Bcac.CL03T12C61 100.0% 100.0%   ggtaagtcacatagaacgtaccgcctttcacctcggtctgggcctgtcctttgatgtctatcccctgggcgaacccgaac
GA2b.Bfra.CL05T12C13  99.7%  81.0%   ggtaggttacataaaacgtgccgccttttacctcggtctgtacctgtcctttgatatctatcccttgggcaaatccgaat
GA2c.Bvul.TF05-18     99.8%  82.0%   ggtaggtcacatagaacgtaccgccttttacctcggtctgtgcctgtcctttgatatctatcccttgggcaaatccgaat
GA2d.Buni.BIOML-A83   99.2%  69.8%   ggtaagttacatagaaagtacctcccttaacttctgtttgtacctgacctttaaaatcaataccttgggcgaagccaaat
GA2e.Bsal.DSM.18170   99.7%  79.1%   gataggtcacataaaaggttccgcccttgacttccgtctgcacttgtcccttgacatctatcccttgggcgaatccgaac
consensus/100%                       GuTAuGTsACATAuAAsGTsCCsCCsTTsACsTCsGTsTGsuCsTGsCCsTTuAsuTCsATsCCsTGGGCuAAsCCuAAs
consensus/90%                        GuTAuGTsACATAuAAsGTsCCsCCsTTsACsTCsGTsTGsuCsTGsCCsTTuAsuTCsATsCCsTGGGCuAAsCCuAAs
consensus/80%                        GGTAuGTsACATAuAAsGTuCCGCCsTTsACsTCsGTCTGsuCCTGTCCTTTGAsATCTATCCCTTGGGCuAAsCCGAAs
consensus/70%                        GGTAuGTsACATAuAAsGTuCCGCCsTTsACsTCsGTCTGsuCCTGTCCTTTGAsATCTATCCCTTGGGCuAAsCCGAAs

                              cov    pid 11441.        .         .         .         .         .         .         .        . 11520
GA2a.Bcac.CL03T12C61 100.0% 100.0%   tcacagccgataagctggtaactgtccttatacatatcgaccagcgaagaagcggcaagattgccgatctttaagaatac
GA2b.Bfra.CL05T12C13  99.7%  81.0%   tcgcagccgatgagctggtagctgtccttgtacatgtcggtcagtgaggaggcagccaggttgcctatctttaagaatac
GA2c.Bvul.TF05-18     99.8%  82.0%   tcgcagccgatgagctggtagctgtccttgtacatgtcggtcagtgaggaggcagccaggttgcctatctttaagaatac
GA2d.Buni.BIOML-A83   99.2%  69.8%   tcacaccctatcagttgatagctgtccttatacatatccgtcagcgatgtagctgccagattgccgattttttaaaaatac
GA2e.Bsal.DSM.18170   99.7%  79.1%   tcacaacctatcagttgataactgtccttatacatgtctgtcagcgaggtggctgccagattgccgattttttaagaatac
consensus/100%                       TCuCAsCCsATsAGsTGuTAuCTGTCCTTuTACATuTCsusCAGsGAsGsuGCsGCsAGuTTGCCsATsTTTAAuAATAC
consensus/90%                        TCuCAsCCsATsAGsTGuTAuCTGTCCTTuTACATuTCsusCAGsGAsGsuGCsGCsAGuTTGCCsATsTTTAAuAATAC
consensus/80%                        TCuCAuCCsATsAGsTGuTAuCTGTCCTTuTACATuTCsGTCAGsGAuGsuGCsGCCAGuTTGCCsATsTTTAAGAATAC
consensus/70%                        TCuCAuCCsATsAGsTGuTAuCTGTCCTTuTACATuTCsGTCAGsGAuGsuGCsGCCAGuTTGCCsATsTTTAAGAATAC
```

```
                    cov    pid 11521.      .       .       .       .       .       .       .       . 11600
GA2a.Bcac.CL03T12C61 100.0% 100.0% tgtatatccaaacatactgtacttga---ttattggtgaatattccaggttaattcattctgttcgtccagacccacctc
GA2b.Bfra.CL05T12C13  99.7%  81.0% ggtatatccaaacatacgatacttga---ttattggtgaatattccaggttaattcattctgttcgtccagacctacctc
GA2c.Bvul.TF05-18     99.8%  82.0% ggtatatccaaacatagtgtacttga---ttattggtgaatattccaggttaattcattctgttcgtccagacctacctc
GA2d.Buni.BIOML-A83   99.2%  69.8% tgtatatccaaacataagaatcttga---tttcagtgaatatcccaagttaattcattcgcgtcatccagtcctacctt
GA2e.Bsal.DSM.18170   99.7%  79.1% tgtgtatccaaacatagtgcatacaaatttaggagtgaatattcaaggttaattcgtttcgttcatccaggccgacctc
consensus/100%                     sGTuTATCCAAACATAssusssssuA...TTsssuGTGAATATsCsAuGTTAATTCuTTssssTCuTCCAGsCCsACCTs
consensus/90%                      sGTuTATCCAAACATAssusssssuA...TTsssuGTGAATATsCsAuGTTAATTCuTTssssTCuTCCAGsCCsACCTs
consensus/80%                      sGTATATCCAAACATAssusACTTGA...TTATsuGTGAATATTCCAGGTTAATTCATTCsGTTCuTCCAGuCCsACCTC
consensus/70%                      sGTATATCCAAACATAssusACTTGA...TTATsuGTGAATATTCCAGGTTAATTCATTCsGTTCuTCCAGuCCsACCTC

                    cov    pid 11601.      .       .       .       .       .       .       .       . 11680
GA2a.Bcac.CL03T12C61 100.0% 100.0% cagattttttcctttgcacagctcttcattgatgataagccgcgaaatcggtctgcgcaggtagttgcggataaccccgg
GA2b.Bfra.CL05T12C13  99.7%  81.0% cagactttttcctttgcccagctcctcgttgatgataagccgtgaaatcggtctgcgcagatagttacggatcacaccgg
GA2c.Bvul.TF05-18     99.8%  82.0% cagactttttcctttgcccagctcctcgttgatgataagccgtgaaatcggcggcgcaggtagttacggatgaccccgg
GA2d.Buni.BIOML-A83   99.2%  69.8% caagattttgcctttcgataactcttcctttattataagacggaaataggcctacgtaaataattacggatcacaccgg
GA2e.Bsal.DSM.18170   99.7%  79.1% cagtgtctttccctggcacagttcctctttgataatgaggcgcgatatgggtctgcgcagatagttacggattacaccgg
consensus/100%                     CAussTsTTsCCsTsssssAusTCsTCsTTsATsATuAGsCGsGAsATsGGsCsuCGsAuuTAuTTuCGGATsACsCCGG
consensus/90%                      CAussTsTTsCCsTsssssAusTCsTCsTTsATsATuAGsCGsGAsATsGGsCsuCGsAuuTAuTTuCGGATsACsCCGG
consensus/80%                      CAGusTTTTTCCTTTGCsCAGCTCsTCsTTGATuATAAGsCGsGAAATsGGsCTGCGCAGuTAGTTACGGATsACsCCGG
consensus/70%                      CAGusTTTTTCCTTTGCsCAGCTCsTCsTTGATuATAAGsCGsGAAATsGGsCTGCGCAGuTAGTTACGGATsACsCCGG

                    cov    pid 11681.      .       .       .       .       .       .       .       . 11760
GA2a.Bcac.CL03T12C61 100.0% 100.0% caacctgacgggcgccgtatttgggagtgaatcctttgtgggcaagcattttccgggcatcgtcactgatggcgataccg
GA2b.Bfra.CL05T12C13  99.7%  81.0% ccacttggcgggcaccgtatttgggagtgaatcctttgtgtgcaagcattttacgggcatcatcgctaatggtgatgccg
GA2c.Bvul.TF05-18     99.8%  82.0% caacttggcgggcaccgtatttgggagtgaagcctttgtgggcgagcatcttccgggcatcatcgctgatggtgatgccg
GA2d.Buni.BIOML-A83   99.2%  69.8% caacctgccttgctccatatttaggggtaaatcctttatgcgcaagcagcttacgggcatcatcacttatttcaattgct
GA2e.Bsal.DSM.18170   99.7%  79.1% ccacttggcgtgcgccgtatttgggagtaaatcccttatgggcaagcatctttcgggcatcgtcactgatgatgatgttg
consensus/100%                     CsACsTGsCssGCsCCuTATTTuGGuGTuAAsCCsTTuTGsGCuAGCAssTTsCGGGCATCuTCuCTsATsssuATssss
consensus/90%                      CsACsTGsCssGCsCCuTATTTuGGuGTuAAsCCsTTuTGsGCuAGCAssTTsCGGGCATCuTCuCTsATsssuATssss
consensus/80%                      CsACsTGuCGsGCuCCGTATTTGGGAGTuAATCCTTTuTGsGCAAGCATsTTsCGGGCATCuTCuCTuATGusGATusCG
consensus/70%                      CsACsTGuCGsGCuCCGTATTTGGGAGTuAATCCTTTuTGsGCAAGCATsTTsCGGGCATCuTCuCTuATGusGATusCG

                    cov    pid 11761.      .       .       .       .       .       .       .       . 11840
GA2a.Bcac.CL03T12C61 100.0% 100.0% ataccttgcttgtccagcagtttcctgacactgttgaactgtatatcgaatatcttcaacagaatgtcttcacggatagg
GA2b.Bfra.CL05T12C13  99.7%  81.0% ataccctgcttgtccagaagttttctgactccattgaactgtatgtcgaatatttcaggagtatgtcttcgcggatggg
GA2c.Bvul.TF05-18     99.8%  82.0% ataccctgcttgtccagaagttttctgactccgttgaactgtatatcgaatattttaggagtatctcttcacggatggg
GA2d.Buni.BIOML-A83   99.2%  69.8% ataccttgacggtcaagcagttttctaaggccattaaactgtatatcgaatatcatcagcaaaatatcctcgctgatagg
GA2e.Bsal.DSM.18170   99.7%  79.1% ataccctgtttgtccagtagtttcttgaccccgttgaattgtatatcgaatatttcaataggatatcttcgctgatagg
consensus/100%                     ATACCsTGsssGTCsAGsAGTTTssTuAssCsuTTuAAsTGTATuTCGAATATssTsAusAusATsTCsTCuCsGATuGG
consensus/90%                      ATACCsTGsssGTCsAGsAGTTTssTuAssCsuTTuAAsTGTATuTCGAATATssTsAusAusATsTCsTCuCsGATuGG
consensus/80%                      ATACCsTGsTTGTCCAGsAGTTTsCTGACsCCuTTGAACTGTATATCGAATATsTTCAusAGsATuTCTTCuCsGATuGG
consensus/70%                      ATACCsTGsTTGTCCAGsAGTTTsCTGACsCCuTTGAACTGTATATCGAATATsTTCAusAGsATuTCTTCuCsGATuGG
```

```
                   cov    pid 11841.       .       .       .       .       .       .       .       . 11920
GA2a.Bcac.CL03T12C61 100.0% 100.0% agagaacgggacgatttctgacagacgcgccaggaactccggacggaaatactgccccatcacctccatgatctgggtgg
GA2b.Bfra.CL05T12C13  99.7%  81.0% ggagaacggaacgatttcagacaggcgtgccagaaactccggacggaaatactgccccatcacctccatgatctgggtgg
GA2c.Bvul.TF05-18     99.8%  82.0% ggagaacggaacgatttcagacaggcgtgccagaaactccggtcggaaatactgtcccattacctccatgatctgggtgg
GA2d.Buni.BIOML-A83   99.2%  69.8% agagaatggaacaatttcagataatctggccagaaactccggacggaaatgtcttcccatcacttccattatctgtgtgg
GA2e.Bsal.DSM.18170   99.7%  79.1% ggagaatgggacaatttcagacaggcggcaaggaattctgggcggaaatactgccccatcacctccattatctggttgg
consensus/100%                     uGAGAAsGGuACuATTTCsGAsAusCssGCsAGuAAsTCsGGsCGGAAATussssCCCATsACsTCCATsATCTGssTGG
consensus/90%                      uGAGAAsGGuACuATTTCsGAsAusCssGCsAGuAAsTCsGGsCGGAAATussssCCCATsACsTCCATsATCTGssTGG
consensus/80%                      uGAGAAsGGuACuATTTCAGACAGuCGsGCCAGuAACTCCGGuCGGAAATACTGsCCCATCACCTCCATsATCTGGGTGG
consensus/70%                      uGAGAAsGGuACuATTTCAGACAGuCGsGCCAGuAACTCCGGuCGGAAATACTGsCCCATCACCTCCATsATCTGGGTGG

                   cov    pid 11921.       .       .       .       .       .       .       .       . 12000
GA2a.Bcac.CL03T12C61 100.0% 100.0% tagccggcacattgcccgattccaactgcttcgtcagccattcgctgcccacattggaggtgaacagcacgatggagttg
GA2b.Bfra.CL05T12C13  99.7%  81.0% tggacggaacattgccggactcaagttgcttggtcagccactcgcttcctacattggatgtgaacagcacgatggagttg
GA2c.Bvul.TF05-18     99.8%  82.0% tgggcggaacattcccggattcaagttgcctggtcagccattcactgcccacgttggaggtaaacagcacgatggagttg
GA2d.Buni.BIOML-A83   99.2%  69.8% tagaaggtacacttcccgattccaactgtttagtcagccactcgctgcccacattggatgtaaacagaataatggaattg
GA2e.Bsal.DSM.18170   99.7%  79.1% tggtcggtacattgcccgattccagctgtcttgtcagccactcgcttcccacattggaggtgaacaggataatggagttc
consensus/100%                     TuGssGGsACAsTsCCsGAsTCsAusTGssTsGTCAGCCAsTCuCTsCCsACuTTGGAsGTuAACAGsAsuATGGAuTTs
consensus/90%                      TuGssGGsACAsTsCCsGAsTCsAusTGssTsGTCAGCCAsTCuCTsCCsACuTTGGAsGTuAACAGsAsuATGGAuTTs
consensus/80%                      TuGsCGGsACATTsCCsGATTCsAusTGssTsGTCAGCCAsTCGCTsCCCACATTGGAsGTuAACAGsAsuATGGAGTTG
consensus/70%                      TuGsCGGsACATTsCCsGATTCsAusTGssTsGTCAGCCAsTCGCTsCCCACATTGGAsGTuAACAGsAsuATGGAGTTG

                   cov    pid 12001.       .       .       .       .       .       .       .       . 12080
GA2a.Bcac.CL03T12C61 100.0% 100.0% gagaagtccccttccttgcccagacggtcatgcagtttcccttcatccatgatttgcaggaaaatatcataaacggacgg
GA2b.Bfra.CL05T12C13  99.7%  81.0% gagaaatccccttccttgcccagacggtcatgcagttttccttcgtccatgatttgcaggaaaatgtcatatacggacgg
GA2c.Bvul.TF05-18     99.8%  82.0% gaaaaatccccctctttgcctaaacggtcatgcagcttcccctcgtccattatttgcaggaaaatgtcatatacggacgg
GA2d.Buni.BIOML-A83   99.2%  69.8% gaaaaatcaccttctttgcctaaacggtcatgaagtttgccctcatccattatttggaggaatatatcatataccgatgg
GA2e.Bsal.DSM.18170   99.7%  79.1% gaaaaatccccttcctttcccagccggtcatgcagtttcccttcgtccatgatttgtaggaaaatgtcatatacggaagg
consensus/100%                     GAuAAuTCsCCsTCsTTsCCsAusCGGTCATGsAGsTTsCCsTCuTCCATsATTTGsAGGAAsATuTCATAsACsGAsGG
consensus/90%                      GAuAAuTCsCCsTCsTTsCCsAusCGGTCATGsAGsTTsCCsTCuTCCATsATTTGsAGGAAsATuTCATAsACsGAsGG
consensus/80%                      GAuAAATCCCCTTCsTTGCCsAuACGGTCATGCAGTTTsCCsTCuTCCATsATTTGsAGGAAAATuTCATATACGGAsGG
consensus/70%                      GAuAAATCCCCTTCsTTGCCsAuACGGTCATGCAGTTTsCCsTCuTCCATsATTTGsAGGAAAATuTCATATACGGAsGG

                   cov    pid 12081.       .       .       .       .       .       .       .       . 12160
GA2a.Bcac.CL03T12C61 100.0% 100.0% atgcgctttctctatctcgtcaaagaggaccaccgcatagggttgctggcggattttattcaccagcataccgccctcct
GA2b.Bfra.CL05T12C13  99.7%  81.0% gtgcgctttctctatttcatcaaagagtacaacggcgtatggctgttgccgtattttattgaccagcatgccgccttcct
GA2c.Bvul.TF05-18     99.8%  82.0% atgcgctttctctatctcgtcaaagaggactacggcgtatggctgttggcgtattttattgactaacataccgccttcct
GA2d.Buni.BIOML-A83   99.2%  69.8% atgagctttctctatttcatcaaaaagaaccactgcataaggctgctgccttatcttgtttaccagcataccgccttctt
GA2e.Bsal.DSM.18170   99.7%  79.1% gtgcgctttctctatctcgtcaaataacacgacggcgtatggttgctggcgtatcttgttgaccagcataccgccttcct
consensus/100%                     uTGsGCTTTCTCTATsTCuTCAAAsAusACsACsGCuTAsGGsTGsTGsCssATsTTuTTsACsAuCATuCCGCCsTCsT
consensus/90%                      uTGsGCTTTCTCTATsTCuTCAAAsAusACsACsGCuTAsGGsTGsTGsCssATsTTuTTsACsAuCATuCCGCCsTCsT
consensus/80%                      uTGCGCTTTCTCTATsTCuTCAAAuAGsACsACsGCuTAsGGsTGsTGsCGTATsTTuTTsACCAGCATACCGCCTTCCT
consensus/70%                      uTGCGCTTTCTCTATsTCuTCAAAuAGsACsACsGCuTAsGGsTGsTGsCGTATsTTuTTsACCAGCATACCGCCTTCCT
```

```
                    cov    pid 12161.       .         .         .         .         .         .         .         . 12240
GA2a.Bcac.CL03T12C61 100.0% 100.0% catagcccacgtatcccggaggcgccccgtaaagcagggcggcggagtgctcctctttgaattcggacatatcaaaacgg
GA2b.Bfra.CL05T12C13  99.7%  81.0% catatcccacatatcccggaggcgctccgtacagcagggcggcggagtgttcttccttgaactcggacatgtcgaagcgg
GA2c.Bvul.TF05-18     99.8%  82.0% catagcccacatatcccggaggtgctccgtaaagcagggctgcggaatgttcctccttgaattcggacatgtcaaagcgg
GA2d.Buni.BIOML-A83   99.2%  69.8% catatcctacatatcccggaggagcaccatacagaagggccgcagagtgttcttccttgaattccgacatatcaaagcgt
GA2e.Bsal.DSM.18170   99.7%  79.1% catagcccacgtatcccggaggcgcaccgtatagcagggcggcagagtgttcttccttgaattcggacatgtcaaagcgg
consensus/100%                     CATAsCCsACuTATCCCGGAGGsGCsCCuTAsAGsAGGGCsGCuGAuTGsTCsTCsTTGAAsTCsGACATuTCuAAuCGs
consensus/90%                      CATAsCCsACuTATCCCGGAGGsGCsCCuTAsAGsAGGGCsGCuGAuTGsTCsTCsTTGAAsTCsGACATuTCuAAuCGs
consensus/80%                      CATAsCCCACuTATCCCGGAGGsGCsCCGTAsAGCAGGGCsGCuGAGTGTTCsTCCTTGAATTCGGACATuTCAAAGCGG
consensus/70%                      CATAsCCCACuTATCCCGGAGGsGCsCCGTAsAGCAGGGCsGCuGAGTGTTCsTCCTTGAATTCGGACATuTCAAAGCGG

                    cov    pid 12241.       .         .         .         .         .         .         .         . 12320
GA2a.Bcac.CL03T12C61 100.0% 100.0% atcatggacttttcatcgttgaacagcgcctccgccaatgccttggcaagttcggtcttgccggtgccggtaggccccag
GA2b.Bfra.CL05T12C13  99.7%  81.0% atcatggatttctcgtcattgaacagggcttccgccagtgccttggcaagttcggtcttgccggtaccggtgggtcccag
GA2c.Bvul.TF05-18     99.8%  82.0% atcatggatttctcgtcattgaacagggcttccgccagtgctttggcgagttcggtcttgccggtaccggtcggtcccaa
GA2d.Buni.BIOML-A83   99.2%  69.8% atcatggattttcatcattgaacaatgtctcagccaaagcctttgccaactctgtttttcctgtaccggtaggtcctaa
GA2e.Bsal.DSM.18170   99.7%  79.1% atcatggacttctcgtcgttgaatagtgtttctgccaatgccttggcgagttcggtctttccggttccggtgggtcctag
consensus/100%                     ATCATGGAsTTsTCuTCuTTGAAsAusGssTCsGCCAusGCsTTsGCsAusTCsGTsTTsCCsGTsCCGGTsGGsCCsAu
consensus/90%                      ATCATGGAsTTsTCuTCuTTGAAsAusGssTCsGCCAusGCsTTsGCsAusTCsGTsTTsCCsGTsCCGGTsGGsCCsAu
consensus/80%                      ATCATGGAsTTsTCuTCuTTGAACAGsGssTCsGCCAuTGCCTTGGCuAGTTCGGTCTTsCCGGTuCCGGTuGGTCCsAu
consensus/70%                      ATCATGGAsTTsTCuTCuTTGAACAGsGssTCsGCCAuTGCCTTGGCuAGTTCGGTCTTsCCGGTuCCGGTuGGTCCsAu

                    cov    pid 12321.       .         .         .         .         .         .         .         . 12400
GA2a.Bcac.CL03T12C61 100.0% 100.0% caggaagaaggagccgatgggctggcccggcttgttcataccgctgcgggattcaagaatggcatcggtcagcgttttca
GA2b.Bfra.CL05T12C13  99.7%  81.0% caggaagaaggagccgatgggctgtcccggcttgttcatgccgctacgggattccaggatggcatccgtcaatgtcttca
GA2c.Bvul.TF05-18     99.8%  82.0% caggaagaaggagccgatgggctgtcccggtttgttcatgccgctacgggattcaaggatggcgtccgtcagcgttttca
GA2d.Buni.BIOML-A83   99.2%  69.8% aaggaagaaggagccgattggctgtcccggcttattcattccgctacgcgattcaaggattgcatccgtaagtgttttca
GA2e.Bsal.DSM.18170   99.7%  79.1% caggaagaaggagccgataggttgccccggcttgttcataccgctgcgggattccagaatagcgtctgtcaatgtcttca
consensus/100%                     sAGGAAGAAGGAGCCGATsGGsTGsCCCGGsTTuTTCATsCCGCTuCGsGATTCsAGuATsGCuTCsGTsAusGTsTTCA
consensus/90%                      sAGGAAGAAGGAGCCGATsGGsTGsCCCGGsTTuTTCATsCCGCTuCGsGATTCsAGuATsGCuTCsGTsAusGTsTTCA
consensus/80%                      CAGGAAGAAGGAGCCGATuGGCTGsCCCGGCTTGTTCATuCCGCTuCGGGATTCsAGuATuGCuTCsGTCAusGTsTTCA
consensus/70%                      CAGGAAGAAGGAGCCGATuGGCTGsCCCGGCTTGTTCATuCCGCTuCGGGATTCsAGuATuGCuTCsGTCAusGTsTTCA

                    cov    pid 12401.       .         .         .         .         .         .         .         . 12480
GA2a.Bcac.CL03T12C61 100.0% 100.0% gcgcctggtcttgaccgacaacccgacggcgcaagtaatcttccatgttgagcaggcgttccttctcttgcgactgtatc
GA2b.Bfra.CL05T12C13  99.7%  81.0% gggcctggtcctgtccgacgacgcgtcgcctcagatagtcttccatattgagtagccgttcctttcttgcgactgtatc
GA2c.Bvul.TF05-18     99.8%  82.0% aagcctggtcctgtccgacgacgcgtcgccgcagatactcttccatattgagtagccgttctttctcctgtgattgtatc
GA2d.Buni.BIOML-A83   99.2%  69.8% aggcctgatcctgcccaacaactcttctacgcaaatagtcctccatattcaacagacgttccttctcctgtgactgtact
GA2e.Bsal.DSM.18170   99.7%  79.1% atgcccggtcttgtccgacgaccctgcgccgcaaatactcctccatattgagcaggcgttcttttcctgggactgtatt
consensus/100%                     usGCCsGuTCsTGsCCuACuACsCssCssCsCAuuTAsTCsTCCATuTTsAusAGsCGTTCsTTsTCsTGsGAsTGTAss
consensus/90%                      usGCCsGuTCsTGsCCuACuACsCssCssCsCAuuTAsTCsTCCATuTTsAusAGsCGTTCsTTsTCsTGsGAsTGTAss
consensus/80%                      usGCCTGGTCsTGsCCGACuACsCssCGsCGCAuATAsTCsTCCATATTGAGsAGsCGTTCsTTsTCsTGsGACTGTATs
consensus/70%                      usGCCTGGTCsTGsCCGACuACsCssCGsCGCAuATAsTCsTCCATATTGAGsAGsCGTTCsTTsTCsTGsGACTGTATs
```

```
                     cov    pid 12481.       .         .         .         .         .         .         .         . 12560
GA2a.Bcac.CL03T12C61 100.0% 100.0% ttgccgataggaatgcccgtcttgctggcaacaacggcggcaacctcataaattcctacctcctctatgttcttttccgc
GA2b.Bfra.CL05T12C13  99.7%  81.0% ttgccgatagggatgcctgtcttgctggcgacaacggcggctacctcatagatacccacctcctctattttcttctctgc
GA2c.Bvul.TF05-18     99.8%  82.0% ttgccgataggaatgcctgtcttgctggcgacaacggcggccacctcgtagatgcctacctcctctattttcttttcggc
GA2d.Buni.BIOML-A83   99.2%  69.8% tttcctattggaataccggttctgcttgctaccaccgcagcgacttcatatatttctacctcttcgattttcttttcggc
GA2e.Bsal.DSM.18170   99.7%  79.1% ttaccgatagggatacctgtcttgctggcaacaacggcagcaacctcatacacttccacctgctctatttctttctcggc
consensus/100%                     TTsCCsATsGGuATuCCsGTssTGCTsGCsACsACsGCuGCsACsTCuTAsAsssCsACCTssTCsATsTssTTsTCsGC
consensus/90%                      TTsCCsATsGGuATuCCsGTssTGCTsGCsACsACsGCuGCsACsTCuTAsAsssCsACCTssTCsATsTssTTsTCsGC
consensus/80%                      TTuCCGATAGGuATuCCsGTCTTGCTGGCuACAACGGCuGCsACCTCATAsATssCsACCTCCTCTATTTTCTTsTCsGC
consensus/70%                      TTuCCGATAGGuATuCCsGTCTTGCTGGCuACAACGGCuGCsACCTCATAsATssCsACCTCCTCTATTTTCTTsTCsGC

                     cov    pid 12561.       .         .         .         .         .         .         .         . 12640
GA2a.Bcac.CL03T12C61 100.0% 100.0% aaatgacaggatggcggccagtgcgctccgcaggtaaaccgccagctcttcatactcctgcaattcatggatatccgcct
GA2b.Bfra.CL05T12C13  99.7%  81.0% aaattggaggaggacgttcaatgcgtttctcagatagccggtcagttcttcatactcctgcaattcatggatgtccgctt
GA2c.Bvul.TF05-18     99.8%  82.0% aaattgaaggatggcgttcaatgcgtttcgcagataagcggcgagttcttcatactcctgtaattcatggatatccgcct
GA2d.Buni.BIOML-A83   99.2%  69.8% aaattcccgcatcaagttcaacgcattcagcagataactcttcaattcagtataatcctgaagttcattcacatccgttt
GA2e.Bsal.DSM.18170   99.7%  79.1% aaaccggagaatggcattcaatgcgtttctcagataagctgccagttcttcatacttctgcaattcttggacattcgtct
consensus/100%                     AAAsssssGsAssusussCAusGCusTsssCAGuTAussssssAusTCsssATAsTsCTGsAuTTCsTssAsuTsCGssT
consensus/90%                      AAAsssssGsAssusussCAusGCusTsssCAGuTAussssssAusTCsssATAsTsCTGsAuTTCsTssAsuTsCGssT
consensus/80%                      AAATsusAGuATGuCGTTCAATGCGTTsCsCAGATAAsCsGsCAGTTCTTCATACTCCTGsAATTCATGGAsATCCGssT
consensus/70%                      AAATsusAGuATGuCGTTCAATGCGTTsCsCAGATAAsCsGsCAGTTCTTCATACTCCTGsAATTCATGGAsATCCGssT

                     cov    pid 12641.       .         .         .         .         .         .         .         . 12720
GA2a.Bcac.CL03T12C61 100.0% 100.0% catcggaaaccacgcccagaaggatggggcttaatttgttgtgcatcaggaaaagcagcgttttcagctcttccaccttt
GA2b.Bfra.CL05T12C13  99.7%  81.0% catcgggtatgactcctaaaaggatggggcttaatttgttgtgcattacgaaaagcagcgttttcaattcttcggtcttt
GA2c.Bvul.TF05-18     99.8%  82.0% cgtcagggatgacacctaaaaggatagggcttaatttgttgtgcattaggaaaagcagcgttttcagctcttccgtcttt
GA2d.Buni.BIOML-A83   99.2%  69.8% catcggttatcactccttgcaagatagggcttagcctattgcgcataacaaacaataaagtctttaagtcctcaactttt
GA2e.Bsal.DSM.18170   99.7%  79.1% cttctggtatgactcccagcaggatagggcttagcttgttgtgcatcagaaatatcaggtccctcagttcttcttccttc
consensus/100%                     CsTCsGssAssACsCCssusAuGATuGGGCTTAussTuTTGsGCATsAsuAAsAssAusssssTsAusTCsTCssssTTs
consensus/90%                      CsTCsGssAssACsCCssusAuGATuGGGCTTAussTuTTGsGCATsAsuAAsAssAusssssTsAusTCsTCssssTTs
consensus/80%                      CuTCuGusATsACsCCsAusAGGATuGGGCTTAusTTGTTGTGCATsAsuAAsAuCAGsGTsTTCAusTCTTCsusCTTT
consensus/70%                      CuTCuGusATsACsCCsAusAGGATuGGGCTTAusTTGTTGTGCATsAsuAAsAuCAGsGTsTTCAusTCTTCsusCTTT

                     cov    pid 12721.       .         .         .         .         .         .         .         . 12800
GA2a.Bcac.CL03T12C61 100.0% 100.0% tcttgaggtgctttttcctccttttctatctcgtccagtcgggcaagcaaggcttccgtatccttctttcctgtttcatt
GA2b.Bfra.CL05T12C13  99.7%  81.0% tcctgaaggtccttttcgtccttttctatttcatccagcccggacagcagggtctccacattcttctttcctgtttcatt
GA2c.Bvul.TF05-18     99.8%  82.0% tcatgggtctctttctctcccttttctatctcatccaaatccggacagcaaagcctccgtgtcctttttccctgtttcatt
GA2d.Buni.BIOML-A83   99.2%  69.8% tcactttcttccgtttcggctttttcaatagtgtctaatccatcaagcaattcttttacgtcctgctgtcctgtttcatt
GA2e.Bsal.DSM.18170   99.7%  79.1% tctacaggctccttttccgcctgctcaatttcgttcaattggccaagcaggatttccgcatcgttcttgcctgtttcatt
consensus/100%                     TCssssssssCssTsTCssCsTssTCsATsssuTssAusssusssAGCAussssTssusuTssTssTssCCTGTTTCATT
consensus/90%                      TCssssssssCssTsTCssCsTssTCsATsssuTssAusssusssAGCAussssTssusuTssTssTssCCTGTTTCATT
consensus/80%                      TCsssuussTCsTTTTCssCCTTTTCsATsTCuTCCAuTCsGsssAGCAuuussTCCusuTCCTTCTTsCCTGTTTCATT
consensus/70%                      TCsssuussTCsTTTTCssCCTTTTCsATsTCuTCCAuTCsGsssAGCAuuussTCCusuTCCTTCTTsCCTGTTTCATT
```

```
                     cov    pid 12801.       .         .         .         .         .         .         .         . 12880
GA2a.Bcac.CL03T12C61 100.0% 100.0% gaccatcttgatggccgaaagtgtcatgtccagcaggccgatggcggagtcgggcaaacggcggtctttcacataccgct
GA2b.Bfra.CL05T12C13  99.7%  81.0% gaccatcttgatggcggagagcgtcatgtccagcagaccgatggcggaatcaggcagacggcggtctttgatataccgtt
GA2c.Bvul.TF05-18     99.8%  82.0% gaccatcttgatggcggaaagcgtcatatccagcagaccgatggcggagtccggcagacggcggtctttcacgtaccgct
GA2d.Buni.BIOML-A83   99.2%  69.8% taccatctttatggcagacaaggtcatatccaaagtccaatggctgagtcaggcaaccgacgttcctttacataacgct
GA2e.Bsal.DSM.18170   99.7%  79.1% caccatttaatggcggagagtgtcatgtccagcagccctatggcagaatccggcaaacggcggtctttcacataccgtt
consensus/100%                     sACCATsTTsATGGCsGAsAusGTCATuTCCAusAGsCCsATGGCsGAuTCsGGCAusCGuCGsTCsTTsAsuTAsCGsT
consensus/90%                      sACCATsTTsATGGCsGAsAusGTCATuTCCAusAGsCCsATGGCsGAuTCsGGCAusCGuCGsTCsTTsAsuTAsCGsT
consensus/80%                      sACCATCTTuATGGCuGAuAGsGTCATuTCCAGCAGsCCuATGGCuGAuTCsGGCAuACGGCGGTCTTTsACATACCGsT
consensus/70%                      sACCATCTTuATGGCuGAuAGsGTCATuTCCAGCAGsCCuATGGCuGAuTCsGGCAuACGGCGGTCTTTsACATACCGsT

                     cov    pid 12881.       .         .         .         .         .         .         .         . 12960
GA2a.Bcac.CL03T12C61 100.0% 100.0% tggcaaggcgcacgcattccgccacggcatcgtcgctgattcccacccggtggtactccacgtatgactgccggacggaa
GA2b.Bfra.CL05T12C13  99.7%  81.0% tggcaagacggacacattcggctacggcttcgttgctgattcttacatgatggtagtccacgtattgtttctgcacggag
GA2c.Bvul.TF05-18     99.8%  82.0% tggcaagacgtacacattcggctacggcatcgtcgcagatctttacctgatggtagtccacatattgtttctgcacggag
GA2d.Buni.BIOML-A83   99.2%  69.8% tggccaaacgcacacattcagccgcggcagtatcgctgataccgacatgatgataatctgtatactgtttctttacacta
GA2e.Bsal.DSM.18170   99.7%  79.1% tggcaaggcgcacacattcggctactgcgtcctcgctgattttacatggtggtagtccacatactgtttctgtacggag
consensus/100%                     TGGCsAuuCGsACuCATTCsGCsuCsGCssssTsGCsGATssssACssGuTGuTAsTCsusuTAssusTsCsssACussu
consensus/90%                      TGGCsAuuCGsACuCATTCsGCsuCsGCssssTsGCsGATssssACssGuTGuTAsTCsusuTAssusTsCsssACussu
consensus/80%                      TGGCAAGuCGsACACATTCuGCsACGGCuTCuTCGCTGATssssACsTGuTGGTAuTCCACuTAsTGTTTCTGsACGGAu
consensus/70%                      TGGCAAGuCGsACACATTCuGCsACGGCuTCuTCGCTGATssssACsTGuTGGTAuTCCACuTAsTGTTTCTGsACGGAu

                     cov    pid 12961.       .         .         .         .         .         .         .         . 13040
GA2a.Bcac.CL03T12C61 100.0% 100.0% tgcagcatctgtatggcggacttcaaatccggctcattgacctgcaccacctcaaaacggcggttgaacgcatggtcagg
GA2b.Bfra.CL05T12C13  99.7%  81.0% tgtagcatctggatagcggactttatgtccggttcgcttacctgtatgacctcaaaacggcggctgaaagcatggtccgg
GA2c.Bvul.TF05-18     99.8%  82.0% tgcagcatctggatggcggactttatgtccggctcgcttatctgcacggtttcgaaacggcggctgaaagcatggtccga
GA2d.Buni.BIOML-A83   99.2%  69.8% tgaagcatctgaatggcagactttaaatcaggctcatttacctgaacgacctcaaatctacgactgaacgcatgatcagg
GA2e.Bsal.DSM.18170   99.7%  79.1% tgcagcatctgaatggcggacttcatgtccggctcattgatttgtatcacctcaaaacggcggttaaaggcatggtccgg
consensus/100%                     TGsAGCATCTGsATuGCuGACTTsAsuTCsGGsTCusTsAssTGsAssussTCuAAsCsuCGusTuAAsGCATGuTCsGu
consensus/90%                      TGsAGCATCTGsATuGCuGACTTsAsuTCsGGsTCusTsAssTGsAssussTCuAAsCsuCGusTuAAsGCATGuTCsGu
consensus/80%                      TGsAGCATCTGuATGGCGGACTTsAsuTCCGGCTCusTsAsCTGsAssACCTCAAAACGGCGGsTGAAsGCATGGTCsGG
consensus/70%                      TGsAGCATCTGuATGGCGGACTTsAsuTCCGGCTCusTsAsCTGsAssACCTCAAAACGGCGGsTGAAsGCATGGTCsGG

                     cov    pid 13041.       .         .         .         .         .         .         .         . 13120
GA2a.Bcac.CL03T12C61 100.0% 100.0% ctctataatcttacggtattcgtcaatggtggtggcgccgataacggtcaggtcgccgtgcgacaattcgggcttcagca
GA2b.Bfra.CL05T12C13  99.7%  81.0% ctcgattatttgcggtattcgtcaatggtggtggcaccgatgacggtcaggtcgccgtgcgacagttcggatttcaata
GA2c.Bvul.TF05-18     99.8%  82.0% ctcaatgatcttgcggtattcgtcaatggtggtggcgccgatgacggtcaggtcgccgtgcgacagttcgggtttcaata
GA2d.Buni.BIOML-A83   99.2%  69.8% ttcgataatcttcctatactcgtctattgtcgtagcaccaattacggtcaaatctccacgtgacaactccggcttcagga
GA2e.Bsal.DSM.18170   99.7%  79.1% ctcgatgatcttacggtattcgtcaatggtagtggcgcctatcacggtcaggtaccgtgcgacaattcgggtttcagca
consensus/100%                     sTCsATsATsTTsCsuTAsTCGTCsATsGTsGTuGCuCCsATsACGGTCAuuTssCCusGsGACAusTCsGusTTCAusA
consensus/90%                      sTCsATsATsTTsCsuTAsTCGTCsATsGTsGTuGCuCCsATsACGGTCAuuTssCCusGsGACAusTCsGusTTCAusA
consensus/80%                      CTCuATuATCTTuCGGTATTCGTCAATGGTuGTGGCuCCuATsACGGTCAGGTCuCCGTGCGACAuTTCGGGsTTCAusA
consensus/70%                      CTCuATuATCTTuCGGTATTCGTCAATGGTuGTGGCuCCuATsACGGTCAGGTCuCCGTGCGACAuTTCGGGsTTCAusA
```

```
                             cov    pid 13121.         .         .         .         .         .         .         .         . 13200
GA2a.Bcac.CL03T12C61 100.0% 100.0%    cattgccggcgccggaattgccctgccggctgtccagcaggatatgaatctcgtcaatgaacaggatggcgttgtcaatg
GA2b.Bfra.CL05T12C13  99.7%  81.0%    catttccagcaccggtatttccttgtcggttgtccagcaatacatggatttcgtcaataaacaggattgcattgtcgata
GA2c.Bvul.TF05-18     99.8%  82.0%    cattccccgcaccggaatttccttgccggttgtccagcagtacgtggatttcgtcaataaacaggatggcattgtcgata
GA2d.Buni.BIOML-A83   99.2%  69.8%    tattacccgctccggaattaccctgtctgctatccagtagaacatgtatttcatctataaacaggatggaattttctata
GA2e.Bsal.DSM.18170   99.7%  79.1%    catttccggcgcccgaattcccttgccggctatccagcaatacatggatttcatcaataaacaggatagcgttgtcgatg
consensus/100%                        sATTsCCsGCsCCsGsATTsCCsTGsCsGsTuTCCAGsAusAsuTGsATsTCuTCsATuAACAGGATsGsuTTsTCsATu
consensus/90%                         sATTsCCsGCsCCsGsATTsCCsTGsCsGsTuTCCAGsAusAsuTGsATsTCuTCsATuAACAGGATsGsuTTsTCsATu
consensus/80%                         CATTsCCsGCuCCGGAATTsCCsTGsCGGsTuTCCAGCAusACATGuATTTCuTCAATAAACAGGATuGCuTTGTCuATu
consensus/70%                         CATTsCCsGCuCCGGAATTsCCsTGsCGGsTuTCCAGCAusACATGuATTTCuTCAATAAACAGGATuGCuTTGTCuATu

                             cov    pid 13201.         .         .         .         .         .         .         .         . 13280
GA2a.Bcac.CL03T12C61 100.0% 100.0%    ccgcgcaactctttcacgatgtttttcaaacggtcctctatctcccccttgtaggtggcgcctgctatgagcgaggcatt
GA2b.Bfra.CL05T12C13  99.7%  81.0%    ttccgcaattctttcaggatgtttttcaggcggtcctcgatttcccccttgtaggtggctcccgcaatcagggaggcgtt
GA2c.Bvul.TF05-18     99.8%  82.0%    ttccgcaattctttcaggatgtttttcaggcggtcctcaatctctcctttataggtggctccggcaatcagggaggcgtt
GA2d.Buni.BIOML-A83   99.2%  69.8%    tggcgaagttccttcaatacattcttcaaacggtcttcgatttctcctttataggtggctccggcaataagcgaagcatt
GA2e.Bsal.DSM.18170   99.7%  79.1%    ccgcgaagttctttcaggatatttttcaggcggtcttcaatttctcctttataggtcgctccggctatgagtgaggcatt
consensus/100%                        sssCGsAusTCsTTCAssAsuTTsTTCAuuCGGTCsTCsATsTCsCCsTTuTAGGTsGCsCCsGCsATsAGsGAuGCuTT
consensus/90%                         sssCGsAusTCsTTCAssAsuTTsTTCAuuCGGTCsTCsATsTCsCCsTTuTAGGTsGCsCCsGCsATsAGsGAuGCuTT
consensus/80%                         sssCGsAuTTCTTTCAuGATuTTTTTCAuuCGGTCsTCuATsTCsCCsTTuTAGGTGGCTCCsGCsATsAGsGAGGCuTT
consensus/70%                         sssCGsAuTTCTTTCAuGATuTTTTTCAuuCGGTCsTCuATsTCsCCsTTuTAGGTGGCTCCsGCsATsAGsGAGGCuTT

                             cov    pid 13281.         .         .         .         .         .         .         .         . 13360
GA2a.Bcac.CL03T12C61 100.0% 100.0%    gtcaagctcaaacacacgggctccctccaggaacgagggcaccttatggttgactatgttccatgcaagcccgtacacaa
GA2b.Bfra.CL05T12C13  99.7%  81.0%    gtccagttcaaatacacgggtttcttccagaaacgagggtactttatggttgactatgtcccaggcaagtccgtaaacca
GA2c.Bvul.TF05-18     99.8%  82.0%    gtctaactcgaagacacgggcttcagccaggaacgaaggcaccttatggttgacaatatcccaggctaacccgtaaacca
GA2d.Buni.BIOML-A83   99.2%  69.8%    gtccaactcgaagatacgggctccttccagaaaagaaggaaccttcttattaacaatatcccatgccagaccataaacca
GA2e.Bsal.DSM.18170   99.7%  79.1%    atccagttcaaagacacgggctccttccaggaaggagggtactttatggttcacgatatcccacgcaagcccataaacta
consensus/100%                        uTCsAusTCuAAsAsACGGGsTsCssCCAGuAAsGAuGGsACsTTsTsuTTsACsATuTsCCAsGCsAusCCuTAsACsA
consensus/90%                         uTCsAusTCuAAsAsACGGGsTsCssCCAGuAAsGAuGGsACsTTsTsuTTsACsATuTsCCAsGCsAusCCuTAsACsA
consensus/80%                         GTCsAusTCuAAsACACGGGCTsCsTCCAGuAAsGAuGGsACsTTATGGTTuACsATuTCCCAsGCsAGsCCuTAAACsA
consensus/70%                         GTCsAusTCuAAsACACGGGCTsCsTCCAGuAAsGAuGGsACsTTATGGTTuACsATuTCCCAsGCsAGsCCuTAAACsA

                             cov    pid 13361.         .         .         .         .         .         .         .         . 13440
GA2a.Bcac.CL03T12C61 100.0% 100.0%    gggctgttttccccaccccggcgtcaccgacaatcagcacgttcgatttgctgcggctgcccagcacctccatgaccatg
GA2b.Bfra.CL05T12C13  99.7%  81.0%    gtgctgtcttttcctactccggcatcaccgacaatcaggacattggacttgctccggcttcccagtatctccatgatcata
GA2c.Bvul.TF05-18     99.8%  82.0%    gtgctgtttttcccacaccggcatcgccgactatcaggacattggacttgctccggcttcccagtatctccatgaccata
GA2d.Buni.BIOML-A83   99.2%  69.8%    aagctgtttttccaactccggcatcacctacaatcaaaacgtttgatttacttcggctacccagcacttccatgaccata
GA2e.Bsal.DSM.18170   99.7%  79.1%    aagccgttttcccgaccccggcatcgccgacaatcagcacattggatttgctgaggctgcctaaaatctccatgatcatg
consensus/100%                        usGCsGTsTTsCCsACsCCGGCuTCuCCsACsATCAusACuTTsGAsTTuCTssGGCTsCCsAusAssTCCATGAsCATu
consensus/90%                         usGCsGTsTTsCCsACsCCGGCuTCuCCsACsATCAusACuTTsGAsTTuCTssGGCTsCCsAusAssTCCATGAsCATu
consensus/80%                         usGCTGTTTTsCCsACsCCGGCATCuCCGACAATCAGsACuTTsGAsTTGCTsCGGCTsCCCAGsAsCTCCATGAsCATu
consensus/70%                         usGCTGTTTTsCCsACsCCGGCATCuCCGACAATCAGsACuTTsGAsTTGCTsCGGCTsCCCAGsAsCTCCATGAsCATu
```

```
                      cov    pid 13441.       .         .         .         .         .         .         .         . 13520
GA2a.Bcac.CL03T12C61 100.0% 100.0%  cggttctcccggtcacggttgatggtcgggtaaagcctgtctgccgcggtggcggtaaggtccacgcaatacttggtgag
GA2b.Bfra.CL05T12C13  99.7%  81.0%  cggtttttcacggtcgcggttgatgaccggatgcagcttgttggccgcatcttcggttagatccacacagtatttgcccaa
GA2c.Bvul.TF05-18     99.8%  82.0%  cggttctcacggtcgcggttgatgaccggatgcagcctgtttgccgcagccgcggtaaggtccacgcaatactttgccag
GA2d.Buni.BIOML-A83   99.2%  69.8%  cgggtctcacggtctctattgataaccggcaataatctttcaggagcggcagccatcagatcaatacaatatttggaaag
GA2e.Bsal.DSM.18170   99.7%  79.1%  cggttctcgcggtcacggttgattaccggatgcaggctgttcgtcgcagcagcggtaaggtccatacagtatttggttaa
consensus/100%                      CGGsTsTCsCGGTCsCsuTTGATsusCGGssusAussTsTssGssGCussssCsuTsAGuTCsAsuCAuTAsTTssssAu
consensus/90%                       CGGsTsTCsCGGTCsCsuTTGATsusCGGssusAussTsTssGssGCussssCsuTsAGuTCsAsuCAuTAsTTssssAu
consensus/80%                       CGGTTCTCuCGGTCuCGGTTGATuACCGGuTusAGsCTGTssGsCGCuGCsGCGGTsAGuTCCAsuCAuTAsTTGGssAu
consensus/70%                       CGGTTCTCuCGGTCuCGGTTGATuACCGGuTusAGsCTGTssGsCGCuGCsGCGGTsAGuTCCAsuCAuTAsTTGGssAu

                      cov    pid 13521.       .         .         .         .         .         .         .         . 13600
GA2a.Bcac.CL03T12C61 100.0% 100.0%  aaaggaagtggaagaggaaacggagctttgaatgaaactctccggcacgttgccggaaactcccgccggttcgttgcctg
GA2b.Bfra.CL05T12C13  99.7%  81.0%  gaaggaggatgaggcggaagtcggggcata---ggacttctccgtggcggcggaaggacatgccggctgcccgttaccta
GA2c.Bvul.TF05-18     99.8%  82.0%  gaaagaggacgaggcggaggccggaacata---ggcatccgccgtggcggcaggagaacataccggctgtccgtttccta
GA2d.Buni.BIOML-A83   99.2%  69.8%  atatgatgaacttcctattcccggaccatagttgtctaataccgaagttgcatgatgatttccacagaaggatttctg
GA2e.Bsal.DSM.18170   99.7%  79.1%  aaaggaagatgaagatgaattggggacaggacaggacttccgtcatgaaaacgggagggttgcctgacggctcattat
consensus/100%                      usAsGAsGsssssssusssssGuussssu...usssssssCCGssusssssussusuAsssssssssssussssTssssss
consensus/90%                       usAsGAsGsssssssusssssGuussssu...usssssssCCGssusssssussusuAsssssssssssussssTssssss
consensus/80%                       uAAuGAuGAsGAuGssGAusssGGusssTu...GussssssCCGssusGusussuGuAssTsCCusssusssuTTssCTu
consensus/70%                       uAAuGAuGAsGAuGssGAusssGGusssTu...GussssssCCGssusGusussuGuAssTsCCusssusssuTTssCTu

                      cov    pid 13601.       .         .         .         .         .         .         .         . 13680
GA2a.Bcac.CL03T12C61 100.0% 100.0%  t---------atatatggaatggatatctttctccaggatagggaaagacctgagctggtcactggaaaaggcgatgccc
GA2b.Bfra.CL05T12C13  99.7%  81.0%  t---------atatatggaatggatatcctttccagaatggggaaagacctgagctggtcgctggaaaaagcgacaccg
GA2c.Bvul.TF05-18     99.8%  82.0%  t---------atatatggaatggatatcctttccagaatggggaaagacctgagctggtcactggaaaaagcgacaccg
GA2d.Buni.BIOML-A83   99.2%  69.8%  tgttattaacatatagggataatatgtctctttcctgaatcggaaaagacctcaactggtcacttgaaaaagcgactcct
GA2e.Bsal.DSM.18170   99.7%  79.1%  t---------atatatggaacggatatcctttccagtatgggaaaggatttgagctggtcgctcgaaaaggcgactccc
consensus/100%                      T.........ATATAsGGAssusATuTCssTsTCCsGsATsGGuAAuGAssTsAuCTGGTCuCTsGAAAAuGCGAssCCs
consensus/90%                       T.........ATATAsGGAssusATuTCssTsTCCsGsATsGGuAAuGAssTsAuCTGGTCuCTsGAAAAuGCGAssCCs
consensus/80%                       T.........ATATATGGAAsGGATATCsTTTTCCAGuATuGGuAAAGACCTGAGCTGGTCuCTsGAAAAuGCGACsCCs
consensus/70%                       T.........ATATATGGAAsGGATATCsTTTTCCAGuATuGGuAAAGACCTGAGCTGGTCuCTsGAAAAuGCGACsCCs

                      cov    pid 13681          .         .         .         .         .         .         .       . 13760
GA2a.Bcac.CL03T12C61 100.0% 100.0%  ggtttcgtcagtgccagcaacacgcagatgggattgatgtcaaacagccccatctgtatccgagcgtcatcggcggcacg
GA2b.Bfra.CL05T12C13  99.7%  81.0%  ggctttgccagtgccaatagcacacagatgggattgatgtcgaacagcccgaactggattcgtgcatcgtctgcggcacg
GA2c.Bvul.TF05-18     99.8%  82.0%  ggctttgtcagtgccaatagcacgcagatgggattgatgtcgaacagcccgaacaggattcgtgcgtcatcggcggctcg
GA2d.Buni.BIOML-A83   99.2%  69.8%  ggtttaccaaggcaataagaacacagattggatttatatcaaataatccaagacgaatacgggcatcatccgcagcccg
GA2e.Bsal.DSM.18170   99.7%  79.1%  ggctttgccagggcagccaatacacatatagggttaatatcgaacagccctagctctatgcgcacatcatcggcagcacg
consensus/100%                      GGsTTsusCAusGCsussAusACuCAsATsGGuTTsATuTCuAAsAusCCsAsssssATsCGsuCuTCuTCsGCuGCsCG
consensus/90%                       GGsTTsusCAusGCsussAusACuCAsATsGGuTTsATuTCuAAsAusCCsAsssssATsCGsuCuTCuTCsGCuGCsCG
consensus/80%                       GGsTTTGsCAGsGCsAssAusACuCAGATuGGATTuATuTCuAACAGCCCsAuCsGsATsCGsGCuTCATCsGCuGCsCG
consensus/70%                       GGsTTTGsCAGsGCsAssAusACuCAGATuGGATTuATuTCuAACAGCCCsAuCsGsATsCGsGCuTCATCsGCuGCsCG
```

```
                   cov    pid 13761.       .         .         .         .         .         .         .         . 13840
GA2a.Bcac.CL03T12C61 100.0% 100.0%  gaaaacggcgtcgatcttctccgacggcttgatttcggcgaaccgggtggatttcggacattgttcaatatggacatccg
GA2b.Bfra.CL05T12C13  99.7%  81.0%  gaaaacggcgtcaatccgttccgaaggcttgatttcggacaactgggtggatttcgggcattgttcgatatacacgtccg
GA2c.Bvul.TF05-18     99.8%  82.0%  gaaaacggagtctatctgctccgatggcttgatctcggacaaccgggtggatttcgggcattgctcgatgtacacgtccg
GA2d.Buni.BIOML-A83   99.2%  69.8%  aaatataaaatcaattttttctgaaggtttaatctcggaaagttgggtagacttgggacattgttcaatatatacatcag
GA2e.Bsal.DSM.18170   99.7%  79.1%  gaataggctgccaatcttttccgaaggcttgatttctgacagctgggtagatttcgggcattgctcgatatgaacatccg
consensus/100%                      uAAsAsussusCsATssssTCsGAsGGsTTuATsTCsGssAussGGGTuGAsTTsGGuCATTGsTCuATuTusACuTCsG
consensus/90%                       uAAsAsussusCsATssssTCsGAsGGsTTuATsTCsGssAussGGGTuGAsTTsGGuCATTGsTCuATuTusACuTCsG
consensus/80%                       GAAsAsGusGTCuATCTssTCCGAsGGCTTGATsTCGGAsAuCsGGGTuGATTTCGGuCATTGsTCuATATusACuTCCG
consensus/70%                       GAAsAsGusGTCuATCTssTCCGAsGGCTTGATsTCGGAsAuCsGGGTuGATTTCGGuCATTGsTCuATATusACuTCCG

                   cov    pid 13841.       .         .         .         .         .         .         .         . 13920
GA2a.Bcac.CL03T12C61 100.0% 100.0%  cccattcctccagataccccaaatccttacctatacttattataaaatcccttagccctacatctttatgaagtagagcc
GA2b.Bfra.CL05T12C13  99.7%  81.0%  cccattcctccagatagcctaaatccttacctatacttattataaaatctcttaaccctacatctttatgaagtagagcc
GA2c.Bvul.TF05-18     99.8%  82.0%  cccactcctccagatagcctaaatccttacctatacttattataaaatctcgtagccctacatccttatgaagtagagcc
GA2d.Buni.BIOML-A83   99.2%  69.8%  cccactcttccagatagcccaaatcctttccaatactaataataaaatctcttagccccacgtccttatggagtagagcc
GA2e.Bsal.DSM.18170   99.7%  79.1%  cccattcttccagatagcccaaatccttacctatacttaaaataaaatctcttagccctacttccttatgaagtaaggct
consensus/100%                      CCCAsTCsTCCAGATAsCCsAAATCCTTsCCsATACTsAssATAAAATCsCsTAuCCCsACsTCsTTATGuAGTAuuGCs
consensus/90%                       CCCAsTCsTCCAGATAsCCsAAATCCTTsCCsATACTsAssATAAAATCsCsTAuCCCsACsTCsTTATGuAGTAuuGCs
consensus/80%                       CCCAsTCsTCCAGATAGCCsAAATCCTTACCTATACTTATsATAAAATCTCTTAGCCCTACuTCsTTATGAAGTAGAGCC
consensus/70%                       CCCAsTCsTCCAGATAGCCsAAATCCTTACCTATACTTATsATAAAATCTCTTAGCCCTACuTCsTTATGAAGTAGAGCC

                   cov    pid 13921.       .         .         .         .         .         .         .         . 14000
GA2a.Bcac.CL03T12C61 100.0% 100.0%  ttcaacaaatgcgagggagtgtaacactcgttcgcattttcacgagccactgcaagtgctatgttaagcgcaatttttac
GA2b.Bfra.CL05T12C13  99.7%  81.0%  ttcaacaaatgcgagggtgtataacactcgttcgcattttcacgagccactgcaagtgctatgttaagcgcaatttttac
GA2c.Bvul.TF05-18     99.8%  82.0%  ttcaacaaatgcgagggagtataacactcgttcgcattttcacgagccactgcaagtgctatgttaagcgcaatttttac
GA2d.Buni.BIOML-A83   99.2%  69.8%  ttcaataaatgcgagggcatataatactcattcgcattctcacgagccactgcctgtgctatgttaagcgcaatttttac
GA2e.Bsal.DSM.18170   99.7%  79.1%  ttcaataaatgcgaaggggtataatactcatttgcattttcacgggccactgcctgtgctatgttaagcgcaatttttat
consensus/100%                      TTCAAsAAATGCGAuGGsuTuTAAsACTCuTTsGCATTsTCACGuGCCACTGCssGTGCTATGTTAAGCGCAATTTTTAs
consensus/90%                       TTCAAsAAATGCGAuGGsuTuTAAsACTCuTTsGCATTsTCACGuGCCACTGCssGTGCTATGTTAAGCGCAATTTTTAs
consensus/80%                       TTCAAsAAATGCGAGGGsGTATAAsACTCuTTCGCATTTTCACGAGCCACTGCssGTGCTATGTTAAGCGCAATTTTTAC
consensus/70%                       TTCAAsAAATGCGAGGGsGTATAAsACTCuTTCGCATTTTCACGAGCCACTGCssGTGCTATGTTAAGCGCAATTTTTAC

                   cov    pid 14001.       .         .         .         .         .         .         .         . 14080
GA2a.Bcac.CL03T12C61 100.0% 100.0%  agaatcatcaagattaatgaaaccaactcctgtatccataatcaat-aagatttaaaaattgcgcaggagaacattgcaa
GA2b.Bfra.CL05T12C13  99.7%  81.0%  agaatcatcaagattgatgaaaccaactcctgtttccataatcg-t-aagatttaaaaattgcgcaggagaacattgcaa
GA2c.Bvul.TF05-18     99.8%  82.0%  agaatcatcaagattaatgaaaccaactcctgtatccataatcatt-aagatttaaaaattgcgcaggagaacattgcaa
GA2d.Buni.BIOML-A83   99.2%  69.8%  agaatcatcaagattaatgaaactaactcctgtatccataatcaataaagatttaaaaattgcgcaggagaacaatgcaa
GA2e.Bsal.DSM.18170   99.7%  79.1%  agaatcatcaagattaatgaaaccaactcctgtatccataatcaat-aagattt-aaaattgcgcaggagaacaccgcaa
consensus/100%                      AGAATCATCAAGATTuATGAAACsAACTCCTGTsTCCATAATCu.T.AAGATTT.AAAATTGCGCAGGAGAACAssGCAA
consensus/90%                       AGAATCATCAAGATTuATGAAACsAACTCCTGTsTCCATAATCu.T.AAGATTT.AAAATTGCGCAGGAGAACAssGCAA
consensus/80%                       AGAATCATCAAGATTAATGAAACCAACTCCTGTATCCATAATCAsT.AAGATTTAAAAATTGCGCAGGAGAACAsTGCAA
consensus/70%                       AGAATCATCAAGATTAATGAAACCAACTCCTGTATCCATAATCAsT.AAGATTTAAAAATTGCGCAGGAGAACAsTGCAA
```

```
                         cov    pid 14081.       .         .         .         .         .         .         .         . 14160
GA2a.Bcac.CL03T12C61 100.0% 100.0%   atataaggcaaaaaaataagcccgccacttttcagggcagatttttttatgttaacgaacataatttttgaaaaaaccaa
GA2b.Bfra.CL05T12C13  99.7%  81.0%   atataaggcaaaaaaaataattcggctattgttggcagcgttttttttatgcttatgggcatattttcggtaaaaacaag
GA2c.Bvul.TF05-18     99.8%  82.0%   atataaggcaaaaaaaataatccagctatttttgaccgttgtttttttatgcttgtgagcatgtttccaacaaaaacaag
GA2d.Buni.BIOML-A83   99.2%  69.8%   atataaaagtaaaaaattaaattcaatgcattatccgacaatttttttatgctcatgagcatattgtacgaaaaaaacat
GA2e.Bsal.DSM.18170   99.7%  79.1%   atataaggcaaaaaacgaaaatccgctactttttag-agcgtttttttatgcctgagggcattttttcgagaaaaatc--
consensus/100%                       ATATAAuussAAAAAsssAusssssssssssTsss.sssssTTTTTTTATGsssusGuuCATssTssssusAAAAAss..
consensus/90%                        ATATAAuussAAAAAsssAusssssssssssTsss.sssssTTTTTTTATGsssusGuuCATssTssssusAAAAAss..
consensus/80%                        ATATAAGGCAAAAAAAssAAssCsuCTusTTTsuusuususTTTTTTTATGCTsusGuGCATuTTTTssusAAAAAssAs
consensus/70%                        ATATAAGGCAAAAAAAssAAssCsuCTusTTTsuusuususTTTTTTTATGCTsusGuGCATuTTTTssusAAAAAssAs

                         cov    pid 14161.       .         .         .         .         .         .         .         . 14240
GA2a.Bcac.CL03T12C61 100.0% 100.0%   aaaatatcgcaaaaaatttcccaaaaaacttttgtaattttcagaaagctccatatattagcccccggaaattgaaagcc
GA2b.Bfra.CL05T12C13  99.7%  81.0%   aaaaaatcatcaaaagtt-----------ctttgtagttttcagaaaatgccatatattgggcgca-gaaattgaaggac
GA2c.Bvul.TF05-18     99.8%  82.0%   aaaaaatcctcgaaagtt-----------ctttgtagttttcagaaaatgccatatattgggcgca-gaaattgaaggac
GA2d.Buni.BIOML-A83   99.2%  69.8%   ccgatttt---------------------ctttgcaatattcgaaaaaaagcatacatta--cgccagaaatctcaaaga
GA2e.Bsal.DSM.18170   99.7%  79.1%   ----------gaaaatttttcctaaaacgctttgttatttcagcaaaatgccataaattgggcacc-gaaattagaagtc
consensus/100%                       .............................sTTTGssuTsTssusAAAusssCATAsATTu..CsCs.GAAATsssAuuss
consensus/90%                        .............................sTTTGssuTsTssusAAAusssCATAsATTu..CsCs.GAAATsssAuuss
consensus/80%                        ssuAssTs..suAAAsTT...........CTTTGTAuTTTTCuuAAAAsuCCATAsATTuGsCuCs.GAAATTuuAuGsC
consensus/70%                        ssuAssTs..suAAAsTT...........CTTTGTAuTTTTCuuAAAAsuCCATAsATTuGsCuCs.GAAATTuuAuGsC

                         cov    pid 14241.       .         .         .         .         .         .         .         . 14320
GA2a.Bcac.CL03T12C61 100.0% 100.0%   aaaggcggggaggcgccgaaccgccggtagcc--ggaaattccgtaatcaacttttttacaaactttaaaactttgagaa
GA2b.Bfra.CL05T12C13  99.7%  81.0%   aaagataggactgcagcaggcctcctttgacc--ggatatttcaaaatcaacttatttttttaa-tttaaaactttgagaa
GA2c.Bvul.TF05-18     99.8%  82.0%   aaagacagggtaacaacagaccgcctctgacc--ggatatttcaaaatcaatttattgtttaa-tttaaaactttgagac
GA2d.Buni.BIOML-A83   99.2%  69.8%   aaaaggaaaatggcaacaggattctgattaccttgaggatttcaaagtgaaccaatttattaa-cttaaaactttaataa
GA2e.Bsal.DSM.18170   99.7%  79.1%   aaaaatgacggggcaaccggtcatggatgagg--gataatttcagaattaacatttttattaa-cttaaaacattgagaa
consensus/100%                       AAAuusuusussuCusCsuusssssssTsuss..GussATTsCusAuTsAAssssTTssssAA.sTTAAAACsTTuAsAs
consensus/90%                        AAAuusuusussuCusCsuusssssssTsuss..GussATTsCusAuTsAAssssTTssssAA.sTTAAAACsTTuAsAs
consensus/80%                        AAAuusuuuusuGCAuCuGusCsCsssTuACC..GuusATTTCAuAATsAACsTsTTTsTTAA.sTTAAAACTTTGAGAA
consensus/70%                        AAAuusuuuusuGCAuCuGusCsCsssTuACC..GuusATTTCAuAATsAACsTsTTTsTTAA.sTTAAAACTTTGAGAA

                         cov    pid 14321.       .         .         .         .         .         .         .         . 14400
GA2a.Bcac.CL03T12C61 100.0% 100.0%   tatggcatttaaagcgtcatttaagttttcagactcaagagagtttgatgtattgagatgggatgtcaagttcaatcgtg
GA2b.Bfra.CL05T12C13  99.7%  81.0%   tatggcatttaaagcgtcatttaagttttcggactcaagagagtttgatgtactgacctggagagttaagttcaatcgtg
GA2c.Bvul.TF05-18     99.8%  82.0%   tatggcatttaaagcgtcatttaagttctcggactcaagagagtttgatgtattgaactggagagtcaagttcaatcgtg
GA2d.Buni.BIOML-A83   99.2%  69.8%   catggcatttaaagcgtcatttaagttttcagactctagagagtttgatgtattgagatgggatgtcaagtttaaccgtg
GA2e.Bsal.DSM.18170   99.7%  79.1%   tatggcatttaaagcgtcatttaagttctcggactcgaaagagtttgatgtgttgagatgggatgtcaagttcaaccgtg
consensus/100%                       sATGGCATTTAAAGCGTCATTTAAGTTsTCuGACTCsAuAGAGTTTGATGTusTGAssTGGuusGTsAAGTTsAAsCGTG
consensus/90%                        sATGGCATTTAAAGCGTCATTTAAGTTsTCuGACTCsAuAGAGTTTGATGTusTGAssTGGuusGTsAAGTTsAAsCGTG
consensus/80%                        TATGGCATTTAAAGCGTCATTTAAGTTsTCuGACTCuAGAGAGTTTGATGTATTGAusTGGuusGTCAAGTTCAAsCGTG
consensus/70%                        TATGGCATTTAAAGCGTCATTTAAGTTsTCuGACTCuAGAGAGTTTGATGTATTGAusTGGuusGTCAAGTTCAAsCGTG
```

```
                    cov    pid 14401.                                                                          . 14480
GA2a.Bcac.CL03T12C61 100.0% 100.0%  atgtggaccccaaaggccgtccggcatctgacatctatggtggtaaaatctatgtggaggttgaatctactcccgatacc
GA2b.Bfra.CL05T12C13  99.7%  81.0%  atgtggaccccaaaggccgtccggcatctgacatttatggtggcacaatctatgtggaaatcgaatcaactcccgatacc
GA2c.Bvul.TF05-18     99.8%  82.0%  atgtggaccccaaaggtcgtccggcatctgacatttatggtggcacaatctatgtggaagtcgaatcgactcccgatacc
GA2d.Buni.BIOML-A83   99.2%  69.8%  atgtagatcctaaaggacgtccggcatctgacatttatggaggtaccattaacgtggaagtcgaatctactcctgacaca
GA2e.Bsal.DSM.18170   99.7%  79.1%  atgtggatcctaagggtcgtccggcatctgacatctatggtggtacaattaatgtcgaggttgaatcaactccggatacg
consensus/100%                      ATGTuGAsCCsAAuGGsCGTCCGGCATCTGACATsTATGGsGGsAssATssAsGTsGAuuTsGAATCsACTCCsGAsACs
consensus/90%                       ATGTuGAsCCsAAuGGsCGTCCGGCATCTGACATsTATGGsGGsAssATssAsGTsGAuuTsGAATCsACTCCsGAsACs
consensus/80%                       ATGTGGAsCCsAAAGGsCGTCCGGCATCTGACATsTATGGTGGsACAATssATGTGGAuGTsGAATCsACTCCsGATACs
consensus/70%                       ATGTGGAsCCsAAAGGsCGTCCGGCATCTGACATsTATGGTGGsACAATssATGTGGAuGTsGAATCsACTCCsGATACs

                    cov    pid 14481.                                                                          . 14560
GA2a.Bcac.CL03T12C61 100.0% 100.0%  attgtgttggacaagatgttcaaacagtatcagcccgtaaacggaagtatcgtcttcaagaaagcggatgaggatgccaa
GA2b.Bfra.CL05T12C13  99.7%  81.0%  attgtgctggataagatgttcaagcagtaccagcccgtaaacggaaacatcgtcttcaagaaagcggatgaagatgccaa
GA2c.Bvul.TF05-18     99.8%  82.0%  attgtgctggataaaatgttcaagcagtaccagcctgtaaacggaaacatcgtcttcaagaaagcggatgaggatgccaa
GA2d.Buni.BIOML-A83   99.2%  69.8%  attgtattggacaagatgttcaaacagtaccagccagtaaacggaaacatcgtcttcaagaaagctgatgaagatgctaa
GA2e.Bsal.DSM.18170   99.7%  79.1%  attgtactggacaaaatgttcaagcagtatcagcctgtaaacggaaacatcgtcttcaagaaagcagacgaggatgccaa
consensus/100%                      ATTGTusTGGAsAAuATGTTCAAuCAGTAsCAGCCsGTAAACGGAAusATCGTCTTCAAGAAAGCsGAsGAuGATGCsAA
consensus/90%                       ATTGTusTGGAsAAuATGTTCAAuCAGTAsCAGCCsGTAAACGGAAusATCGTCTTCAAGAAAGCsGAsGAuGATGCsAA
consensus/80%                       ATTGTusTGGAsAAuATGTTCAAuCAGTAsCAGCCsGTAAACGGAAACATCGTCTTCAAGAAAGCuGATGAuGATGCCAA
consensus/70%                       ATTGTusTGGAsAAuATGTTCAAuCAGTAsCAGCCsGTAAACGGAAACATCGTCTTCAAGAAAGCuGATGAuGATGCCAA

                    cov    pid 14561.                                                                          . 14640
GA2a.Bcac.CL03T12C61 100.0% 100.0%  gatgaaggaactggtatttgagaacggttacgtggtggaatacgaggaggcactgaatgtggccaacccctatccgatga
GA2b.Bfra.CL05T12C13  99.7%  81.0%  aatgaaggaattggtgttcgaaaatggttatgtggtggattacgaagaggcactgaatgtggctaacccttatccgatga
GA2c.Bvul.TF05-18     99.8%  82.0%  aatgaaggaactggtgttcgaaaacggttacgtggtagagtatgaggaggcactgaatgtggctaacgcctatccgatga
GA2d.Buni.BIOML-A83   99.2%  69.8%  aatgaaagaactggtatttgaaaacggttatgtcattaagtacgaggaggcactaagtgtagctaatgattatcctatga
GA2e.Bsal.DSM.18170   99.7%  79.1%  gatgaaggaactggtatttgagaacgggtatattgtgaagtacgaggaggcactgaatgtagcgaactcatacccaatga
consensus/100%                      uATGAAuGAAsTGGTuTTsGAuAAsGGsTAsuTsuTsuAsTAsGAuGAGGCACTuAuTGTuGCsAAssssTAsCCsATGA
consensus/90%                       uATGAAuGAAsTGGTuTTsGAuAAsGGsTAsuTsuTsuAsTAsGAuGAGGCACTuAuTGTuGCsAAssssTAsCCsATGA
consensus/80%                       uATGAAGGAACTGGTuTTsGAuAACGGTTAsGTsGTuuAuTACGAGGAGGCACTGAATGTuGCsAACsCsTATCCuATGA
consensus/70%                       uATGAAGGAACTGGTuTTsGAuAACGGTTAsGTsGTuuAuTACGAGGAGGCACTGAATGTuGCsAACsCsTATCCuATGA

                    cov    pid 14641.                                                                          . 14720
GA2a.Bcac.CL03T12C61 100.0% 100.0%  caatcaagttcgccatttccgcacagacagtgaagatggacgaggctacatttgtacaggattggccggaaaacaactaa
GA2b.Bfra.CL05T12C13  99.7%  81.0%  caatcaagttcgccatttctgcacagaccgtgaagatggacgaggctacatttgtacaggattggccggaaaacaactaa
GA2c.Bvul.TF05-18     99.8%  82.0%  cagtcaagttcgccatttccgcacagaccgtgaaaatggacgaggctacgtttacacaggattggccggaaaacaactaa
GA2d.Buni.BIOML-A83   99.2%  69.8%  caatcaagttcactatttctgctcagacagtaaagatgaacgaagttacatttacacaagactggccggaaaataactaa
GA2e.Bsal.DSM.18170   99.7%  79.1%  caatcaaattctccatctctgctcaaacagtgaagatggctgatgctacatttgtacaagactggccggaaaacaactaa
consensus/100%                      CAuTCAAuTTCsCsATsTCsGCsCAuACsGTuAAuATGussGAsGsTACuTTTusACAuGAsTGGCCGGAAAAsAACTAA
consensus/90%                       CAuTCAAuTTCsCsATsTCsGCsCAuACsGTuAAuATGussGAsGsTACuTTTusACAuGAsTGGCCGGAAAAsAACTAA
consensus/80%                       CAATCAAGTTCuCCATTTCsGCsCAGACsGTGAAGATGGACGAuGCTACATTTusACAuGAsTGGCCGGAAAACAACTAA
consensus/70%                       CAATCAAGTTCuCCATTTCsGCsCAGACsGTGAAGATGGACGAuGCTACATTTusACAuGAsTGGCCGGAAAACAACTAA
```

```
                           cov    pid 14721.         .         .         .         .         .         .         .         . 14800
GA2a.Bcac.CL03T12C61 100.0% 100.0%   cggttgttttctaatcaaagtttttaaggtgccggaatataccaattccggttatttcggcaccaaaaaacataccgact
GA2b.Bfra.CL05T12C13  99.7%  81.0%   tggttgtttcctaatcaaag-ttttaaggtgccggaatataccaattccggttatttcggcaccaaaaaacataccgact
GA2c.Bvul.TF05-18     99.8%  82.0%   cggttgtttcctaatcaaag-ttttaaggtgccggaatataccaattccggttatttcggcaccaaaaaacataccgact
GA2d.Buni.BIOML-A83   99.2%  69.8%   tggttagttttgggttaaag-ttttgaggtgccgggatataccaattccggttattttggcacctcaaaacataccgact
GA2e.Bsal.DSM.18170   99.7%  79.1%   ccgttgttt-ctagtcaaat-tcttggagtgccggaatataccaattccggttatttcggcactcaaaaacataccgacc
consensus/100%                       ssGTTusTT.ssuuTsAAAs.TsTTuuuGTGCCGGuATATACCAATTCCGGTTATTTsGGCACsssAAAACATACCGACs
consensus/90%                        ssGTTusTT.ssuuTsAAAs.TsTTuuuGTGCCGGuATATACCAATTCCGGTTATTTsGGCACsssAAAACATACCGACs
consensus/80%                        sGGTTGTTTsCTAuTCAAAG.TTTTuAGGTGCCGGAATATACCAATTCCGGTTATTTCGGCACCsAAAAACATACCGACT
consensus/70%                        sGGTTGTTTsCTAuTCAAAG.TTTTuAGGTGCCGGAATATACCAATTCCGGTTATTTCGGCACCsAAAAACATACCGACT

                           cov    pid 14801.         .         .         .         .         .         .         .         . 14880
GA2a.Bcac.CL03T12C61 100.0% 100.0%   ggtcggattgattaattaattttcatagcaaa--agatagtatgggagcaagttttctgaatttggtaactaccaaggtg
GA2b.Bfra.CL05T12C13  99.7%  81.0%   agtcggatt-atttatttaaaatcatagttta--agatagtatgggagcaagttttctgaatttggtggctactaaagta
GA2c.Bvul.TF05-18     99.8%  82.0%   ggtcggttt-aataattgaaattcatagttta--agatagtatgggagcaagttttctgaatttggtggctactaaagtg
GA2d.Buni.BIOML-A83   99.2%  69.8%   agtcggtttatttatgtaataaccttatagaaattaataatatgggagcaagttttctgaatctggtggctacaaaggtg
GA2e.Bsal.DSM.18170   99.7%  79.1%   agtcggattaataaatgggaattaaatagatattaataagtatgggagcaagttttctgaatttggtagccaccaaggta
consensus/100%                       uGTCGGsTT.sssssssusssssssssssssA..sussAuTATGGGAGCAAGTTTTCTGAATsTGGTuuCsACsAAuGTu
consensus/90%                        uGTCGGsTT.sssssssusssssssssssssA..sussAuTATGGGAGCAAGTTTTCTGAATsTGGTuuCsACsAAuGTu
consensus/80%                        uGTCGGsTT.ATTAATTuAsAsTCATAusssA..AuATAGTATGGGAGCAAGTTTTCTGAATTTGGTuGCTACsAAuGTu
consensus/70%                        uGTCGGsTT.ATTAATTuAsAsTCATAusssA..AuATAGTATGGGAGCAAGTTTTCTGAATTTGGTuGCTACsAAuGTu

                           cov    pid 14881.         .         .         .         .         .         .         .         . 14960
GA2a.Bcac.CL03T12C61 100.0% 100.0%   acagtgaaaggagaggaacagaagttcgtttcactgcggcttcatcagcggtttaaccggcatcacgcctttactgtaat
GA2b.Bfra.CL05T12C13  99.7%  81.0%   accgtgaaaggagaagaacagaaattcgtttcactgcatctccgtcagggtttcaaccggcatcacaccttcactgtgat
GA2c.Bvul.TF05-18     99.8%  82.0%   accgtgaaaggagaagagcagaagttcgtttcattgtgtctccgtcagggttttaaccggcatcacacctttaccgtggt
GA2d.Buni.BIOML-A83   99.2%  69.8%   acgataaaaggagaggaacagaagttcgtttcccttcgtctccatcagtctttcaacaaacatcacgattttacagtgat
GA2e.Bsal.DSM.18170   99.7%  79.1%   accgtgaagggggaagaacaaaaattcgtttctttacgccttcatcaaggtttcaaccggcatcacacgtttactataat
consensus/100%                       ACsuTuAAuGGuGAuGAuCAuAAuTTCGTTTCssTssusCTsCuTCAusssTTsAACsuuCATCACussTTsACsuTuuT
consensus/90%                        ACsuTuAAuGGuGAuGAuCAuAAuTTCGTTTCssTssusCTsCuTCAusssTTsAACsuuCATCACussTTsACsuTuuT
consensus/80%                        ACsGTGAAAGGAGAuGAACAGAAuTTCGTTTCssTuCGsCTsCuTCAGsGTTTsAACCGGCATCACuCsTTTACsGTuAT
consensus/70%                        ACsGTGAAAGGAGAuGAACAGAAuTTCGTTTCssTuCGsCTsCuTCAGsGTTTsAACCGGCATCACuCsTTTACsGTuAT

                           cov    pid 14961.         .         .         .         .         .         .         .         . 15040
GA2a.Bcac.CL03T12C61 100.0% 100.0%   tgtgaattacctctcgcccggcaatacgttcaagcagacacctgaaaaattcatcaattacatcggggagactgtcagca
GA2b.Bfra.CL05T12C13  99.7%  81.0%   ggtgaattatctgtcgcctaacaatacgttccaacagaccccggaaaaattcatcggctacatcggggagactgccagca
GA2c.Bvul.TF05-18     99.8%  82.0%   ggtgaattatctgtcgcctaacaatacgttccaacaaaccccggaaaaattcatcggttacatcggggaaaccgccagca
GA2d.Buni.BIOML-A83   99.2%  69.8%   tgtcaactatctttcaccaaacaataccttccagcagactccggagaaattcatcaatttcattggagagactatcacga
GA2e.Bsal.DSM.18170   99.7%  79.1%   tgtaaactatctgtcgcctaacaacacctttcagcagactccggagaaattcatcaattacattggggagactgccaata
consensus/100%                       sGTsAAsTAsCTsTCuCCsuuCAAsACsTTssAuCAuACsCCsGAuAAATTCATCuusTsCATsGGuGAuACsusCAssA
consensus/90%                        sGTsAAsTAsCTsTCuCCsuuCAAsACsTTssAuCAuACsCCsGAuAAATTCATCuusTsCATsGGuGAuACsusCAssA
consensus/80%                        sGTuAAsTATCTsTCGCCsAACAATACsTTCCAuCAGACsCCGGAuAAATTCATCuuTTACATsGGGGAGACTGsCAusA
consensus/70%                        sGTuAAsTATCTsTCGCCsAACAATACsTTCCAuCAGACsCCGGAuAAATTCATCuuTTACATsGGGGAGACTGsCAusA
```

```
                         cov    pid 15041.       .         .         .         .         .         .         .         . 15120
GA2a.Bcac.CL03T12C61 100.0% 100.0%  tctcgttcgcccacaagcagacgggcgaaagttatgacttcgaaggggttgtcacacaggtggaaatggtcggaagcatg
GA2b.Bfra.CL05T12C13  99.7%  81.0%  tttcatttgtccataggcagacgggtgagagttatgacttcgagggaatcatcactcaagtggaaatggttggaagcatg
GA2c.Bvul.TF05-18     99.8%  82.0%  tttcgtttgcccacaagcagacgggtgaaagttatgatttcgagggtgtcatcactcgggtggaaatggttggaagcatg
GA2d.Buni.BIOML-A83   99.2%  69.8%  tttcgtttacccatcggcagacaagtgacagttatgtatttgaaggtgtggtaactaaggttgaaatgattggaagtgaa
GA2e.Bsal.DSM.18170   99.7%  79.1%  tttcattcgtccacaagcagaccggagaaagctatgatttcgaagggattatcaatcaggtggaaatgattggaagtgtg
consensus/100%                      TsTCuTTsusCCAssuGCAGACsuGsGAsAGsTATGssTTsGAuGGsuTsuTsAssuuGTsGAAATGuTsGGAAGsusu
consensus/90%                       TsTCuTTsusCCAssuGCAGACsuGsGAsAGsTATGssTTsGAuGGsuTsuTsAssuuGTsGAAATGuTsGGAAGsusu
consensus/80%                       TTTCuTTsGsCCAsAuGCAGACuGGsGAuAGTTATGAsTTCGAuGGsuTsuTCACTCAGGTGGAAATGuTTGGAAGsuTG
consensus/70%                       TTTCuTTsGsCCAsAuGCAGACuGGsGAuAGTTATGAsTTCGAuGGsuTsuTCACTCAGGTGGAAATGuTTGGAAGsuTG

                         cov    pid 15121.       .         .         .         .         .         .         .         . 15200
GA2a.Bcac.CL03T12C61 100.0% 100.0%  ggtgaggcgggcggagtggccattcacggagcaagccccacgatattgtatgagaacaacggaacgcttgactcgtggat
GA2b.Bfra.CL05T12C13  99.7%  81.0%  ggcgagacaggcggagtggccattcacggaacaagccctacgatactgtatgagaacaacagaacactcgattcttggat
GA2c.Bvul.TF05-18     99.8%  82.0%  ggtgagacaggcggagtggccattcatggagcaagccctacgatactctatgagaacaacaggacactcgactcatggat
GA2d.Buni.BIOML-A83   99.2%  69.8%  ggcgaggcaggtggtatagcgattcatggagaaagccccacaattctgttcgacagtaactgcacactagattcatggac
GA2e.Bsal.DSM.18170   99.7%  79.1%  ggcgaaacgggcggagtggctattcatggagcaagccctactatactgtatgaaaataacaagacactcgactcatggac
consensus/100%                      GGsGAuuCuGGsGGsuTuGCsATTCAsGGAusAAGCCCsACsATssTsTssGAsAusAACsusACuCTsGAsTCsTGGAs
consensus/90%                       GGsGAuuCuGGsGGsuTuGCsATTCAsGGAusAAGCCCsACsATssTsTssGAsAusAACsusACuCTsGAsTCsTGGAs
consensus/80%                       GGsGAGuCuGGCGGAGTGGCsATTCAsGGAGCAAGCCCsACuATACTGTATGAuAAsAACuGuACACTsGAsTCuTGGAs
consensus/70%                       GGsGAGuCuGGCGGAGTGGCsATTCAsGGAGCAAGCCCsACuATACTGTATGAuAAsAACuGuACACTsGAsTCuTGGAs

                         cov    pid 15201.       .         .         .         .         .         .         .         . 15280
GA2a.Bcac.CL03T12C61 100.0% 100.0%  ggaccagaccctctccgccatcataaaggaggcgacacaggaatacgggaaggtcccgctggcttccaatcccaaatact
GA2b.Bfra.CL05T12C13  99.7%  81.0%  ggatcagtctctctctaccataataaaggaggtgacgcaggagtatggaaaggtcaatctggtttccaatcccaagtatg
GA2c.Bvul.TF05-18     99.8%  82.0%  ggaccagtccctttccaccataataaaggaagcgacacaggaatatggaaaggtcaacctgatttccaaccccaagtatg
GA2d.Buni.BIOML-A83   99.2%  69.8%  tgaacaatcattatctgcaatcattaatgaagcgacacaagaatatggcaacgagaaaatagttattgcacctaaatata
GA2e.Bsal.DSM.18170   99.7%  79.1%  ggatcagaccttatccgccattataaaggaagtgacacaggagtacggaaaagtcagtctggtctctaatcctgaatatg
consensus/100%                      sGAsCAusCssTsTCsuCsATsATsAAsGAuGsGACuCAuGAuTAsGGsAAsGssssssTuusssssussCCsuAuTAss
consensus/90%                       sGAsCAusCssTsTCsuCsATsATsAAsGAuGsGACuCAuGAuTAsGGsAAsGssssssTuusssssussCCsuAuTAss
consensus/80%                       GGAsCAGsCssTsTCsuCCATsATAAAGGAuGsGACACAGGAuTAsGGuAAuGTCAusCTGGTTTCsAAsCCsAAuTATu
consensus/70%                       GGAsCAGsCssTsTCsuCCATsATAAAGGAuGsGACACAGGAuTAsGGuAAuGTCAusCTGGTTTCsAAsCCsAAuTATu

                         cov    pid 15281.       .         .         .         .         .         .         .         . 15360
GA2a.Bcac.CL03T12C61 100.0% 100.0%  ccgccaaaatcccttacatggcgcagtacaacgagagcgtcttcgactttatgaaccggctctccgcccaatacggcgag
GA2b.Bfra.CL05T12C13  99.7%  81.0%  caacgcccataccctacatggcgcagtataacgagagtgtctttgactttatgaatcgcctttcggctctttatgggggag
GA2c.Bvul.TF05-18     99.8%  82.0%  cgacgcctataccttatatggcgcagtataatgagagtgtctttgacttcatgaaccgcctctcggcactctatggggag
GA2d.Buni.BIOML-A83   99.2%  69.8%  cgaacccgatttcatatctggcacaatacaacgaaagcgcatttgcattcatgaaccgcctttctttgttatatggcgag
GA2e.Bsal.DSM.18170   99.7%  79.1%  atttgcctatcccttacatggcacaatacaacgaaagtgtctttgattttatgaatcgcctttcggccctgtatggggaa
consensus/100%                      ssssssssATssCsTAssTGGCuCAuTAsAAsGAuAGsGssTTsGssTTsATGAAsCGsCTsTCssssssTAsGGsGAu
consensus/90%                       ssssssssATssCsTAssTGGCuCAuTAsAAsGAuAGsGssTTsGssTTsATGAAsCGsCTsTCssssssTAsGGsGAu
consensus/80%                       CsussCCsATsCCsTAsATGGCuCAuTAsAACGAuAGsGTCTTTGAsTTsATGAAsCGCCTsTCsGCsCTsTATGGsGAG
consensus/70%                       CsussCCsATsCCsTAsATGGCuCAuTAsAACGAuAGsGTCTTTGAsTTsATGAAsCGCCTsTCsGCsCTsTATGGsGAG
```

```
                    cov    pid 15361.        .         .         .         .         .         .         .         . 15440
GA2a.Bcac.CL03T12C61 100.0% 100.0%  tggttctattacgacggtaaaaaagtctatttcggcaaacccgaca---aggacaacacggaaaagatcatgtacgatgt
GA2b.Bfra.CL05T12C13  99.7%  81.0%  tggttctattatgacgggacgaaagtgtatttcggcaagcctgaca---gggacaatacggagaagattgtttatgacat
GA2c.Bvul.TF05-18     99.8%  82.0%  tggttctattatgatgggacaaaagtgtatttcggaaagccggaga---aggacaatacggagaaaatcgtttacgacat
GA2d.Buni.BIOML-A83   99.2%  69.8%  tggttctattatgacggcaataaaatttacttcggtaaaccggaaa---atattaatacggcaaagcttcaatatgacgt
GA2e.Bsal.DSM.18170   99.7%  79.1%  tggtttattacgatggcgccaaaacttatttggcaagccggacaaggaggacgaccctaagaagatcttgtatgacgt
consensus/100%                      TGGTTsTATTAsGAsGGsussAAAussTAsTTsGGsAAuCCsGAsA...usussuAssCsusuAAusTssssTAsGAsuT
consensus/90%                       TGGTTsTATTAsGAsGGsussAAAussTAsTTsGGsAAuCCsGAsA...usussuAssCsusuAAusTssssTAsGAsuT
consensus/80%                       TGGTTCTATTAsGAsGGsAssAAAuTsTATTTCGGsAAuCCsGAsA...AGGACAAsACGGAuAAGATssTsTAsGACuT
consensus/70%                       TGGTTCTATTAsGAsGGsAssAAAuTsTATTTCGGsAAuCCsGAsA...AGGACAAsACGGAuAAGATssTsTAsGACuT

                    cov    pid 15441.        .         .         .         .         .         .         .         . 15520
GA2a.Bcac.CL03T12C61 100.0% 100.0%  ggacctggaggaggtacgtctggtggcaaatctggtgccgggcaaatctgcaagatacgattatgtagcgcaggagaaca
GA2b.Bfra.CL05T12C13  99.7%  81.0%  ggatttggaggaggtacgtctggttgccaatctggtaccgggtaaatccgcaagatacgattatgtagcgcaggagaaca
GA2c.Bvul.TF05-18     99.8%  82.0%  ggatctggaagaagtgcgtttggttgccaacctgataccgggaaaatctgcaagatacgattatgtagcgcaggagaaca
GA2d.Buni.BIOML-A83   99.2%  69.8%  agatttgaggaagttcgcttgatagccaatatggttcctggaaaatctgcaagatacgattatgtagcgcaggagaaca
GA2e.Bsal.DSM.18170   99.7%  79.1%  ggacttggaagaagtaaggttggtagccaatctggtaccggggaaatctgccagatacgattatgtagcgcaggagaaca
consensus/100%                      uGAssTsGAuGAuGTssGssTGuTsGCsAAssTGuTsCCsGGsAAATCsGCsAGATACGATTATGTAGCGCAGGAGAACA
consensus/90%                       uGAssTsGAuGAuGTssGssTGuTsGCsAAssTGuTsCCsGGsAAATCsGCsAGATACGATTATGTAGCGCAGGAGAACA
consensus/80%                       GGAssTGGAuGAuGTuCGssTGGTsGCCAATCTGGTuCCGGGsAAATCTGCAAGATACGATTATGTAGCGCAGGAGAACA
consensus/70%                       GGAssTGGAuGAuGTuCGssTGGTsGCCAATCTGGTuCCGGGsAAATCTGCAAGATACGATTATGTAGCGCAGGAGAACA

                    cov    pid 15521.        .         .         .         .         .         .         .         . 15600
GA2a.Bcac.CL03T12C61 100.0% 100.0%  aacaacataatgcagacacgcccgccaaacccgatggcatgaatgacctccagtccattgcgcatgactgttccgaaaag
GA2b.Bfra.CL05T12C13  99.7%  81.0%  aacagcataatgcggatacgcctgccaaaccagatggcatgaacgaccttcagtctattgcacacagctgctctgaaaag
GA2c.Bvul.TF05-18     99.8%  82.0%  aacagcataatgcagatacgcccgccaaaccggatggtatgaacgatcttcagtccattgcgcacggttgctccgaaaag
GA2d.Buni.BIOML-A83   99.2%  69.8%  aagaacataatgcggatacaccagccaaaccggaaggatgagcgatcttcaatctattgcacatgcacgttcagaaaag
GA2e.Bsal.DSM.18170   99.7%  79.1%  aaatacataaggcagatacgcccgctaaaccggaaggaatgaacgacctccagtccattgctcatggctgttcggaaaag
consensus/100%                      AAssuCATAAsGCuGAsACuCCsGCsAAACCsGAsuGsATGAusGAsCTsCAuTCsATTGCsCAsusssGsTCsGAAAAG
consensus/90%                       AAssuCATAAsGCuGAsACuCCsGCsAAACCsGAsuGsATGAusGAsCTsCAuTCsATTGCsCAsusssGsTCsGAAAAG
consensus/80%                       AAsAuCATAATGCuGATACGCCsGCCAAACCuGAsGGsATGAACGAsCTsCAGTCsATTGCuCAsGusTGsTCsGAAAAG
consensus/70%                       AAsAuCATAATGCuGATACGCCsGCCAAACCuGAsGGsATGAACGAsCTsCAGTCsATTGCuCAsGusTGsTCsGAAAAG

                    cov    pid 15601.        .         .         .         .         .         .         .         . 15680
GA2a.Bcac.CL03T12C61 100.0% 100.0%  gcgtatggggcaaagacgactgccgccgccaatccgcacatcgccgacaaggcggggctggacgggcagatgaaggtcct
GA2b.Bfra.CL05T12C13  99.7%  81.0%  gcatataccgcaaaaacaacctcggctgccgaccccatgtaacggacaaggcggaactggacgaacagatgaggattgt
GA2c.Bvul.TF05-18     99.8%  82.0%  gcatacgccgcaaaaacaacctcggcagccaatcctcatataacggacaaggctggattggacgagcagatgaaagtcgt
GA2d.Buni.BIOML-A83   99.2%  69.8%  gcatatccagtgaaagtcatatcccagccaatccacatatcaaggataaggcagaacttgatgaccagatgaaaagagt
GA2e.Bsal.DSM.18170   99.7%  79.1%  gcttacagcgcccgcaccacttctgcggcaaatccgcatatcacagacaaggcgggattggacgaacaaatgaaaattgt
consensus/100%                      GCsTAssssGsssusussAsssCssCsGCsuAsCCsCAsuTsussGAsAAGGCsGuusTsGAsGusCAuATGAuuusssT
consensus/90%                       GCsTAssssGsssusussAsssCssCsGCsuAsCCsCAsuTsussGAsAAGGCsGuusTsGAsGusCAuATGAuuusssT
consensus/80%                       GCuTAsussGCuAAuACsACsTCsGCsGCCAATCCsCATATsACuGACAAGGCuGuAsTGGACGAuCAGATGAAuuTsGT
consensus/70%                       GCuTAsussGCuAAuACsACsTCsGCsGCCAATCCsCATATsACuGACAAGGCuGuAsTGGACGAuCAGATGAAuuTsGT
```

```
                      cov     pid 15681.       .       .       .       .       .       .       .       . 15760
GA2a.Bcac.CL03T12C61 100.0% 100.0%  caaaaatgagtccggcgccaatttgttgaacataaggggcataggaaagacatgccggatccggataggcgaagtcattg
GA2b.Bfra.CL05T12C13  99.7%  81.0%  aaagaatgcgtctggcgccaatctcttgaacatcaaaggcataggaaagacttgccggataaggattggtgaaatcatcg
GA2c.Bvul.TF05-18     99.8%  82.0%  cagaaacgaatctggagccaatctattgaatataaagggcatagggaagacttgccggatcaggatcggcgaaatcattg
GA2d.Buni.BIOML-A83   99.2%  69.8%  acgaaatgcttctggagcaagactgctcaatataagtggtacaggaaagacttgccgtatcagaatcggcgaaataattg
GA2e.Bsal.DSM.18170   99.7%  79.1%  aaaaaattcatccggtgccggtctcttgaatataaaggggatcggaaaaacatgccggatcaggatcggagaggttattg
consensus/100%                      ssuuAAssssTCsGGsGCsuussTssTsAAsATsAusGGsAssGGuAAuACsTGCCGsATssGuATsGGsGAuuTsATsG
consensus/90%                       ssuuAAssssTCsGGsGCsuussTssTsAAsATsAusGGsAssGGuAAuACsTGCCGsATssGuATsGGsGAuuTsATsG
consensus/80%                       sAuAAATGsuTCsGGsGCCAuTCTsTTGAAsATAAuuGGsATAGGAAAGACsTGCCGGATCAGGATsGGsGAAuTsATTG
consensus/70%                       sAuAAATGsuTCsGGsGCCAuTCTsTTGAAsATAAuuGGsATAGGAAAGACsTGCCGGATCAGGATsGGsGAAuTsATTG

                      cov     pid 15761.       .       .       .       .       .       .       .       . 15840
GA2a.Bcac.CL03T12C61 100.0% 100.0%  aggtctctttccccggtgacatgaacctgccgcctttgggaaaattccgcatcaccgaaatcacgcacgaggtgcgccgt
GA2b.Bfra.CL05T12C13  99.7%  81.0%  atgtttctttccctgacaggatgaaactgcctcctttaggcaagttccgtattgtcgggattgagcatgaggtacgccgt
GA2c.Bvul.TF05-18     99.8%  82.0%  atgtctctttccctgacaggatgggtctgtctcctttaggaaagttccgcattgtcgaagtcgtgcacgaggtacgccgt
GA2d.Buni.BIOML-A83   99.2%  69.8%  atgtttcgttccccgacaagatgaaactttctccgctcggcaaatatcttgtaacggaaattattcacgaagtacaccgg
GA2e.Bsal.DSM.18170   99.7%  79.1%  acgtggctttttccggatagga tgaaattgtctcctttagggaaattccgtattactgaaatcacccacgaggtgaatcgg
consensus/100%                      AsGTssCsTTsCCsGusuusATGuussTssCsCCssTsGGsAAuTssCssuTsussGuuuTsussCAsGAuGTusussCGs
consensus/90%                       AsGTssCsTTsCCsGusuusATGuussTssCsCCssTsGGsAAuTssCssuTsussGuuuTsussCAsGAuGTusussCGs
consensus/80%                       AsGTsTCTTTCCCsGAsAuGATGAAsCTGsCTCCTTTuGGsAAuTTCCGsATsussGAAATsussCACGAGGTuCuCCGs
consensus/70%                       AsGTsTCTTTCCCsGAsAuGATGAAsCTGsCTCCTTTuGGsAAuTTCCGsATsussGAAATsussCACGAGGTuCuCCGs

                      cov     pid 15841.       .       .       .       .       .       .       .       . 15920
GA2a.Bcac.CL03T12C61 100.0% 100.0%  gacgggcattatgccaacacgtttgccgggattcctgacggaacggtaaacatccctgttcccgacgcaaaactcccgct
GA2b.Bfra.CL05T12C13  99.7%  81.0%  gacggacattactccaatagtttgtcggtgtaccggacggtacggtacatattcctgttcccgatgcgaagcggccgtt
GA2c.Bvul.TF05-18     99.8%  82.0%  gacggacactatacgaactccttcgtcggtattccggacggtacggtacatatccctgtccccgatgtgaggcttccgct
GA2d.Buni.BIOML-A83   99.2%  69.8%  gacggtcattactccaatacatttttcggaattccaagcgatacaacaaatctgcccgtatctgatgtcaggtttcctac
GA2e.Bsal.DSM.18170   99.7%  79.1%  gacggacattatatgaatagtttgtcggcatcccggatcaaatgacacatatccctgtgcccaatgtgaaaatgccgtt
consensus/100%                      GACGGsCAsTAssssAAssssTTsssCGGsuTsCCsuussusAsuusAsAssTsCCsGTssCsuAsGssAuussssCCsss
consensus/90%                       GACGGsCAsTAssssAAssssTTsssCGGsuTsCCsuussusAsuusAsAssTsCCsGTssCsuAsGssAuussssCCsss
consensus/80%                       GACGGuCATTAssCsAAsAssTTTGTCGGsATsCCuGACGusACGusAsATATsCCTGTsCCCGATGsuAuusTsCCGsT
consensus/70%                       GACGGuCATTAssCsAAsAssTTTGTCGGsATsCCuGACGusACGusAsATATsCCTGTsCCCGATGsuAuusTsCCGsT

                      cov     pid 15921.       .       .       .       .       .       .       .       . 16000
GA2a.Bcac.CL03T12C61 100.0% 100.0%  ggcattgcccgaacttgcgacggtgaagaaaaatgacgacgaaaaggaacagggacgggtgaaagtgagtttcgactggc
GA2b.Bfra.CL05T12C13  99.7%  81.0%  ggcgttgccggaactggctacggtgaaggaaaacaacgacgacaagggacagggacgtgtgaaggtggctttcgactggc
GA2c.Bvul.TF05-18     99.8%  82.0%  ggcgttgccggaactggctacggtaaggaagaacgatgatgagaagggcagggacgcgtgaaagtggctttcgactggc
GA2d.Buni.BIOML-A83   99.2%  69.8%  agcaatgccggagctggctacagttcaggaaaataaagatgaaaaagggcaaggaagagttaaggtgcactttcaatggc
GA2e.Bsal.DSM.18170   99.7%  79.1%  ggcattgcctgagttggcaacggtggagaagaacgatgacgaaaaaggacagggacgcgtgaaggttgtctttgatttggc
consensus/100%                      uGCusTGCCsGAusTsGCsACuGTssuGuAuAAsuAsGAsGAsAAuGuuCAuGGAsGsGTsAAuGTssssTTssAsTGGC
consensus/90%                       uGCusTGCCsGAusTsGCsACuGTssuGuAuAAsuAsGAsGAsAAuGuuCAuGGAsGsGTsAAuGTssssTTssAsTGGC
consensus/80%                       GGCuTTGCCsGAuCTGGCsACGGTuuAGuAuAAsuAsGAsGAuAAuGGuCAGGGACGsGTGAAuGTGussTTsGAsTGGC
consensus/70%                       GGCuTTGCCsGAuCTGGCsACGGTuuAGuAuAAsuAsGAsGAuAAuGGuCAGGGACGsGTGAAuGTGussTTsGAsTGGC
```

```
                        cov    pid 16001.        .         .         .         .         .         .         .         . 16080
GA2a.Bcac.CL03T12C61 100.0% 100.0%    agaaaaacgggaaaacaaccaactggatcagggtgcagagccccaatgcgggagtaagcgacgccgtatccaaaaaccgc
GA2b.Bfra.CL05T12C13  99.7%  81.0%    agaaaaacggcaagacaaccaattgggtcagggtgcagactcctaatgcgggagtaagtggtgcggttcccaagaatcgc
GA2c.Bvul.TF05-18     99.8%  82.0%    agaagaatgataagacaaccaattggatcagggtgcagagtccgaatgcgggagtaagcgatgcggttcccaaaaatcgc
GA2d.Buni.BIOML-A83   99.2%  69.8%    aaaaaggcagcaaaactacaaattggatcagagtacaaagtcctaatgccggaaccagtgatgccgtaagcaaaaaccgc
GA2e.Bsal.DSM.18170   99.7%  79.1%    agaaaaagggaaaaacgaccaattggatcagggtacagagccctaatgcgggaacaagcgatgcggtatccaaaaaccgg
consensus/100%                        AuAAuuusuusAAuACsACsAAsTGGuTCAGuGTuCAuAssCCsAATGCsGGAussAGsGusGCsGTsssCAAuAAsCGs
consensus/90%                         AuAAuuusuusAAuACsACsAAsTGGuTCAGuGTuCAuAssCCsAATGCsGGAussAGsGusGCsGTsssCAAuAAsCGs
consensus/80%                         AGAAAAAsGGsAAuACuACCAATTGGATCAGGGTuCAGAGsCCsAATGCGGGAusAAGsGATGCsGTssCCAAAAAsCGC
consensus/70%                         AGAAAAAsGGsAAuACuACCAATTGGATCAGGGTuCAGAGsCCsAATGCGGGAusAAGsGATGCsGTssCCAAAAAsCGC

                        cov    pid 16081.        .         .         .         .         .         .         .         . 16160
GA2a.Bcac.CL03T12C61 100.0% 100.0%    ggatgggtgtttgttcccgaagtcggtgaccaggtcatggtggggtacgaacacggcaatccggaccgcccctatgtgac
GA2b.Bfra.CL05T12C13  99.7%  81.0%    ggatgggtatttgttccggaagtcggcgatcaggttatggtaagctacgagcatggcaacccggaccgcccctatgttac
GA2c.Bvul.TF05-18     99.8%  82.0%    ggatgggtatttgtcccggaagtcggagaccaggtcatggtcggctatgagcacggcaatccggatcgcccttatgtaac
GA2d.Buni.BIOML-A83   99.2%  69.8%    ggacttgtattcgtaccggaggttggtgaccaggttatgattggctatgaacatggaaacccggaccgcccctatgtttc
GA2e.Bsal.DSM.18170   99.7%  79.1%    ggatgggtatttgttccggaagtcggggaccaggtgatggtcggttatgaacacggcaatccggaccgtccttatgtaac
consensus/100%                        GGAsssGTuTTsGTsCCsGAuGTsGGsGAsCAGGTsATGuTsuGsTAsGAuCAsGGsAAsCCGGAsCGsCCsTATGTssC
consensus/90%                         GGAsssGTuTTsGTsCCsGAuGTsGGsGAsCAGGTsATGuTsuGsTAsGAuCAsGGsAAsCCGGAsCGsCCsTATGTssC
consensus/80%                         GGATGGGTATTTGTsCCGGAAGTCGGsGACCAGGTsATGGTsGGsTAsGAuCAsGGCAAsCCGGACCGCCCsTATGTsAC
consensus/70%                         GGATGGGTATTTGTsCCGGAAGTCGGsGACCAGGTsATGGTsGGsTAsGAuCAsGGCAAsCCGGACCGCCCsTATGTsAC

                        cov    pid 16161.        .         .         .         .         .         .         .         . 16240
GA2a.Bcac.CL03T12C61 100.0% 100.0%    cggagccatgttccattccgcttcaggaaaggaggggacaaggacaacaagaccaaaagcattatcacgcgcagcggaa
GA2b.Bfra.CL05T12C13  99.7%  81.0%    gggatcggtgttccattccggttcaggtaaaggaggggacaaggacaacaaggtcaagagcattatcacacgcagcggca
GA2c.Bvul.TF05-18     99.8%  82.0%    gggagccatgttccattccgcttcgggaaaggtggcgatgagaacaacaagaccaaaagcatcatcacacgcagcggta
GA2d.Buni.BIOML-A83   99.2%  69.8%    cggagccatgttccactcaggatcgggacaaggaggaggtgataacaatcagcttaaaacaatcattacccgtagtggta
GA2e.Bsal.DSM.18170   99.7%  79.1%    cggagccatgtttcacaaggactcgggaaagggcggagacaaggacaacaaactgaaaagtatcatcacccgaagtggaa
consensus/100%                        sGGAsCsuTGTTsCAssssGssTCuGGssAuGGsGGsGusuAsuACAAssAusssAAuAssATsATsACsCGsAGsGGsA
consensus/90%                         sGGAsCsuTGTTsCAssssGssTCuGGssAuGGsGGsGusuAsuACAAssAusssAAuAssATsATsACsCGsAGsGGsA
consensus/80%                         sGGAGCCATGTTCCAsTCsGssTCuGGAAAuGGsGGuGAsuAGuACAACAAGsssAAAAGsATsATCACsCGsAGsGGsA
consensus/70%                         sGGAGCCATGTTCCAsTCsGssTCuGGAAAuGGsGGuGAsuAGuACAACAAGsssAAAAGsATsATCACsCGsAGsGGsA

                        cov    pid 16241.        .         .         .         .         .         .         .         . 16320
GA2a.Bcac.CL03T12C61 100.0% 100.0%    gcgccatcgttttcgatgatgagacgggaagcattgtcatcaccgaccggacgggaaaacaactgatcctgctggacggg
GA2b.Bfra.CL05T12C13  99.7%  81.0%    atgccatcgttttgatgatgaaacgggaagcatcgttattaccgaccagacgggaaaacagctgatcatgttggatggc
GA2c.Bvul.TF05-18     99.8%  82.0%    acgccatcgttttcgatgatgagacgggaagtatcgtcattactgaccggacgggaaagcagctgattctgctggacggc
GA2d.Buni.BIOML-A83   99.2%  69.8%    atgctatcatcttcaatgatgaagcgggaagtattactataacagaccagacaggaaaacaactgattcttcttgatgga
GA2e.Bsal.DSM.18170   99.7%  79.1%    acaccattctttttgatgatgagacgggaagtatcaccattatggaccagactgggaaacagcttattgttcttggatggg
consensus/100%                        usuCsATssTsTTsuATGATGAuuCGGGAAGsATsussATsAssGACCuGACsGGuAAuCAuCTsATssTssTsGAsGGs
consensus/90%                         usuCsATssTsTTsuATGATGAuuCGGGAAGsATsussATsAssGACCuGACsGGuAAuCAuCTsATssTssTsGAsGGs
consensus/80%                         AsGCCATCuTTTTsGATGATGAuACGGGAAGsATsussATsACsGACCuGACuGGAAAACAuCTGATssTsCTGGAsGGs
consensus/70%                         AsGCCATCuTTTTsGATGATGAuACGGGAAGsATsussATsACsGACCuGACuGGAAAACAuCTGATssTsCTGGAsGGs
```

```
                     cov    pid 16321.       .         .         .         .         .         .         .         . 16400
GA2a.Bcac.CL03T12C61 100.0% 100.0%  aaggacgccattacgattatggcgaaaaagagcgtagtcatcaccaacgaggacaagtccgttatcgtaatggacgaaaa
GA2b.Bfra.CL05T12C13  99.7%  81.0%  acggatgccattaccgtcatggcgaaaaagagcattaccctaccaacgaggcggaatcggtcatcgtaatggatgataa
GA2c.Bvul.TF05-18     99.8%  82.0%  acggatgccattaccgtcatggcgaaaaaagcgttaccattaccaacgaggatgaatcggtcatcataatggacgaaaa
GA2d.Buni.BIOML-A83   99.2%  69.8%  aaggacagcattaccattatggcgaaaaagagtgtgattatcacaaacgaatcggaatcaataattgcaatggaggataa
GA2e.Bsal.DSM.18170   99.7%  79.1%  aaagatacgattaatatcatggcaaagaaaacggtgaccgtcaccaacgaagaagagtcggttattatcatggatggcag
consensus/100%                      AsuGAsussATTAssuTsATGGCuAAuAAuAssuTsussTsACsAACGAusssuAuTCsuTsATsussATGGAsGusAu
consensus/90%                       AsuGAsussATTAssuTsATGGCuAAuAAuAssuTsussTsACsAACGAusssuAuTCsuTsATsussATGGAsGusAu
consensus/80%                       AsGGAsuCCATTACsuTsATGGCGAAAAAuAGsGTsAsCuTsACCAACGAuGssGAuTCuGTsATsuTAATGGAsGAsAA
consensus/70%                       AsGGAsuCCATTACsuTsATGGCGAAAAAuAGsGTsAsCuTsACCAACGAuGssGAuTCuGTsATsuTAATGGAsGAsAA

                     cov    pid 16401.       .         .         .         .         .         .         .         . 16480
GA2a.Bcac.CL03T12C61 100.0% 100.0%  gtccatcggtatgcaggcggacacgatttccatcgaaggaaagaaaaacattacgttggtttcgggcaaggaaagtatgg
GA2b.Bfra.CL05T12C13  99.7%  81.0%  gtcgataggcttgcaggccgataccattgctctcgaaggcaggaaaagtgtaacattgctgtccggcaacgaatgtatgg
GA2c.Bvul.TF05-18     99.8%  82.0%  gtcgataggcttgcaggcggataccatttccattgaaggcaggaaaagcgtgacgttgttgtccggcaacgagaacatgg
GA2d.Buni.BIOML-A83   99.2%  69.8%  atccattggcatgaaagccgaaacaatctgtctcgaagctgaaaagaacattgcacttgtttctggtgacgaaaatctga
GA2e.Bsal.DSM.18170   99.7%  79.1%  gtctatcggcttgcagtctgatacgatttccattgaagggaggaaaagcattacactgttgtcaggcggtgagaacatga
consensus/100%                      uTCsATsGGssTGsAusCsGAsACsATsssssTsGAAGssuuuAAuAusuTsuCusTssTsTCsGGsuusGAusussTGu
consensus/90%                       uTCsATsGGssTGsAusCsGAsACsATsssssTsGAAGssuuuAAuAusuTsuCusTssTsTCsGGsuusGAusussTGu
consensus/80%                       GTCsATsGGCsTGCAGGCsGAsACsATTTCssTsGAAGGsAuGAAAAuCuTsACusTGsTsTCsGGCuAsGAuAusATGu
consensus/70%                       GTCsATsGGCsTGCAGGCsGAsACsATTTCssTsGAAGGsAuGAAAAuCuTsACusTGsTsTCsGGCuAsGAuAusATGu

                     cov    pid 16481.       .         .         .         .         .         .         .         . 16560
GA2a.Bcac.CL03T12C61 100.0% 100.0%  tattcagcagcgaaaaaaacatcatcaacggcagtgccaccaatatcaagctggaggcggccaaggaatatgacttgaat
GA2b.Bfra.CL05T12C13  99.7%  81.0%  tgctcagcagcgaaaagagtataattagcagcagcggtaccaatatcaaacaggaggcggaaaaggattacgatgttgct
GA2c.Bvul.TF05-18     99.8%  82.0%  tactcagcagcgagaagaatataatcagtggaagcggtaccaatataaaaatggaagcggccaaagaatatgacctgacc
GA2d.Buni.BIOML-A83   99.2%  69.8%  ttatcagtaaagaaaagacagccatagctacaaccgggacaaatataaaggttgaagcacaaagtcaatatgagcaaacc
GA2e.Bsal.DSM.18170   99.7%  79.1%  tactcagcggtgaaaagaagataatcggcagcagtggaaccaatatcaagcaggaggcttccaaaggatatgacttgagt
consensus/100%                      TssTCAGsuusGAuAAuAssussATsusussAssGssACsAATATsAAusssGAuGCssssAussusTAsGAssssuss
consensus/90%                       TssTCAGsuusGAuAAuAssussATsusussAssGssACsAATATsAAusssGAuGCssssAussusTAsGAssssuss
consensus/80%                       TusTCAGCAGsGAAAAGAusATsATsuusuGsAGsGGsACCAATATsAAussGGAuGCusssAAuGAATATGAssTuAss
consensus/70%                       TusTCAGCAGsGAAAAGAusATsATsuusuGsAGsGGsACCAATATsAAussGGAuGCusssAAuGAATATGAssTuAss

                     cov    pid 16561.       .         .         .         .         .         .         .         . 16640
GA2a.Bcac.CL03T12C61 100.0% 100.0%  acacagaccggaaccatgaaaggcacgaacctcaccgtggagggaacggcaaacatgaccgtaacgggaggcattgtgaa
GA2b.Bfra.CL05T12C13  99.7%  81.0%  gcaaagaacggaacggtcaacggtgtaaatctgatgatagagggaaagggaaacgtaacggtttcgggaggaatagtaaa
GA2c.Bvul.TF05-18     99.8%  82.0%  acccggactggaacgctcaacggtacgaacctcgtaatagaaggcacggcggatgtggcggtttcgggaggaatggtaaa
GA2d.Buni.BIOML-A83   99.2%  69.8%  agcaaagcaggtacagttacagccaacaacatgtccatagaaggtacagccgatgttaccgtaacaggaggcatcgtaaa
GA2e.Bsal.DSM.18170   99.7%  79.1%  actcagaaaggcacagtcaaaggtacaaacttgtcaatcgaaggcacagccgatgtagcggtaacaggaggaatcataaa
consensus/100%                      usssuuussGGsACssTsAssGssussAAssTssssuTsGAuGGsAsuGssuAsuTsuCsGTssCuGGAGGsATsuTuAA
consensus/90%                       usssuuussGGsACssTsAssGssussAAssTssssuTsGAuGGsAsuGssuAsuTsuCsGTssCuGGAGGsATsuTuAA
consensus/80%                       ACssAGAssGGsACuuTsAAsGGsAsuAACsTssssATuGAuGGsACuGCsuAsGTuuCsGTssCuGGAGGsATsGTAAA
consensus/70%                       ACssAGAssGGsACuuTsAAsGGsAsuAACsTssssATuGAuGGsACuGCsuAsGTuuCsGTssCuGGAGGsATsGTAAA
```

```
                     cov    pid 16641.         .         .         .         .         .         .         .         . 16720
GA2a.Bcac.CL03T12C61 100.0% 100.0%  aattaattcataaacggattggatatgggagcatcagcatctgtaatacaggaatactacaaggcggtcgattactgggc
GA2b.Bfra.CL05T12C13  99.7%  81.0%  gtttaactcataattcggttggccatgggagcatctgcatctgtgatacaggaatattacaaagcggtcgattactgggc
GA2c.Bvul.TF05-18     99.8%  82.0%  aatcaattcgtaatccggttggttatgggagcatctgcatctgttatacaggaatattacaaagcggtcgattactgggc
GA2d.Buni.BIOML-A83   99.2%  69.8%  aataaactcttaaacaaact-aatatgggagcatcagcatctgttatacaagaatactataaagcggttgattactgggc
GA2e.Bsal.DSM.18170   99.7%  79.1%  gttcaactcataaaacctcaggatatgggagcatctgcgtctgttgtacaggaatacttaaggcggtcgattattgggc
consensus/100%                      usTsAAsTCsTAAsssssss.ussATGGGAGCATCsGCuTCTGTsuTACAuGAATAsTssAAuGCGGTsGATTAsTGGGC
consensus/90%                       usTsAAsTCsTAAsssssss.ussATGGGAGCATCsGCuTCTGTsuTACAuGAATAsTssAAuGCGGTsGATTAsTGGGC
consensus/80%                       usTsAAsTCuTAAsssuusTGGsTATGGGAGCATCsGCATCTGTsATACAGGAATAsTAsAAuGCGGTCGATTACTGGGC
consensus/70%                       usTsAAsTCuTAAsssuusTGGsTATGGGAGCATCsGCATCTGTsATACAGGAATAsTAsAAuGCGGTCGATTACTGGGC

                     cov    pid 16721.         .         .         .         .         .         .         .         . 16800
GA2a.Bcac.CL03T12C61 100.0% 100.0%  ggacatagccggaaagaaagactggaaactggcgatatggattgtcggaaggaacgatgtggacctggtggacaagttcc
GA2b.Bfra.CL05T12C13  99.7%  81.0%  ggacatagtcggcaacagggactggaaactgtctgtatggattgtcgggcagaatgatgtggacttggtagacaggtttc
GA2c.Bvul.TF05-18     99.8%  82.0%  ggacatagccggtaacagggactggaaactgtctgtatggattgtcggtcagaatgatgtggacctggtggacaggtttt
GA2d.Buni.BIOML-A83   99.2%  69.8%  tgaaatagaagtccgtaaaaactggaagctggcaatatgggttgtcgaagctacagacgtagatttgattgatcgttttt
GA2e.Bsal.DSM.18170   99.7%  79.1%  ggatattgccgataaggaggactggaaactggctatatggattgccggacagaacgatgtggatttggttgacaggttct
consensus/100%                      sGAsATsGssGsssusuuuuACTGGAAuCTGsCsuTATGGuTTGsCGussssAssGAsGTuGAssTGuTsGAssusTTss
consensus/90%                       sGAsATsGssGsssusuuuuACTGGAAuCTGsCsuTATGGuTTGsCGussssAssGAsGTuGAssTGuTsGAssusTTss
consensus/80%                       GGAsATAGsCGusAAsAuuGACTGGAAACTGsCsuTATGGATTGTCGGusuGAAsGATGTGGAssTGGTsGACAGGTTss
consensus/70%                       GGAsATAGsCGusAAsAuuGACTGGAAACTGsCsuTATGGATTGTCGGusuGAAsGATGTGGAssTGGTsGACAGGTTss

                     cov    pid 16801.         .         .         .         .         .         .         .         . 16880
GA2a.Bcac.CL03T12C61 100.0% 100.0%  tggagatagagcgttctcctgtaggacagttcgatgacatatttttccgtttcgacacgccctacagaggcgatgacgac
GA2b.Bfra.CL05T12C13  99.7%  81.0%  tggaaatagaacgttctccggtagggcagttcgatgacatattctttcgttttgatacgccgtacaggggggatgacgag
GA2c.Bvul.TF05-18     99.8%  82.0%  tggaaatagaacgttctccggtaggacggttcgacgatatattctttcgttttgatactccgtataaggggatgacgag
GA2d.Buni.BIOML-A83   99.2%  69.8%  ttgaaatagagcgttccccggtaggacagtttgatgacattttttccgctttgactcgatatatgagggcgatgatgaa
GA2e.Bsal.DSM.18170   99.7%  79.1%  tggaaatcgaacgctcgcctgtcggacagttcgaggatatttcttccggtttgagacgccgtatgagggaagcggtgat
consensus/100%                      TsGAuATsGAuCGsTCsCCsGTsGGuCuGTTsGAsGAsATsTTsTTsCGsTTsGAssCssssTAsuuuGGsuusGusGAs
consensus/90%                       TsGAuATsGAuCGsTCsCCsGTsGGuCuGTTsGAsGAsATsTTsTTsCGsTTsGAssCssssTAsuuuGGsuusGusGAs
consensus/80%                       TGGAAATAGAuCGTTCsCCsGTAGGACAGTTCGAsGAsATsTTsTTsCGsTTTGAsACGCCuTAsuuGGGsGATGAsGAs
consensus/70%                       TGGAAATAGAuCGTTCsCCsGTAGGACAGTTCGAsGAsATsTTsTTsCGsTTTGAsACGCCuTAsuuGGGsGATGAsGAs

                     cov    pid 16881.         .         .         .         .         .         .         .         . 16960
GA2a.Bcac.CL03T12C61 100.0% 100.0%  gaatatgccgcacagctctggcaggagtatgccggatggtttgatgaacaggcggaagagaaggatgacatgctcaaggc
GA2b.Bfra.CL05T12C13  99.7%  81.0%  gaatacactgaacagctctggcaggaatatgcgggttggttttcagaaaaggttgaggagaagtatgatattctcaaggc
GA2c.Bvul.TF05-18     99.8%  82.0%  gaatacgccgaacagctctggcaggaatatgcgggctggttctcggaaaaggttgaggagaagtatgatattctcagggc
GA2d.Buni.BIOML-A83   99.2%  69.8%  aaattcacagagcaactttggaaagaatatgtcagctggtttacagaagaaattgatgaaagtatgatgttctcaaggc
GA2e.Bsal.DSM.18170   99.7%  79.1%  gaatacaccgaacagctatggcaggaatatgccggctggttctcagaagaagtggaggaaaaatacgacatcatcaaggc
consensus/100%                      uAATssuCsGsuCAuCTsTGGsAuGAuTATGssuGsTGGTTssssGAAsAuussGAsGAuAAusAsGAsuTssTCAuGGC
consensus/90%                       uAATssuCsGsuCAuCTsTGGsAuGAuTATGssuGsTGGTTssssGAAsAuussGAsGAuAAusAsGAsuTssTCAuGGC
consensus/80%                       GAATACuCsGAACAGCTsTGGCAGGAATATGCsGGsTGGTTssCuGAAuAuGTsGAuGAuAAGTATGAsATsCTCAAGGC
consensus/70%                       GAATACuCsGAACAGCTsTGGCAGGAATATGCsGGsTGGTTssCuGAAuAuGTsGAuGAuAAGTATGAsATsCTCAAGGC
```

```
                        cov    pid 16961.        .         .         .         .         .         .         .         . 17040
GA2a.Bcac.CL03T12C61 100.0% 100.0%  gttgcggcacgacgggctgctcaggacggagtaccgccccgacacgtcggccgagcctacggcggcaaacctgtggaagg
GA2b.Bfra.CL05T12C13  99.7%  81.0%  tctgcggcacgacgggctgctgaaggaagaatacattcctgatgtgtcggttgagcatacggcgggcaacttgtggagag
GA2c.Bvul.TF05-18     99.8%  82.0%  tctgcggcacgacgggctgttgaaggaagaatatacccccgatgtgtcggttgaacatacggcgggcaacttgtggagag
GA2d.Buni.BIOML-A83   99.2%  69.8%  attgaaacaggacggactattaaccggagactattttccccggacagacattcctcatactactgttgccttatggcagg
GA2e.Bsal.DSM.18170   99.7%  79.1%  cttgcggcacgatggactgttgaaagaagaatatgtcccggacatgtctgtcgaacatacggcagccaatctgtggaaag
consensus/100%                      ssTGsuuCAsGAsGGuCTusTsAssusuGAsTAssssCCssusususssusssssCsTACsuCsGssusssTuTGGsuuG
consensus/90%                       ssTGsuuCAsGAsGGuCTusTsAssusuGAsTAssssCCssusususssusssssCsTACsuCsGssusssTuTGGsuuG
consensus/80%                       ssTGCGGCACGACGGuCTGsTuAuuGuAGAuTAssssCCsGAsusGTCsGTsGAuCATACGGCuGssAACsTGTGGAuuG
consensus/70%                       ssTGCGGCACGACGGuCTGsTuAuuGuAGAuTAssssCCsGAsusGTCsGTsGAuCATACGGCuGssAACsTGTGGAuuG

                        cov    pid 17041.        .         .         .         .         .         .         .         . 17120
GA2a.Bcac.CL03T12C61 100.0% 100.0%  agatgttgcgcttcaaggagtgcatatcccgcctggaaaatgccttcttctgcatctattttcctccggaacaatccggg
GA2b.Bfra.CL05T12C13  99.7%  81.0%  agatgttgcgtttcaaggcatgtatctcccggttggatgatgcctttttctgtctgtactttcctccggaacaggaaagg
GA2c.Bvul.TF05-18     99.8%  82.0%  agatgttgcgtttcaaggcatgtatctcccggttggatgatgcctttttctgtctgtactttcctccggaacaggaaagg
GA2d.Buni.BIOML-A83   99.2%  69.8%  agatgctacggctaaatcctgtatctccggactggaagacaccggctttgcatctatttccctcccggacaattgaaa
GA2e.Bsal.DSM.18170   99.7%  79.1%  agatgttgcgtttcaaggcatgtatatccggattggagaatgcctgtttctgcatctattttcctctgggacaggaaagg
consensus/100%                      AGATGsTuCGssTsAAusssTGsATsTCCsGssTGGAsuAsuCCsssTTsTGssTsTAsTTsCCTCssGuACAusssuuu
consensus/90%                       AGATGsTuCGssTsAAusssTGsATsTCCsGssTGGAsuAsuCCsssTTsTGssTsTAsTTsCCTCssGuACAusssuuu
consensus/80%                       AGATGTTGCGsTTCAAGGCuTGTATsTCCsGusTGGAsuATGCCTssTTCTGssTsTAsTTTCCTCCGGuACAussuAGG
consensus/70%                       AGATGTTGCGsTTCAAGGCuTGTATsTCCsGusTGGAsuATGCCTssTTCTGssTsTAsTTTCCTCCGGuACAussuAGG

                        cov    pid 17121.        .         .         .         .         .         .         .         . 17200
GA2a.Bcac.CL03T12C61 100.0% 100.0%  gaatttccccggacggaatggttcggacaggtactgaaagagggtgttccgcagggcatccgtctgacaaccattgattt
GA2b.Bfra.CL05T12C13  99.7%  81.0%  gggtattcccggacaggatggttcggcaatgtgctgaaagaaggtgtcccgcagggaatccggatgacaaccattgactt
GA2c.Bvul.TF05-18     99.8%  82.0%  gggtgctcccggacaggatggttcggcaatgtgctgaaagagggcgtcccgcagggaatccggatgacaaccattgattt
GA2d.Buni.BIOML-A83   99.2%  69.8%  ggtccgaaacgtacagaatggttctcaaaagtcttatcagaaggtataccgcaaggaattcggttagccactattgatat
GA2e.Bsal.DSM.18170   99.7%  79.1%  gggctttccaggaccgaatggttcaagaaggtcttggaagaagggattccgcagggaatccggttaacgaccattgacat
consensus/100%                      GussssssssGsACsGuATGGTTCssssAsGTssTussAGAuGGsuTsCCGCAuGGsATsCGssTuuCsACsATTGAssT
consensus/90%                       GussssssssGsACsGuATGGTTCssssAsGTssTussAGAuGGsuTsCCGCAuGGsATsCGssTuuCsACsATTGAssT
consensus/80%                       GGussssCCCGGACuGuATGGTTCuusAAsGTssTGuAAGAuGGsuTsCCGCAGGGAATCCGGsTuACuACCATTGAssT
consensus/70%                       GGussssCCCGGACuGuATGGTTCuusAAsGTssTGuAAGAuGGsuTsCCGCAGGGAATCCGGsTuACuACCATTGAssT

                        cov    pid 17201.        .         .         .         .         .         .         .         . 17280
GA2a.Bcac.CL03T12C61 100.0% 100.0%  gaagaaggacagaaatgtcgctttggacgagtcgccggaggtggtccacatacgtccacgctttgacatggcggcggcac
GA2b.Bfra.CL05T12C13  99.7%  81.0%  gaaaaagaacaggagtatccggctgggtgagtctcgggaagtggttgcatacgcccgcagttcgatatggcggcggctt
GA2c.Bvul.TF05-18     99.8%  82.0%  gaaagggaacaggaatgtcaggctggacgagtcccgggaggtggttgcatacgccccggttcgatatggcggcggcat
GA2d.Buni.BIOML-A83   99.2%  69.8%  agccgggaaccgtagcatacgacttgacaactcgcggatggtagcctgtatttatcccaaattcaatctggttcaggctt
GA2e.Bsal.DSM.18170   99.7%  79.1%  gaagaaaaacagaagtgtcaatctggatgaatcgccggctgtaatttatatctgccccaagttcgatatggcggcggccc
consensus/100%                      uussuuuuACsGsAusuTsssssTsGusuAsTCsCsGussGTuussusATssusCCssusTTsuAssTGGssssGGCss
consensus/90%                       uussuuuuACsGsAusuTsssssTsGusuAsTCsCsGussGTuussusATssusCCssusTTsuAssTGGssssGGCss
consensus/80%                       GAAuuuGAACAGuAuTuTCsusCTGGAsGAuTCsCsGGsuGTuGTsTusATssGsCCssuuTTCGATATGGCGGCGGCss
consensus/70%                       GAAuuuGAACAGuAuTuTCsusCTGGAsGAuTCsCsGGsuGTuGTsTusATssGsCCssuuTTCGATATGGCGGCGGCss
```

```
                      cov    pid 17281.         .         .         .         .         .         .         .         . 17360
GA2a.Bcac.CL03T12C61 100.0% 100.0%   tgcataaccggatggcacgggcggatgccggcaacgacctgatcgcgccggagaaccggttcaaacaacaggtgacggcg
GA2b.Bfra.CL05T12C13  99.7%  81.0%   tacataaccggatggcacggtcggatagcggtaacgacctgattgcacctgaaaatcggttcaaacagcaggttacggtt
GA2c.Bvul.TF05-18     99.8%  82.0%   tgcataaccggatggcacggtcggatagcggtaatgacctgattgcgcctgaaaaccggttcaagcagcaggtgacggtt
GA2d.Buni.BIOML-A83   99.2%  69.8%   tgcgcaaccgtatagcacaaggagatgccggaggagaccttacagctcccgaaaacaagtttaaacaacaggttaccgtt
GA2e.Bsal.DSM.18170   99.7%  79.1%   tgcataaccggatggcacggagcgatacgggcaatgacctgattgcacccgaaaaccggttcaaacagcaggtgagtgcc
consensus/100%                       TuCusAACCGsATuGCACuusssGATussGGsuusGACCTsAssGCsCCsGAuAAssuGTTsAAuCAuCAGGTsAssGss
consensus/90%                        TuCusAACCGsATuGCACuusssGATussGGsuusGACCTsAssGCsCCsGAuAAssuGTTsAAuCAuCAGGTsAssGss
consensus/80%                        TGCATAACCGGATGGCACGGssuGATusCGGsAAsGACCTGATsGCuCCsGAAAACCGGTTCAAACAuCAGGTsACsGss
consensus/70%                        TGCATAACCGGATGGCACGGssuGATusCGGsAAsGACCTGATsGCuCCsGAAAACCGGTTCAAACAuCAGGTsACsGss

                      cov    pid 17361.         .         .         .         .         .         .         .         . 17440
GA2a.Bcac.CL03T12C61 100.0% 100.0%   gtcatggactgcacgcggaaacaggatttgaaaccgctggaaaaggaaataaggaagctcctggatatcgccggggaact
GA2b.Bfra.CL05T12C13  99.7%  81.0%   gtcatggattctacacaaaagcaggattggaaattattggacagggaaatccgaaaactgttggatattgcacaggaaat
GA2c.Bvul.TF05-18     99.8%  82.0%   gtcatggattgtacacaaaagcaggattggaaattattggacagggaaatccgaaaactattggatatttccaaagaaat
GA2d.Buni.BIOML-A83   99.2%  69.8%   gtaatggactgtacccaaaaacaggataaaaaactgttaggaagggaaatcaacaaacttttggacatagccaaagaact
GA2e.Bsal.DSM.18170   99.7%  79.1%   gtcatggattgcacgcaaaaacaggattggaaactgttggacaaggaaatccggaaactgctggatgtagccaaggagtt
consensus/100%                       GTsATGGAsTssACsCuuAAuCAGGATssuAAAssusTuGusAuGGAAATssusAAuCTssTGGAsuTssCssuuGAusT
consensus/90%                        GTsATGGAsTssACsCuuAAuCAGGATssuAAAssusTuGusAuGGAAATssusAAuCTssTGGAsuTssCssuuGAusT
consensus/80%                        GTCATGGAsTGsACuCAAAAuCAGGATTuGAAAsTuTTGGAsAuGGAAATCsGuAAACTssTGGATATsGCCuAuGAAsT
consensus/70%                        GTCATGGAsTGsACuCAAAAuCAGGATTuGAAAsTuTTGGAsAuGGAAATCsGuAAACTssTGGATATsGCCuAuGAAsT

                      cov    pid 17441.         .         .         .         .         .         .         .         . 17520
GA2a.Bcac.CL03T12C61 100.0% 100.0%   gaaggatgcgaatattcggatatccgctctcttcatcgcttccgaagcctgctatgctatccgcgaatacaaaagcagca
GA2b.Bfra.CL05T12C13  99.7%  81.0%   aaaggatacgaatatccggatttccgctctgcttatcgcatccgaggcatgttatgctatcagggaatataagcatagca
GA2c.Bvul.TF05-18     99.8%  82.0%   gaaggatacgaatatccggacttccgctctgttcatagcgtctgaggcgtgttacgcgatccgggaatacaggcatagca
GA2d.Buni.BIOML-A83   99.2%  69.8%   aaaagacaatggtatctatatctcttcactgtttattgcatctgaagcattctatgccatcagagaatataaggacagca
GA2e.Bsal.DSM.18170   99.7%  79.1%   gaaggatacgaatatattgacgtccgctctgttcatcgccgctgaagcgtgttatgcgattcgggaatacaaacatagca
consensus/100%                       uAAuGAsussuuTATssssAssTCssCsCTssTsATsGCssCsGAuGCsTssTAsGCsATssGsGAATAsAuususAGCA
consensus/90%                        uAAuGAsussuuTATssssAssTCssCsCTssTsATsGCssCsGAuGCsTssTAsGCsATssGsGAATAsAuususAGCA
consensus/80%                        uAAGGATACGAATATssuGAssTCCGCTCTGTTsATsGCsTCsGAuGCuTGsTATGCsATCsGuGAATAsAAusAsAGCA
consensus/70%                        uAAGGATACGAATATssuGAssTCCGCTCTGTTsATsGCsTCsGAuGCuTGsTATGCsATCsGuGAATAsAAusAsAGCA

                      cov    pid 17521.         .         .         .         .         .         .         .         . 17600
GA2a.Bcac.CL03T12C61 100.0% 100.0%   tgaaatacagcgacaagaccatccgcgaagcggaaaaggcgatgaaggatggcgaggcggcgggatacagttactggaag
GA2b.Bfra.CL05T12C13  99.7%  81.0%   tgaaatacagcgacgaaaccgtcctggaggcggaaaaggcgatgcagggcggtgaaacggcaggatatagttattggaaa
GA2c.Bvul.TF05-18     99.8%  82.0%   tgaagtacagtgatgcgaccatcaaagaggcggagaaggcgatggaaagtggcgaagcggcaggatacagttattggaaa
GA2d.Buni.BIOML-A83   99.2%  69.8%   tgaagtacagtgaaattactcttaatgaagcccaaaaggctatgatgggcggtgatacgacaggatatagctactggaaa
GA2e.Bsal.DSM.18170   99.7%  79.1%   taaagtacagcgacgacacgatcagggaagcggaaaaggcgatggataatggcgaagcggctggatatagctactggaaa
consensus/100%                       TuAAuTACAGsGAsussACssTssssGAuGCssAuAAGGCsATGsssuusGGsGAsuCGuCsGGATAsAGsTAsTGGAAu
consensus/90%                        TuAAuTACAGsGAsussACssTssssGAuGCssAuAAGGCsATGsssuusGGsGAsuCGuCsGGATAsAGsTAsTGGAAu
consensus/80%                        TGAAuTACAGsGAsussACsuTCsusGAuGCGGAAAAGGCGATGuAuuusGGsGAuuCGGCuGGATAsAGsTAsTGGAAA
consensus/70%                        TGAAuTACAGsGAsussACsuTCsusGAuGCGGAAAAGGCGATGuAuuusGGsGAuuCGGCuGGATAsAGsTAsTGGAAA
```

```
                     cov     pid 17601.       .         .         .         .         .         .         .        . 17680
GA2a.Bcac.CL03T12C61 100.0% 100.0% atggctgtttccatgaaggcggcaatcctgacggcagaaaagaaacgcgaagaggcggtggctttatacgaggggatagc
GA2b.Bfra.CL05T12C13  99.7%  81.0% atggctgtttccatgaaagcggccattctgacagccgggaaaaaacgggaggaagcggttgccttgtatgacacgatggc
GA2c.Bvul.TF05-18     99.8%  82.0% atggccgtatccatgaaagcggcgattctgacagccgggaaaaaacgggaggaggcggttgccttgtatgaagggatggc
GA2d.Buni.BIOML-A83   99.2%  69.8% atggcggcttccatgaaggctgccatacttaccgcagaaaagaagcgtgacgaagcgatttcactatataaggagatagc
GA2e.Bsal.DSM.18170   99.7%  79.1% atggcaacttccatgaaagcggctgtcctgacggcagagaaaaaacgggaggaagcaattgcactgtatgagggactggc
consensus/100%                     ATGGCsusstCCATGAAuGCsGCsuTsCTsACsGCsGuuAAuAAuCGsGAsGAuGCuuTssCssTuTAsuAsususTuGC
consensus/90%                      ATGGCsusstCCATGAAuGCsGCsuTsCTsACsGCsGuuAAuAAuCGsGAsGAuGCuuTssCssTuTAsuAsususTuGC
consensus/80%                      ATGGCsGsTTCCATGAAuGCGGCsATsCTGACuGCsGuuAAuAAACGsGAuGAuGCGuTTGCssTuTATGAuGuGATuGC
consensus/70%                      ATGGCsGsTTCCATGAAuGCGGCsATsCTGACuGCsGuuAAuAAACGsGAuGAuGCGuTTGCssTuTATGAuGuGATuGC

                     cov     pid 17681.       .         .         .         .         .         .         .        . 17760
GA2a.Bcac.CL03T12C61 100.0% 100.0% cgggaaagctgtcgaacggaaggaccccttattatgtcatggagggctaccggatgtgcggcttcctgcggtacgaggagg
GA2b.Bfra.CL05T12C13  99.7%  81.0% taaaaaagccgtcatgcaaaaggacccttattatgtcatggaaggctatcgcatgtgtggtttcctgcgctatgaggatg
GA2c.Bvul.TF05-18     99.8%  82.0% acggaaagccatcacgcaaaaagacccttattatgtcatggaaggctaccgcatgtgtggtttcctgcgctacgaggatg
GA2d.Buni.BIOML-A83   99.2%  69.8% cttgaaagctgttgagcaaaaagatgcctactatgtgatggaaggttaccgtatgtgtggattcctgcgttatgaagagg
GA2e.Bsal.DSM.18170   99.7%  79.1% ggagaaagctatcggacaaaaagatccctactatgtcatggaaggttatcgtatgtgtggctttttacgctacgaggaag
consensus/100%                     sssuAAAGCsuTsusuCuuAAuGAssCsTAsTATGTsATGGAuGGsTAsCGsATGTGsGGsTTssTuCGsTAsGAuGAsG
consensus/90%                      sssuAAAGCsuTsusuCuuAAuGAssCsTAsTATGTsATGGAuGGsTAsCGsATGTGsGGsTTssTuCGsTAsGAuGAsG
consensus/80%                      ssuGAAAGCsuTCusuCAAAAuGAsCCsTAsTATGTCATGGAAGGsTAsCGsATGTGTGGsTTCCTGCGsTAsGAGGAsG
consensus/70%                      ssuGAAAGCsuTCusuCAAAAuGAsCCsTAsTATGTCATGGAAGGsTAsCGsATGTGTGGsTTCCTGCGsTAsGAGGAsG

                     cov     pid 17761.       .         .         .         .         .         .         .        . 17840
GA2a.Bcac.CL03T12C61 100.0% 100.0% gaaagctggaacaggcgttcgaatatttcctgctggcattggcgggaggaagctatctttccgaagacatcagacggagc
GA2b.Bfra.CL05T12C13  99.7%  81.0% gaaagatggaacaggctttcgaatattttttgcttgccctggcaggaggcagctacctttctgccgaaatcaggcggagt
GA2c.Bvul.TF05-18     99.8%  82.0% ggaagatggaacaggctttcgagtattttttgctcgccctgtccgggggggagttacctttccggggaaatcaggcggagt
GA2d.Buni.BIOML-A83   99.2%  69.8% gcaagatggaaagtgctttcgagttctttttactatcactggcaggcggaagttatcttcctgaaaacattagaagaaac
GA2e.Bsal.DSM.18170   99.7%  79.1% gccagctgaatcaggctttcgagtattttctgcttgctttggcagccgggagttatctttccgaagaaatcagacggaac
consensus/100%                     GssAGsTGuAssusGCsTTCGAuTssTTssTuCTssCssTGsCsGssGGsAGsTAsCTTsCsGssuAsATsAGusGuAus
consensus/90%                      GssAGsTGuAssusGCsTTCGAuTssTTssTuCTssCssTGsCsGssGGsAGsTAsCTTsCsGssuAsATsAGusGuAus
consensus/80%                      GsAAGsTGGAACAGGCTTTCGAuTATTTTsTGCTsGCssTGGCuGGsGGuAGsTAsCTTTCsGuuGAsATCAGuCGGAus
consensus/70%                      GsAAGsTGGAACAGGCTTTCGAuTATTTTsTGCTsGCssTGGCuGGsGGuAGsTAsCTTTCsGuuGAsATCAGuCGGAus

                     cov     pid 17841.       .         .         .         .         .         .         .        . 17920
GA2a.Bcac.CL03T12C61 100.0% 100.0% tccaccttcacctacgcggcacacctggcgctccatacgggcaggcaggtgcgtgcgccgattgacatcgaggtgctgga
GA2b.Bfra.CL05T12C13  99.7%  81.0% tccacgttcgtttatgcggcgtatctggctcttcatacgggaaggttggttcgtgctccaattgatgtcgggatattgga
GA2c.Bvul.TF05-18     99.8%  82.0% tccacgttcacttacgcggcacatctggctcttcacacaggaagacaggtacgttccccgcttgatgtctcgattttgga
GA2d.Buni.BIOML-A83   99.2%  69.8% tcgacatttacctatgccgcttatctcgctttgcatacaggtaaacaagtaagggctccttcagacatagaaatactcga
GA2e.Bsal.DSM.18170   99.7%  79.1% tccacgtttacttatgcggcatatctggcgctccacactggcaggcaggtacgtgcgccgattgatatggaggtcttgga
consensus/100%                     TCsACsTTsussTAsGCsGCssAsCTsGCssTsCAsACsGGsAuussuGTssGssCsCCssssGAsuTsssuuTssTsGA
consensus/90%                      TCsACsTTsussTAsGCsGCssAsCTsGCssTsCAsACsGGsAuussuGTssGssCsCCssssGAsuTsssuuTssTsGA
consensus/80%                      TCCACuTTsACsTAsGCGGCusATCTGGCsCTsCAsACuGGsAGuCAGGTuCGTGCsCCusTTGAsuTsGuGuTssTGGA
consensus/70%                      TCCACuTTsACsTAsGCGGCusATCTGGCsCTsCAsACuGGsAGuCAGGTuCGTGCsCCusTTGAsuTsGuGuTssTGGA
```

```
                       cov     pid 17921.       .         .         .         .         .         .         .         . 18000
GA2a.Bcac.CL03T12C61 100.0% 100.0% acgccagcttcacgaatggctgggcgaggactggaaggaactggtggagaacccctccatgcggcaggcggaagccagaa
GA2b.Bfra.CL05T12C13  99.7%  81.0% acggcagctctgcgaatggattgggaggattggaaggagctggtggacaatccggatatgcagcaggcgaaggccagaa
GA2c.Bvul.TF05-18     99.8%  82.0% aggacagctccgggagtggctcggggacgactggaaggaattggtggacaatccggacatgcagctggcgaaggccagaa
GA2d.Buni.BIOML-A83   99.2%  69.8% aaaacaattacaggaatggcttggcaaagattggagggagcttgttgataacccatccatgcgacaagccaaggccagaa
GA2e.Bsal.DSM.18170   99.7%  79.1% agggcagcttcaggaatggctgggagaagattggaaacaactggtggagggtccggccatgcagcaggcaaaagccagaa
consensus/100%                     AsusCAusTssusGAuTGGsTsGGsuAsGAsTGGAuusAusTsGTsGAsuusCCssssATGCuuCsuGCsuAuGCCAGAA
consensus/90%                      AsusCAusTssusGAuTGGsTsGGsuAsGAsTGGAuusAusTsGTsGAsuusCCssssATGCuuCsuGCsuAuGCCAGAA
consensus/80%                      AsGuCAGCTsCusGAATGGCTsGGsGAuGAsTGGAAGGAuCTGGTGGAsAAsCCussCATGCuGCAGGCuAAuGCCAGAA
consensus/70%                      AsGuCAGCTsCusGAATGGCTsGGsGAuGAsTGGAAGGAuCTGGTGGAsAAsCCussCATGCuGCAGGCuAAuGCCAGAA
```

```
                       cov     pid 18001.       .         .         .         .         .         .         .         . 18080
GA2a.Bcac.CL03T12C61 100.0% 100.0% gaaaaggggtattttcaactgaaagagaatttaatcaacat-atagtttaaacttaaatatcga-atttatggatcttg
GA2b.Bfra.CL05T12C13  99.7%  81.0% gaaggggaagtttttcagttgagcgag-atttaattaatat-atatttaaatttaaaattgaataagtatggatcttg
GA2c.Bvul.TF05-18     99.8%  82.0% gaaaaggggtattttcaattgacagag-atttaattaacat-atattttaatttaaaattgaataagtatggaccttg
GA2d.Buni.BIOML-A83   99.2%  69.8% gaaagaagaacatattcagttaaaatcaagagaaatagttaacatattttaatttaaaattagaatagtatggaacttg
GA2e.Bsal.DSM.18170   99.7%  79.1% gaaaaagtaagattttcggatgagtgag-atttactaacata-tttttttaattataaaacagattaagtatggatttag
consensus/100%                     GAAuuuusuussTsTTCuusTuAssssu.usssAsTsussss.sTssTTTsAsssTAAAssssus.sssTATGGAssTsG
consensus/90%                      GAAuuuusuussTsTTCuusTuAssssu.usssAsTsussss.sTssTTTsAsssTAAAssssus.sssTATGGAssTsG
consensus/80%                      GAAAuuGuuusATTTTCAusTGAusGAG.ATTTAATsAssAs.ATATTTTsAATTTAAAAsTuuAsAAGTATGGAsCTTG
consensus/70%                      GAAAuuGuuusATTTTCAusTGAusGAG.ATTTAATsAssAs.ATATTTTsAATTTAAAAsTuuAsAAGTATGGAsCTTG
```

```
                       cov     pid 18081.       .         .         .         .         .         .         .         . 18160
GA2a.Bcac.CL03T12C61 100.0% 100.0% gaaattacggaatcggcggaaacgaagtgaagattgacgcctccgaaggcattgcggaaattccacaaaaccggacatta
GA2b.Bfra.CL05T12C13  99.7%  81.0% gtaattatggaatcggcggaaacgaagtgaagattgacgcttccgaaggtattgcggaaattccacaaaatcgaacctta
GA2c.Bvul.TF05-18     99.8%  82.0% gaaattatggaatcggcggaaacgaggtgaagattgacgcttccgaaggtattgcggaaattccacaaaaccgaacgtta
GA2d.Buni.BIOML-A83   99.2%  69.8% gaaattatggaatcggcggaaacgaggtaaaactcgaagcatccgaaggtattgcggatattccacaaaaccgaacttta
GA2e.Bsal.DSM.18170   99.7%  79.1% gaaattacggaataggcggaaatgaagtgaaaatggatgcctccgaaggtattgcggaaattccacagaaccggacatta
consensus/100%                     GsAATTAsGGAATsGGCGGAAAsGAuGTuAAusTsGAsGCsTCCGAAGGsATTGCGGAsATTCCACAuAAsCGuACsTTA
consensus/90%                      GsAATTAsGGAATsGGCGGAAAsGAuGTuAAusTsGAsGCsTCCGAAGGsATTGCGGAsATTCCACAuAAsCGuACsTTA
consensus/80%                      GAAATTAsGGAATCGGCGGAAACGAuGTGAAuATsGAsGCsTCCGAAGGTATTGCGGAAATTCCACAAAACCGuACsTTA
consensus/70%                      GAAATTAsGGAATCGGCGGAAACGAuGTGAAuATsGAsGCsTCCGAAGGTATTGCGGAAATTCCACAAAACCGuACsTTA
```

```
                       cov     pid 18161.       .         .         .         .         .         .         .         . 18240
GA2a.Bcac.CL03T12C61 100.0% 100.0% ttggtggagcagttgactaaagacgaaccggtggctcccgaagtgattacggggctgaccaatatcgaccaggtcttcgc
GA2b.Bfra.CL05T12C13  99.7%  81.0% ttggtggaacagctgacgaaggatgagccggtggctcccgaagtggttgcaggtctgaccaacattgacgaagtctttgc
GA2c.Bvul.TF05-18     99.8%  82.0% ttggtggaacagttgacaaaagacgaaccggtggctcccgaagtgatcgcaggcttgaccaacatcgacgaagtgtttgc
GA2d.Buni.BIOML-A83   99.2%  69.8% atggttgagcaattgacaaaggacgaaccggtagcacctgaagttgtaacaggtcttaccaacattgacgaagtctttgc
GA2e.Bsal.DSM.18170   99.7%  79.1% ttggttgaacaattgacaaaggacgaaccggtagctcccgaagtggtcatcggtctgactagcattgacgaagtctttgc
consensus/100%                     sTGGTsGAuCAusTGACsAAuGAsGAuCCGGTuGCsCCsGAAGTsuTsussGGssTsACsAusATsGACsAuGTsTTsGC
consensus/90%                      sTGGTsGAuCAusTGACsAAuGAsGAuCCGGTuGCsCCsGAAGTsuTsussGGssTsACsAusATsGACsAuGTsTTsGC
consensus/80%                      TTGGTsGAuCAuTTGACuAAuGACGAACCGGTuGCTCCCGAAGTGuTsuCuGGsCTGACCAACATsGACGAAGTCTTTGC
consensus/70%                      TTGGTsGAuCAuTTGACuAAuGACGAACCGGTuGCTCCCGAAGTGuTsuCuGGsCTGACCAACATsGACGAAGTCTTTGC
```

```
                            cov    pid 18241.       .        .        .        .        .        .        .        . 18320
GA2a.Bcac.CL03T12C61 100.0% 100.0% gcatttcttaccggagatagatattgagttcaccgatgcggaaggtgctcccgtggaggaaaacttccgttttcacaatg
GA2b.Bfra.CL05T12C13  99.7%  81.0% acatttccggccggaaatagatatcgagttcgcggatgcggacggtgctcccgtagaggaaaatttccgtttccacaatg
GA2c.Bvul.TF05-18     99.8%  82.0% acatttccggccggaaatagatatcgagttctcggatgcggagggggctcccgtagaggaaaatttccgttttcaaaatg
GA2d.Buni.BIOML-A83   99.2%  69.8% acatttccagccggaaatagatattgagttctctgatgcggacggagcaccagtcgaggagaactttcgttttaacaatg
GA2e.Bsal.DSM.18170   99.7%  79.1% acatttccagccggaaattgatatagagttcgctgatgcggaaggggctcctgttgaagagaacttccgtttccacaatg
consensus/100%                     uCATTTCssuCCGGAuATsGATATsGAGTTCsCsGATGCGGAsGGsGCsCCsGTsGAuGAuAAsTTsCGTTTssAsAATG
consensus/90%                      uCATTTCssuCCGGAuATsGATATsGAGTTCsCsGATGCGGAsGGsGCsCCsGTsGAuGAuAAsTTsCGTTTssAsAATG
consensus/80%                      ACATTTCCuGCCGGAAATAGATATsGAGTTCsCsGATGCGGAsGGsGCTCCsGTsGAGGAuAAsTTCCGTTTsCACAATG
consensus/70%                      ACATTTCCuGCCGGAAATAGATATsGAGTTCsCsGATGCGGAsGGsGCTCCsGTsGAGGAuAAsTTCCGTTTsCACAATG

                            cov    pid 18321.       .        .        .        .        .        .        .        . 18400
GA2a.Bcac.CL03T12C61 100.0% 100.0% tgggggacttttctgtcaagaaaatgacggagcaaagtcatttcctgtcaggactgaatacggaaaagcagttcgccgac
GA2b.Bfra.CL05T12C13  99.7%  81.0% tcggtgattttctgtcaggaaaatgacggagcagagttcattcttgtccggcctgaatgcggaaaagcagtttgcggac
GA2c.Bvul.TF05-18     99.8%  82.0% tcggcgattttctgtcaagaagatgacggaacagagtcctttcttgtccggactgaatgcggagaaacagtttgcggac
GA2d.Buni.BIOML-A83   99.2%  69.8% ttggtgacttttctgttgccaagatgacagaacatagtccttttctatccagtctgaatgctgaaaaacaatttgcagac
GA2e.Bsal.DSM.18170   99.7%  79.1% tgggagatttctccgtgaagaaaatgacggaacagagtcctttcctgtcggggctgaatgcggaaaaacagtttgctgac
consensus/100%                     TsGGsGAsTTsTCsGTsussAAuATGACuGAuCAsAGTsssTTssTuTCsuGsCTGAATuCsGAuAAuCAuTTsGCsGAC
consensus/90%                      TsGGsGAsTTsTCsGTsussAAuATGACuGAuCAsAGTsssTTssTuTCsuGsCTGAATuCsGAuAAuCAuTTsGCsGAC
consensus/80%                      TsGGsGAsTTTTCTGTsAuGAAuATGACGGAuCAuAGTCCTTTCsTGTCsGGsCTGAATGCGGAAAAuCAGTTTGCsGAC
consensus/70%                      TsGGsGAsTTTTCTGTsAuGAAuATGACGGAuCAuAGTCCTTTCsTGTCsGGsCTGAATGCGGAAAAuCAGTTTGCsGAC

                            cov    pid 18401.       .        .        .        .        .        .        .        . 18480
GA2a.Bcac.CL03T12C61 100.0% 100.0% aggatggagaagtcgttgcgctccaacaaggtgctgcaacgggtattggagaatccggcgacacgcgaggcattcgtcaa
GA2b.Bfra.CL05T12C13  99.7%  81.0% aggatggagaagtcgttgcgttccaacaaggtgctgcaacgggtgctggaaaatccggagacacgtggagcgtttgtcaa
GA2c.Bvul.TF05-18     99.8%  82.0% aggatggagaagtcattgcgctccaacaaggtgttgcagcgggtactggaaaatccggagacacgcggggctttcgtcaa
GA2d.Buni.BIOML-A83   99.2%  69.8% agaatggaaaagtctttgcgttcgaacaaggttctgcaacgggttctggagaatcccactacacgtgacgcctttgtcaa
GA2e.Bsal.DSM.18170   99.7%  79.1% aggatggagaagtcgttacggtctaacaaggtgctgcaacgggttctggaaaacccggctacacgcaaggcgttcgtcaa
consensus/100%                     AGuATGGAuAAGTCsTTuCGsTCsAACAAGGTssTGCAuCGGGTssTGGAuAAsCCsussACACGsuusGCsTTsGTCAA
consensus/90%                      AGuATGGAuAAGTCsTTuCGsTCsAACAAGGTssTGCAuCGGGTssTGGAuAAsCCsussACACGsuusGCsTTsGTCAA
consensus/80%                      AGGATGGAGAAGTCuTTGCGsTCsAACAAGGTGCTGCAACGGGTsCTGGAuAATCCGGssACACGsGuuGCsTTsGTCAA
consensus/70%                      AGGATGGAGAAGTCuTTGCGsTCsAACAAGGTGCTGCAACGGGTsCTGGAuAATCCGGssACACGsGuuGCsTTsGTCAA

                            cov    pid 18481.       .        .        .        .        .        .        .        . 18560
GA2a.Bcac.CL03T12C61 100.0% 100.0% ttcgctttctgccatgctggacgatttaaagaaaaacgagaagtctaaccctgcaaatgaatgacaacagctatgagtga
GA2b.Bfra.CL05T12C13  99.7%  81.0% tgcgatttctgccattttggacgatttgaagaacaatgagaagtctaaccccactaatgaatg---atagttatgagcga
GA2c.Bvul.TF05-18     99.8%  82.0% tgcgatttccgccattctggacgatttgaagaacaacgagaagtctaatcctgctaatgaatg---atagttatgagcga
GA2d.Buni.BIOML-A83   99.2%  69.8% tgcactttcagtcatgctggacgaactgaaaaacaacgaaaagtctaactctgtaaatgaatg---atagttatgagtga
GA2e.Bsal.DSM.18170   99.7%  79.1% tacactgtctatcatgttggacgaattaaaggaaaatgagaagtctaaccctgtaaatgaatg---agaaccatgagcga
consensus/100%                     TsCusTsTCsusCATssTGGACGAssTuAAuuAsAAsGAuAAGTCTAAssCsussAATGAATG...AsAussATGAGsGA
consensus/90%                      TsCusTsTCsusCATssTGGACGAssTuAAuuAsAAsGAuAAGTCTAAssCsussAATGAATG...AsAussATGAGsGA
consensus/80%                      TuCusTTTCsGsCATssTGGACGAsTTuAAGAAsAAsGAGAAGTCTAACCCTGssAATGAATG...AsAGsTATGAGsGA
consensus/70%                      TuCusTTTCsGsCATssTGGACGAsTTuAAGAAsAAsGAGAAGTCTAACCCTGssAATGAATG...AsAGsTATGAGsGA
```

```
                  cov    pid 18561.       .         .         .         .         .         .         .         . 18640
GA2a.Bcac.CL03T12C61 100.0% 100.0%  acagaacacacacagggaggcagccgtacaacaacagcaggctgcggcacagtccagaaacctgaacgcggagctacagg
GA2b.Bfra.CL05T12C13  99.7%  81.0%  acagaatacacaaagagaagcagccatacagcagcaacaggctgtggcgcagtccagaaatctgaacgctgaattgcaga
GA2c.Bvul.TF05-18     99.8%  82.0%  acagaatacacaaagagaagctgccgtacagcagcaacaggctgtggcgcagtccagaaatctgaatgcagaattgcagg
GA2d.Buni.BIOML-A83   99.2%  69.8%  acagaatcctaaaatagaagctgcgg---aacaacaagttgctgccgtacaaaccagaaacttaaatactgagttacagg
GA2e.Bsal.DSM.18170   99.7%  79.1%  acagaacacacagaaggaaaccgcagtacagcaacagcaggctgcggtacaaaccaaaaacttgaatgcggagttacagg
consensus/100%                      ACAGAAssCssAsAsuGAuuCsGCsu...AuCAuCAusssGCTGssGsuCAusCCAuAAAssTuAAsuCsGAusTuCAGu
consensus/90%                       ACAGAAssCssAsAsuGAuuCsGCsu...AuCAuCAusssGCTGssGsuCAusCCAuAAAssTuAAsuCsGAusTuCAGu
consensus/80%                       ACAGAAsACACAuAuuGAAGCsGCsGTACAuCAuCAuCAGGCTGsGGsuCAusCCAGAAAssTGAAsGCsGAuTTuCAGG
consensus/70%                       ACAGAAsACACAuAuuGAAGCsGCsGTACAuCAuCAuCAGGCTGsGGsuCAusCCAGAAAssTGAAsGCsGAuTTuCAGG

                  cov    pid 18641.       .         .         .         .         .         .         .         . 18720
GA2a.Bcac.CL03T12C61 100.0% 100.0%  tcctggctaagtttggtggtttcaatgtcctggagtcctctgtggacggcatacagaacctgaacccggagcgcaaggcg
GA2b.Bfra.CL05T12C13  99.7%  81.0%  ttctggctaagtttggcggtttcaatgtcttggaatcttccgtggacggcatacagaacatgaatccggagcgtaaggca
GA2c.Bvul.TF05-18     99.8%  82.0%  ttctggctaagtttggaggttttaatgtcctggaatcttccgtggacggcatacagaacctgaacccggaacgtaaggcg
GA2d.Buni.BIOML-A83   99.2%  69.8%  ctcttgccaaatttggtggtttcaatgtattggaatcttctgtagatggaatacagaacctcaatccagagcgtaaggca
GA2e.Bsal.DSM.18170   99.7%  79.1%  ctttggcaaagttcggaggtttcaacgtcttggaatcttcggtggacggcatacagaatttgaatccggaacgtaaggca
consensus/100%                      sssTsGCsAAuTTsGGsGGTTTsAAsGTssTGGAuTCsTCsGTuGAsGGsATACAGAAssTsAAsCCuGAuCGsAAGGCu
consensus/90%                       sssTsGCsAAuTTsGGsGGTTTsAAsGTssTGGAuTCsTCsGTuGAsGGsATACAGAAssTsAAsCCuGAuCGsAAGGCu
consensus/80%                       sTCTGGCsAAGTTTGGsGGTTTCAATGTCsTGGAATCTTCsGTGGACGGCATACAGAACsTGAAsCCGGAuCGTAAGGCu
consensus/70%                       sTCTGGCsAAGTTTGGsGGTTTCAATGTCsTGGAATCTTCsGTGGACGGCATACAGAACsTGAAsCCGGAuCGTAAGGCu

                  cov    pid 18721.       .         .         .         .         .         .         .         . 18800
GA2a.Bcac.CL03T12C61 100.0% 100.0%  cgccgtaacattttccttacagaccccgaacgggcgcctgaacgcaaggagctggagaaacggctcgaaatgtgggtgga
GA2b.Bfra.CL05T12C13  99.7%  81.0%  cggcggaacatattcctttccgaccccgaacgggcggctgagcgcaaggagctggaaaaacggcttgagatgtgggtcga
GA2c.Bvul.TF05-18     99.8%  82.0%  cggcggaacatattcctttccgatcctgaacgggcgggcgagcgcaaggaactgaaaaaacggctggaaatgtggctgga
GA2d.Buni.BIOML-A83   99.2%  69.8%  agacgcaatatctatttgaccagtaatgaaaaagcggctgaacgtaaagaacttgcaaagagtattgaaatgtgggtaga
GA2e.Bsal.DSM.18170   99.7%  79.1%  cgccggaatatattccttactgaccgggaacgggctaacgagcgtaaggaactgagaaaaaggattgagatgtggataga
consensus/100%                      sGsCGsAAsATsTsssTssCsuusssssGAAsuuGCssssGAuCGsAAuGAuCTsusuAAusGssTsGAuATGTGGsTsGA
consensus/90%                       sGsCGsAAsATsTsssTssCsuusssssGAAsuuGCssssGAuCGsAAuGAuCTsusuAAusGssTsGAuATGTGGsTsGA
consensus/80%                       CGsCGsAAsATsTTCCTTsCsGAsCssGAACGGGCGussGAuCGsAAGGAuCTGuuAAAAsGGsTsGAuATGTGGuTuGA
consensus/70%                       CGsCGsAAsATsTTCCTTsCsGAsCssGAACGGGCGussGAuCGsAAGGAuCTGuuAAAAsGGsTsGAuATGTGGuTuGA

                  cov    pid 18801.       .         .         .         .         .         .         .         . 18880
GA2a.Bcac.CL03T12C61 100.0% 100.0%  catgctgcaaggcaacaagtccatcagcgaaatgattgaactgtgccagaagaaatcggcggccgtctccgatctgcttt
GA2b.Bfra.CL05T12C13  99.7%  81.0%  tatgttgaaaggcaacaaatccatcagcgagatggtggaactgtgccagaagaagtctgcggcggtgtccggtctgctat
GA2c.Bvul.TF05-18     99.8%  82.0%  tatgctggagggtaacaaatccatcagcgagatggtggaattgtgccagaagaaatcggcggcggtatccggtctgcttt
GA2d.Buni.BIOML-A83   99.2%  69.8%  aatgctgaaaagcggcaaaactatcacagaaatgattgatgcttgtcagaaaaagtcagcagctgtatcagatttacttt
GA2e.Bsal.DSM.18170   99.7%  79.1%  tatgctgaaagacggcaagtccatcagcgaaatggtggatttgtgccagaaaaagtctgctgccgtctctgacctgctct
consensus/100%                      sATGsTGsAuuusuuCAAusCsATCAssGAuATGuTsGAssssTGsCAGAAuAAuTCsGCsGCsGTsTCsGussTuCTsT
consensus/90%                       sATGsTGsAuuusuuCAAusCsATCAssGAuATGuTsGAssssTGsCAGAAuAAuTCsGCsGCsGTsTCsGussTuCTsT
consensus/80%                       sATGCTGuAAGGCuuCAAuTCCATCAGCGAuATGuTsGAssTGTGCCAGAAuAAuTCsGCuGCsGTsTCsGuTCTGCTsT
consensus/70%                       sATGCTGuAAGGCuuCAAuTCCATCAGCGAuATGuTsGAssTGTGCCAGAAuAAuTCsGCuGCsGTsTCsGuTCTGCTsT
```

```
                      cov    pid 18881.         .         .         .         .         .         .         .         . 18960
GA2a.Bcac.CL03T12C61 100.0% 100.0%   cccagaaccagctgaaggcggtggaagaggtgaaacaattggagaagtcataccgtacggtcatgcttttctacaagaat
GA2b.Bfra.CL05T12C13  99.7%  81.0%   cccagaaccagctgaaagcggtggaagaggtcaggcagttggagaagtcctaccgcacggtcatgctcttctacaagaat
GA2c.Bvul.TF05-18     99.8%  82.0%   cccaaaaccagttgaaggcggtagaggaggtcaagcagttggagaagtcttaccgcacggtcatgctcttctacaagaat
GA2d.Buni.BIOML-A83   99.2%  69.8%   cacaaaaccaacttaaagcggtagaggaagtcaaacaattggaaaaatcgtaccgtacagtcatgctcttctacaagaac
GA2e.Bsal.DSM.18170   99.7%  79.1%   ctcaaaatcagctgaaggcggtagaagaagtgaaacaattggaaaaatcataccgcacggtcatgcttttctacaagaat
consensus/100%                       CsCAuAAsCAusTsAAuGCGGTuGAuGAuGTsAuuCAuTTGGAuAAuTCsTACCGsACuGTCATGCTsTTCTACAAGAAs
consensus/90%                        CsCAuAAsCAusTsAAuGCGGTuGAuGAuGTsAuuCAuTTGGAuAAuTCsTACCGsACuGTCATGCTsTTCTACAAGAAs
consensus/80%                        CsCAuAACCAGCTGAAuGCGGTuGAuGAuGTsAAuCAuTTGGAuAAuTCsTACCGsACGGTCATGCTsTTCTACAAGAAT
consensus/70%                        CsCAuAACCAGCTGAAuGCGGTuGAuGAuGTsAAuCAuTTGGAuAAuTCsTACCGsACGGTCATGCTsTTCTACAAGAAT

                      cov    pid 18961.         .         .         .         .         .         .         .         . 19040
GA2a.Bcac.CL03T12C61 100.0% 100.0%   acggattccgacaagattaagaacatttccatcgtcaatgcttccatggagcaactcaccgacctggacaactctttctt
GA2b.Bfra.CL05T12C13  99.7%  81.0%   acggattccgacaagatcaagaatatctccattgtgaacgcttcgatgagccagcttaccgatttggacaactccttctt
GA2c.Bvul.TF05-18     99.8%  82.0%   acggattccgacaagatcaagaacatctccatcgtgaacgcttcgatggaccagcttaccgatttggacaattctttctt
GA2d.Buni.BIOML-A83   99.2%  69.8%   actgactcggacaaaatcaagaatgtttccattgtcaatgcatctatggctcagcttacagatcttgacaactcattctt
GA2e.Bsal.DSM.18170   99.7%  79.1%   acggatg
```
